# A De Novo Metalloenzyme for Cerium Photoredox Catalysis

**DOI:** 10.1021/jacs.4c04618

**Published:** 2024-08-08

**Authors:** Andreas Sebastian Klein, Florian Leiss-Maier, Rahel Mühlhofer, Benedikt Boesen, Ghulam Mustafa, Hannah Kugler, Cathleen Zeymer

**Affiliations:** † Center for Functional Protein Assemblies & Department of Bioscience, TUM School of Natural Sciences, Technical University of Munich (TUM), 85748 Garching, Germany; ‡ TUM Catalysis Research Center, Technical University of Munich (TUM), 85748 Garching, Germany

## Abstract

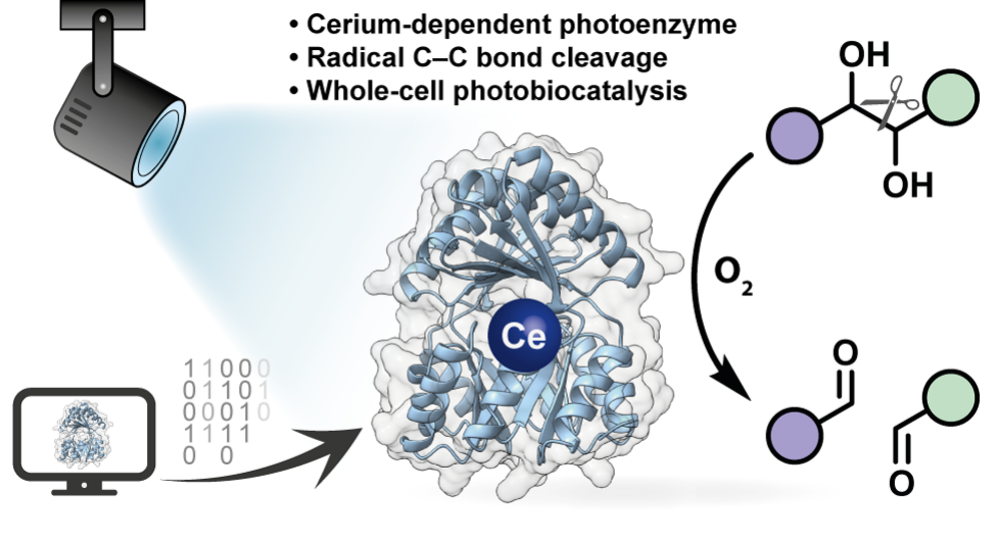

Cerium photoredox catalysis has emerged as a powerful strategy to activate molecules under mild conditions. Radical intermediates are formed using visible light and simple complexes of the earth-abundant lanthanide. Here, we report an artificial photoenzyme enabling this chemistry inside a protein. We utilize a de novo designed protein scaffold that tightly binds lanthanide ions in its central cavity. Upon visible-light irradiation, the cerium-dependent enzyme catalyzes the radical C–C bond cleavage of 1,2-diols in aqueous solution. Protein engineering led to variants with improved photostability and metal binding behavior. The photoenzyme cleaves a range of aromatic and aliphatic substrates, including lignin surrogates. Surface display of the protein scaffold on *Escherichia coli* facilitates whole-cell photobiocatalysis. Furthermore, we show that also natural lanthanide-binding proteins are suitable for this approach. Our study thus demonstrates a new-to-nature enzymatic photoredox activity with broad catalytic potential.

## Introduction

Enzymes are highly efficient, selective, and sustainable catalysts. Their use in chemical and pharmaceutical industry increased substantially in recent years.^1^ However, it remains challenging to develop enzymes that catalyze chemical reactions beyond nature’s synthetic repertoire. To broaden the scope of biocatalysis, artificial metalloenzymes and photoenzymes have been generated by combining the catalytic strengths of transition metal cofactors and photosensitizers, respectively, with the selectivity of the chiral protein environment.^2,3^ As these catalysts are genetically encodable, protein engineering can be applied to optimize efficiency and selectivity. To that end, rational enzyme design is often combined with subsequent directed evolution, which mimics natural selection in the laboratory.^4,5^ Recent highlights include hetero-Diels–Alder reactions or [2 + 2] photocycloadditions catalyzed efficiently and with tight stereocontrol in a designed metalloenzyme or photoenzyme, respectively.^6−8^

Transition metal-dependent photoredox catalysis is a versatile strategy for the direct activation and functionalization of organic molecules using visible light.^9^ Only recently, however, the field has started to exploit the photocatalytic potential of lanthanides.^10,11^ These elements from the f-block of the periodic table mainly exist as trivalent cations that form structurally diverse complexes with high coordination numbers (CN = 8 to 12). Cerium has slightly different chemical and photophysical properties compared to the other elements of the 4f series. It is stable in two oxidation states (+III/+IV) and undergoes electronic transitions tunable by the chemical environment, which makes it attractive for photoredox catalysis. Compared to iridium or ruthenium used in classic photoredox catalysts, cerium is >10,000-fold more abundant in the earth’s crust (ca. 65 ppm, similar to copper)^12^ and may therefore be an ecologically and economically meaningful alternative. The emerging field of lanthanide photocatalysis is thus dominated by cerium photoredox chemistry, which offers two distinct mechanisms to generate reactive radical intermediates: (i) single electron transfer (SET) from Ce(III) in its excited state and (ii) photoinduced ligand-to-metal charge transfer (LMCT) in Ce(IV) complexes.

Recent studies have demonstrated the photocatalytic power and versatility of cerium complexes. For instance, the hexachlorocerate(III) anion [Ce^III^Cl_6_]^3–^ is one of the most potent photoreductants.^13^ It enables the reductive dehalogenation of chloroarenes and catalyzes Miyaura-type borylations of aryl halides under mild conditions.^14^ Furthermore, the alkylation, arylation, and amination of unactivated alkanes was achieved using cerium photoredox catalysis based on LMCT.^15,16^ Mechanistic studies proposed either chlorine radicals or alkoxy radicals to be the catalytically relevant species.^17,18^ A variety of other photocatalytic transformations based on alkoxy radical mediated C–C bond cleavage has been reported previously and served as an inspiration for our work.^19−23^

While such reactive radicals can be generated under mild conditions, only very few catalytic systems have been reported to control these species and achieve stereoselectivity.^25^ We hypothesized that this challenge may be tackled by performing cerium photoredox chemistry inside an enzyme.

**Figure 1:**
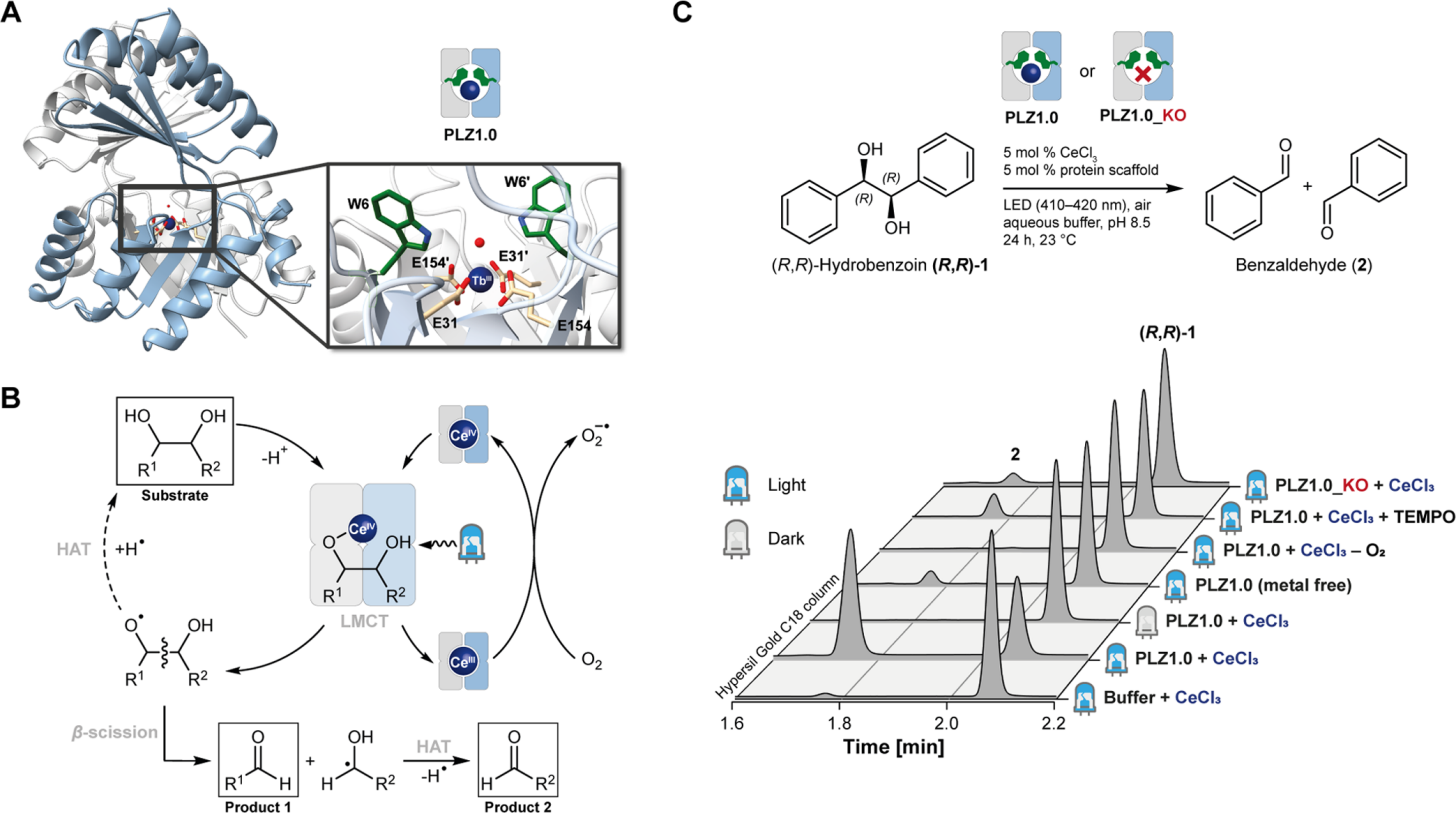
Cerium photoredox catalysis in a lanthanide-binding de novo protein. (A) Crystal structure of the TFD scaffold (= PLZ1.0 or *PhotoLanZyme* 1.0), here shown in complex with Tb(III). The dimeric TIM barrel scaffold possesses a lanthanide binding site that consists of 2 × 2 coordinating glutamate residues. Close-by tryptophan residues were installed to measure lanthanide binding via sensitized Tb(III) luminescence. PDB entry: 6ZV9.^24^ (B) Proposed mechanism for a radical 1,2-diol cleavage catalyzed by a Ce(III/IV)-dependent artificial photoenzyme. LMCT = ligand-to-metal charge transfer, HAT = hydrogen atom transfer. (C) Proof of concept: Cerium-bound PLZ1.0 catalyzes the photocatalytic cleavage of hydrobenzoin (**1**) to benzaldehyde (**2**). The reaction was monitored by HPLC. Controls include samples without protein, without irradiation, without CeCl_3_, without oxygen, with TEMPO as a radical scavenger, and using a metal binding deficient knockout variant (PLZ1.0_KO).

Computationally designed proteins are well suited for artificial metalloenzyme engineering, as their structure and geometry can be precisely tailored to enable selective metal binding.^26,27^ These scaffolds are often also highly robust and, at the same time, evolutionarily unbiased. We recently explored de novo (βα)_8_ barrels,^28^ so-called triose-phosphate isomerase (TIM) barrels, as a potential platform for lanthanide binding.^24^ To design an optimal coordination environment, we drew inspiration from nature, where lanthanide-dependent proteins and enzymes from certain methylotrophic bacteria possess binding sites with a spherical arrangement of several coordinating carboxy groups.^29−31^ We thus installed four glutamate residues in the center of a symmetric de novo TIM barrel, which is formed from two (βα)_4_ half-barrels tethered to a lid domain (TIM-ferredoxin dimer = TFD).^24^ Its specific lanthanide coordination was demonstrated by tryptophan-enhanced terbium(III) luminescence and X-ray crystallography (Figure 1A). The dimeric de novo protein binds lanthanides with femtomolar affinity^32^ and remains folded at up to 95 °C. It possesses an internal cavity above the metal binding site that could serve as a reaction chamber. With these properties, it provided an ideal starting point for us to engineer the first lanthanide-dependent photoenzyme.

## Results

### Photoenzymatic C–C Bond Cleavage of 1,2-Diols

To demonstrate the feasibility of cerium photoredox catalysis in our de novo protein, we chose the radical C–C bond cleavage of 1,2-diols as a model reaction. This transformation was recently reported to occur photocatalytically in the presence of CeCl_3_ in organic solvent with visible light.^22^ It is proposed to proceed via β-scission of alkoxy radicals formed upon photoinduced LMCT, while the regeneration of Ce(IV) requires SET to atmospheric oxygen (Figure 1B). The cleavage of hydrobenzoin (**1**) in aqueous buffer with CeCl_3_ yielded only trace amounts of the product benzaldehyde (**2**). In contrast, our lanthanide- binding TFD protein, now dubbed “*PhotoLanZyme* 1.0” (**PLZ1.0**), was indeed capable of catalyzing this reaction (Figure 1C). We found that wavelengths of 420 nm and below gave the highest yields, which is in agreement with the absorption of Ce(IV) in aqueous buffer (Figure S2). Furthermore, light on/off kinetics showed that the reaction only proceeds in the light (Figure S6). As the recycling of Ce(IV) may be a limiting factor, we set up reactions with (NH_4_)_2_Ce(NO_3_)_6_ instead of CeCl_3_ and also tested the addition of 9,10-diphenylanthracene (DPA) as a mediator. However, both strategies did not increase the amount of product formation (Figure S7).

Importantly, no significant turnover was observed for all negative controls: in the absence of light, CeCl_3_ or oxygen, and when using a knockout variant (**PLZ1.0_KO**) in which all coordinating glutamates were replaced by glutamines (Figure 1C). The low background activity in the absence of CeCl_3_ may be due to traces of other metal ions in the sample or metal-independent oxidative processes on the protein surface upon irradiation. To support the proposed radical mechanism, we performed the reaction in the presence of (2,2,6,6-tetramethyl-piperidin-1-yl)oxyl (TEMPO) as a radical scavenger, leading to significantly reduced product formation. These results demonstrate that catalysis in aqueous solution requires cerium, light, oxygen, and specific metal coordination inside the protein.

### Protein Engineering to Improve Photostability and Metal Binding Kinetics

Next, we set out to improve the photoenzyme by protein engineering. To be able to generate asymmetric enzyme variants, we engineered a single-chain version of the protein scaffold, named **PLZ1.1**. To that end, the C-terminus of one subunit of the homodimeric **PLZ1.0** was covalently linked via a single glycine with the N-terminus of the other one.

**Figure 2:**
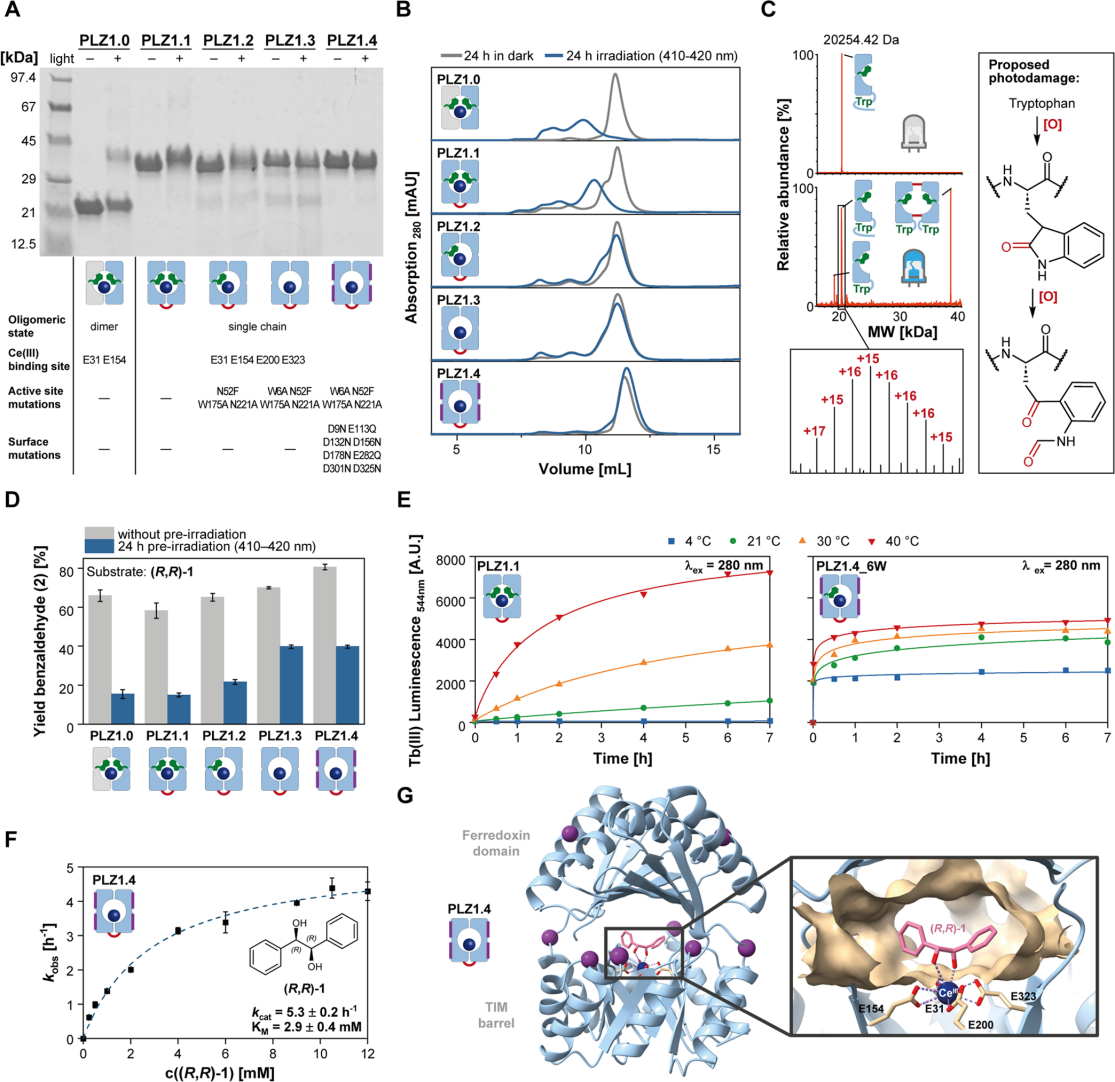
Optimization of photostability and metal binding behavior by protein engineering. (A, B) Photodamage of enzyme variants PLZ1.1 to PLZ1.4 after overnight irradiation in the presence of CeCl_3_ monitored by SDS-PAGE (A) and analytical size exclusion chromatography (B). PLZ1.1 was generated by fusing the C-terminus of one monomer to the N-terminus of the other monomer. In PLZ1.2 and PLZ1.3, active-site tryptophans W6 and W175 were sequentially removed. PLZ1.4 contains further mutations on the protein surface. (C) Photodamage analysis by protein mass spectrometry of PLZ1.0 before and after irradiation in the presence of CeCl_3_. (D) Photocatalytic performance of PLZ variants with and without preirradiation. (E) Influence of temperature and surface engineering on lanthanide binding kinetics measured by tryptophan-enhanced Tb(III) luminescence. Note: One of the previously removed active-site tryptophans (W6) had to be reintroduced into PLZ1.4 for these measurements. (F) Michaelis–Menten kinetics for the photoenzymatic cleavage of **(*****R***,***R*****)-1**. (G) AlphaFold2 model of PLZ1.4 and substrate docking for **(*****R***,***R*****)-1**. Purple spheres indicate surface mutations to suppress unspecific metal binding.

When analyzing the enzymes’ performance, we identified three main problems: (i) the protein suffered from severe photodamage, (ii) cerium binding to the active site was very slow and (iii) a substantial fraction of metal interacted unspecifically with the protein surface. We hypothesized that photodamage may be initiated by the photooxidation of tryptophans, which is a well-known photochemical process (Figure S8).^33,34^ Two tryptophans were originally installed directly next to the lanthanide binding site to facilitate tryptophan-enhanced terbium luminescence as a readout for metal binding. We sequentially mutated these positions, generating **PLZ1.2** and **PLZ1.3**. Furthermore, these variants carry two additional mutations rationally introduced to increase the asymmetry of the active site with the intention to enable stereocontrol. We characterized the photodamage of all cerium-bound PLZ variants after overnight irradiation using gel electrophoresis (SDS-PAGE), analytical size-exclusion chromatography (SEC), and electrospray ionization-mass spectrometry (ESI-MS). Here, **PLZ1.0** showed the most severe effects, including multiple oxidations with Δ*m* = 16 Da, truncation after a tryptophan residue close to the C-terminus, as well as covalent cross-linking of the truncated monomers (Figures 2A–C and S9). In contrast, variants without active-site tryptophans were significantly more photostable. This was further quantified by determining reaction yields after preirradiating the cerium-bound proteins for 24 h prior to substrate addition (Figure 2D).

Lanthanide binding to the active site can be monitored spectroscopically with TbCl_3_ in all variants with an active-site tryptophan (Figure S11). Here, we observed very slow binding kinetics at room temperature and essentially no signal increase at 4 °C (Figures 2E and S12). From 40 °C, however, metal binding was significantly more efficient. For all further reactions, we thus incubated the proteins with CeCl_3_ at 40 °C for 2.5 h prior to substrate addition. Furthermore, we observed unspecific cerium binding on the protein surface, which led to background activity in the KO variants. This effect could be suppressed by buffer exchange over a desalting column to remove weakly binding metal prior to photocatalysis. Still, this procedure is not feasible when screening enzyme variants in parallel using microtiter plates. We thus decided to re-engineer the protein surface. Here, we identified clusters of negatively charged residues, which were predicted to bind metal according to the software tool *BioMetAll*,^35^ and then introduced eight mutations (6× D to N and 2× E to Q) to minimize lanthanide binding to the protein surface (Figure S13). This led to a high terbium luminescence signal directly after mixing (Figure 2E) and less unspecifically bound metal as quantified by inductively coupled plasma-MS (ICP-MS) (Figure S14).

The optimized variant **PLZ1.4** combines increased photostability with improved metal binding properties and was thus characterized in more detail. Overnight photoreactions with 5 mol % enzyme gave up to 80% yield. The enzyme’s total turnover number was determined to be TTN = 78 ± 5 (Figure S15). We also measured Michaelis–Menten kinetics for diol substrate **1** and determined *k*_cat_ = 5.3 ± 0.2 h^–1^ and K_M_ = 2.9 ± 0.4 mM (Figure 2F).

### Substrate Scope

With the optimized photoenzyme **PLZ1.4** in hand, we started exploring its substrate scope (Table 1 and Figures S16–S34). Aromatic substrates and products could be detected directly using their ultraviolet (UV) absorption, while the aldehydes produced from aliphatic substrates were derivatized with 2-amino-benzamidoxime (ABAO) prior to high-performance liquid chromatography (HPLC) analysis (Figures S3 and S4).^36^ For hydrobenzoin (**1**) and 1-phenyl-1,2-ethanediol (**3**), all stereoisomers were separately available, but other substrates were synthesized as mixtures of stereoisomers. We screened several substituted aromatic diols (**4**, **6**, **8**) and found similar yields as observed for hydrobenzoin (**1**). Tertiary diol **10** was less well accepted. Single aromatic alcohols (**12**, **14**) and aliphatic diols (**15**, **17**) also gave lower yields. Interestingly, substrates **12** and **19** yielded a product mixture consisting of benzaldehyde (**2**) and benzyl alcohol (**13**), while the cleavage of the aromatic amino alcohol **21** gave two molecules of **2** (see Figure S39 for mechanistic proposals). The observed range of yields reflects both the enzyme’s chemoselectivity as well as the intrinsic reactivity differences of the various substrates.

**Table 1:**
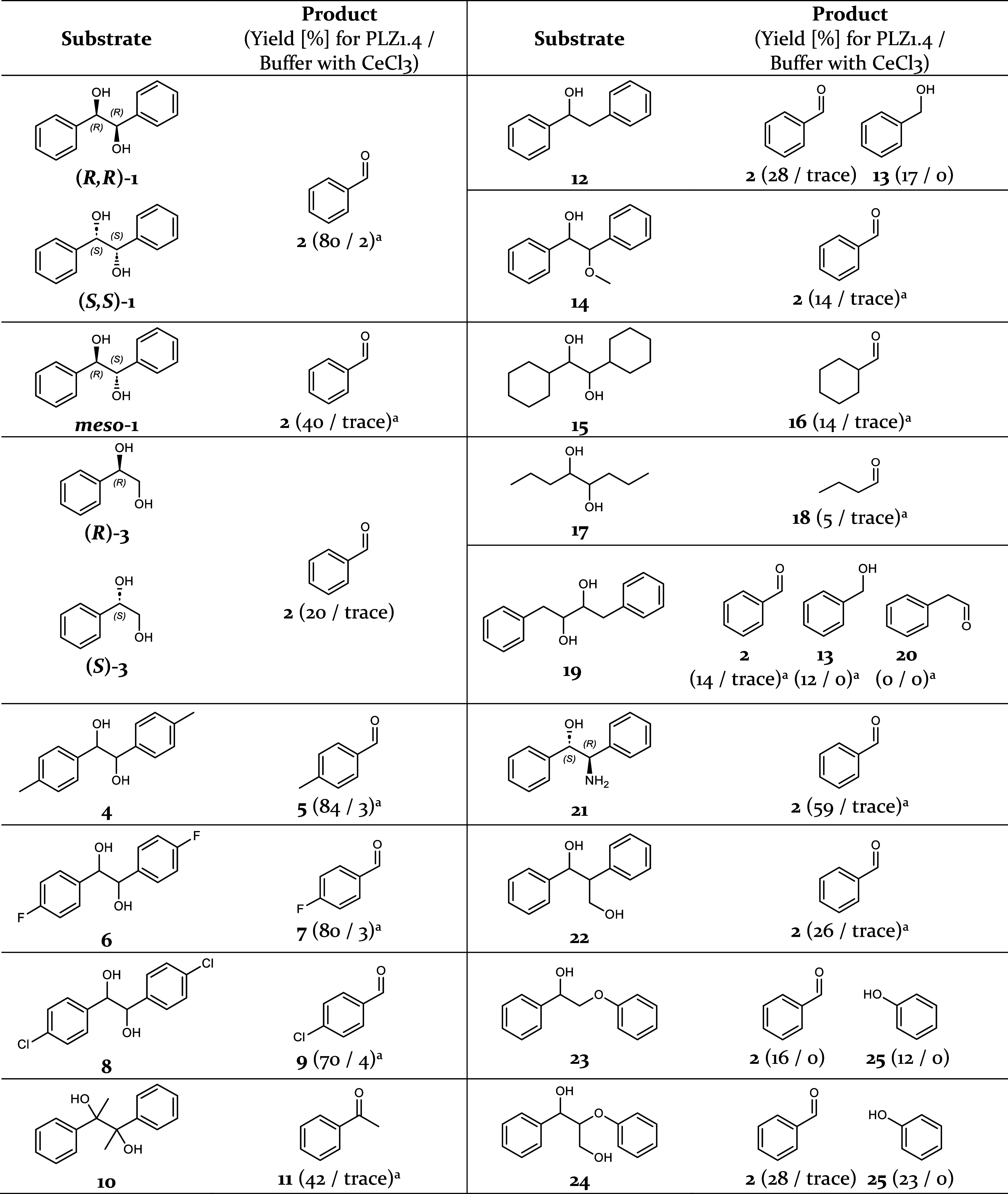
Substrate Scope^b^

a Yield for the formation of two molecules product per molecule substrate.

b Photoenzymatic reactions were performed in aqueous buffer and under aerobic conditions upon irradiation at 410-420 nm for 24 h with final concentrations of 100 μM metalloenzyme, 2 mM substrate, and 10% (v/v) acetonitrile. The yields were determined by HPLC. The accuracy (ca. ± 3%) is limited by intensity variations between single LEDs in the photoreactors.

We were most interested in identifying initial stereoselectivity enabled by the chiral protein environment and thus compared the turnover rates of **(*****R***,***R*****)-1**, **(*****S***,***S*****)-1**, and ***meso*****-1** premixed in a single reaction vial. While a pronounced diastereoselectivity was observed, no enantioselectivity could be detected (Figure S35). However, when separating the reaction mixture after the photocatalytic cleavage of **19** on a chiral column, we reproducibly saw low enantiomeric excess (ee) for the unreacted substrate (Figures S36–S38). The efficiency of such kinetic resolutions is typically evaluated by calculating the enantioselectivity value *S* from ee and conversion. However, we observed only very low enantioselectivities (for instance 5% ee at 40% conversion of **19** for **PLZ1.4**), so that a quantitative analysis of *S* was not meaningful.

Also the initial single-chain variant **PLZ1.1** showed a low selectivity, albeit with preference for the opposite enantiomer. **PLZ1.3** and **PLZ1.4**, which only differ in surface mutations, preferentially cleave **(*****R***,***R*****)-19**, while **PLZ1.1** prefers **(*****S***,***S*****)-19** (Figure S37). These results indicate that our photocatalytic reaction indeed happens inside the protein and that its stereochemical outcome is influenced by the binding pocket.

We obtained an AlphaFold2 model^37,38^ of **PLZ1.4**, which overall resembles the crystal structure of **PLZ1.0**,^24^ and performed substrate docking for **1** and **19** (Figures 2G, S52, and S54). The central cavity of the enzyme is large and the two domains are tethered by flexible linkers, which allows for several substrate conformations of all three stereoisomers to bind with similar binding energy and thus explains the low enantioselectivity (Video S1). However, based on these results, rational engineering and directed evolution can be performed synergistically in the future to improve activity and selectivity.

### Cleavage of Lignin Surrogates and Whole-Cell Photobiocatalysis

**Figure 3:**
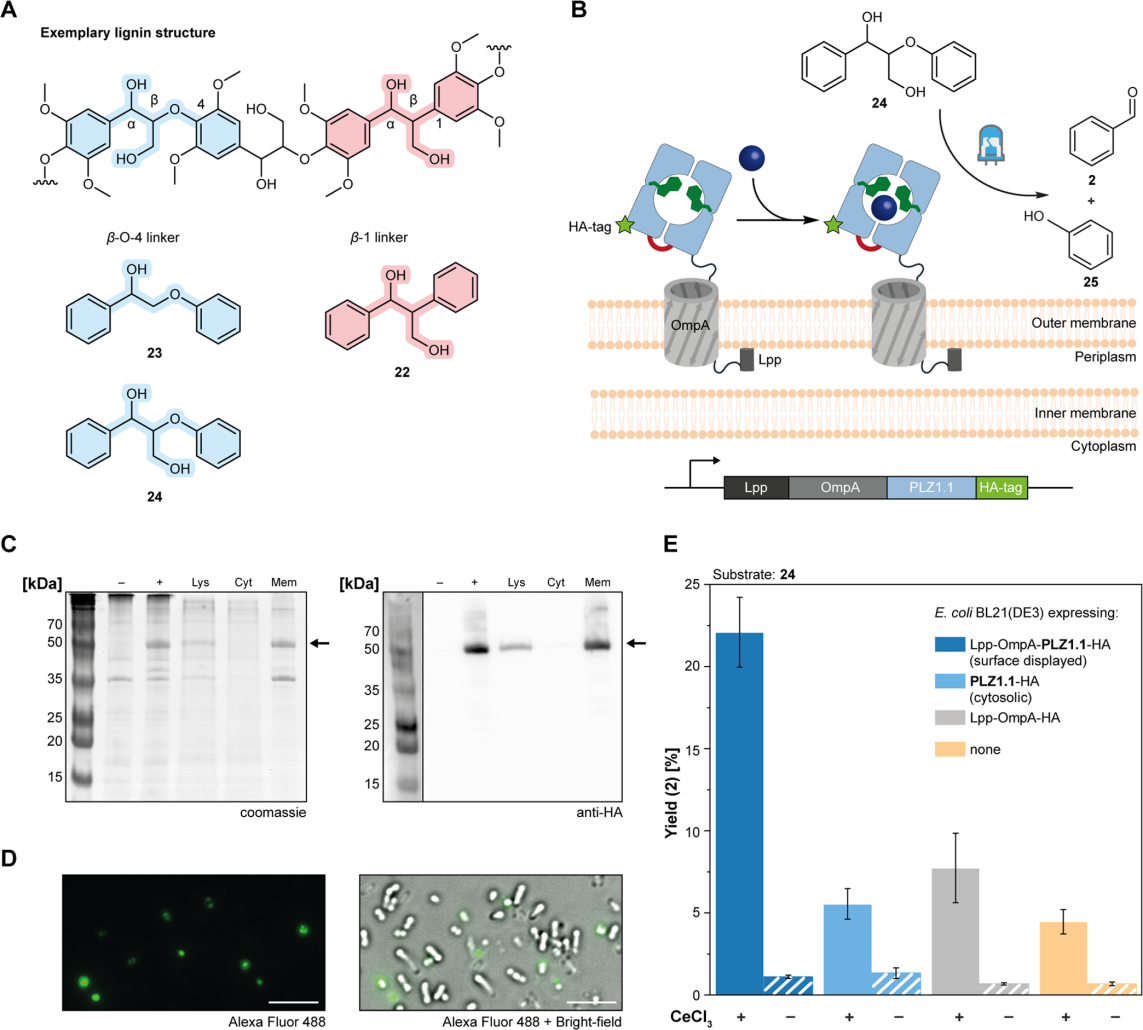
Whole-cell photobiocatalysis toward lignin degradation. (A) Exemplary structure of polymeric lignin and surrogates **22**–**24**, highlighting the most common linkages. (B) *Escherichia coli* surface display of PLZ1.1 as a Lpp-OmpA fusion construct with a C-terminal HA tag. PLZ1.1 was chosen over PLZ1.4, as the latter gave lower expression yields for the surface-displayed construct. (C) SDS-PAGE and Western blot analysis of *E. coli* cells expressing Lpp-OmpA-PLZ1.1-HA. Samples before induction (−), after expression (+), cell lysate (Lys), cytoplasmic fraction (Cyt), and membrane fraction (Mem) are shown. The arrow indicates the molecular weight of the fusion construct. (D) Fluorescence microscopy of *E. coli* cells expressing Lpp-OmpA-PLZ1.1-HA. The cells were treated with a primary anti-HA antibody and a secondary fluorescent antibody. The scale bar corresponds to 5 μm. (E) Photocatalytic degradation of lignin surrogate **24** by whole cells incubated with CeCl_3_. The yield of benzaldehyde (**2**) was determined in triplicates. Controls without CeCl_3_ are shown as striped bars.

Aromatic diol motifs are found in lignin, a complex organic polymer that together with cellulose is responsible for the rigidness of plant cell walls, especially in wood (Figure 3A). It is one of the most abundant renewable resources for aromatic compounds and its chemical or enzymatic degradation is a promising way toward valorization.^39−41^ We asked whether our photoenzyme would also accept small-molecule lignin surrogates (**22**, **23**, **24**).^42,43^ For all three substrates, benzaldehyde (**2**) was detected after enzymatic cerium photoredox catalysis using purified **PLZ1.4**. Substrates **23** and **24** yielded also phenol (**25**) as the second cleavage product (Table 1). For substrate **24**, we also measured enzyme kinetics with *k*_cat_ = 1.5 ± 0.2 h^–1^ and *K*_M_ = 5.5 ± 1.0 mM (Figure S42).

Interestingly, the HPLC trace for 1,3-diol **22** showed 1,2-diol **3** as a reaction intermediate, which is then cleaved one more time to give a second equivalent of benzaldehyde (**2**). To investigate this mechanism further, we performed trapping experiments with TEMPO (Figure S40). The results confirmed the presence of two benzylic radicals (identified as TEMPO adducts by LC-MS), which are then hydroxylated. To test whether the respective oxygen atoms originate from water or molecular oxygen, we set up photoenzymatic reactions in H_2_^18^O (Figure S41). Quantitative ^18^O-isotope labeling of benzaldehyde (**2**) was observed as expected due to the rapid exchange with water after the reaction. Next, we thus tested the cleavage of substrate **12** in H_2_^18^O, where also benzyl alcohol (**13**) is formed that cannot undergo this exchange. Here, we found exclusively benzyl alcohol with ^16^O, not ^18^O, indicating that the benzylic radicals react with molecular oxygen, but not water.

The photoenzymatic process would be most economical if whole-cell biocatalysis was established. This would require artificial metalloenzyme formation in the cellular environment. We thus explored a cell surface display approach. We generated a fusion construct in which **PLZ1.1** is attached to a Lpp-OmpA sequence (Figure 3B), described previously for surface display of an artificial metalloenzyme on *E. coli* cells.^45^ The successful display was verified by detecting a C-terminal HA tag in both Western blot analysis after cell fractionation and fluorescence microscopy (Figures 3C,D, S43, and S44). We found that lignin surrogate **24** was degraded to benzaldehyde (**2**) and phenol (**25**) using whole cells treated with CeCl_3_ in the presence of visible light. Here, the surface-displayed construct was compared to several controls, including cells expressing the enzyme cytoplasmically, an Lpp-OmpA construct without the enzyme, and uninduced *E. coli* BL21 cells, all in the presence and absence of CeCl_3_ (Figures 3E and S45–S49). These results show that the cerium-dependent photoenzyme is compatible with whole-cell biocatalysis and may be further developed toward lignin degradation.

### Cerium Photoredox Catalysis with a Natural Lanthanide-Dependent Enzyme

**Figure 4:**
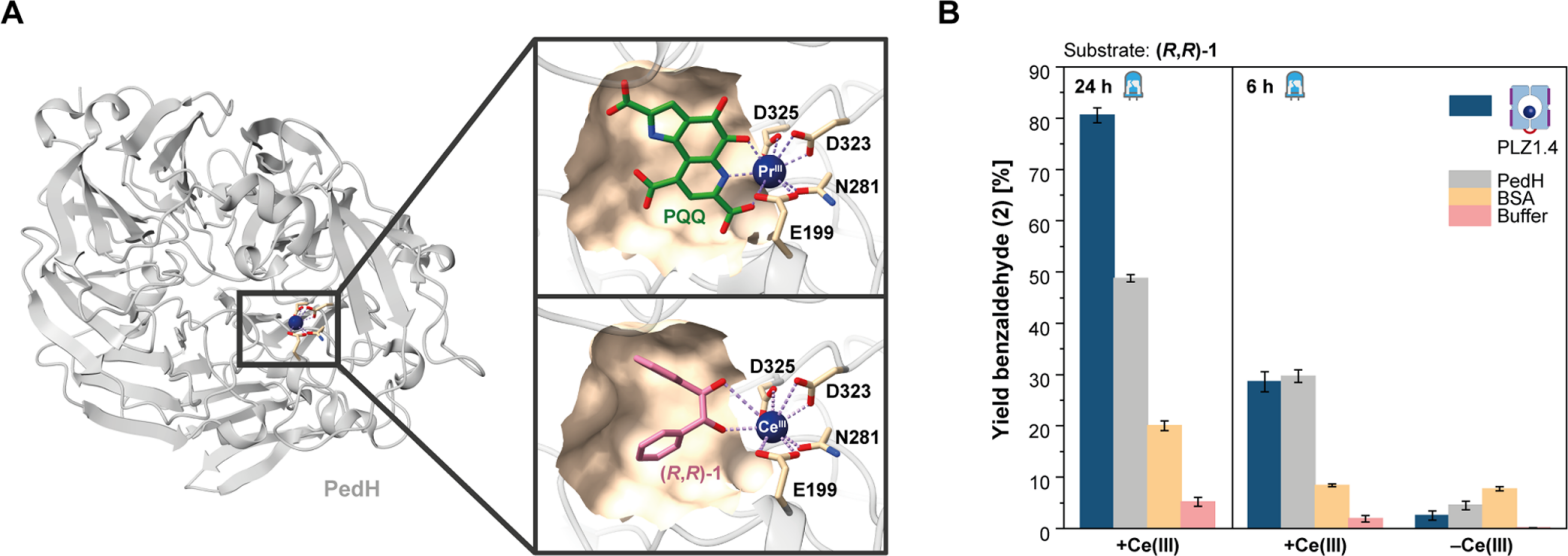
Photocatalytic diol cleavage by the natural lanthanide-dependent redox enzyme PedH from *P. putida*. (A) Crystal structure of a previously engineered variant of PedH (PDB entry: 6ZCV).^44^ The alcohol dehydrogenase has a lanthanide binding site and utilizes the redox cofactor pyrroloquinoline quinone (PQQ). The natural active site is shown in the upper panel. The lower panel shows a docking model in which **(*****R***,***R*****)-1** occupies the PQQ binding pocket. (B) Photocatalytic cleavage of **(*****R***,***R*****)-1** by PedH reconstituted with CeCl_3_ in the absence of PQQ. The yields were compared to PLZ1.4 as the positive control and BSA as the negative control. Photostability was assessed by comparing the yields after 6 and 24 h. While being fully denatured after 6 h (see Figure S10), PedH aggregates may still unspecifically bind Ce(III), leading to some residual activity.

Over the last years, several lanthanide-dependent enzymes have been found in nature and were characterized in detail, with high-resolution structures being available^31^ We wondered whether these natural proteins may also be applied for cerium photoredox catalysis and chose the lanthanide-dependent alcohol dehydrogenase PedH from *Pseudomonas putida* KT2440 as a test case. The enzyme utilizes the redox cofactor pyrroloquinoline quinone (PQQ), which is only active when interacting with a Lewis acidic lanthanide ion in the active site. We expressed a previously engineered variant of PedH^44^ recombinantly in *E. coli* and reconstituted the apoenzyme with CeCl_3_, but added no PQQ. The rationale was that the free space in the PQQ binding pocket may then be occupied by our diol substrates, which was supported by substrate docking results (Figure 4A and Video S2).

We first measured the binding affinity of PedH for Ce(III) in displacement titrations against Tb(III) using the tryptophan-enhanced terbium luminescence readout. Even in the absence of PQQ, PedH binds the lanthanide ions with nanomolar affinity (Figures S50 and S51). Next, we performed the photocatalytic diol cleavage of **(*****R***,***R*****)-1** using cerium-bound PedH in comparison to **PLZ1.4** as a positive control and bovine serum albumin (BSA) as a negative control. PedH gave similar yields as **PLZ1.4** after 6 h of irradiation, but turned out to be less photostable (Figures 4B and S10). These results show that also natural lanthanide binding proteins may be utilized for enzymatic cerium photoredox catalysis. However, the low photostability is a major limitation, while the de novo scaffold’s robustness brings a clear advantage.

## Discussion

Our results demonstrate that cerium photoredox catalysis can be performed efficiently inside of suitable protein scaffolds that possess a lanthanide binding site. The newly generated photoenzymes catalyze radical C–C bond cleavages. We optimized the catalysts, investigated mechanistic details, and explored potential applications.

Forming radical species in the presence of oxygen, we initially observed severe oxidative photodamage of the de novo scaffold, but could increase its photostability by protein engineering. Here, it was crucial to remove the tryptophan residues in close proximity to the metal binding site. We also demonstrated that the protein environment is able to induce initial stereocontrol, notably with opposite enantiopreference for two of the PLZ variants. Even though the selectivities are very low and not yet practically useful for kinetic resolutions, we generated a starting point for further enzyme engineering. We also made first steps toward another potential application of *PhotoLanZymes*, namely the degradation of lignin into synthetically valuable building blocks. This important challenge toward sustainable chemistry has been targeted previously by separate photocatalytic^42,46,47^ or enzymatic^41^ approaches, but not yet with a hybrid photobiocatalyst. However, we only tested small-molecule lignin surrogates in this study. Further enzyme engineering and process optimization will be required to degrade actual lignin.

Photoenzymatic catalysis has gained momentum in recent years.^3^ Natural redox enzymes have been shown to catalyze new-to-nature radical chemistry in the presence of light,^48−51^ but also rationally designed photoenzymes have been generated, for instance by covalently attaching photocatalytic moieties^52,53^ or by genetically encoding a triplet sensitizer.^7,8,54^ However, in most cases, the reactions have to be performed under inert reaction conditions. Our work adds a new mode of action to the photoenzymatic toolbox, namely cerium photoredox catalysis, which is compatible with whole-cell biocatalysis and aerobic conditions. The scope of this chemistry goes far beyond the diol cleavages shown here. Based on our results, cerium-dependent photoenzymes for stereoselective C–H activation and C–C bond forming reactions, which proceed via alkyl or aryl radicals, may be developed next. To that end, the choice of protein scaffold will be crucial. Our de novo TIM barrel binds lanthanides with very high affinity, but the large cavity size and high flexibility of the domain-connecting linkers turned out be a limitation when aiming for precise stereocontrol in the substrate binding site. Our ongoing efforts thus focus on evaluating alternative scaffolds, considering both natural as well as de novo proteins. For the latter, we see great potential in applying the recently developed AI-based protein design tools (AlphaFold, RoseTTAFold, RF diffusion and ProteinMPNN), which bring the field closer to the ultimate goal of building robust and tailor-made de novo proteins around just any active site of choice.^55−58^

Even with the perfect protein scaffold in hand, fine-tuning the photoenzymes’ catalytic parameters will still need experimental optimization. One of the most efficient ways to do so is directed evolution by in vivo selection. Here, we implemented an *E. coli* surface display strategy, which allows us to form artificial cerium enzymes in the context of whole cells.^59,60^ The next step toward in vivo selection is to couple the survival of the cells to a cerium and light-dependent enzymatic activity. We believe that this approach will facilitate the development of artificial cerium enzymes for a broad range of synthetically valuable photoredox transformations.

## Data Availability

All raw data files associated with this study are openly available in the repository mediaTUM at DOI: 10.14459/2024mp1743919.

## Supporting Information Available

Experimental procedures and additional data on syntheses, characterizations, quantifications, mechanistic studies, and computational work (PDF)
All obtained docking poses for **PLZ1.4** (Video S1) (MP4)
All obtained docking poses for PedH (Video S2) (MP4)

## Abbreviations

ABAO: 2-amino-benzamidoxime
BSA: bovine serum albumin
DPA: 9,10-diphenylanthracene
ESI-MS: electrospray ionization mass spectrometry
ICP-MS: inductively coupled plasma mass spectrometry
KO: knockout
LC-MS: liquid chromatography coupled to mass spectrometry
LMCT: ligand-to-metal charge transfer
PLZ: PhotoLanZyme
PQQ: pyrroloquinoline quinone
SET: single electron transfer
SDS-PAGE: sodium dodecyl sulfate polyacrylamide gel electrophoresis
SEC: size exclusion chromatography
TEMPO: 2,2,6,6-tetramethylpiperidinyloxyl
TFD: TIM-ferredoxin dimer
