# Supplementary material for: A De Novo Metalloenzyme for Cerium Photoredox Catalysis
Source: J Am Chem Soc. 2024 Aug 8;146(38):25976–85. doi: 10.1021/jacs.4c04618 (PMC11440500; doi:10.1021/jacs.4c04618)
Supplement: Supplementary file 1 — ja4c04618_si_001.pdf [file ja4c04618_si_001.pdf]

Supporting Information

A de novo metalloenzyme for cerium photoredox catalysis

Andreas Sebastian Klein[1]†, Florian Leiss-Maier[1]†, Rahel Mühlhofer[1], Benedikt Boesen[1], Ghulam Mustafa[1], Hannah Kugler[1], Cathleen Zeymer[1,2]*

[1]Center for Functional Protein Assemblies & Department of Bioscience, TUM School of Natural Sciences, Technical University of Munich (TUM), 85748 Garching, Germany

[2]TUM Catalysis Research Center, Technical University of Munich (TUM), 85748 Garching, Germany

† These authors contributed equally to this work.
*Corresponding author: Cathleen Zeymer, cathleen.zeymer@tum.de

Table of Contents

S1 General methods 4
S2 Molecular cloning, recombinant expression and protein purification 8
S3 Procedures for photoenzymatic reactions 18
S4 Photostability studies 27
S5 Lanthanide binding to PLZ variants 30
S6 Protein surface engineering 31
S7 Determination of total turnover numbers 32
S8 Substrate scope 33
S9 Mechanistic studies 56
S10 Whole-cell photobiocatalysis 61
S11 Lanthanide binding measurements with PedH 66
S12 Molecular modeling and docking of diol substrates 69
S13 Circular dichroism spectra of PLZ variants and PedH 71
S14 Chemical syntheses 72
S15 NMR spectra 82
S16 HPLC traces of all substrate and product reference compounds 96
S17 Overview of molecules 110
S18 References 111

List of Supplementary Figures

Figure S1. The 6-vial photoreactor setup 7
Figure S2. Screening for optimal pH and wavelength 19
Figure S3/S4. Calibration curves for HPLC-based quantification 22/23
Figure S5. Photoenzymatic time course 24
Figure S6. Light on/off kinetics 24
Figure S7. Regeneration of Ce(IV) 26
Figure S8. Oxidative modifications of tryptophan and methionine 28
Figure S9. Photodamage analysis of PLZ variants by mass spectrometry 28
Figure S10. Photostability of PedH 29
Figure S11. Tryptophan-enhanced Tb(III) luminescence 30
Figure S12. Tb(III) binding kinetics of PLZ1.0 30
Figure S13. Surface modifications in PLZ 31
Figure S14. ICP-MS measurements 32
Figure S15. Determination of total turnover numbers (TTN) 32
Figure S16–S34. HPLC traces for all reactions shown in the substrate scope 33
Figure S35. Chiral HPLC analysis for diol **1** 52
Figure S36/S37. Initial stereoselectivity for diol **19** 53
Figure S38. Assignment of stereoisomers of diol **19** in chiral HPLC traces 55
Figure S39. Observed oxidations during the photocleavage of selected substrates 56
Figure S40. Radical trapping with TEMPO 57
Figure S41. ^{18}O isotope labeling 59
Figure S42. Michaelis-Menten kinetics for lignin surrogate **24** 60
Figure S43. SDS-PAGE and Western blot analysis for cell surface display 62
Figure S44. Negative control for fluorescence microscopy 63
Figure S45–S49. HPLC traces for whole-cell photobiocatalysis 64
Figure S50. Tb(III) binding to PedH in the absence of PQQ 67
Figure S51. Ce(III) binding to PedH in the absence of PQQ 68
Figure S52. AlphaFold2 prediction of PLZ1.4 69
Figure S53. Validation of the docking method using PQQ and PedH 70

Figure S54. Substrate docking results for PLZ1.4 70
Figure S55/S56. CD spectra of all proteins……………………………………………......................71
Figure S57. HPLC chromatogram of the stereoselective synthesis of **(*R*,*R*)-19** 80
Figure S58. HPLC chromatogram of the stereoselective synthesis of **(*S*,*S*)-19** 80
Figure S59–S72. ^{1}H- and ^{13}C-NMR-spectra 82
Figure S73–S100. HPLC chromatograms of all compounds 96

S1 General methods

Chemical syntheses: All reactions requiring an inert atmosphere were performed with dried glassware, under an atmosphere of dry nitrogen, and applying standard *Schlenk* techniques. Reactions were monitored by thin layer chromatography (TLC) on pre-coated plastic sheets (Polygram SIL G/UV254, *Macherey–Nagel*, Düren, Germany) with detection by UV light at 245 nm, *p*-anisaldehyde staining or $KMnO_4$ staining ($KMnO_4$ staining solution: 1.5 g $KMnO_4$, 10 g K_2CO_3, 1.25 mL 10% (*v*/*v*) NaOH, 200 mL H_2O; *p*-anisaldehyde staining solution: 3 mL *p*-anisaldehyde, 6 mL conc. H_2SO_4 in 300 mL AcOH) followed by brief heating with a heat gun. Removal of solvent was performed at 40 °C *in vacuo*. Preparative column chromatography was performed over silica gel 60 (particle size 0.040–0.063 mm, 230–240 mesh, *Macherey–Nagel*, Düren, Germany or Biotage Sfär Silica Duo 60 µm, *Biotage*, Uppsala, Sweden). (*S*,*S*)-Hydrobenzoin [***(S,S)*-1**] was purified by sublimation; all other chemicals and solvents were used as purchased without further purification.

HPLC: HPLC analysis was performed on a Vanquish System (*Thermo Fisher Scientific,* Waltham, MA, USA) equipped with an achiral Hypersil Gold C18 column (100 x 2.1 mm, 3 µm particle size, *Thermo Fisher Scientific,* Waltham, MA, USA) at 45 °C, a chiral Chiralcel OJ-RH column (150 x 4.6 mm, 5 µm particle size, Daicel Corporation, Osaka, Japan) at room temperature, and a VF-D11-A diode array detector. Detection wavelengths were 205 nm and 254 nm, 3D field (190 nm–450 nm).

Method 1 (achiral Hypersil Gold C18 column): Water + 0.1% (*v*/*v*) trifluoroacetic acid (TFA) as solvent "A" and acetonitrile + 0.1% (*v*/*v*) TFA as solvent "B" were used as eluents for the following gradient program:

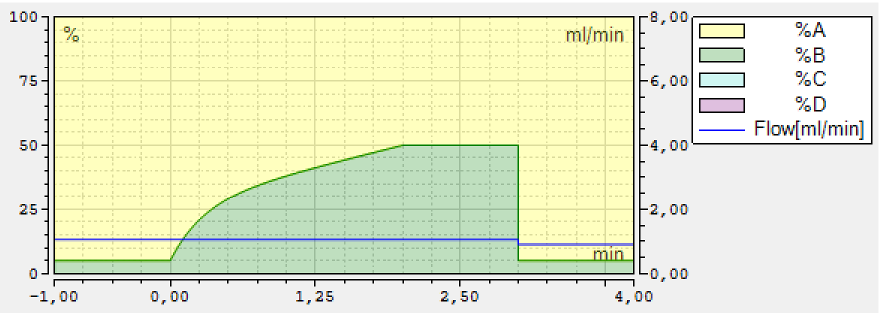

No	Time	Flow [ml/min]	%B	%C	%D	Curve
1	-1,000	Equilibration				
2	-1,000	1,050	5,0	0,0	0,0	5
3	*New Row*					
4	0,000	Run				
5	0,000	1,050	5,0	0,0	0,0	5
6	2,000	1,050	50,0	0,0	0,0	3
7	3,000	1,050	50,0	0,0	0,0	5
8	3,000	0,900	5,0	0,0	0,0	5
9	4,000	0,900	5,0	0,0	0,0	5
10	*New Row*					
11	4,000	Stop Run				

Insert

Delete

Method 2 (chiral Chiralcel OJ-RH column): Water as solvent "C" and acetonitrile as solvent "D" were used as eluents for the following gradient program:

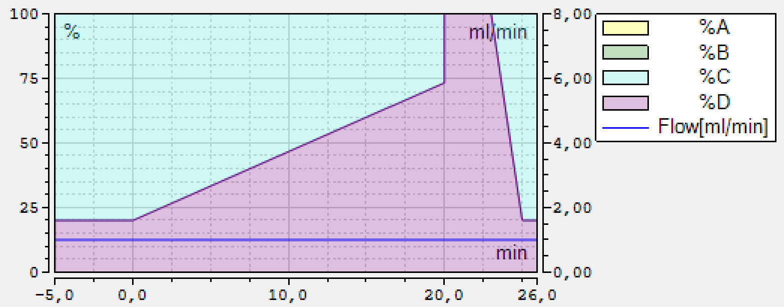

No	Time	Flow [ml/min]	%B	%C	%D	Curve
1	-5,000	Equilibration				
2	-5,000	1,000	0,0	80,0	20,0	5
3	*New Row*					
4	0,000	Run				
5	0,000	1,000	0,0	80,0	20,0	5
6	20,000	1,000	0,0	26,7	73,3	5
7	20,000	1,000	0,0	0,0	100,0	5
8	23,000	1,000	0,0	0,0	100,0	5
9	25,000	1,000	0,0	80,0	20,0	5
10	26,000	1,000	0,0	80,0	20,0	5
11	*New Row*					
12	26,000	Stop Run				

Method 3 (chiral Chiralcel OJ-RH column): Water as solvent "C" and acetonitrile as solvent "D" were used as eluents for the following program:

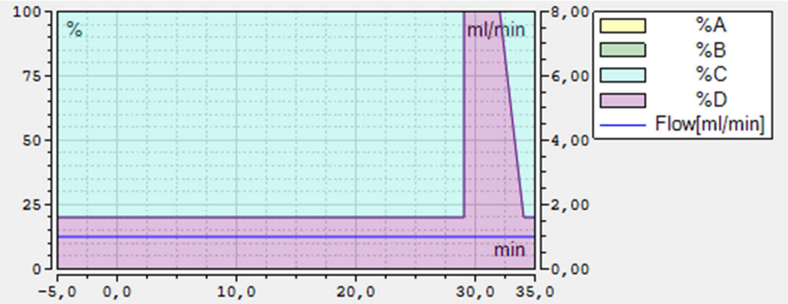

No	Time	Flow [ml/min]	%B	%C	%D	Curve
1	-5,000	Equilibration				
2	-5,000	1,000	0,0	80,0	20,0	5
3	*New Row*					
4	0,000	Run				
5	0,000	1,000	0,0	80,0	20,0	5
6	29,000	1,000	0,0	80,0	20,0	5
7	29,000	1,000	0,0	0,0	100,0	5
8	32,000	1,000	0,0	0,0	100,0	5
9	34,000	1,000	0,0	80,0	20,0	5
10	35,000	1,000	0,0	80,0	20,0	5
11	*New Row*					
12	35,000	Stop Run				

LC-MS: The measurements were performed using an *Agilent* 1290 Infinity II series UHPLC (*Agilent Technologies*, Santa Clara, CA, USA) equipped with a diode array detector (G7117C) and a single-quadrupole mass detector (G6135B) with an AJS (Agilent Jet Stream) electrospray ion source. Analytes were separated on a Poroshell 120 EC-C18 column (100 x 2.1 mm, 1.9 µm particle size, *Agilent Technologies*, Santa Clara, CA, USA) at 30 °C. Water + 0.1% (*v*/*v*) formic acid and acetonitrile + 0.1% (*v*/*v*) formic acid were used as eluents for the following gradient program (flow rate 0.4 mL/min):

0.00 min: water + 0.1% (*v*/*v*) formic acid/acetonitrile + 0.1% (*v*/*v*) formic acid (95:5),
1.00 min: water + 0.1% (*v*/*v*) formic acid/acetonitrile + 0.1% (*v*/*v*) formic acid (95:5),
16.00 min: water + 0.1% (*v*/*v*) formic acid/acetonitrile + 0.1% (*v*/*v*) formic acid (5:95),
16.10 min: 100% acetonitrile + 0.1% (*v*/*v*) formic acid,
20.0 min: 100% acetonitrile + 0.1% (*v*/*v*) formic acid,
20.01 min: water + 0.1% (*v*/*v*) formic acid/acetonitrile + 0.1% (*v*/*v*) formic acid (95:5).

1 µL of each sample was injected. Mass spectrometry detection was set to positive mode with a range of *m*/*z* = 50–650. Substances were identified by their UV absorption spectra and their mass to charge ratio (*m*/*z*). Detection wavelengths were 254 nm and 3D field (220 nm–450 nm).

NMR spectroscopy: ^{1}H- and ^{13}C-NMR spectra were recorded at room temperature on a *Bruker* Avance III HD 300 or a *Bruker* Avance III HD 400 nuclear magnetic resonance spectrometer (*Bruker,* Billerica, USA) at ambient temperature in $CDCl_3$ at 300/75 MHz or 400/101 MHz, respectively. The chemical shifts are given in ppm relative to tetramethylsilane [^{1}H: δ($SiMe_4$) = 0.00 ppm] as an internal standard or relative to the solvent [^{1}H: δ($CDCl_3$) = 7.26 ppm; ^{13}C: δ($CDCl_3$) = 77.16 ppm, ^{1}H: δ(CD_3OD) = 3.31 ppm; ^{13}C: δ(CD_3OD) = 49.00 ppm].; ^{1}H: δ(DMSO-d6) = 2.50 ppm; ^{13}C: δ(DMSO-d6) = 39.52 ppm] Signals were assigned by means of ^{1}H, ^{13}C, ^{1}H-^{1}H-COSY-, ^{1}H-^{13}C-HSQC- and ^{1}H-^{13}C-HMBC-experiments; splitting patterns are given as singlet (s), doublet (d), triplet (t), quartett (q), doublet of doublet (dd), doublet of doublet of doublet (ddd), multiplet (m) and broad singlet (brs). Coupling constants (*J*) are reported in Hz.

GC-MS: The measurements were performed using an Agilent 7890B GC system (*Agilent Technologies*, Santa Clara, CA, USA) equipped with an Agilent VF-200ms column (30 m x 0.25 mm x 0.25 µm) and an Agilent MSD 5977A Mass detector. The temperatures of the MS source and the MS spectrometer were fixed at 230 °C and 150 °C, respectively. Samples (1 µL injection volume) were analyzed using following gradient for the column oven: 60 °C (3 min) raised to 300 °C (15 °C/min) and maintained at that temperature for 5 min.

UV/Vis spectroscopy: UV/Vis spectra were recorded on a *Jasco* V-750 photometer *(JASCO Deutschland* GmbH, Pfungstadt, Germany) at room temperature (data interval: 1 nm; bandwidth: 0.2 nm; response: 0.24 s, path length: 1 cm). Background absorption was corrected by recording a blank spectrum in advance.

Circular dichroism (CD): CD spectra of all proteins were recorded at 200–280 nm on a Chirascan-plus CD spectrometer (*Applied Photophysics*, Leatherhead, England) using a quartz cuvette with 1 mm path length. A bandwidth of 1.0 nm and 1.0 nm steps at 0.5 s per point were

used. 10 individual measurements at 20 °C were averaged for each spectrum. The CD signals were recorded in millidegrees (m°) and subsequently converted into mean residue molar ellipticity values.

The 6-vial photoreactor setup: All LEDs were purchased at *Avonec* (*Avonec*, Wesel, Germany) as high power LEDs soldered on starboards (455–460 nm 5 W, 435–440 nm 5 W, 440–450 nm 3 W, 430–435 nm 3 W, 410–420 nm 3 W, 390–400 nm 3 W, 380–390 nm 3 W). The LEDs were glued using Keratherm Bond 100 RT thermal adhesive (*KERAFOL*, Eschenbach in der Oberpfalz, Germany) onto *Fischer Elektronik* SK 42 heat sinks (100x160x25 mm, aluminum, 0.95K/W; *Fischer Elektronik*, Lüdenscheid, Germany; **Figure S1A**). Cooling of the LED heat sink and the six reaction vials (each positioned precisely above one LED) was carried out using a hollow aluminum block (**Figure S1B**) attached to a Minichiller 280 OLÉ cooling unit (*Peter Huber Kältemaschinenbau* SE, Offenburg, Germany). The cooling water temperature was set to 18 °C giving a reproducible reaction temperature of approx. 23 °C. The MW LCM-40 LED drivers (*MEAN WELL Enterprises* Co, New Taipei City, Taiwan) were operated at a constant current of 0.7 A. Importantly, this setup ensured reproducible conditions for all photoenzymatic reactions.

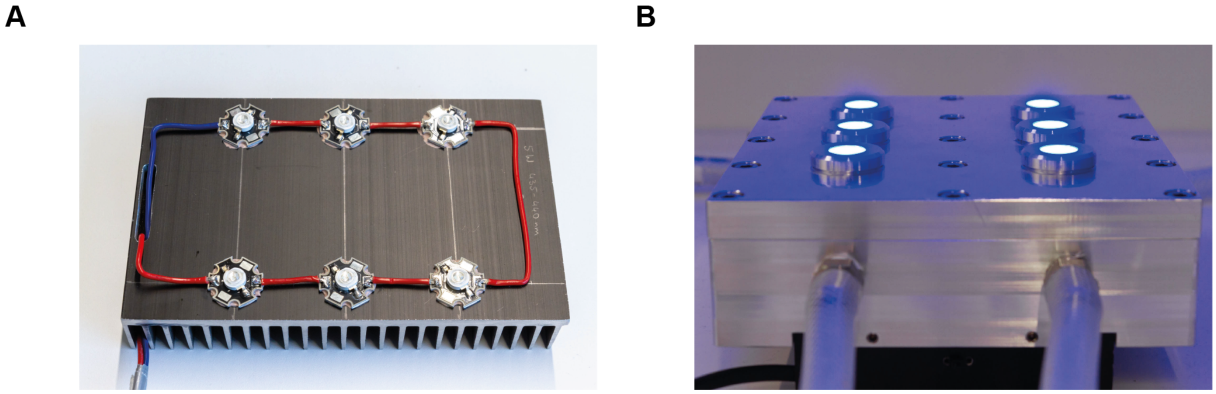

Figure S1. The 6-vial photoreactor setup. **(A)** High power LEDs glued onto the heat sink. **(B)** The water-cooled aluminum block positions the reaction vials above the LEDs.

S2 Molecular cloning, recombinant expression and protein purification

S2.1 Bacterial strains and culture conditions

Cultivation of *Escherichia coli* (*E. coli*) BL21-Gold(DE3), SoluBL21, and DH10B strains was carried out in LB (lysogeny broth) liquid medium (*Carl Roth*, Karlsruhe, Germany: 10 g/L tryptone, 5 g/L yeast extract, 10 g/L sodium chloride) or on LB agar plates [addition of 1.5% (*w*/*v*) agar] at 37 °C. Ampicillin (final concentration of 100 µg/mL) or kanamycin (final concentration of 30 µg/mL) were added as antibiotics to the culture medium. All cultivation media were prepared using distilled water and sterilized by autoclaving.

S2.2 Molecular cloning

Synthetic genes were ordered from *Twist Bioscience* (South San Francisco, CA, USA). All PCR reactions were performed as described in **Table S1** and **Table S2** using primers described in **Table S3**. All vectors were assembled using an isothermal *Gibson Assembly* (1 h, 50 °C) for one or multiple PCR fragments.[1] Single point mutations were introduced according to a method described by *Heydenreich et al*. (2017) using the ColE1 origin of replication primers #1 and #2 and the corresponding primers for the point mutation (**Table S3**).[2] Amplification of the fragments and linearized vectors were confirmed by agarose gel electrophoresis. *Dpn*I digestion (37 °C, 1 h or overnight) was performed to remove methylated template DNA from PCR-amplified products. The purification of those products was achieved either by using the PCR Purification Kit (*Jena Bioscience* GmbH, Jena, Germany) or after agarose gel electrophoresis from respective bands using the Monarch DNA Gel Extraction Kit (*New England Biolabs* GmbH, Frankfurt am Main, Germany).

After the assembly reaction of each construct, chemically competent *E. coli* DH10B cells were transformed by heat shock. Following addition of the assembly mixture, cells were incubated on ice for 30 min, followed by 60 s heat shock at 42 °C, addition of 750 µL LB medium or SOC medium (20 g/L tryptone, 5 g/L yeast extract, 584 mg/L sodium chloride, 186 mg/L potassium chloride, 2 g/L magnesium chloride hexahydrate, 1204 mg/L magnesium sulfate, 3.6 g/L glucose) and incubation for 1 h at 37 °C while shaking at 700 rpm. Afterwards, transformed cells were cultivated overnight on ampicillin or kanamycin containing LB agar plates at 37 °C. The successful assembly of the desired vector was tested by colony PCR and sequences were verified by *Sanger* sequencing (*GENEWIZ Germany* GmbH, Leipzig, Germany).

Table S1. Composition of PCR reactions.

**Component**	**Amount**
dNTPs (10 mM)	1 µL
Template DNA (3–5 ng/µL)	1 µL
Primer fw (10 mM)	2.5 µL
Primer rv (10 mM)	2.5 µL
Q5 buffer (5x)	10 µL
Q5 High-Fidelity polymerase	1 µL
DMSO	1.5 µL
ad *aqua dest.*	to 50 µL

Table S2. General PCR program used for the amplification of fragments and linearized vectors.

Step	Temp. [°C]	Duration	Cycle
Initial denaturation	98	1 min	
Denaturation<br>Annealing<br>Elongation	98<br>Primer dep.<br>72	10 s<br>30 s<br>20–30 s/kbp	35x
Final elongation	72	10 min	

Table S3. Primers used for cloning.

Primer #	Sequence (5‘-3‘)	Comment / Construct
1	GGAGCGAACGACCTACACCGAACTGAGATACCTACAGC G	ColE1 origin binding
2	CGCTGTAGGTATCTCAGTTCGGTGTAGGTCGTTCGCTCC	ColE1 origin binding
3	ATTCTTATTGTATTTGGTGGCGGGATGACC	PLZ1.2 (N52F)
4	TCCCGCCACCAAATACAATAAGAATATCACCACC	PLZ1.2 (N52F)
5	ATCCTTATCGTTGCGGGCGGCGGCATGACA	PLZ1.2 (N221A)
6	TGCCGCCGCCCGCAACGATAAGGATGTCGCC	PLZ1.2 (N221A)
7	ATTCTGATTGTCGCGGCTAAGGACGTGGATG	PLZ1.2 (W175A)
8	CGTCCTTAGCCGCGACAATCAGAATGTCGCC	PLZ1.2 (W175A)
9	CTGATAGTGGCCGCCAAAGACGTTGATGAG	PLZ1.3 (W6A)
10	CAACGTCTTTGGCGGCCACTATCAGAATGTC	PLZ1.3 (W6A)
11	CTCGAGGAATTCTGCAGATATC	PLZ1.4 pET17 backbone
12	ATGTATATCTCCTTCTTAAAGTTAAAC	PLZ1.4 pET17 backbone
13	TTAACTTTAAGAAGGAGATATACATATGGGTGACATTCT GATAGTG	PLZ1.4_W6
14	CAATCAGAATGTCGCCACCTTTCTTCAACGC	PLZ1.4_W6
15	AAGAAAGGTGGCGACATTCTGATTGTCGCG	PLZ1.4_W6
16	ATGGATATCTGCAGAATTCCTCGAGTTATTTTCGAACT GCGG	PLZ1.4_W6
17	CTGATAGTGGCCGCCAAAAACGTTGATGAG	PLZ1.4 (W6A)
18	CAACGTTTTTGGCGGCCACTATCAGAATGTC	PLZ1.4 (W6A)
19	CTTTAAGAAGGAGATATACATATGGCAGTGTCTAACGA GG	PedH_F412V_W561A
20	CGGATCTCAGTGGTGGTGGTGGTGGTGCTCGAGCGGTTT AACCGAGGCGGTC	PedH_F412V_W561A
21	TATTTTCAGGGCGCTAGTGCAGGTGACATTCTGATAGTG TGGGC	PLZ1.1 backbone pET29b for surface display construct
22	ATGTATATCTCCTTCTTAAAGTTAAACAAAATTATTTCT AGAGGGG	PLZ1.1 backbone pET29b for surface display construct
23	TTAACTTTAAGAAGGAGATATACATATGAAAGCTACTA AACTGGTACTGGGC	Lpp Signal peptide and 1st 9 a.a. from genomic BL21-Gold(DE3) DNA
24	GTTAATGCCCTGATCGATTTTAGCGTTGCTGGAG	Lpp Signal peptide and 1st 9 a.a. from genomic BL21-Gold(DE3) DNA
25	AAAATCGATCAGGGCATTAACCCGTATGTTGGCTTT	OmpA a.a. 46-159 from genomic BL21-Gold(DE3) DNA
26	GCCCTGAAAATAAAGATTCTCGCCCGGAATGCCGTTGTC CGGACG	OmpA a.a. 46-159 from genomic BL21-Gold(DE3) DNA
27	CCATATGATGTTCCAGATTATGCTTAAGATCCGGCTGCT AACAAAGC	HA-tag on PZL1.1 in pET29b
28	TAATCTGGAACATCATATGGATAGCCCTGAAAATAAAG ATTCTCgc	HA-tag on PZL1.1 in pET29b

S2.3 DNA and protein sequences of all constructs

Molecular weights (MW) and molar extinction coefficients (ε_{280}) were calculated using the ProtParam tool on the Expasy Server.[3]

PLZ1.0 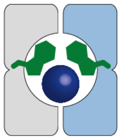 **E31+E154** **N-Strep** **PLZ1.0** (pET29b vector)

MW = 20.386 kDa **ε_{280} [$M^{-1}cm^{-1}$] = 22000**

ATGGCTAGTGCATGGTCTCATCCTCAATTCGAAAAATCCGGCTCCATGGGTGATATTTTGATTGTGTGG
GCTAAGGACGTGGATGAAATGCTGAAACAAGTTGAGATTTTACGCCGTCTTGGAGCGAAACAAATTGC
AGTAGAATCCTCGGATTGGCGTATCTTACAGGAAGCACTGAAAAAGGGTGGTGATATCCTCATCGTTA
ACGGCGGCGGAATGACAATTACTTTTCGCGGGGATGACCTTGAAGCGTTACTGAAAGCGGCTATCGAG
ATGATCAAACAAGCGCTGAAGTTCGGGGCCACCATTACCCTTAGCCTTGATGGAAACGACTTAAACAT
CAATATCACAGGTGTCCCGGAACAGGTACGCAAGGAATTGGCCAAAGAAGCAGAGCGTTTAGCTAAA
GAATTTGGAATTACGGTCACGCGCACTGGGGGTGGTGATGTGGACGAAATGTTAAAGCAGGTAGAAA
TTCTTCGTCGCTTAGGGGCAAAACAAATCGCTGTCGAAAGTGATGACTGGCGCATTCTCCAGGAAGCC
CTCAAAAAGGGCTAA

MASAWSHPQFEKSGSMGDILIVWAKDVDEMLKQVEILRRLGAKQIAVESSDWRILQEALKKGGDILIVNG
GGMTITFRGDDLEALLKAAIEMIKQALKFGATITLSLDGNDLNINITGVPEQVRKELAKEAERLAKEFGITVT
RTGGGDVDEMLKQVEILRRLGAKQIAVESDDWRILQEALKKG

PLZ1.0_KO 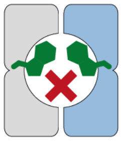 **Q31+Q154** **N-Strep** **PLZ1.0** (pET29b vector)

MW = 20.384 kDa **ε_{280} [$M^{-1}cm^{-1}$] = 22000**

ATGGCTAGTGCATGGTCTCATCCTCAATTCGAAAAATCCGGCTCCATGGGTGATATTTTGATTGTGTGG
GCTAAGGACGTGGATGAAATGCTGAAACAAGTTGAGATTTTACGCCGTCTTGGAGCGAAACAAATTGC
AGTACAATCCTCGGATTGGCGTATCTTACAGGAAGCACTGAAAAAGGGTGGTGATATCCTCATCGTTA
ACGGCGGCGGAATGACAATTACTTTTCGCGGGGATGACCTTGAAGCGTTACTGAAAGCGGCTATCGAG
ATGATCAAACAAGCGCTGAAGTTCGGGGCCACCATTACCCTTAGCCTTGATGGAAACGACTTAAACAT
CAATATCACAGGTGTCCCGGAACAGGTACGCAAGGAATTGGCCAAAGAAGCAGAGCGTTTAGCTAAA
GAATTTGGAATTACGGTCACGCGCACTGGGGGTGGTGATGTGGACGAAATGTTAAAGCAGGTAGAAA
TTCTTCGTCGCTTAGGGGCAAAACAAATCGCTGTCCAAAGTGATGACTGGCGCATTCTCCAGGAAGCC
CTCAAAAAGGGCTAA

MASAWSHPQFEKSGSMGDILIVWAKDVDEMLKQVEILRRLGAKQIAVQSSDWRILQEALKKGGDILIVNG
GGMTITFRGDDLEALLKAAIEMIKQALKFGATITLSLDGNDLNINITGVPEQVRKELAKEAERLAKEFGITVT
RTGGGDVDEMLKQVEILRRLGAKQIAVQSDDWRILQEALKKG

PLZ1.1 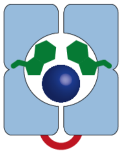 **E31+E154+E200+E323 PLZ1.1 C-Strep** (pET17b vector)

MW = 38.614 kDa **ε_{280} [$M^{-1}cm^{-1}$] = 38500**

ATGGGTGACATTCTGATAGTGTGGGCCAAAGACGTTGATGAGATGCTCAAGCAGGTCGAAATTTTGCG
TCGCTTGGGCGCGAAACAGATTGCCGTTGAGAGTAGTGACTGGCGCATACTGCAGGAAGCGCTTAAGA
AGGGTGGTGATATTCTTATTGTAAATGGTGGCGGGATGACCATTACCTTCCGCGGGGATGACCTGGAA
GCCTTGCTCAAAGCAGCTATAGAAATGATAAAGCAGGCACTTAAGTTCGGGGCCACTATAACATTGAG
CCTTGACGGAAACGACCTGAATATTAACATTACCGGCGTCCCAGAGCAGGTCCGCAAGGAGTTGGCTA
AAGAGGCCGAGCGGCTGGCTAAGGAATTCGGGATCACAGTGACTCGAACCGGTGGCGGAGATGTGGA
CGAGATGCTCAAACAGGTGGAGATACTTAGGAGACTCGGGGCCAAGCAGATCGCCGTGGAGTCTGAT
GACTGGCGAATACTGCAGGAAGCGTTGAAGAAAGGTGGCGACATTCTGATTGTCTGGGCTAAGGACG
TGGATGAAATGCTCAAGCAGGTCGAAATCCTTAGGCGTCTCGGCGCGAAACAAATTGCTGTCGAATCC
TCTGATTGGAGAATTCTCCAGGAAGCCCTCAAGAAGGGCGGCGACATCCTTATCGTTAACGGCGGCGG
CATGACAATTACTTTTCGGGGCGACGACCTTGAAGCCCTGCTGAAGGCCGCCATTGAGATGATTAAAC
AGGCCCTTAAATTCGGCGCAACCATCACACTCTCCTTGGACGGCAACGATCTGAACATCAACATCACA
GGAGTGCCAGAACAGGTTCGTAAAGAACTGGCGAAAGAGGCCGAAAGGCTGGCCAAGGAATTTGGTA
TCACAGTTACAAGAACTGGAGGAGGCGATGTTGATGAGATGCTCAAGCAAGTCGAAATCCTCAGGCG
TCTCGGTGCCAAACAGATTGCTGTTGAGTCCGACGATTGGAGAATCCTTCAGGAAGCCCTTAAGAAGG
GCGGATCCGCTTGGAGCCACCCGCAGTTCGAAAAATAA

MGDILIVWAKDVDEMLKQVEILRRLGAKQIAVESSDWRILQEALKKGGDILIVNGGGMTITFRGDDLEALL
KAAIEMIKQALKFGATITLSLDGNDLNINITGVPEQVRKELAKEAERLAKEFGITVTRTGGGDVDEMLKQV
EILRRLGAKQIAVESDDWRILQEALKKGGDILIVWAKDVDEMLKQVEILRRLGAKQIAVESSDWRILQEAL
KKGGDILIVNGGGMTITFRGDDLEALLKAAIEMIKQALKFGATITLSLDGNDLNINITGVPEQVRKELAKEA
ERLAKEFGITVTRTGGGDVDEMLKQVEILRRLGAKQIAVESDDWRILQEALKKGGSAWSHPQFEK

PLZ1.2 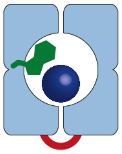 **E31+E154+E200+E323 PLZ1.2 C-Strep** (pET17b vector)

N52F+N221A+W175A

MW = 38.489 kDa **ε_{280} [$M^{-1}cm^{-1}$] = 33000**

ATGGGTGACATTCTGATAGTGTGGGCCAAAGACGTTGATGAGATGCTCAAGCAGGTCGAAATTTTGCG
TCGCTTGGGCGCGAAACAGATTGCCGTTGAGAGTAGTGACTGGCGCATACTGCAGGAAGCGCTTAAGA
AGGGTGGTGATATTCTTATTGTATTTGGTGGCGGGATGACCATTACCTTCCGCGGGGATGACCTGGAA
GCCTTGCTCAAAGCAGCTATAGAAATGATAAAGCAGGCACTTAAGTTCGGGGCCACTATAACATTGAG
CCTTGACGGAAACGACCTGAATATTAACATTACCGGCGTCCCAGAGCAGGTCCGCAAGGAGTTGGCTA
AAGAGGCCGAGCGGCTGGCTAAGGAATTCGGGATCACAGTGACTCGAACCGGTGGCGGAGATGTGGA
CGAGATGCTCAAACAGGTGGAGATACTTAGGAGACTCGGGGCCAAGCAGATCGCCGTGGAGTCTGAT
GACTGGCGAATACTGCAGGAAGCGTTGAAGAAAGGTGGCGACATTCTGATTGTCGCGGCTAAGGACG
TGGATGAAATGCTCAAGCAGGTCGAAATCCTTAGGCGTCTCGGCGCGAAACAAATTGCTGTCGAATCC
TCTGATTGGAGAATTCTCCAGGAAGCCCTCAAGAAGGGCGGCGACATCCTTATCGTTGCGGGCGGCGG
CATGACAATTACTTTTCGGGGCGACGACCTTGAAGCCCTGCTGAAGGCCGCCATTGAGATGATTAAAC
AGGCCCTTAAATTCGGCGCAACCATCACACTCTCCTTGGACGGCAACGATCTGAACATCAACATCACA
GGAGTGCCAGAACAGGTTCGTAAAGAACTGGCGAAAGAGGCCGAAAGGCTGGCCAAGGAATTTGGTA
TCACAGTTACAAGAACTGGAGGAGGCGATGTTGATGAGATGCTCAAGCAAGTCGAAATCCTCAGGCG
TCTCGGTGCCAAACAGATTGCTGTTGAGTCCGACGATTGGAGAATCCTTCAGGAAGCCCTTAAGAAGG
GCGGATCCGCTTGGAGCCACCCGCAGTTCGAAAAATAA

MGDILIVWAKDVDEMLKQVEILRRLGAKQIAVESSDWRILQEALKKGGDILIVFGGGMTITFRGDDLEALL
KAAIEMIKQALKFGATITLSLDGNDLNINITGVPEQVRKELAKEAERLAKEFGITVTRTGGGDVDEMLKQV
EILRRLGAKQIAVESDDWRILQEALKKGGDILIVAAKDVDEMLKQVEILRRLGAKQIAVESSDWRILQEALK
KGGDILIVAGGGMTITFRGDDLEALLKAAIEMIKQALKFGATITLSLDGNDLNINITGVPEQVRKELAKEAE
RLAKEFGITVTRTGGGDVDEMLKQVEILRRLGAKQIAVESDDWRILQEALKKGGSAWSHPQFEK

PLZ1.3 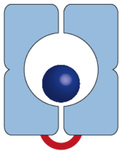 **E31+E154+E200+E323 PLZ1.3 C-Strep** (pET17b vector)

N52F+N221A+W175A+W6A

M_W = 38.374 kDa **ε_{280} [$M^{-1}cm^{-1}$] = 27500**

ATGGGTGACATTCTGATAGTGGCCGCCAAAGACGTTGATGAGATGCTCAAGCAGGTCGAAATTTTGCG
TCGCTTGGGCGCGAAACAGATTGCCGTTGAGAGTAGTGACTGGCGCATACTGCAGGAAGCGCTTAAGA
AGGGTGGTGATATTCTTATTGTATTTGGTGGCGGGATGACCATTACCTTCCGCGGGGATGACCTGGAA
GCCTTGCTCAAAGCAGCTATAGAAATGATAAAGCAGGCACTTAAGTTCGGGGCCACTATAACATTGAG
CCTTGACGGAAACGACCTGAATATTAACATTACCGGCGTCCCAGAGCAGGTCCGCAAGGAGTTGGCTA
AAGAGGCCGAGCGGCTGGCTAAGGAATTCGGGATCACAGTGACTCGAACCGGTGGCGGAGATGTGGA
CGAGATGCTCAAACAGGTGGAGATACTTAGGAGACTCGGGGCCAAGCAGATCGCCGTGGAGTCTGAT
GACTGGCGAATACTGCAGGAAGCGTTGAAGAAAGGTGGCGACATTCTGATTGTCGCGGCTAAGGACG
TGGATGAAATGCTCAAGCAGGTCGAAATCCTTAGGCGTCTCGGCGCGAAACAAATTGCTGTCGAATCC
TCTGATTGGAGAATTCTCCAGGAAGCCCTCAAGAAGGGCGGCGACATCCTTATCGTTGCGGGCGGCGG
CATGACAATTACTTTCGGGGCGACGACCTTGAAGCCCTGCTGAAGGCCGCCATTGAGATGATTAAAC
AGGCCCTTAAATTCGGCGCAACCATCACACTCTCCTTGGACGGCAACGATCTGAACATCAACATCACA
GGAGTGCCAGAACAGGTTCGTAAAGAACTGGCGAAAGAGGCCGAAAGGCTGGCCAAGGAATTTGGTA
TCACAGTTACAAGAACTGGAGGAGGCGATGTTGATGAGATGCTCAAGCAAGTCGAAATCCTCAGGCG
TCTCGGTGCCAAACAGATTGCTGTTGAGTCCGACGATTGGAGAATCCTTCAGGAAGCCCTTAAGAAGG
GCGGATCCGCTTGGAGCCACCCGCAGTTCGAAAAATAA

MGDILIVAAKDVDEMLKQVEILRRLGAKQIAVESSDWRILQEALKKGGDILIVFGGGMTITFRGDDLEALL
KAAIEMIKQALKFGATITLSLDGNDLNINITGVPEQVRKELAKEAERLAKEFGITVTRTGGGDVDEMLKQV
EILRRLGAKQIAVESDDWRILQEALKKGGDILIVAAKDVDEMLKQVEILRRLGAKQIAVESSDWRILQEALK
KGGDILIVAGGGMTITFRGDDLEALLKAAIEMIKQALKFGATITLSLDGNDLNINITGVPEQVRKELAKEAE
RLAKEFGITVTRTGGGDVDEMLKQVEILRRLGAKQIAVESDDWRILQEALKKGGSAWSHPQFEK

PLZ1.4 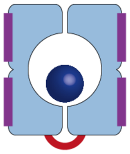 **E31+E154+E200+E323+N52F+N221A+W175A+W6A**

D9N+E113Q+D132N+D156N+D178N+E282Q+D301N+D325N

PLZ1.4 **C-Strep** (pET17b vector)

MW = 38.366 kDa **ε_{280} [M^{-1}cm^{-1}] = 27500**

ATGGGTGACATTCTGATAGTGGCCGCCAAAAACGTTGATGAGATGCTCAAGCAGGTCGAAATTTTGCG
TCGCTTGGGCGCGAAACAGATTGCCGTTGAGAGTAGTGACTGGCGCATACTGCAGGAAGCGCTTAAGA
AGGGTGGTGATATTCTTATTGTATTTGGTGGCGGGATGACCATTACCTTCCGCGGGGATGACCTGGAA
GCCTTGCTCAAAGCAGCTATAGAAATGATAAAGCAGGCACTTAAGTTCGGGGCCACTATAACATTGAG
CCTTGACGGAAACGACCTGAATATTAACATTACCGGCGTCCCAGAGCAGGTCCGCAAGGAGTTGGCTA
AACAGGCCGAGCGGCTGGCTAAGGAATTCGGGATCACAGTGACTCGAACCGGTGGCGGAAACGTGGA
CGAGATGCTCAAACAGGTGGAGATACTTAGGAGACTCGGGGCCAAGCAGATCGCCGTGGAGTCTAAC
GACTGGCGAATACTGCAGGAAGCGTTGAAGAAAGGTGGCGACATTCTGATTGTCGCGGCTAAGAACG
TGGATGAAATGCTCAAGCAGGTCGAAATCCTTAGGCGTCTCGGCGCCAAACAGATTGCTGTCGAATCC
AGTGACTGGAGAATTCTCCAGGAAGCGCTCAAGAAGGGCGGTGATATCCTTATCGTTGCGGGCGGCGG
CATGACAATTACTTTTCGGGGCGACGACCTTGAAGCCCTGCTGAAGGCCGCCATTGAGATGATTAAAC
AAGCGCTGAAATTCGGCGCAACCATCACACTCTCCTTGGACGGCAACGATCTGAACATCAACATCACA
GGAGTGCCAGAACAGGTTCGTAAAGAACTGGCGAAACAGGCCGAAAGGCTGGCCAAGGAATTTGGTA
TCACAGTTACACGTACGGGTGGAGGCAACGTTGATGAGATGCTGAAACAAGTCGAGATCCTCCGCCGT
CTTGGTGCAAAACAAATCGCAGTGGAGTCCAACGACTGGCGTATTCTTCAAGAGGCGCTTAAGAAAGG
TGGATCCGCTTGGAGCCACCCGCAGTTCGAAAAATAA

MGDILIVAAKNVDEMLKQVEILRRLGAKQIAVESSDWRILQEALKKGGDILIVFGGGMTITFRGDDLEALL
KAAIEMIKQALKFGATITLSLDGNDLNINITGVPEQVRKELAKQAERLAKEFGITVTRTGGGNVDEMLKQV
EILRRLGAKQIAVESNDWRILQEALKKGGDILIVAAKNVDEMLKQVEILRRLGAKQIAVESSDWRILQEALK
KGGDILIVAGGGMTITFRGDDLEALLKAAIEMIKQALKFGATITLSLDGNDLNINITGVPEQVRKELAKQAE
RLAKEFGITVTRTGGGNVDEMLKQVEILRRLGAKQIAVESNDWRILQEALKKGGSAWSHPQFEK

Lpp-OmpA-PLZ1.1-HA 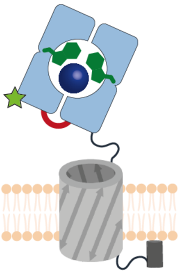 **Lpp** **OmpA** **TEV** **PLZ1.1** **C-Strep** **HA**

(pET29b vector)

MW = 56.619 kDa **ε_{280} [M^{-1}cm^{-1}] = 74370**

ATGAAAGCTACTAAACTGGTACTGGGCGCGGTAATCCTGGGTTCTACTCTGCTGGCAGGTTGCTCCAG
CAACGCTAAAATCGATCAGGGCATTAACCCGTATGTTGGCTTTGAAATGGGTTACGACTGGTTAGGTC
GTATGCCGTACAAAGGCAGCGTTGAAAACGGTGCATACAAAGCTCAGGGCGTTCAACTGACCGCTAA
ACTGGGTTACCCAATCACTGACGACCTGGACATCTACACTCGTCTGGGTGGTATGGTATGGCGTGCAG
ACACTAAATCCAACGTTTATGGTAAAAACCACGACACCGGCGTTTCTCCGGTCTTCGCTGGCGGTGTTG
AGTACGCGATCACTCCTGAAATCGCTACCCGTCTGGAATACCAGTGGACCAACAACATCGGTGACGCA
CACACCATCGGCACTCGTCCGGACAACGGCATTCCGGGCGAGAATCTTTATTTTCAGGGCGCTAGTGC
AGGTGACATTCTGATAGTGTGGGCCAAAGACGTTGATGAGATGCTCAAGCAGGTCGAAATTTTGCGTC

GCTTGGGCGCGAAACAGATTGCCGTTGAGAGTAGTGACTGGCGCATACTGCAGGAAGCGCTTAAGAA
GGGTGGTGATATTCTTATTGTAAATGGTGGCGGGATGACCATTACCTTCCGCGGGGATGACCTGGAAG
CCTTGCTCAAAGCAGCTATAGAAATGATAAAGCAGGCACTTAAGTTCGGGGCCACTATAACATTGAGC
CTTGACGGAAACGACCTGAATATTAACATTACCGGCGTCCCAGAGCAGGTCCGCAAGGAGTTGGCTAA
AGAGGCCGAGCGGCTGGCTAAGGAATTCGGGATCACAGTGACTCGAACCGGTGGCGGAGATGTGGAC
GAGATGCTCAAACAGGTGGAGATACTTAGGAGACTCGGGGCCAAGCAGATCGCCGTGGAGTCTGATG
ACTGGCGAATACTGCAGGAAGCGTTGAAGAAAGGTGGCGACATTCTGATTGTCTGGGCTAAGGACGT
GGATGAAATGCTCAAGCAGGTCGAAATCCTTAGGCGTCTCGGCGCGAAACAAATTGCTGTCGAATCCT
CTGATTGGAGAATTCTCCAGGAAGCCCTCAAGAAGGGCGGCGACATCCTTATCGTTAACGGCGGCGGC
ATGACAATTACTTTTCGGGGCGACGACCTTGAAGCCCTGCTGAAGGCCGCCATTGAGATGATTAAACA
GGCCCTTAAATTCGGCGCAACCATCACACTCTCCTTGGACGGCAACGATCTGAACATCAACATCACAG
GAGTGCCAGAACAGGTTCGTAAAGAACTGGCGAAAGAGGCCGAAAGGCTGGCCAAGGAATTTGGTAT
CACAGTTACAAGAACTGGAGGAGGCGATGTTGATGAGATGCTCAAGCAAGTCGAAATCCTCAGGCGT
CTCGGTGCCAAACAGATTGCTGTTGAGTCCGACGATTGGAGAATCCTTCAGGAAGCCCTTAAGAAGGG
CGGATCCGCTTGGAGCCACCCGCAGTTCGAAAAATATCCATATGATGTTCCAGATTATGCTTAA

MKATKLVLGAVILGSTLLAGCSSNAKIDQGINPYVGFEMGYDWLGRMPYKGSVENGAYKAQGVQLTAKL
GYPITDDLDIYTRLGGMVWRADTKSNVYGKNHDTGVSPVFAGGVEYAITPEIATRLEYQWTNNIGDAHTI
GTRPDNGIPGENLYFQGASAGDILIVWAKDVDEMLKQVEILRRLGAKQIAVESSDWRILQEALKKGGDILIV
NGGGMTITFRGDDLEALLKAAIEMIKQALKFGATITLSLDGNDLNINITGVPEQVRKELAKEAERLAKEFGI
TVTRTGGGDVDEMLKQVEILRRLGAKQIAVESDDWRILQEALKKGGDILIVWAKDVDEMLKQVEILRRLG
AKQIAVESSDWRILQEALKKGGDILIVNGGGMTITFRGDDLEALLKAAIEMIKQALKFGATITLSLDGNDLN
INITGVPEQVRKELAKEAERLAKEFGITVTRTGGGDVDEMLKQVEILRRLGAKQIAVESDDWRILQEALKK
GGSAWSHPQFEKYPYDVPDYA

Lpp-OmpA-HA 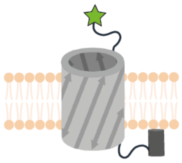 **Lpp OmpA TEV HA**
(pET29b vector)

MW = 17.925 kDa ε_{280} [$M^{-1}cm^{-1}$] = 35870

ATGAAAGCTACTAAACTGGTACTGGGCGCGGTAATCCTGGGTTCTACTCTGCTGGCAGGTTGCTCCAG
CAACGCTAAAATCGATCAGGGCATTAACCCGTATGTTGGCTTTGAAATGGGTTACGACTGGTTAGGTC
GTATGCCGTACAAAGGCAGCGTTGAAAACGGTGCATACAAAGCTCAGGGCGTTCAACTGACCGCTAA
ACTGGGTTACCCAATCACTGACGACCTGGACATCTACACTCGTCTGGGTGGTATGGTATGGCGTGCAG
ACACTAAATCCAACGTTTATGGTAAAAACCACGACACCGGCGTTTCTCCGGTCTTCGCTGGCGGTGTTG
AGTACGCGATCACTCCTGAAATCGCTACCCGTCTGGAATACCAGTGGACCAACAACATCGGTGACGCA
CACACCATCGGCACTCGTCCGGACAACGGCATTCCGGGCGAGAATCTTTATTTTCAGGGCTATCCATAT
GATGTTCCAGATTATGCTTAA

MKATKLVLGAVILGSTLLAGCSSNAKIDQGINPYVGFEMGYDWLGRMPYKGSVENGAYKAQGVQLTAKL
GYPITDDLDIYTRLGGMVWRADTKSNVYGKNHDTGVSPVFAGGVEYAITPEIATRLEYQWTNNIGDAHTI
GTRPDNGIPGENLYFQGYPYDVPDYA

PedH **F412V+W561A** **PedH** **His-tag** (pET29b vector)

M_W = 63.097 kDa **ε_{280} [$M^{-1}cm^{-1}$] = 141415**

Note: PedH has been engineered previously to accept larger substrates. Mutations F412V and W561A enlarge the substrate channel.[4] This variant is referred to as "PedH" in our work.

ATGGCAGTGTCTAACGAGGAGATTCTGCAAGATCCAAAGAATCCACAACAAATTGTAACGAACGGAT
TAGGAGTACAAGGTCAACGTTATTCGCCTTTGGATTTGCTGAACGTTAACAATGTGAAAGAATTGCGT
CCTGTATGGGCTTTCTCGTTTGGTGGCGAGAAACAACGTGGTCAACAAGCGCAACCATTAATTAAAGA
TGGCGTTATGTATCTCACGGGAAGCTATAGCCGCGTATTTGCGGTTGACGCACGTACTGGTAAGAAGT
TGTGGCAGTATGACGCCCGTCTCCCCGACGATATTCGTCCATGTTGTGATGTTATTAATCGTGGGGTGG
CCCTTTATGGTAATTTAGTATTCTTTGGAACTCTCGATGCGAAACTTGTAGCGCTTAATAAAGATACGG
GTAAAGTAGTTTGGTCAAAGAAAGTAGCGGATCATAAAGAGGGATATTCGATTAGTGCTGCCCCCATG
ATTGTGAACGGAAAATTGATTACCGGTGTAGCAGGTGGTGAATTTGGGGTTGTCGGGAAAATTCAAGC
ATATAATCCCGAAAATGGTGAGTTATTGTGGATGCGTCCTACAGTCGAGGGCCACATGGGTTACGTAT
ATAAAGACGGTAAAGCTATTGAAAATGGCATCAGTGGCGGCGAAGCCGGGAAAACGTGGCCAGGGGA
TCTCTGGAAAACTGGTGGTGCGGCCCCCTGGTTAGGCGGCTATTATGATCCGGAGACGAATTTGATTTT
ATTCGGGACGGGGAATCCTGCACCTTGGAATAGCCATTTGCGTCCAGGCGATAATCTTTATAGCTCGTC
ACGTCTTGCCCTTAATCCAGATGATGGTACGATTAAATGGCATTTTCAATCGACACCCCACGATGGTTG
GGATTTTGATGGTGTGAATGAACTCATTAGTTTTAATTATAAAGATGGTGGAAAAGAAGTGAAAGCAG
CAGCTACCGCGGATCGTAATGGCTTCTTCTATGTATTAGATCGTACGAATGGTAAATTTATTCGTGGTT
TTCCTTTTGTAGATAAAATTACTTGGGCGACCGGTCTGGATAAAGATGGGCGCCCCATTTATAATGATG
CAAGTCGTCCTGGGGCCCCGGGGTCGGAAGCGAAAGGGTCATCCGTATTTGTGGCCCCAGCGGTACTG
GGTGCGAAGAATTGGATGCCAATGGCGTATAATAAAGATACGGGCTTGTTTTATGTACCAAGTAATGA
ATGGGGAATGGATATTTGGAATGAGGGAATTGCATACAAGAAGGGCGCCGCCTTTCTGGGCGCGGGG
TTTACTATTAAACCACTTAACGAGGATTATATTGGTGTTCTTCGTGCAATTGATCCCGTTTCCGGGAAA
GAGGTCTGGCGTCATAAGAATTACGCCCCATTATGGGGTGGCGTTCTTACTACGAAAGGGAATTTAGT
GTTTACTGGAACTCCTGAAGGGTTTCTTCAAGCGTTTAATGCCAAAACTGGGGATAAAGTTTGGGAGT
TTCAAACTGGTAGCGGTGTATTGGGGTCACCGGTGACGTGGGAGATGGATGGTGAACAGTATGTCTCC
GTGGTGTCGGGTTGGGGTGGTGCAGTCCCTTTAGCGGGTGGAGAGGTAGCGAAGCGTGTGAAAGATTT
TAATCAAGGTGGAATGTTGTGGACATTTAAACTGCCGAAACAACTGCAACAGACCGCCTCGGTTAAAC
CGCTCGAGCACCACCACCACCACCACTGA

MAVSNEEILQDPKNPQQIVTNGLGVQGQRYSPLDLLNVNNVKELRPVWAFSFGGEKQRGQQAQPLIKDGV
MYLTGSYSRVFAVDARTGKKLWQYDARLPDDIRPCCDVINRGVALYGNLVFFGTLDAKLVALNKDTGKV
VWSKKVADHKEGYSISAAPMIVNGKLITGVAGGEFGVVGKIQAYNPENGELLWMRPTVEGHMGYVYKD
GKAIENGISGGEAGKTWPGDLWKTGGAAPWLGGYYDPETNLILFGTGNPAPWNSHLRPGDNLYSSSRLAL
NPDDGTIKWHFQSTPHDGWDFDGVNELISFNYKDGGKEVKAAATADRNGFFYVLDRTNGKFIRGFPFVDK
ITWATGLDKDGRPIYNDASRPGAPGSEAKGSSVFVAPAVLGAKNWMPMAYNKDTGLFYVPSNEWGMDI
WNEGIAYKKGAAFLGAGFTIKPLNEDYIGVLRAIDPVSGKEVWRHKNYAPLWGGVLTTKGNLVFTGTPEG
FLQAFNAKTGDKVWEFQTGSGVLGSPVTWEMDGEQYVSVVSGWGGAVPLAGGEVAKRVKDFNQGGML
WTFKLPKQLQQTASVKPLEHHHHHH

S2.4 Expression and purification protocols

PhotoLanZymes (PLZ)

Strep wash buffer	Strep elution buffer	FPLC buffer
40 mM HEPES, pH 8.0<br>150 mM NaCl<br>1 mM EDTA	40 mM HEPES, pH 8.0<br>150 mM NaCl<br>1 mM EDTA<br>2.5 mM Desthiobiotin	25 mM HEPES, pH 8.5<br>100 mM NaCl

Expression: A 5 L *Erlenmeyer* flask containing LB medium (1.5 L) and the respective antibiotic was inoculated to an OD_{600} of 0.05–0.08 with a pre-culture of *E. coli* BL21-Gold(DE3) or *E. coli* SoluBL21 harbouring the expression vector for the protein of interest. **PLZ1.0:** The culture was incubated at 37 °C (180 rpm, orbit diameter 2.5 cm) until reaching an OD_{600} of 0.4. Incubation was continued at 18 °C until reaching an OD_{600} of 0.7, gene expression was induced with isopropyl-β-D-thiogalactopyranosid (IPTG, final concentration 0.5 mM) and incubation was carried out for additional 16 h. Cells were harvested by centrifugation (45 min, 4100 rcf, 4 °C) and stored at -20 °C as wet cell pellet. **Single-chain constructs (PLZ1.1–1.4):** The culture was incubated at 37 °C (180 rpm, orbit diameter 2.5 cm) until reaching an OD_{600} of 0.7, gene expression was induced with IPTG (final concentration 0.5 mM) and incubation was carried out for additional 5.5 h at 37 °C. Cells were harvested by centrifugation (45 min, 4100 rcf, 4 °C) and stored at -20 °C as wet cell pellet.

Cell lysis: The frozen cell pellet was thawed and resuspended in Strep wash buffer (40 mL) containing DNase I (0.05 mg/mL, *AppliChem* GmbH, ≥ 3000 U/mg) and Lysozyme (0.5 mg/mL, *Carl Roth* GmbH + Co. KG, ≥45000 FIP U/mg) at 4 °C and incubated for 30 min on ice. The cells were lysed either *via* sonication using a *Branson* SFX 500 sonifier (*Emerson Electric* Co., St. Louis, MO, USA; power-on time: 10 min, pulsed time: 5 s on, 7 s off, 35% power) or *via* French press (Constant Cell, *Constant Systems*, Daventry, United Kingdom). After centrifugation (25 min, 11,000 rcf, 4 °C), the clarified cell lysate was used for affinity chromatography.

Strep-Tactin gravity flow column: The column was filled with Strep-Tactin Superflow high capacity resin (*IBA Lifesciences* GmbH, Göttingen, Germany; 10 mL column volume) and equilibrated with Strep wash buffer (3 x 50 mL). The protein solution was added slowly to the column. The flow-through was collected and loaded onto the column a second time. Afterwards the flow-through was discarded and the column matrix washed with Strep wash buffer (3 x 20 mL). The bound protein was eluted with Strep elution buffer (2 x 10 mL+1 x 15 mL). The combined protein fractions were concentrated using *Merck Millipore* Amicon Ultra centrifugal filter devices (*Merck* KGaA, Darmstadt, Germany; 30 kDa cut-off, 4000 rcf, 4 °C) to a final volume of 5 mL.

Size-exclusion chromatography and buffer exchange: The protein was further purified by size-exclusion chromatography (SEC) using a NGC Quest 10 Plus Chromatography System (*Bio-Rad Laboratories* GmbH, Feldkirchen, Germany) equipped with a multi-wavelength (UV/Vis;

260 nm and 280 nm were recorded) and conductivity detector, a Superdex 75 Increase 10/300 GL column and a HiLoad 16/600 Superdex 75 pg column (*Cytiva*, Marlborough, MA, USA). The purification of proteins was carried out either at room temperature (21 °C) or at 6 °C and the columns were equilibrated with the FPLC buffer beforehand. Following methods were applied for analytical and preparative separation:

	**Analytical separation**	**Preparative separation**	
**Column**	Superdex 75 Increase 10/300 GL	Superdex 75 Increase 10/300 GL	HiLoad 16/600 Superdex 75 pg
**Sample loop size**	100 µL	1 mL	5 mL
**Flow rate [mL/min]**	0.6	0.65	1
**Equilibration [mL]**	2	2	5
**Sample application [mL]**	0.5	2	10
**Elution [mL]**	26	26	129
**Fraction size [mL]**	0.5	0.5	1

Fractions were analyzed by the FPLC chromatogram and SDS-PAGE. Fractions containing the protein of interest with the correct oligomeric state were combined and concentrated using *Merck Millipore* Amicon Ultra centrifugal filter devices (*Merck* KGaA, Darmstadt, Germany; 30 kDa or 10 kDa cut-off, 4000 rcf, 4 °C). The concentrated protein samples were frozen in liquid nitrogen and stored at -70 °C. Protein concentrations were determined by UV absorption at 280 nm using calculated ε_{280} values.

Alcohol dehydrogenase (PedH)

**Ni-NTA wash buffer**	**Ni-NTA elution buffer A**	**FPLC buffer**
25 mM HEPES, pH 7.5<br>300 mM NaCl<br>30 mM Imidazole	25 mM HEPES, pH 7.5<br>300 mM NaCl<br>300 mM Imidazole<br><br>**Ni-NTA elution buffer B**<br><br>25 mM HEPES, pH 7.5<br>300 mM NaCl<br>500 mM Imidazole	25 mM HEPES, pH 8.5<br>100 mM NaCl

Expression: A 5 L *Erlenmeyer* flask containing LB medium (1.5 L) and kanamycin (final concentration of 30 µg/mL) was inoculated with 15 mL of a pre-culture of *E. coli* BL21-Gold(DE3) harboring the expression vector for the protein of interest. The culture was incubated at 37 °C (180 rpm, orbit diameter 2.5 cm) until reaching an OD_{600} of 0.6–0.8 and gene expression was induced with IPTG (final concentration 0.5 mM) followed by incubation at 16 °C for additional 16 h. Cells were harvested by centrifugation (20 min, 4100 rcf, 6 °C) and stored at -20 °C as wet cell pellet.

Cell lysis: The cell pellet of 1.5 L expression culture was divided and resuspended in 2 x 35 mL Ni-NTA wash buffer containing a spatula tip of lysozyme (*Carl Roth* GmbH + Co. KG, ≥45000 FIP U/mg) and DNaseI (*AppliChem* GmbH, ≥ 3000 U/mg). Followed by 30 min incubation on ice, the cells were lysed *via* sonication using a *Branson* SFX 500 sonifier (*Emerson Electric* Co., St. Louis, MO, USA; power-on time: 10 min, pulsed time: 5 s on, 7 s off, 35% power). After centrifugation (40 min, 11,000 rcf, 6 °C), the clarified cell lysate was used for affinity chromatography.

Ni-NTA gravity flow column: The cleared lysate was loaded onto a column packed with 3 mL Ni-NTA agarose resin (*Qiagen* N.V., Venlo, Netherlands) equilibrated with 3–5 column volumes of Ni-NTA wash buffer. The flow-through of the first loading was collected and loaded onto the column a second time. After washing the column with 10 CV Ni-NTA wash buffer, PedH was eluted in 3 CV Ni-NTA elution buffer A. Remaining PedH was eluted in 3 CV Ni-NTA elution buffer B and all elution fractions were combined. The combined protein solutions were concentrated using *Merck Millipore* Amicon Ultra centrifugal filter devices (*Merck* KGaA, Darmstadt, Germany; 30 kDa cut-off, 4000 rcf, 4 °C) to a final concentration of 7–8 mg/mL.

Size-exclusion chromatography and buffer exchange: The protein was further purified by SEC using a Superdex 200 Increase 10/300 GL column (*Cytiva*, Marlborough, MA, USA; sample loop size: 1 mL; flow rate: 0.5 mL/min; equilibration: 2 mL; sample application: 2 mL; elution: 26 mL; fraction size: 0.5 mL; temperature: 6 °C). Fractions were analyzed by the FPLC chromatogram and SDS-PAGE. Fractions containing the protein of interest with the correct oligomeric state were combined and again concentrated using *Merck Millipore* Amicon Ultra centrifugal filter devices (*Merck* KGaA, Darmstadt, Germany; 30 kDa cut-off, 4000 rcf, 4 °C). The concentrated protein samples were frozen in liquid nitrogen and stored at -70 °C.

S3 Procedures for photoenzymatic reactions

All lanthanide chlorides used in this study were purchased as "trace metal basis" with a purity of 99.9% or higher. Aqueous stocks of 200 mM were prepared with deionized water and stored at -20 °C as 1 mL aliquots. All photoreactions were performed in GC vials using the 6-vial photoreactor setup described above.

S3.1 Initial screening for optimal pH and wavelength

For the initial pH screening, a 1:1 buffer mixture of 25 mM CHES (pK_a = 9.49, 25 °C; buffer range: 8.6–10.0) and 25 mM HEPES (pK_a = 7.48, 25 °C; buffer range: 6.8–8.2) in 100 mM NaCl was prepared and the pH of this mixture was adjusted to pH 6.5–9.0.

PLZ1.0 and the $CeCl_3$ were diluted in the buffer mixture to reach a concentration of 22.2 µM (PLZ1.0: dimer concentration). 225 µL of this solution was transferred to a 1.5 mL glass vial and 25 µL of **(*R*,*R*)-1** solution (20 mM in acetonitrile) was added to reach a final concentration of 20 µM enzyme + Ce(III), 2 mM substrate and 10% (*v*/*v*) acetonitrile as co-solvent. Photoreactions were carried out in photoreactors at 435–440 nm (5 W) at approx. 23 °C for 21 h. The yield of the product benzaldehyde (**2**) was quantified by HPLC (**Figure S2A**).

For the wavelength screening the optimal pH 8.5 was chosen for the buffer (25 mM HEPES, 100 mM NaCl, pH 8.5). The photoreaction was carried out as described in the pH screening at different wavelengths and irradiation powers for 19 h. Photoreactions with buffer only, buffer + $CeCl_3$ and PLZ1.0 without $CeCl_3$ served as controls. The yield of the product benzaldehyde (**2**) was quantified by HPLC (**Figure S2B–E**).

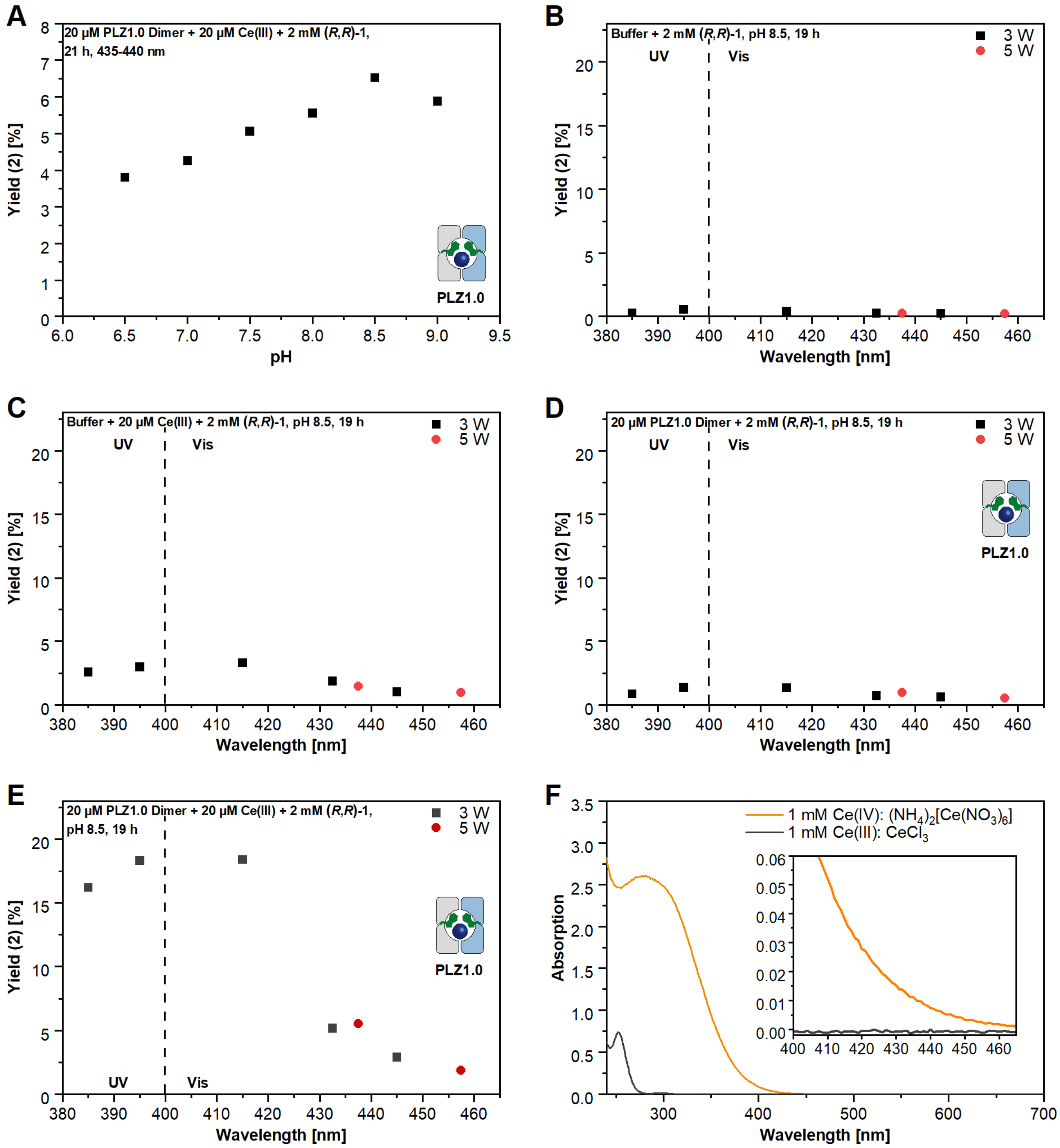

Figure S2. Screening pH and wavelength for photoreactions with PLZ1.0 and ***(R,R)*-1** as the substrate. **(A)** Photocatalytic cleavage of ***(R,R)*-1** in buffers with different pH. **(B–E)** Photocatalytic cleavage of ***(R,R)*-1** at different wavelength and irradiation power including control experiments. **(F)** UV/Vis absorption spectra of $Ce^{III}Cl_3$ and $(NH_4)_2[Ce^{IV}(NO_3)_6]$ in water.

S3.2 General procedure for all photoreactions

Stock solutions of the protein of interest and $CeCl_3$ were diluted in buffer (25 mM HEPES, 100 mM NaCl, pH 8.5) to a concentration of 111 µM (PLZ1.0: dimer concentration; PLZ1.1–PLZ1.4: single-chain concentration → one equivalent Ce(III) per protein scaffold) followed by incubation for 2.5 h at 40 °C. 90 µL of this solution was transferred to a 1.5 mL glass vial and 10 µL of substrate solution (20 mM in acetonitrile) was added to reach a final concentration of 100 µM metalloenzyme, 2 mM substrate and 10% (*v*/*v*) acetonitrile as co-solvent. Photoreactions were carried out in the 6-vial photoreactor setup at 410–420 nm (3 W) at approx. 23 °C for 24 h. For the initial controls, the excess or unspecifically bound Ce(III) was removed after incubation by using a desalting column (PD10, Sephadex G-25 resin, *Cytiva*, Marlborough, MA, USA).

S3.3 Deviations from the general procedure

Addition of TEMPO as a radical scavenger: A substrate solution of 40 mM in acetonitrile and a TEMPO solution of 200 mM in acetonitrile were prepared. 5 µL of each solution were added to 90 µL metalloenzyme solution to reach a final concentration of 100 µM metalloenzyme, 2 mM substrate, 10 mM TEMPO and 10% (*v*/*v*) acetonitrile as co-solvent.

Reactions in the absence of O_2: Water and acetonitrile were degassed by three repetitions of a freeze-pump-thaw cycle. The protein was diluted in buffer (25 mM HEPES, 100 mM NaCl, pH 8.5) to reach a concentration of 125 µM (PLZ1.0: dimer concentration; PLZ1.1–PLZ1.4: single-chain concentration) followed by lyophilization overnight. Lyophilized enzyme, $CeCl_3$ and substrates were transferred as solids into a glove box (<0.3 ppm O_2) as well as the degassed water and acetonitrile as frozen liquids. A $CeCl_3$ stock solution (1.25 mM) and a substrate stock solution (20 mM) were prepared by either adding the degassed water or acetonitrile, respectively. The enzyme was rehydrated by adding degassed water to a concentration of 125 µM. 80 µL of enzyme solution and 10 µL of $CeCl_3$ solution were mixed to reach a concentration of 111 µM metalloenzyme. After incubation, 10 µL of substrate solution (20 mM) was added, the reaction vial air-tight sealed, and the photoreactions carried out as described in the general procedure.

Alcohol dehydrogenase PedH: All photoreactions involving the alcohol dehydrogenase PedH from *P. putida* KT2440 were performed with 2.5% (*v*/*v*) acetonitrile as co-solvent. Therefore, the substrate stock solution (20 mM) was prepared in a mixture of buffer (25 mM HEPES, 100 mM NaCl, pH 8.5) and acetonitrile [75:25 (*v*/*v*)]. For reasons of comparability all other experiments in the context of PedH were also performed with 2.5% (*v*/*v*) acetonitrile as co-solvent. Samples were taken after 6 h and 24 h.

S3.4 HPLC-based quantification of substrates and products

UV absorption: Product yields after photocatalysis were determined by HPLC using Method 1 (achiral column). One reaction volume of acetonitrile containing the internal standard 1,2,3-trimethoxybenzene (TMB, 200 µM) was added after the photoreaction to precipitate the protein. After short mixing of the sample, the denatured protein was separated by centrifugation (8 min, 12066 rcf, RT) and the supernatant was used for quantification via HPLC. If necessary, reaction samples were diluted 1:4 before quantification. The final concentration of the internal standard TMB was always 100 µM and amounts of product or starting material were calculated by the area ratio of the compound (at 254 nm or 205 nm) and the internal standard (at 205 nm) using following calibration curves (**Figure S3**).

Evaluation of experimental errors: The experimental error when determining product yields by HPLC repeatedly from the identical sample was ca. 1%. The error when irradiating identical reaction mixtures in different positions in the photoreactor was up to 3%. Biological repeats from different protein batches were subject to stronger variation. Here, first the activity towards the standard substrate **(*R*,*R*)-1** was tested. If the error was within 10%, the new protein batch was used for other experiments.

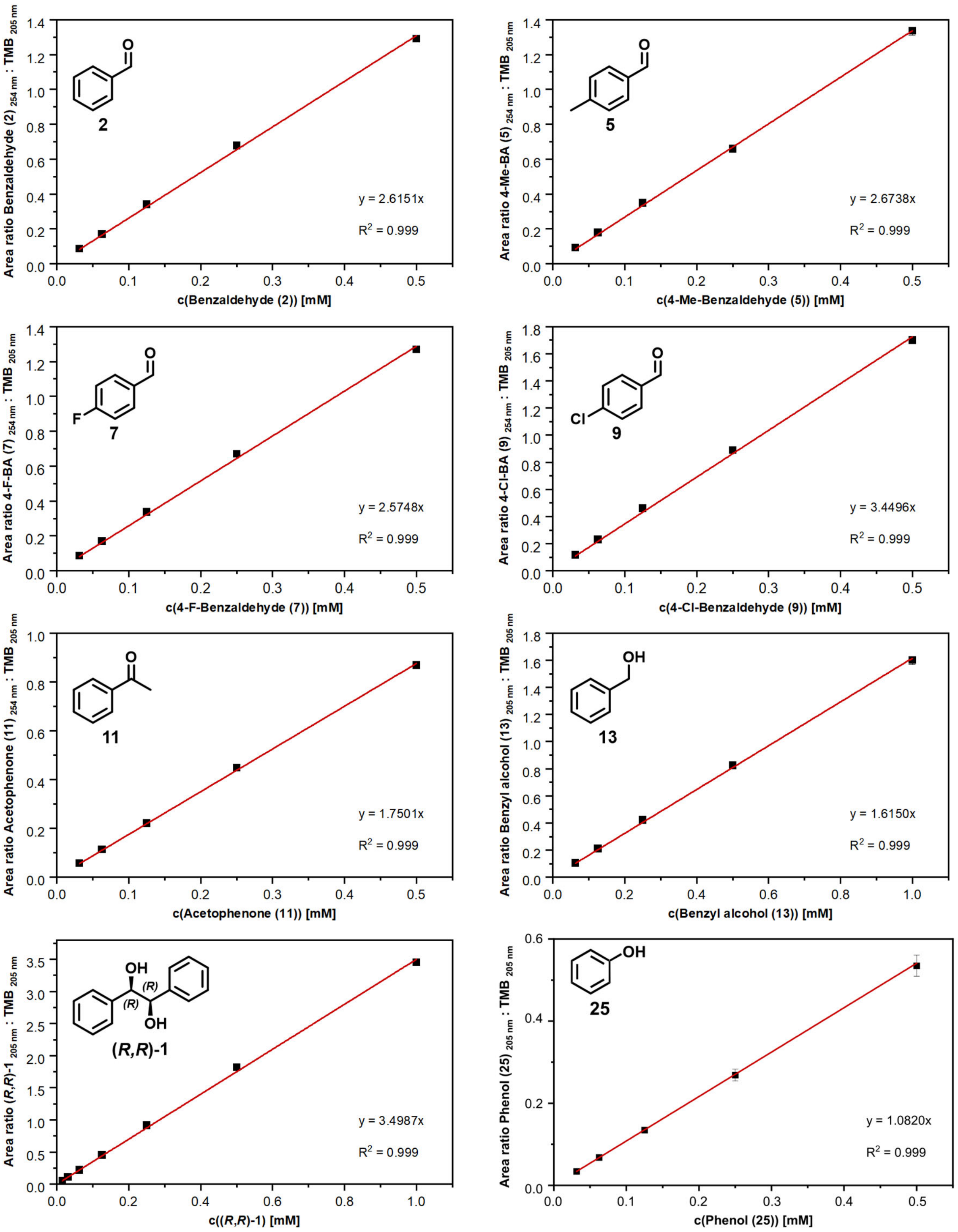

Figure S3. Calibration curves for the quantification of substrates and products via UV absorption, using TMB as the internal standard in HPLC runs. All data points were measured as triplicates. [TMB: 1,2,3-Trimethoxybenzene; BA: Benzaldehyde]

Derivatization with 2-aminobenzamidoxime (ABAO, 34):

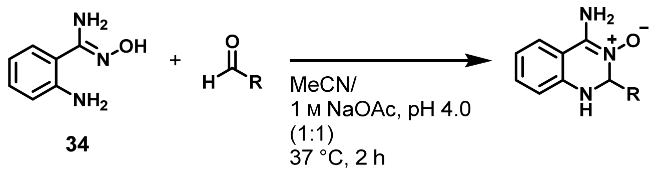

Aldehyde products that were not detectable by UV absorption were derivatized using ABAO (**34**) by a modified protocol from *Ressmann et al.* (2019).[5] After completion of the photoreaction, acetonitrile withinternal standard (TMB) was added and precipitated protein was removed by centrifugation. 190 µL of the supernatant were mixed with 10 µL ABAO (**34**) solution [100 mM ABAO (**34**) in acetonitrile/1 M NaOAc pH 4.0, 1:1] to yield a final concentration of 5 mM ABAO (**34**). The mixture was incubated at 37 °C for 2 h to ensure full conversion of the aldehyde. The mixture was centrifuged again to remove possible precipitate and subsequently subjected to HPLC analysis. The ABAO (**34**) derivatives were detected at 230 nm using HPLC Method 1 (S1 General Methods). Amounts of product were calculated by the area ratio of the ABAO (**34**)-derivatized compound (at 230 nm) and the internal standard 1,2,3-Trimethoxybenzene (TMB, 205 nm) using the calibration curves shown in **Figure S4**.

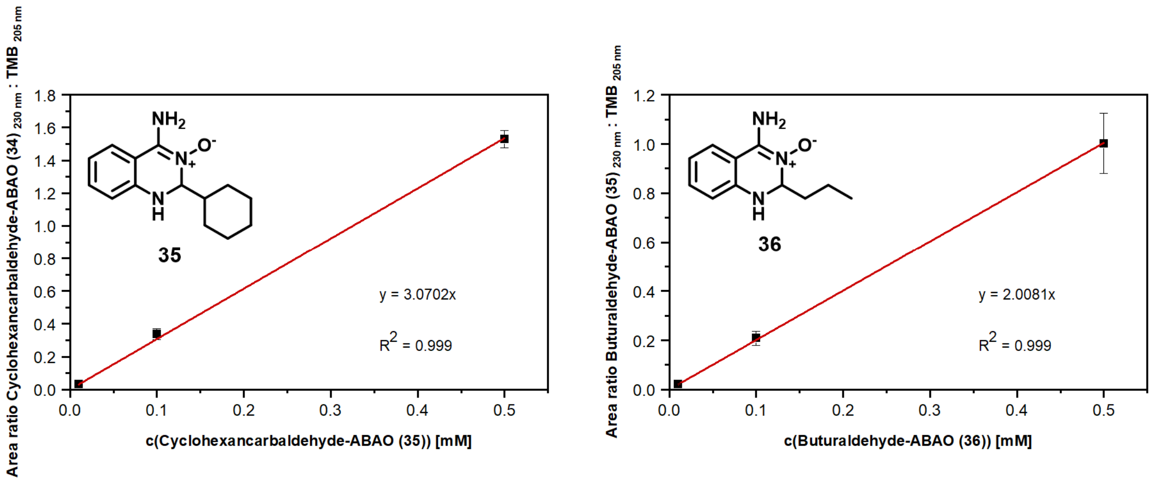

Figure S4. Calibration curves for quantification of ABAO(**34**)-derivatized compounds **35** and **36** (originating from cleavage of substrates **15** and **17**) via HPLC. All data points were measured as duplicates. [TMB: 1,2,3-Trimethoxybenzene]

S3.5 Time course measurements and enzyme kinetics

To determine substrate conversion and product formation over time, samples were prepared as described in the general procedure, but at defined time points (**Figure S5**).

Control experiment to test for a radical chain reaction: Light on/off time courses were set up as described in the general procedure. The volume of the reaction mixture was increased to 160 µL per reaction vial and light-on (2 h) and light-off (1 h) periods alternated. 20 µL sample was taken out of the reaction mixture before and after a light-off period to determine the yield of benzaldehyde (**2**) and the remaining ***(R,R)*-1**. Buffer and Buffer + Ce(III) were used as control reactions. Product formation was found to be strictly light-dependent, thus excluding mechanisms that involve a radical chain reaction in the dark (**Figure S6**).

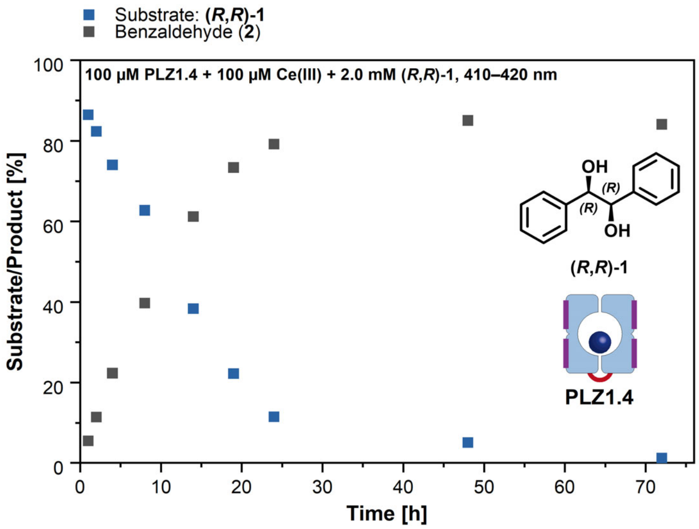

Figure S5. Time-dependent substrate conversion and product formation in the photocatalytic cleavage of ***(R,R)*-1** by PLZ1.4 at 410–420 nm irradiation.

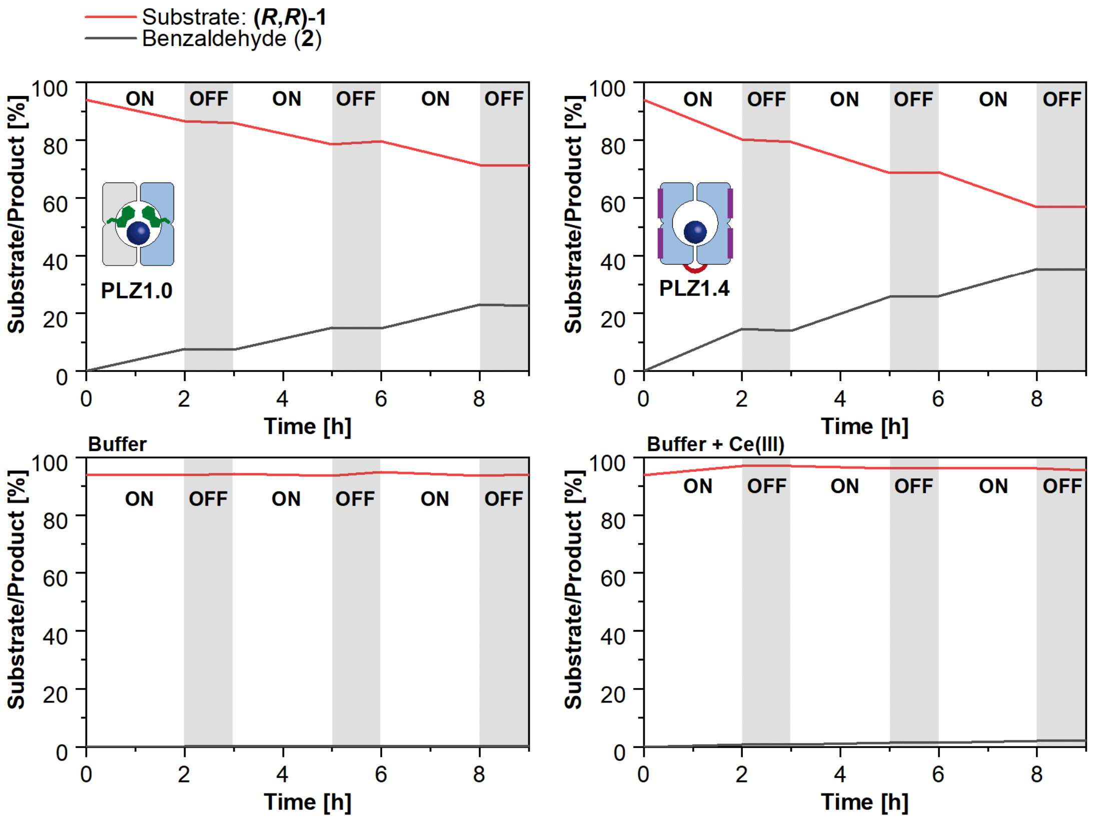

Figure S6. Light on/off kinetics with PLZ1.0 and PLZ1.4 and ***(R,R)*-1** as the substrate. Buffer and Buffer + Ce(III) were used as controls. All experiments were performed with irradiation at 410–420 nm.

Michaelis-Menten kinetics for (*R*,*R*)-1: 27.8 µM PLZ1.4 was incubated with one equivalent $CeCl_3$ for 2.5 h at 40 °C. After addition of 10 µL substrate solution (0–120 mM in acetonitrile) to 90 µL of metalloenzyme, the photoreactions were carried out in 6-vial photoreactors at 410-420 nm (3 W) at approx. 23 °C for 2 h with a final concentration of 25 µM metalloenzyme, 0–12 mM substrate and 10% (*v*/*v*) acetonitrile as co-solvent. The initial velocities of benzaldehyde (**2**) formation were quantified in triplicates, whereby a formation of two benzaldehyde (**2**) molecules was assumed for the conversion of one substrate molecule **(*R*,*R*)-1**. The kinetic parameters k_{cat} and K_M were determined by fitting the data with the Michaelis Menten equation, with $[E]_0$ = enzyme concentration and $[S]_0$ = substrate concentration:

$$k_{obs} = \frac{v}{[E]_0} = \frac{k_{cat}\ [S]_0}{K_M + [S]_0}$$

S3.6 Ce(IV) regeneration

As Ce(IV) recycling is essential for a productive catalytic cycle, we assessed whether initial incubation of the enzyme with Ce(IV) instead of Ce(III), or the addition of the cocatalyst 9,10-diphenylanthracene (DPA) would increase yields for the photocatalytic cleavage of **(*R*,*R*)-1** by PLZ1.0. Photoreactions were set up according to the standard procedure with minor changes: For the incubation with Ce(IV), $(NH_4)_2Ce(NO_3)_6$ was added to the pre-incubation instead of $CeCl_3$. After incubation, 5 µL of an 80 mM stock of **(*R*,*R*)-1** were added to the enzyme and the volume was filled to 100 µL by adding either 5 µL acetonitrile or 5 µL of a 2 mM stock of DPA in acetonitrile. After illumination, the analytes were separated according to HPLC Method 1 (S1, achiral column). All measurements were performed in triplicates.

Figure S7. shows the results. Addition of Ce(IV) instead of Ce(III) lowered the yield by 35%. This is presumably due to the rapid formation of cerium oxide nanoparticles from Ce(IV) salts in aqueous solution, as reported by Pettinger *et al.*[6] The addition of DPA, which was reported to enhance the turnover of the catalytic Ce(III)/Ce(IV) cycle did not increase yields either.[7] This might be due to the large size of DPA, which excludes it from the active site of the enzyme. Addition of more equivalents of DPA was not possible due to the poor solubility in acetonitrile and aqueous buffers.

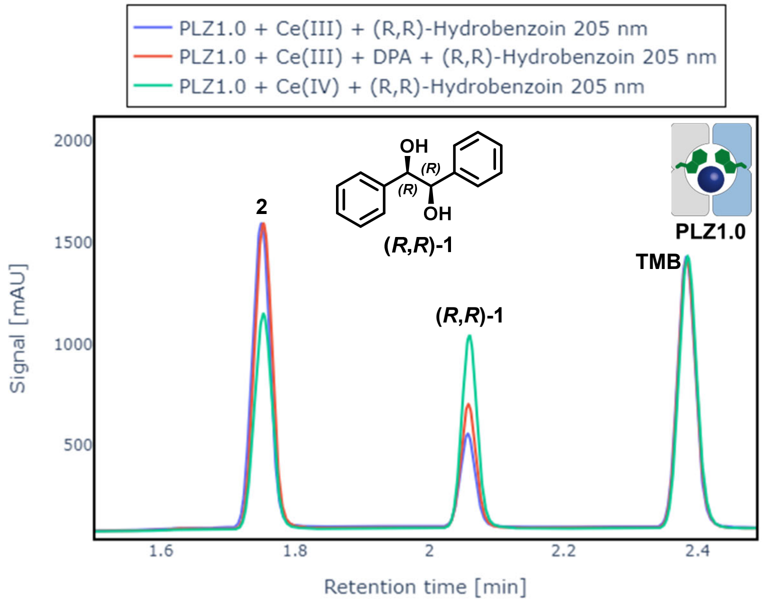

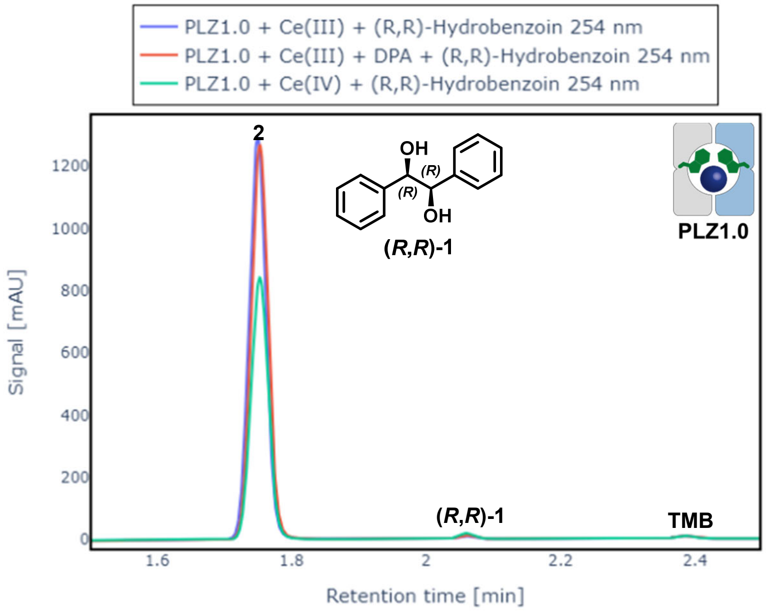

Reaction	Area Benzaldehyde (2) [mAU*min]	Area 1,3,5-Trimethoxybenzene (TMB) [mAU*min]	Yield Benzaldehyde (2)
PLZ1.0 + Ce(III)	38.582	42.378	70 ± 1 %
PLZ1.0 + Ce(IV)	25.718	42.591	46 ± 2 %
PLZ1.0 + Ce(III) + DPA	37.596	42.659	68 ± 2 %

Figure S7. Experiments on the regeneration of Ce(IV). Photocatalytic cleavage of ***(R,R)*-1** by PLZ1.0 incubated with either $Ce^{III}Cl_3$ or $(NH_4)_2Ce^{IV}(NO_3)_6$ and in the presence of DPA as a potential co-catalyst. Absorption at 205 nm (upper panel) and 254 nm (lower panel). Measurements were performed in triplicates.

S4 Photostability studies

PLZ variants: The enzymes were prepared as described in the general procedures for photocatalysis, but with 200 µL sample volume. After incubation with $CeCl_3$, 100 µL of the sample was kept in the dark at 4 °C, whereas the other 100 µL was irradiated in the photoreactors at 410–420 nm (3 W) at approx. 23 °C for 24 h.

PedH: The enzyme was prepared as described in the general procedures for photocatalysis. Additional samples containing BSA were prepared as controls. The samples were either kept in the dark at approx. 23 °C or irradiated in the photoreactors at 410–420 nm (3 W) at approx. 23 °C for 6 h. The enzyme's cofactor pyrroloquinoline quinone (PQQ, disodium salt) at a final concentration equal to that of PedH was added to the samples where desired.

The following techniques were used to analyze photostability.

- SDS-PAGE and analytical size-exclusion chromatography: Analytical SEC was performed as described in S2.4 for protein purification. For SDS-PAGE analysis, 3.8 µg protein per sample was separated using a 4–20% Mini-PROTEAN TGX Precast Protein Gel (*Bio-Rad Laboratories* GmbH, Feldkirchen, Germany).

- Photocatalysis after pre-irradiation: To investigate the residual diol cleavage activity of different protein constructs after 24 h irradiation the enzymes were prepared as described in the general procedures for photocatalysis. After incubation with $CeCl_3$ the substrate (*R*,*R*)-Hydrobenzoin [**(*R*,*R*)-1**] was either added directly to the reaction mixture or the reaction mixture was first pre-irradiated for 24 h at 410–420 nm (3 W) and afterwards the photocatalysis was started by the addition of substrate. In both experiments the product yield was determined after 24 h photocatalysis reaction.

- Protein mass spectrometry: Experiments were performed using electrospray ionization on a SYNAPT XS High Resolution Mass Spectrometer (*Waters*, Milford, MA, USA). 2 µL of a 0.1 mg/mL protein sample were injected and separated on an ACQUITY UPLC Protein BEH C4 column (*Waters*, Milford, MA, USA) using a linear gradient of 5% (*v*/*v*) to 85% (*v*/*v*) acetonitrile in water with a flow rate of 0.4 mL/min at 65 °C. Acetonitrile and water were supplied with 0.1% (*v*/*v*) formic acid to acidify the samples. Results were analyzed using MassLynx v4.2 (*Waters*, Milford, MA, USA).

Results: Known oxidation pathways of tryptophan and methionine typically cause increases of +16, +32, and +4 m/z in the mass spectra (**Figure S8**).[8,9] For PLZ1.0, these increases can be observed to a great extent when zooming into the main peak of the spectrum. The observable photodamage was reduced in PLZ1.4 by removing tryptophan residues near the active site. **Figure S9** shows the mass spectra of PLZ1.0 and PLZ1.4 before and after illumination.

PedH precipitated during the irradiation. This led to a reduced protein concentration in the samples for SDS-PAGE and protein ESI-MS, making it difficult to unambiguously detect degraded or damaged PedH species. The presence of PQQ even intensified the photodamage. This may be due to the fact that PQQ itself absorbs visible light and can form radicals.[10] **Figure S10** shows the SDS-PAGE gel of PedH and BSA, ESI-MS data of PedH before and after irradiation, and the gel-like precipitate formed upon long-term irradiation of PedH.

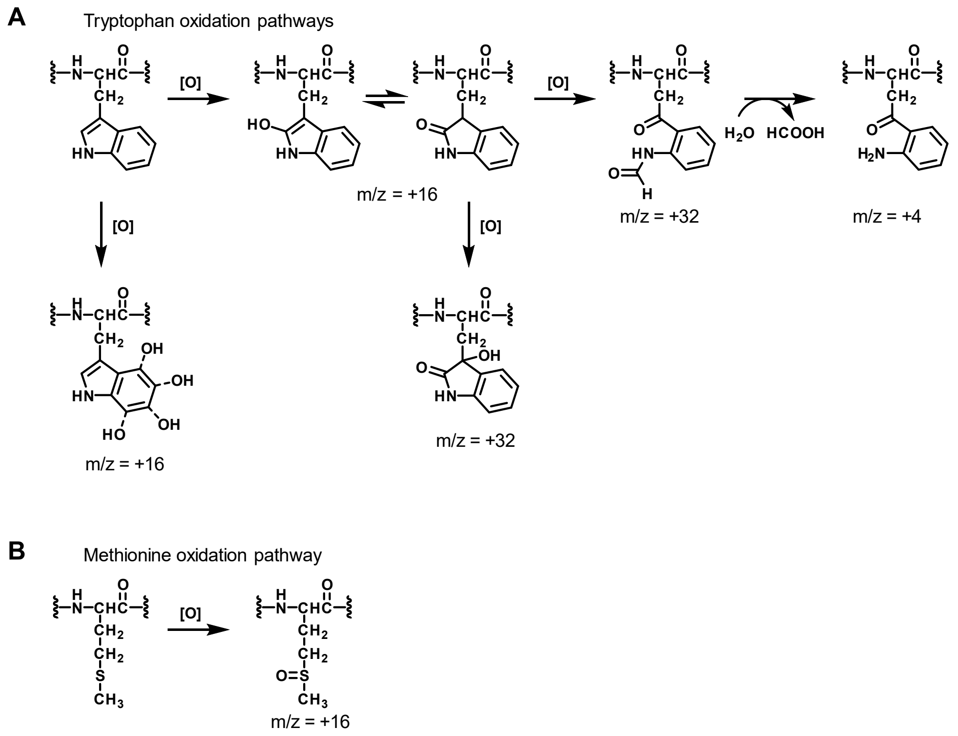

Figure S8. Known oxidative modifications of tryptophan **(A)** and methionine **(B)** and commonly detected mass shifts in protein mass spectrometry.

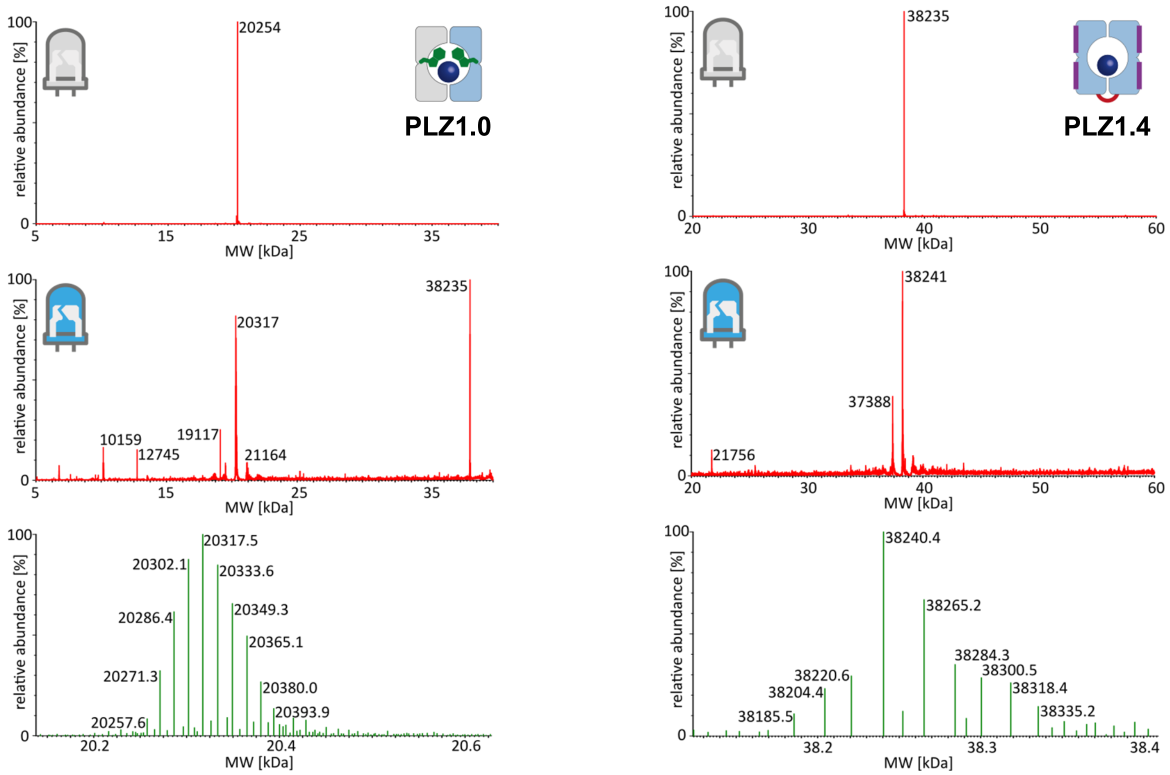

Figure S9. Mass spectrometry analysis of PLZ1.0 (left) and PLZ1.4 (right) before and after irradiation. The bottom row shows detailed mass spectra zoomed into the main peak of the illuminated sample. Expected masses: PLZ1.0: 20254.4 Da; PLZ1.4: 38234.5 Da (N-terminal methionine is cleaved)

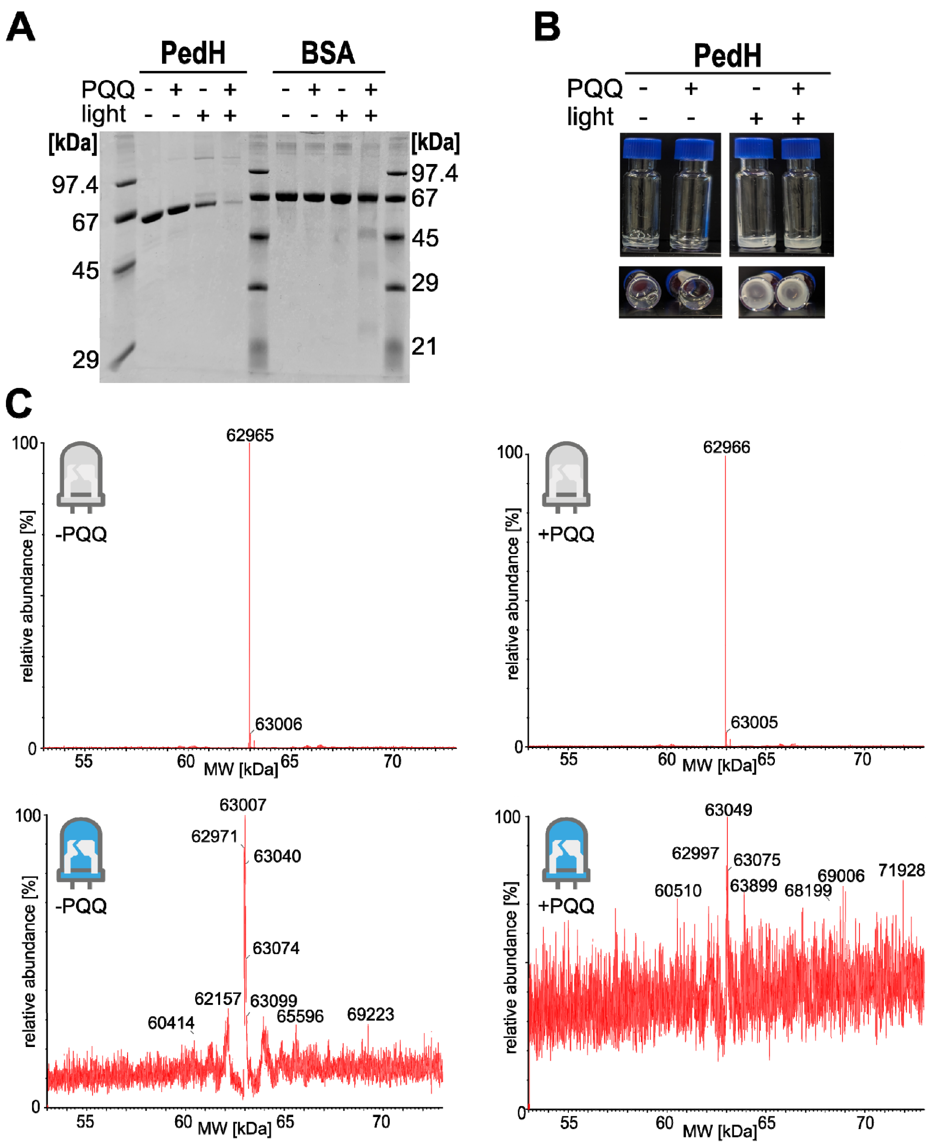

Figure S10. Photostability of PedH. **(A)** SDS-PAGE gel depicting the effect of photodamage on PedH and the control protein BSA. **(B)** Photos of the samples that were kept in the dark (left) or irradiated in the photoreactor (right). **(C)** Mass spectrometry analysis of PedH before (top) or after (bottom) irradiation and in the presence (right) or absence (left) of its natural cofactor PQQ. Expected mass: 62966.2 Da (N-terminal methionine is cleaved)

S5 Lanthanide binding to PLZ variants

The tryptophan-enhanced terbium luminescence readout

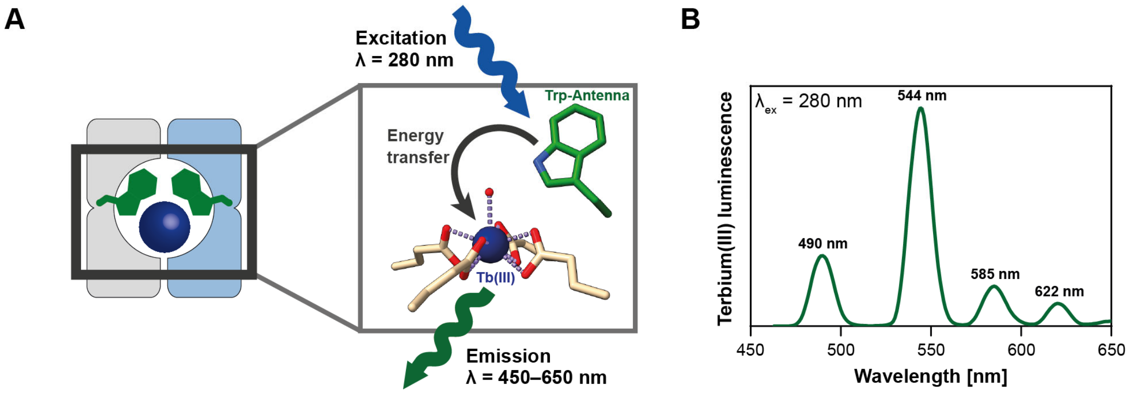

Figure S11. A tryptophan close to the lanthanide binding site serves as an intrinsic antenna to facilitate energy transfer to the bound Tb(III), which enhances its luminescence signal.

Terbium binding kinetics of PLZ1.0, PLZ1.1 and PLZ1.4_W6

For acquisition of the time- and temperature-dependent terbium luminescence, aliquots of the protein of interest and a $TbCl_3$ stock in water were thawed. All solutions were prepared and incubated at the corresponding temperature for the measurement (4 °C, 21 °C, 30 °C, 40 °C). Protein and $TbCl_3$ were diluted in buffer (25 mM HEPES, 100 mM NaCl, pH 8.5) to reach a final concentration of 10 µM each (PLZ1.0: dimer concentration; PLZ1.1 and PLZ1.4_W6: single-chain concentration). The terbium luminescence was measured in a microplate reader (Varioskan LUX, *Thermo Fisher Scientific*) in black 96-well microtiter plates over the course of 6 hours in time-resolved fluorescence mode (TRF; 50 µs delay time, 1 ms integration time, 100 ms measurement time) using 100 µL of the tempered metalloenzyme solution (λ_{ex} = 280 nm, λ_{em} = 544 nm; **Figure S12**).

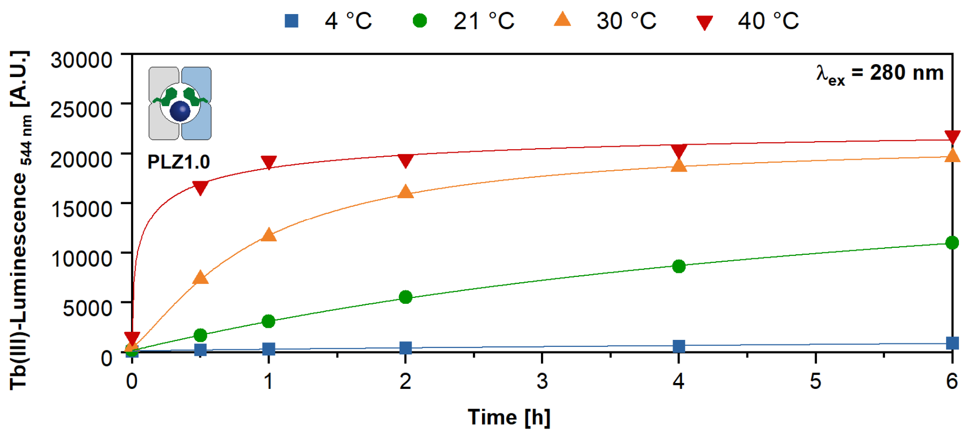

Figure S12. Terbium binding kinetics at different temperatures for PLZ1.0 (10 µM protein scaffold + 10 µM $TbCl_3$); λ_{ex} = 280 nm, λ_{em} = 544 nm).

S6 Protein surface engineering

S6.1 Choice of mutations

Metal binding sites on the protein surface were predicted using *BioMetAll* v1.0 (a software tool by Sánchez-Aparicio *et al*., 2021)[11] and the crystal structure of PLZ1.0 (PDB ID: 6ZV9). The default parameters were used, where *BioMetAll* takes pdb coordinates as an input file and outputs all possible metal binding sites that can be formed by three or four coordinating residues. The identity of the metal was not specified. All coordination sites found consisted of glutamate and aspartate clusters. To reduce unspecific lanthanide binding on the surface of the protein, eight glutamates and aspartates were rationally mutated to glutamine and asparagine, respectively (D9N, E113Q, D132N, D156N, D178N, E282Q, D301N, D325N; positions are highlighted in **Figure S13**). The *BioMetAll* prediction of the surface-engineered protein did not return any metal-binding centers on the surface.

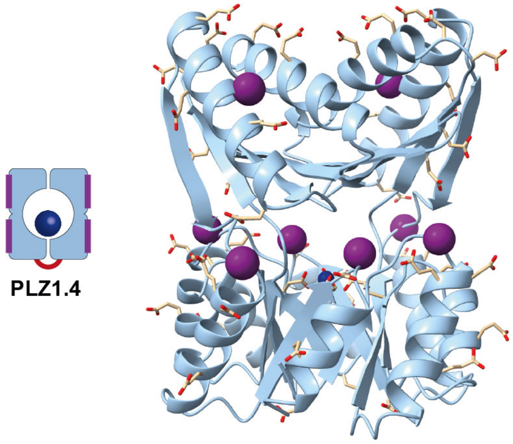

Figure S13. Surface modifications in PLZ1.4 (D9N, E113Q, D132N, D156N, D178N, E282Q, D301N, D325N) mapped as purple spheres on an AlphaFold2 structure prediction of PLZ1.4. All glutamate and aspartate surface residues are shown as stick models.

S6.2 Cerium loading quantified by ICP-MS

All buffers used for ICP-MS were treated with Chelex100 resin prior to use. The protein samples were prepared by incubating a 500 µL solution containing 30 µM protein and 30 µM $CeCl_3$ in FPLC buffer (25 mM HEPES, pH 8.5, 100 mM NaCl) for 2.5 h at 40 °C. Free metal was removed by dialyzing the sample in 1 L buffer (dilution 1:2000) for 2 h twice. All samples were diluted to a final concentration of 2.4 µM in 5 mL FPLC buffer. The chelexed FPLC buffer was submitted to determine the background concentration of cerium. ICP-MS was performed on a Nexion 350D from Perkin Elmer in standard mode. Cerium(III) (*VWR*) at a concentration of 10 µg/mL was used as reference standard for calibration. Rhodium was used as an internal standard. All samples were acidified with 5% nitric acid (65%, Suprapur, *Merck*) and further diluted 1:10 prior to the measurement. The measured cerium concentrations were set in relation to the known protein concentration, where 100% cerium loading refers to one cerium ion per dimer (**Figure S14**).

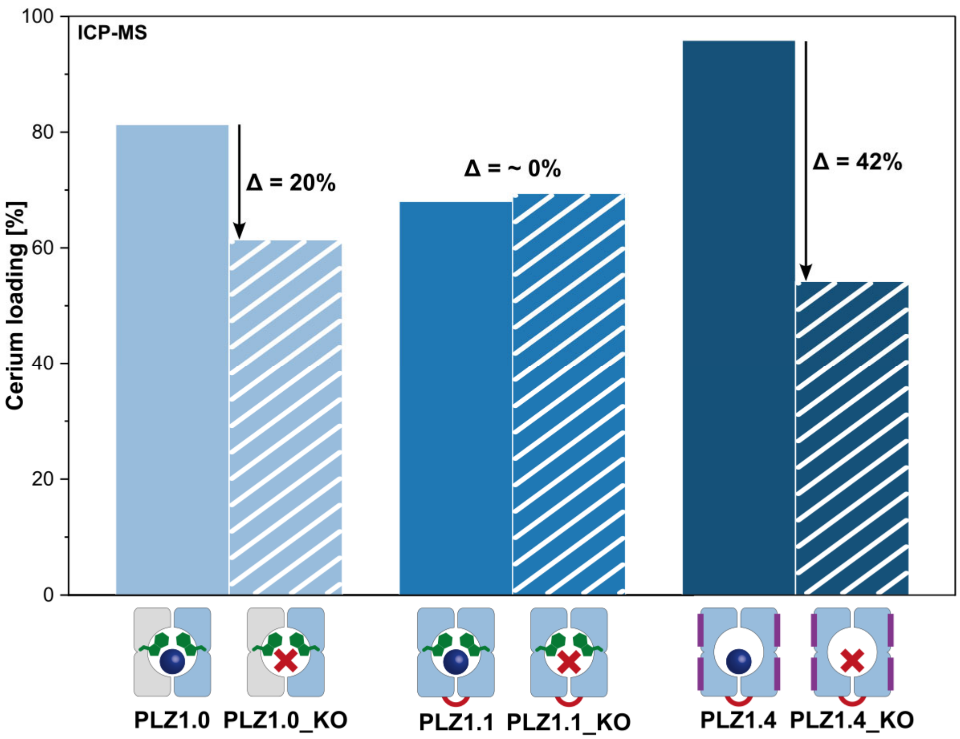

Figure S14. ICP-MS data of PLZ1.0, PLZ1.1, and PLZ1.4, and their respective knock-out variants (in which the glutamates of the lanthanide binding site are mutated to glutamines), quantifying the amount of cerium(III) in the samples after incubation with 1 eq. $CeCl_3$ and a short dialysis. The slightly lower cerium(III) loading of PLZ1.1 compared to PLZ1.0 is in line with the activity measurements in Figure 2D. The difference in cerium(III) loading between WT and KO is highest for the surface-optimized variant PLZ1.4.

S7 Determination of total turnover numbers

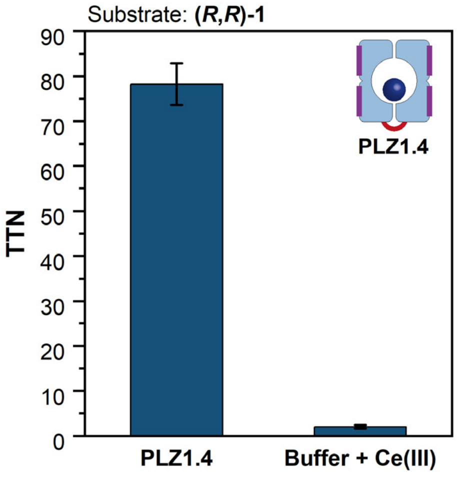

Figure S15. Determination of total turnover numbers (TTN) for PLZ1.4 for substrate ***(R,R)*-1**.

The metalloenzyme was prepared as described in the general procedures for photocatalysis. To determine its total turnover number (TTN), photoreactions were performed with a larger excess of substrate over catalyst and an extended irradiation time.

12 mM ***(R,R)*-1** and 100 µM PLZ1.4 were irradiated for 5 days. To calculate the turnovers per catalyst, product yields were quantified, assuming the formation of two molecules benzaldehyde (**2**) per molecule ***(R,R)*-1**. 100 µM $CeCl_3$ in buffer served as a control and showed significantly less turnovers (TTN = 2.0 ± 0.4) compared to PLZ1.4 (TTN = 78.2 ± 4.7; **Figure S15**).

S8 Substrate scope

S8.1 HPLC traces for all reactions shown in the substrate scope

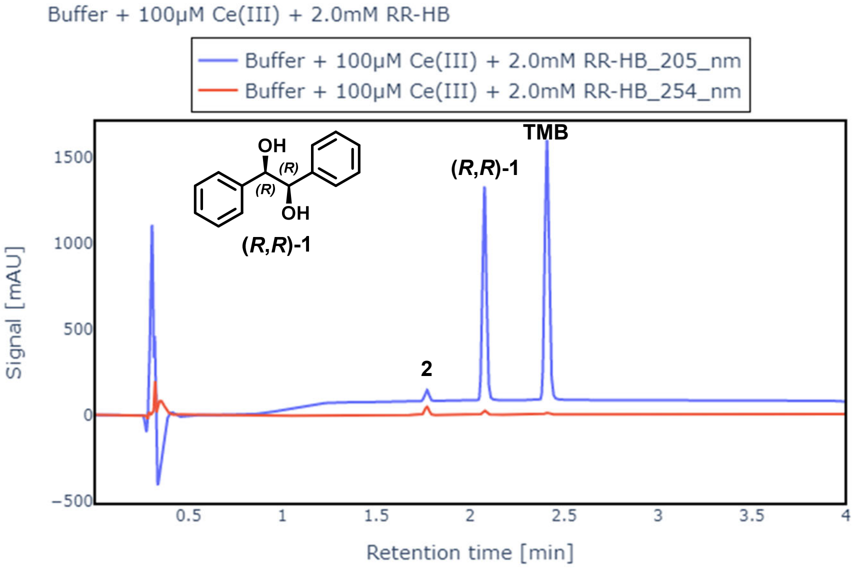

Compound	Retention time [min]	Wavelength [nm]	Area [mAU·min]
Benzaldehyde (**2**)	1.769	254	1.317
(*R*,*R*)-Hydrobenzoin [**(*R*,*R*)-1**]	2.076	205	30.922
1,3,5-Trimethoxybenzene (**TMB**)	2.408	205	41.521

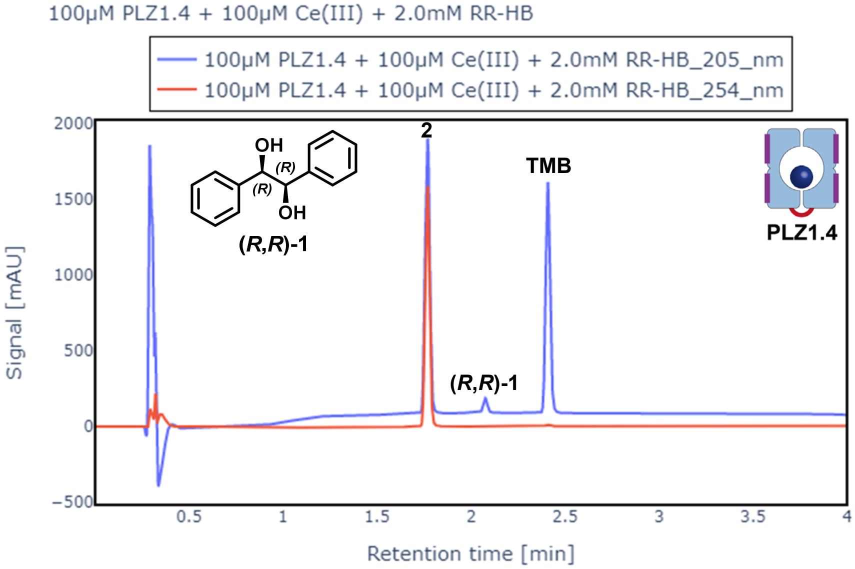

Compound	Retention time [min]	Wavelength [nm]	Area [mAU·min]
Benzaldehyde (**2**)	1.770	254	43.754
(*R*,*R*)-Hydrobenzoin [**(*R*,*R*)-1**]	2.076	205	2.467
1,3,5-Trimethoxybenzene (**TMB**)	2.408	205	41.585

Figure S16. Photocatalytic cleavage of **(*R*,*R*)-1**; HPLC Method 1 (achiral, Hypersil Gold C18).

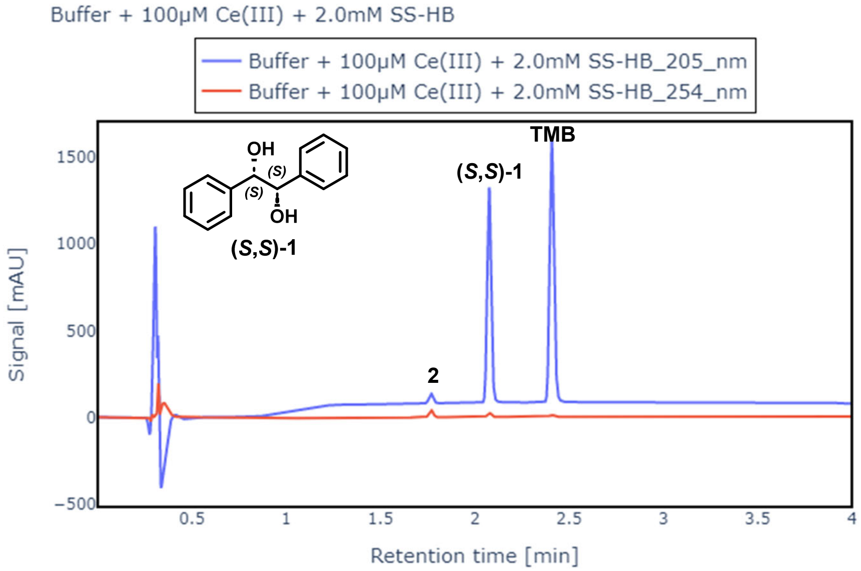

Compound	Retention time [min]	Wavelength [nm]	Area [mAU·min]
Benzaldehyde (**2**)	1.769	254	1.137
(*S*,*S*)-Hydrobenzoin [**(*S*,*S*)-1**]	2.077	205	31.036
1,3,5-Trimethoxybenzene (**TMB**)	2.408	205	41.482

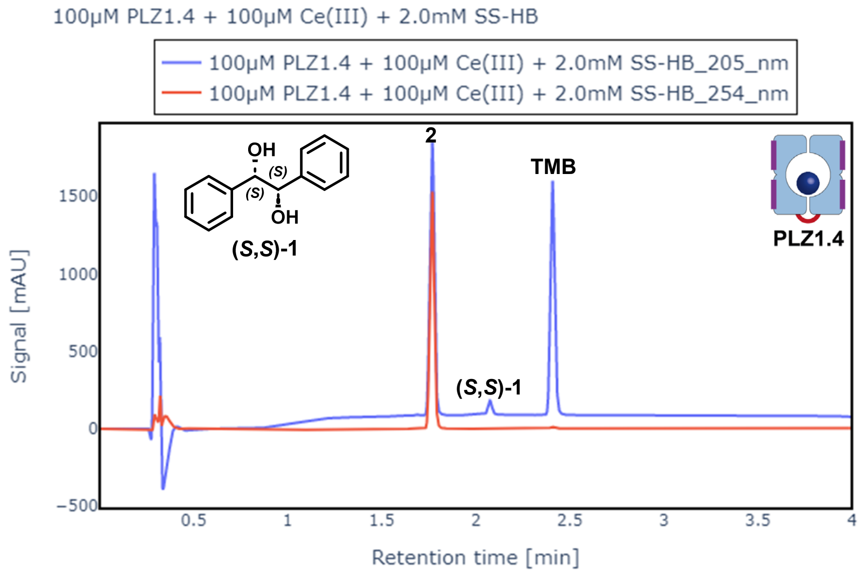

Compound	Retention time [min]	Wavelength [nm]	Area [mAU·min]
Benzaldehyde (**2**)	1.769	254	42.070
(*S*,*S*)-Hydrobenzoin [**(*S*,*S*)-1**]	2.076	205	2.274
1,3,5-Trimethoxybenzene (**TMB**)	2.408	205	41.385

Figure S17. Photocatalytic cleavage of **(*S*,*S*)-1**; HPLC Method 1 (achiral, Hypersil Gold C18).

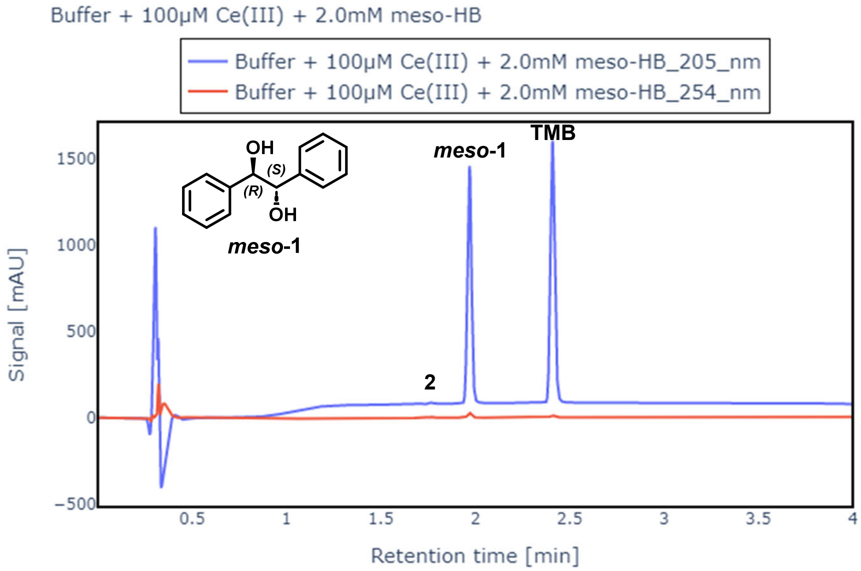

Compound	Retention time [min]	Wavelength [nm]	Area [mAU·min]
Benzaldehyde (**2**)	1.769	254	0.141
meso-Hydrobenzoin (***meso*-1**)	1.970	205	35.273
1,3,5-Trimethoxybenzene (**TMB**)	2.409	205	41.533

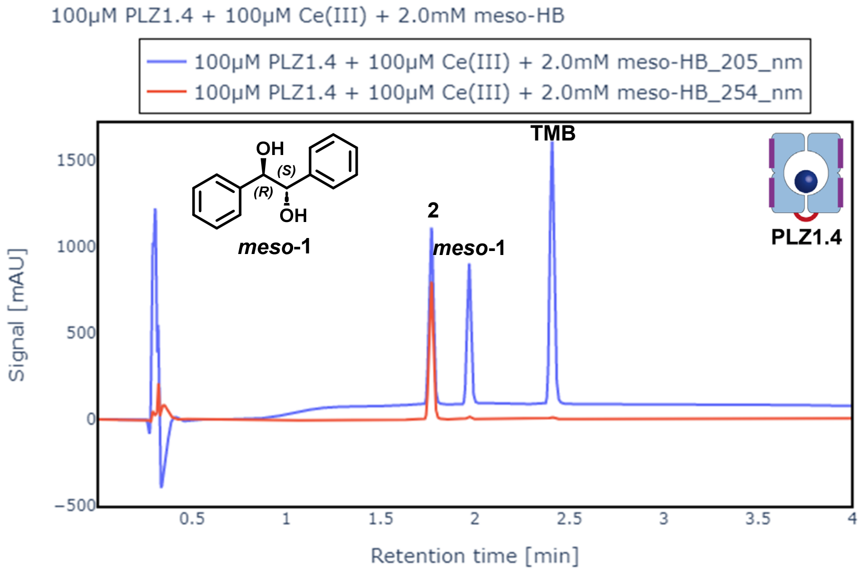

Compound	Retention time [min]	Wavelength [nm]	Area [mAU·min]
Benzaldehyde (**2**)	1.769	254	21.890
meso-Hydrobenzoin (***meso*-1**)	1.969	205	20.703
1,3,5-Trimethoxybenzene (**TMB**)	2.408	205	41.505

Figure S18. Photocatalytic cleavage of ***meso*-1**; HPLC Method 1 (achiral, Hypersil Gold C18).

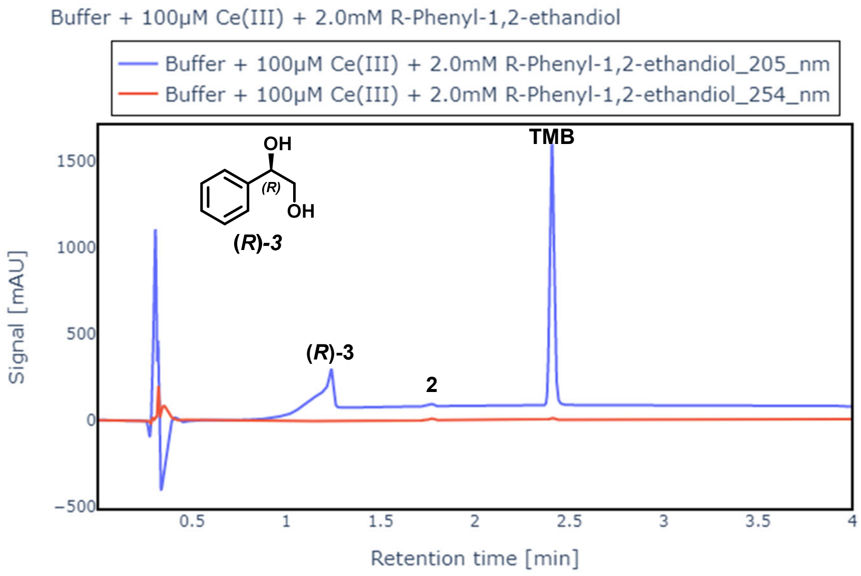

Compound	Retention time [min]	Wavelength [nm]	Area [mAU·min]
(*R*)-Phenyl-1,2-ethandiol [(***R***)-**3**]	1.238	205	19.433
Benzaldehyde (**2**)	1.769	254	0.289
1,3,5-Trimethoxybenzene (**TMB**)	2.408	205	41.449

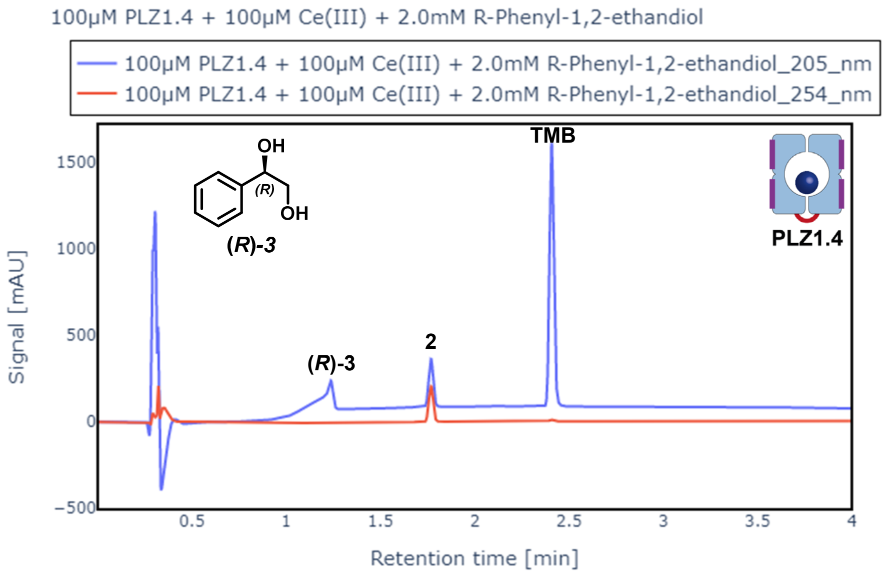

Compound	Retention time [min]	Wavelength [nm]	Area [mAU·min]
(*R*)-Phenyl-1,2-ethandiol [(***R***)-**3**]	1.238	205	14.533
Benzaldehyde (**2**)	1.768	254	5.694
1,3,5-Trimethoxybenzene (**TMB**)	2.408	205	41.457

Figure S19. Photocatalytic cleavage of (***R***)-**3**; HPLC Method 1 (achiral, Hypersil Gold C18).

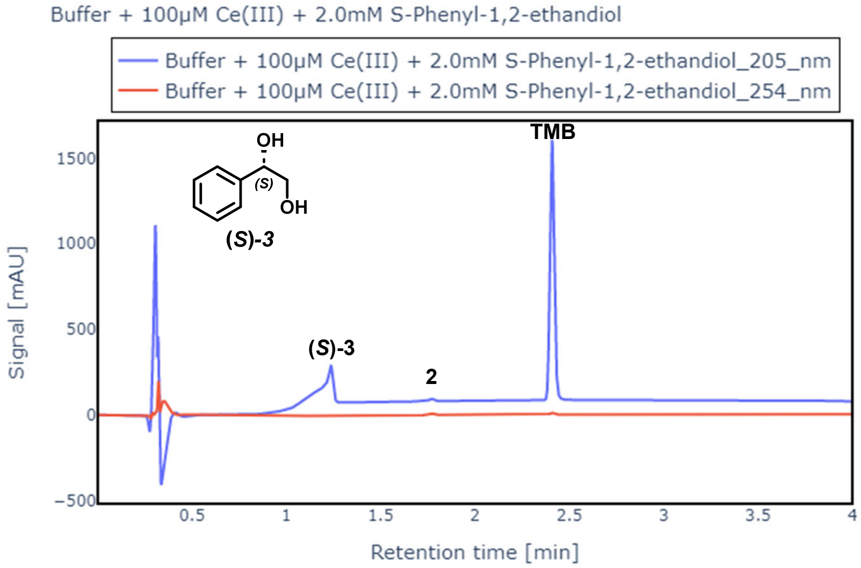

**Compound**	**Retention time [min]**	**Wavelength [nm]**	**Area [mAU·min]**
(*R*)-Phenyl-1,2-ethandiol [**(*S*)-3**]	1.237	205	18.441
Benzaldehyde (**2**)	1.768	254	0.287
1,3,5-Trimethoxybenzene (**TMB**)	2.408	205	41.467

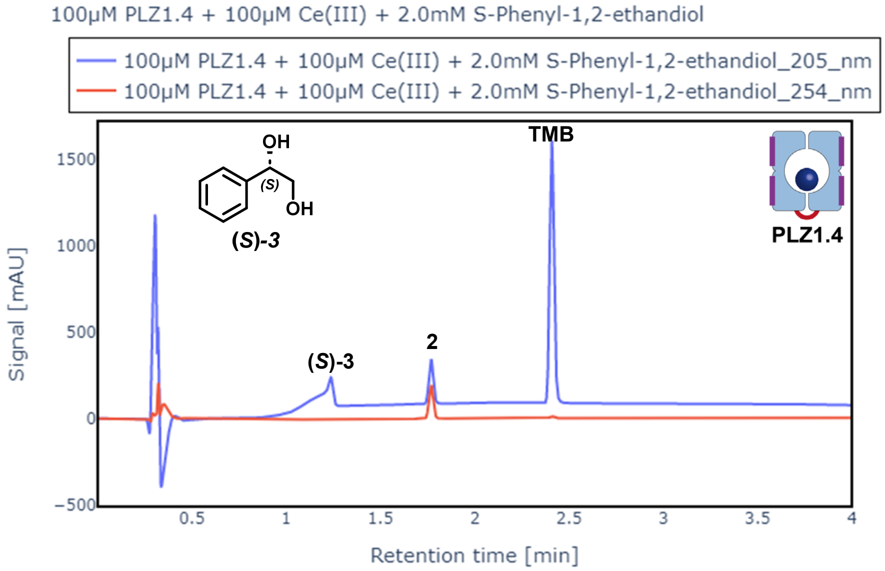

**Compound**	**Retention time [min]**	**Wavelength [nm]**	**Area [mAU·min]**
(*R*)-Phenyl-1,2-ethandiol [**(*S*)-3**]	1.238	205	14.587
Benzaldehyde (**2**)	1.769	254	5.266
1,3,5-Trimethoxybenzene (**TMB**)	2.409	205	41.388

Figure S20. Photocatalytic cleavage of **(*S*)-3**; HPLC Method 1 (achiral, Hypersil Gold C18).

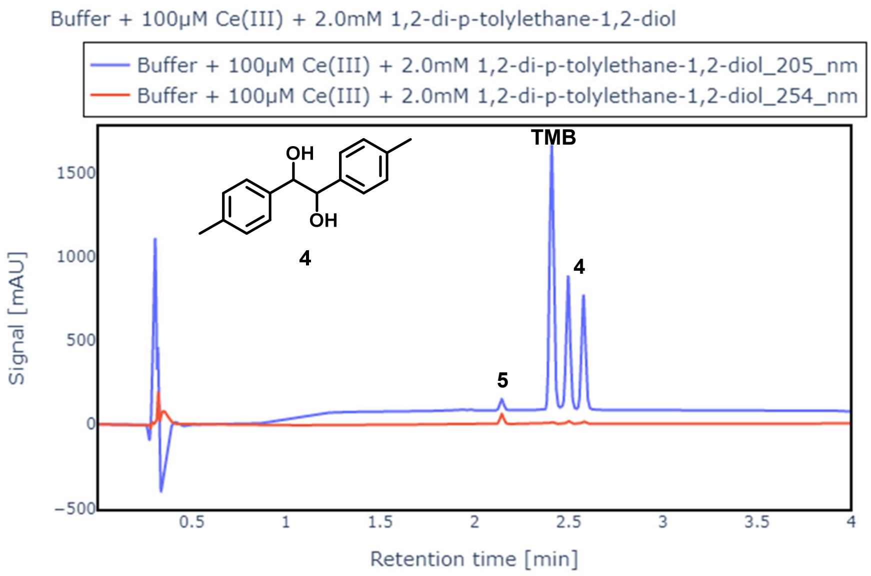

Compound	Retention time [min]	Wavelength [nm]	Area [mAU·min]
4-Methylbenzaldehyde (**5**)	2.145	254	1.453
1,3,5-Trimethoxybenzene (**TMB**)	2.410	205	41.183
1,2-di-p-tolylethane-1,2-diol (**4**)	2.498+2.578	205	38.326

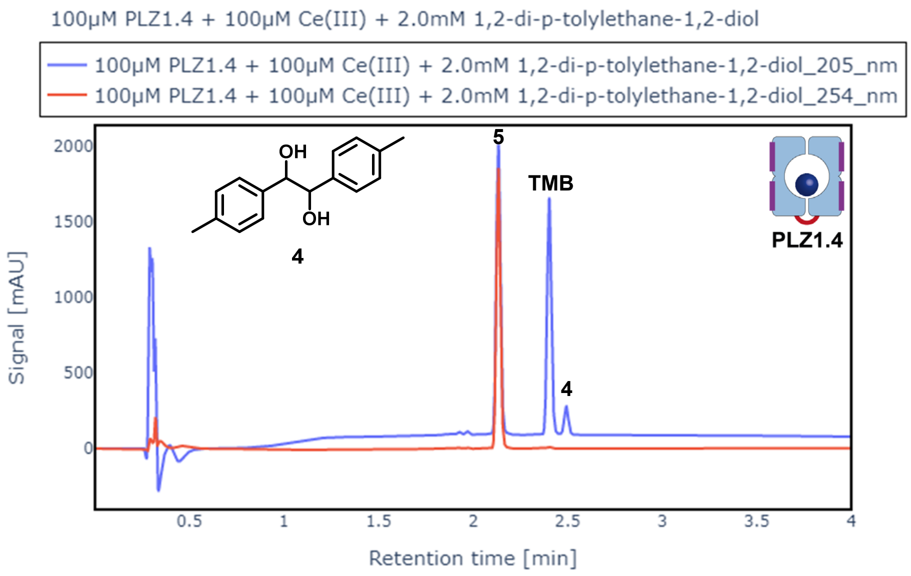

Compound	Retention time [min]	Wavelength [nm]	Area [mAU·min]
4-Methylbenzaldehyde (**5**)	2.134	254	46.688
1,3,5-Trimethoxybenzene (**TMB**)	2.403	205	41.457
1,2-di-p-tolylethane-1,2-diol (**4**)	2.493	205	5.053

Figure S21. Photocatalytic cleavage of **4**; HPLC Method 1 (achiral, Hypersil Gold C18).

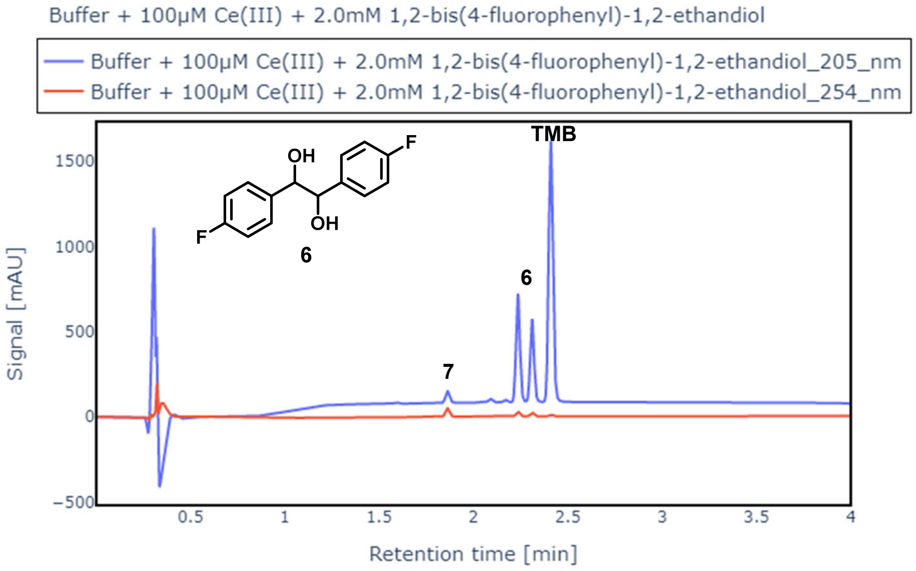

Compound	Retention time [min]	Wavelength [nm]	Area [mAU·min]
4-Flourobenzaldehyde (**7**)	1.863	254	1.305
1,2-bis(4-Fluorophenyl)-1,2-ethandiol (**6**)	2.236+2.311	205	27.483
1,3,5-Trimethoxybenzene (**TMB**)	2.410	205	41.168

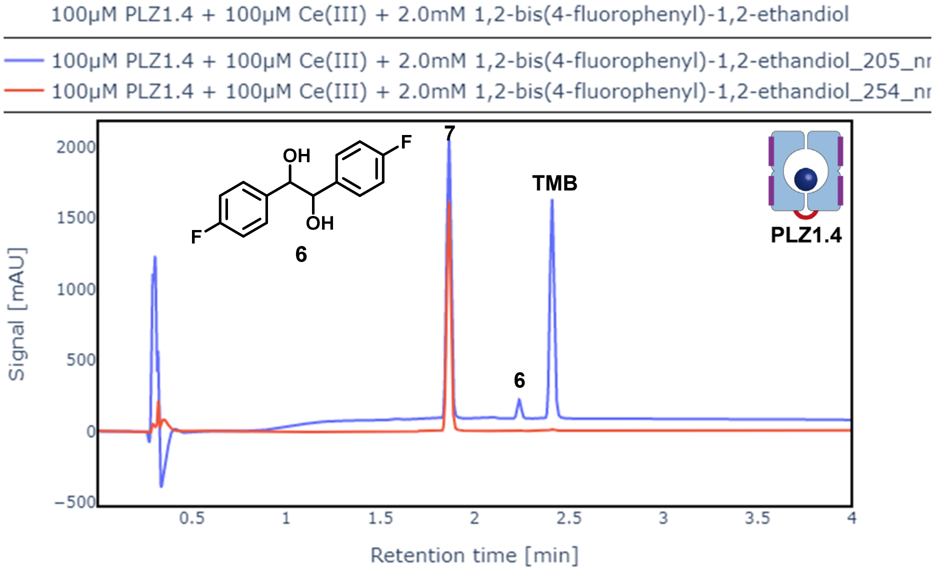

Compound	Retention time [min]	Wavelength [nm]	Area [mAU·min]
4-Flourobenzaldehyde (**7**)	1.863	254	42.604
1,2-bis(4-Fluorophenyl)-1,2-ethandiol (**6**)	2.236	205	3.239
1,3,5-Trimethoxybenzene (**TMB**)	2.410	205	41.521

Figure S22. Photocatalytic cleavage of **6**; HPLC Method 1 (achiral, Hypersil Gold C18).

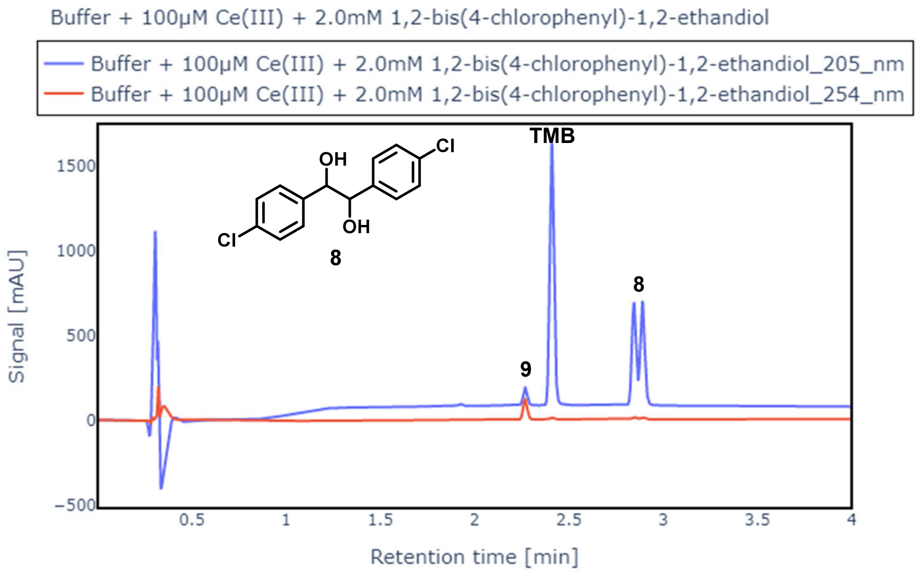

Compound	Retention time [min]	Wavelength [nm]	Area [mAU·min]
4-Chlorobenzaldehyde (**9**)	2.268	254	3.054
1,3,5-Trimethoxybenzene (**TMB**)	2.409	205	41.491
1,2-bis(4-Chlorophenyl)-1,2-ethandiol (**8**)	2.845+2.890	205	33.363

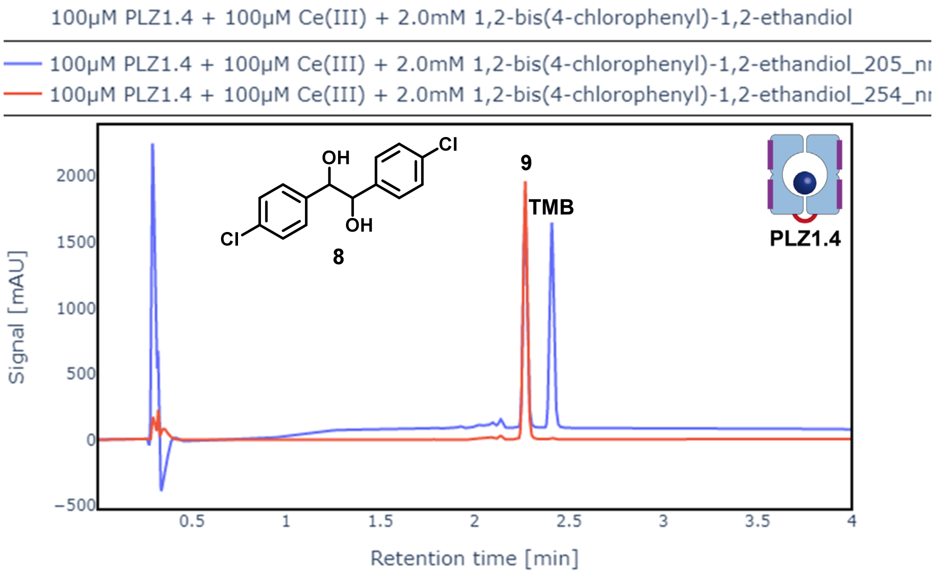

Compound	Retention time [min]	Wavelength [nm]	Area [mAU·min]
4-Chlorobenzaldehyde (**9**)	2.268	254	50.317
1,3,5-Trimethoxybenzene (**TMB**)	2.409	205	41.465
1,2-bis(4-Chlorophenyl)-1,2-ethandiol (**8**)	2.845+2.890	205	0.0

Figure S23. Photocatalytic cleavage of **8**; HPLC Method 1 (achiral, Hypersil Gold C18).

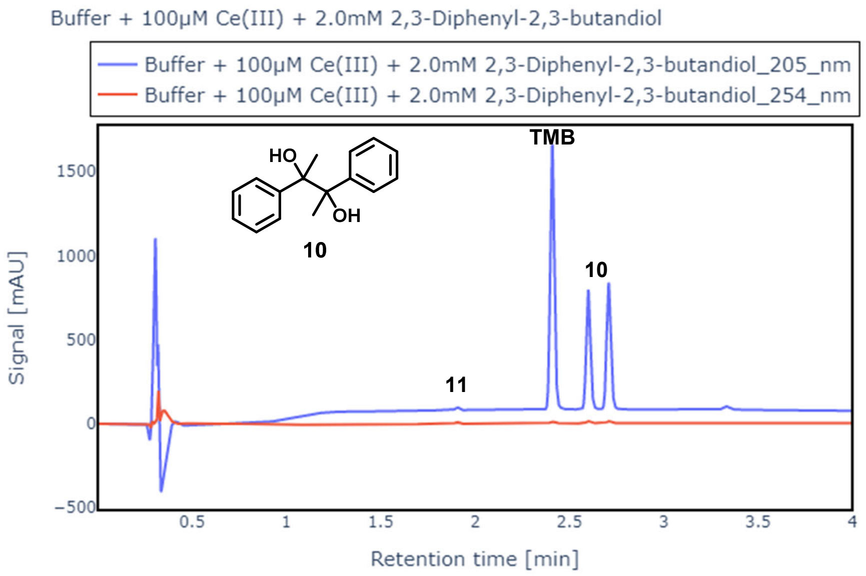

Compound	Retention time [min]	Wavelength [nm]	Area [mAU·min]
Acetophenone (**11**)	1.906	254	0.245
1,3,5-Trimethoxybenzene (**TMB**)	2.409	205	41.197
2,3-Diphenyl-2,3-butandiol (**10**)	2.600+2.708	205	38.821

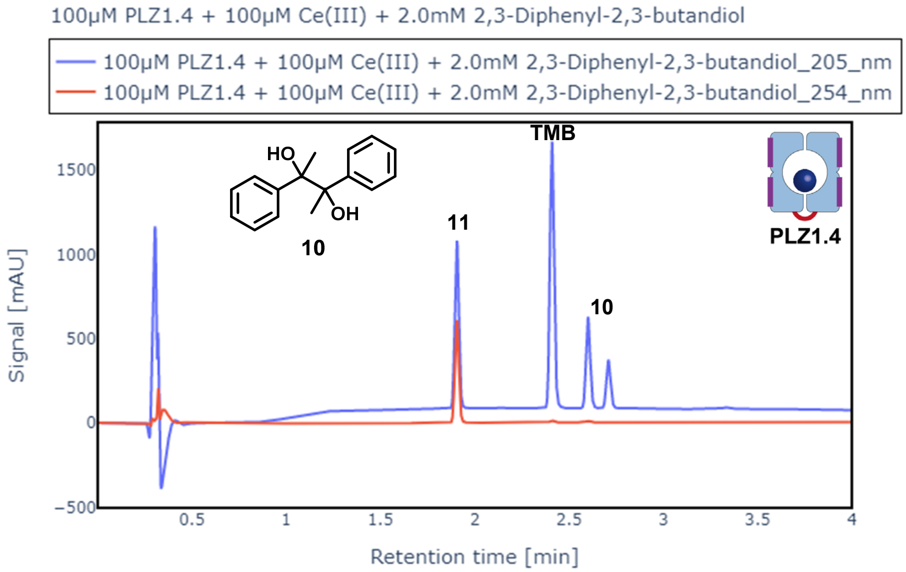

Compound	Retention time [min]	Wavelength [nm]	Area [mAU·min]
Acetophenone (**11**)	1.906	254	15.314
1,3,5-Trimethoxybenzene (**TMB**)	2.410	205	41.471
2,3-Diphenyl-2,3-butandiol (**10**)	2.601+2.708	205	21.856

Figure S24. Photocatalytic cleavage of **10**; HPLC Method 1 (achiral, Hypersil Gold C18).

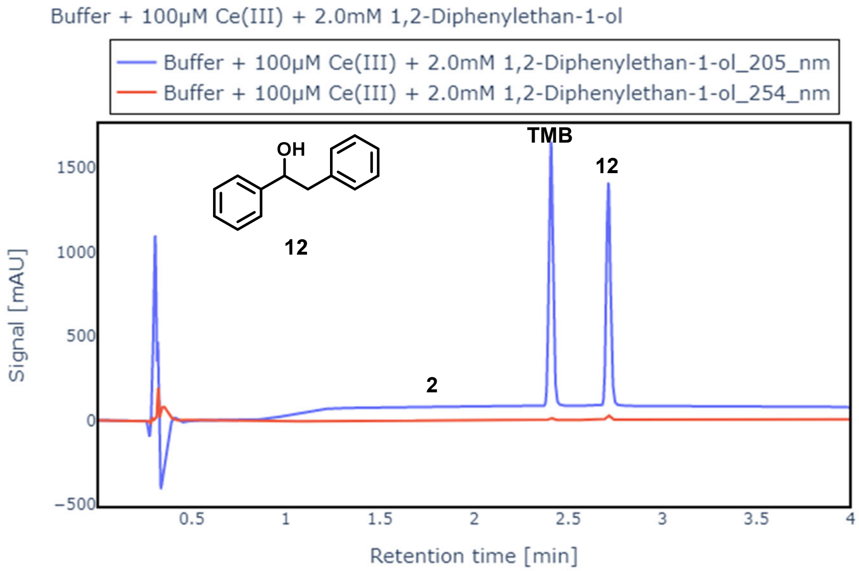

Compound	Retention time [min]	Wavelength [nm]	Area [mAU·min]
Benzaldehyde (**2**)	1.770	254	0.017
1,3,5-Trimethoxybenzene (**TMB**)	2.408	205	40.904
1,2-Diphenylethan-1-ol (**12**)	2.714	205	34.750

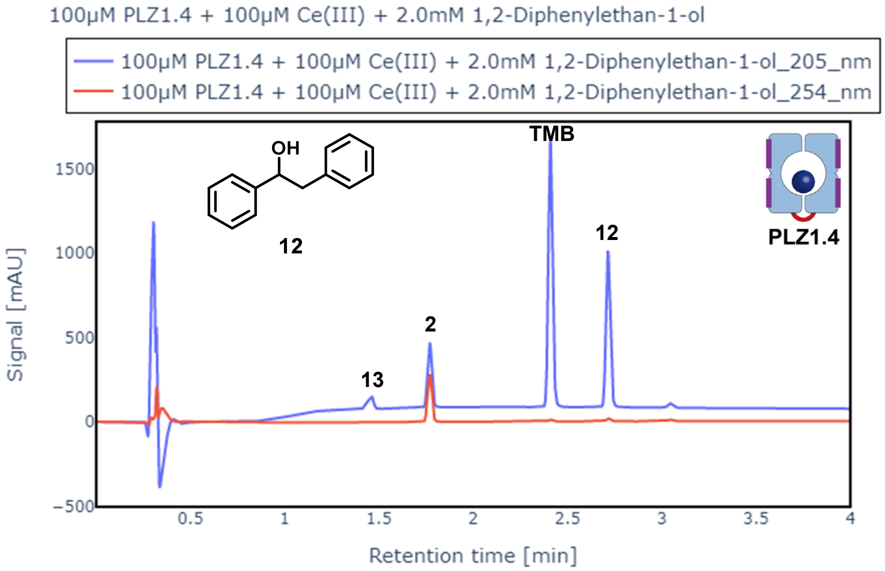

Compound	Retention time [min]	Wavelength [nm]	Area [mAU·min]
Benzyl alcohol (**13**)	1.462	205	2.869
Benzaldehyde (**2**)	1.770	254	7.640
1,3,5-Trimethoxybenzene (**TMB**)	2.409	205	41.195
1,2-Diphenylethan-1-ol (**12**)	2.714	205	24.157

Figure S25. Photocatalytic cleavage of **12**; HPLC Method 1 (achiral, Hypersil Gold C18).

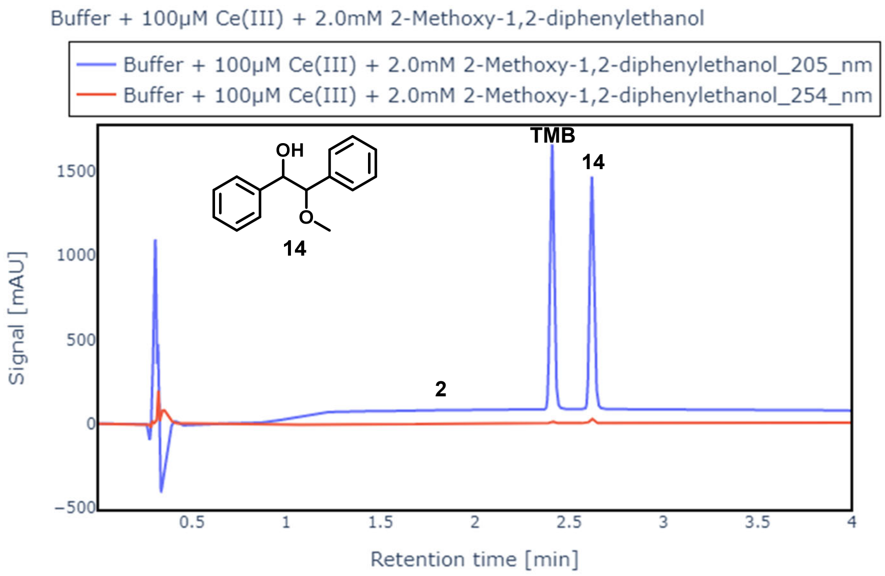

Compound	Retention time [min]	Wavelength [nm]	Area [mAU·min]
Benzaldehyde (**2**)	1.772	254	0.051
1,3,5-Trimethoxybenzene (**TMB**)	2.410	205	41.068
2-Methoxy-1,2-diphenylethanol (**14**)	2.621	205	37.274

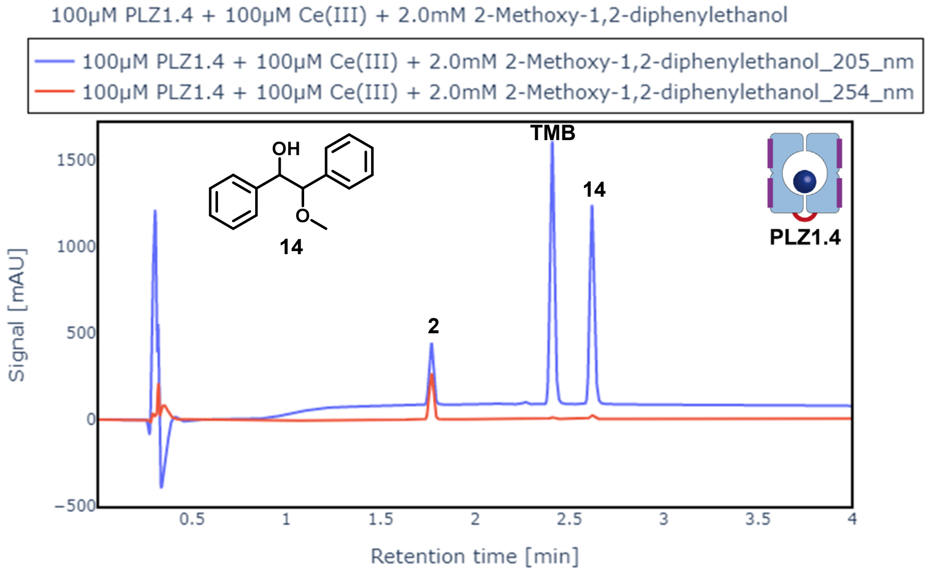

Compound	Retention time [min]	Wavelength [nm]	Area [mAU·min]
Benzaldehyde (**2**)	1.769	254	7.351
1,3,5-Trimethoxybenzene (**TMB**)	2.409	205	41.647
2-Methoxy-1,2-diphenylethanol (**14**)	2.620	205	32.336

Figure S26. Photocatalytic cleavage of **14**; HPLC Method 1 (achiral, Hypersil Gold C18).

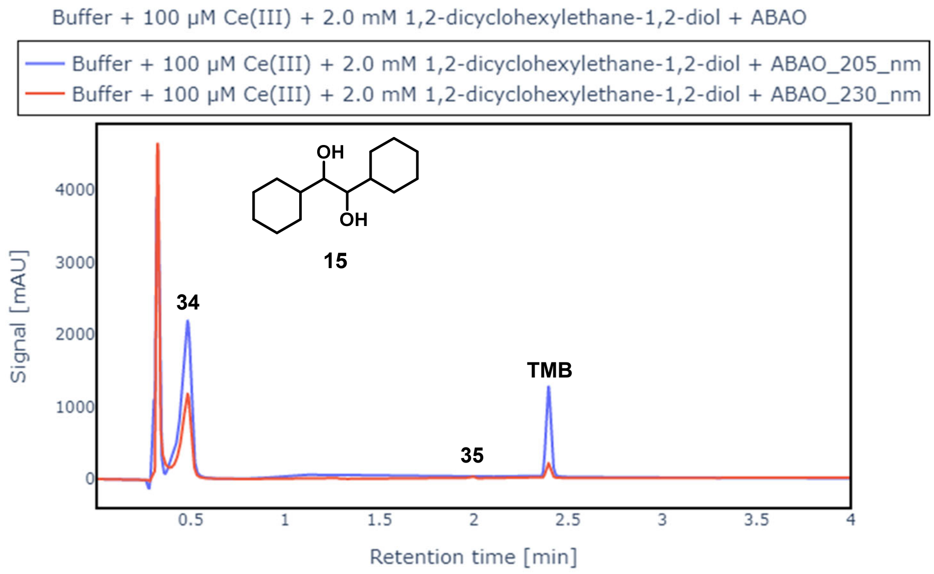

Compound	Retention time [min]	Wavelength [nm]	Area [mAU·min]
Unreacted ABAO (**34**)	0.483	205	133.841
Cyclohecancarbaldehyde-ABAO (**35**)	1.993	230	0.881
1,3,5-Trimethoxybenzene (**TMB**)	2.397	205	39.520

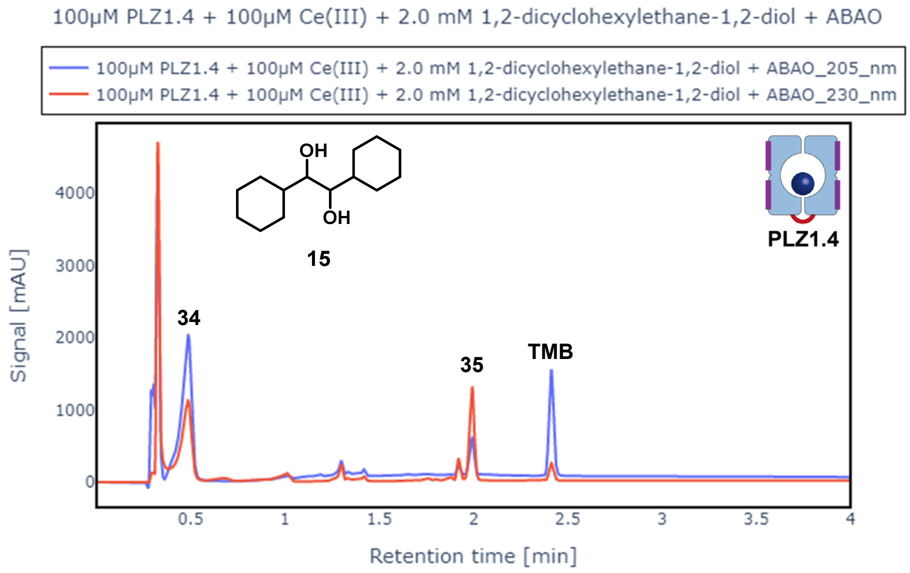

Compound	Retention time [min]	Wavelength [nm]	Area [mAU·min]
Unreacted ABAO (**34**)	0.487	205	127.158
Cyclohecancarbaldehyde-ABAO (**35**)	1.993	230	36.376
1,3,5-Trimethoxybenzene (**TMB**)	2.412	205	43.273

Figure S27. Photocatalytic cleavage of **15**; HPLC Method 1 (achiral, Hypersil Gold C18).

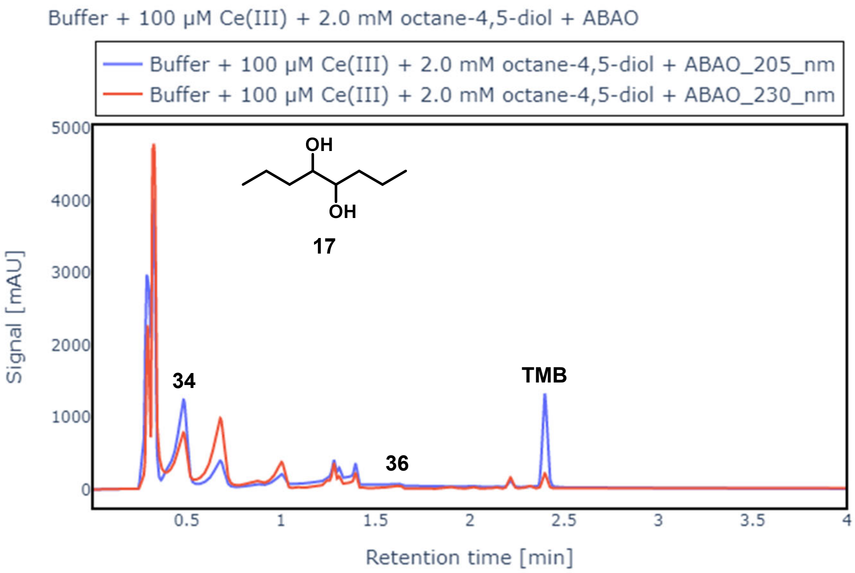

Compound	Retention time [min]	Wavelength [nm]	Area [mAU·min]
Unreacted ABAO (**34**)	0.483	205	75.480
Butyraldehyde-ABAO (**36**)	1.632	230	1.368
1,3,5-Trimethoxybenzene (**TMB**)	2.398	205	39.893

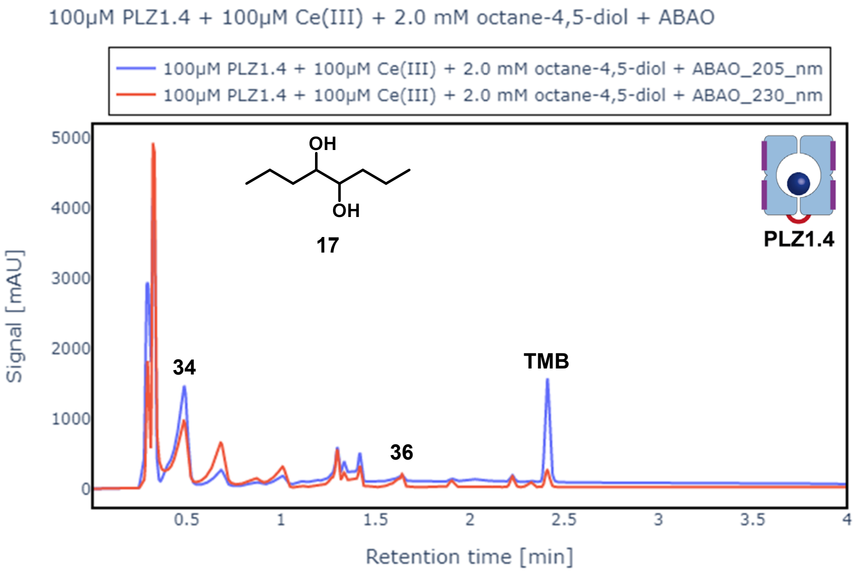

Compound	Retention time [min]	Wavelength [nm]	Area [mAU·min]
Unreacted ABAO (**34**)	0.487	205	94.303
Butyraldehyde-ABAO (**36**)	1.642	230	8.482
1,3,5-Trimethoxybenzene (**TMB**)	2.413	205	43.177

Figure S28. Photocatalytic cleavage of **17**; HPLC Method 1 (achiral, Hypersil Gold C18).

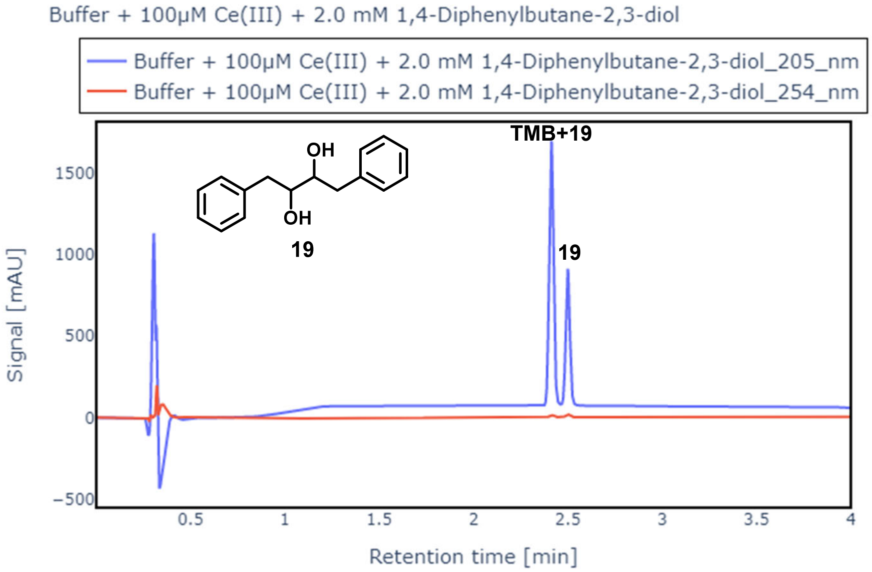

Compound	Retention time [min]	Wavelength [nm]	Area [mAU·min]
1,3,5-Trimethoxybenzene (**TMB**) + **19**	2.413	205	44.312
1,4-Diphenylbutane-2,3-diol (**19**)	2.500	205	21.743

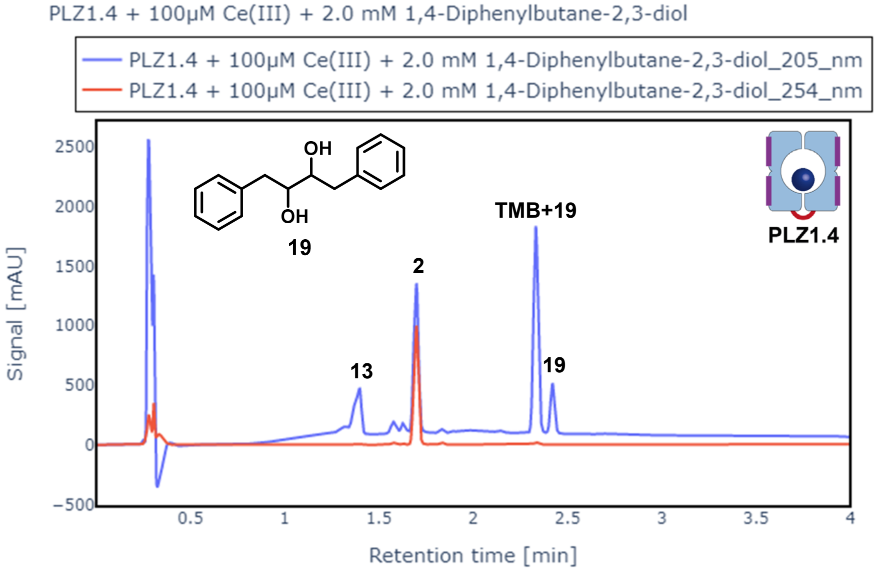

Compound	Retention time [min]	Wavelength [nm]	Area [mAU·min]
Benzyl alcohol (**13**)	1.397	205	17.419
Benzaldehyde (**2**)	1.700	254	28.863
1,3,5-Trimethoxybenzene (**TMB**) + **19**	2.332	205	50.022
1,4-Diphenylbutane-2,3-diol (**19**)	2.421	205	12.528

Figure S29. Photocatalytic cleavage of **19**; HPLC Method 1 (achiral, Hypersil Gold C18).

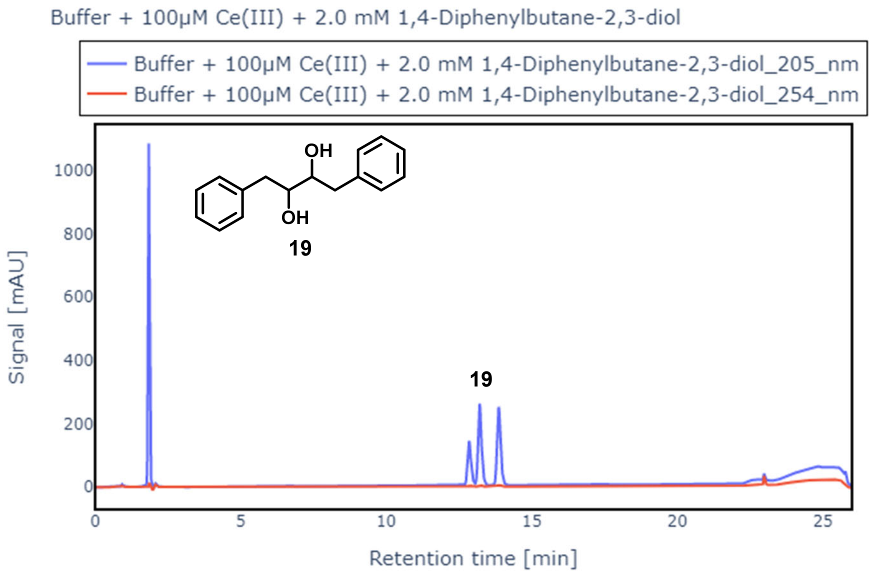

Compound	Retention time [min]	Wavelength [nm]	Area [mAU·min]
1,4-Diphenylbutane-2,3-diol [**19**: *meso*,(*S*,*S*),(*R*,*R*)]	12.848, 13.210, 13.863	205	19.471, 35.558, 35.345

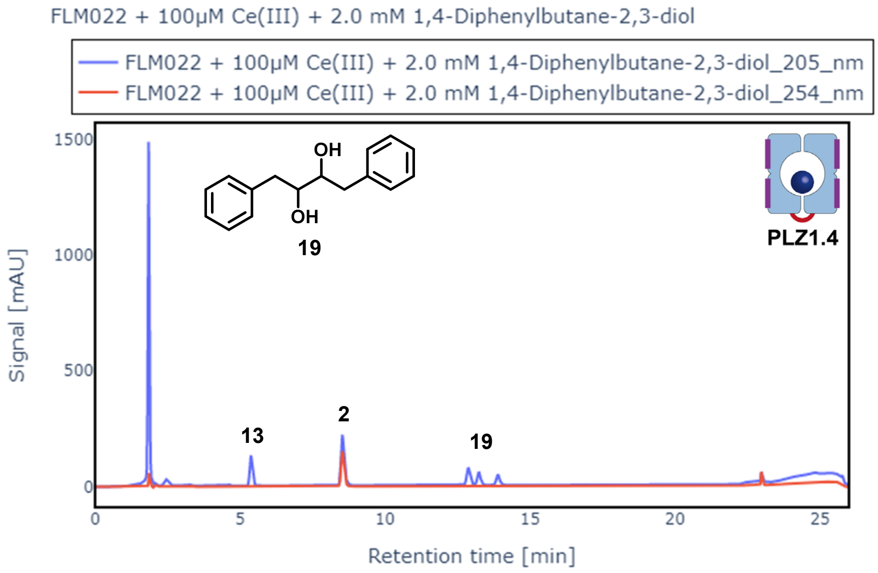

Compound	Retention time [min]	Wavelength [nm]	Area [mAU·min]
Benzyl alcohol (**13**)	5.376	205	14.414
Benzaldehyde (**2**)	8.528	254	18.260
1,4-Diphenylbutane-2,3-diol [**19**: *meso*,(*S*,*S*),(*R*,*R*)]	12.848, 13.210, 13.863	205	10.832, 8.070, 6.693

Figure S30. Photocatalytic cleavage of **19**; HPLC Method 2 (chiral, Chiralcel OJ-RH).

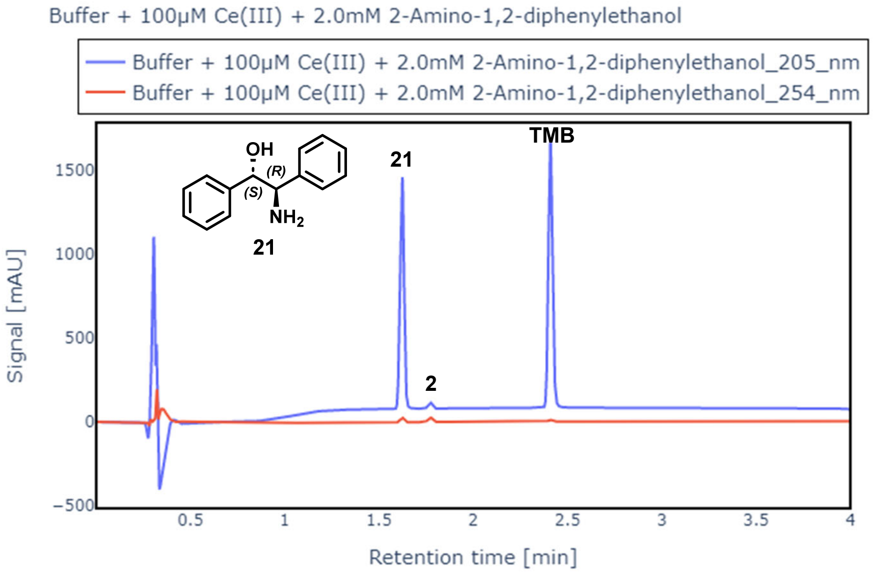

Compound	Retention time [min]	Wavelength [nm]	Area [mAU·min]
(1*S*,2*R*)-2-Amino-1,2-diphenylethan-1-ol (**21**)	1.624	205	34.215
Benzaldehyde (**2**)	1.773	254	0.734
1,3,5-Trimethoxybenzene (**TMB**)	2.409	205	41.380

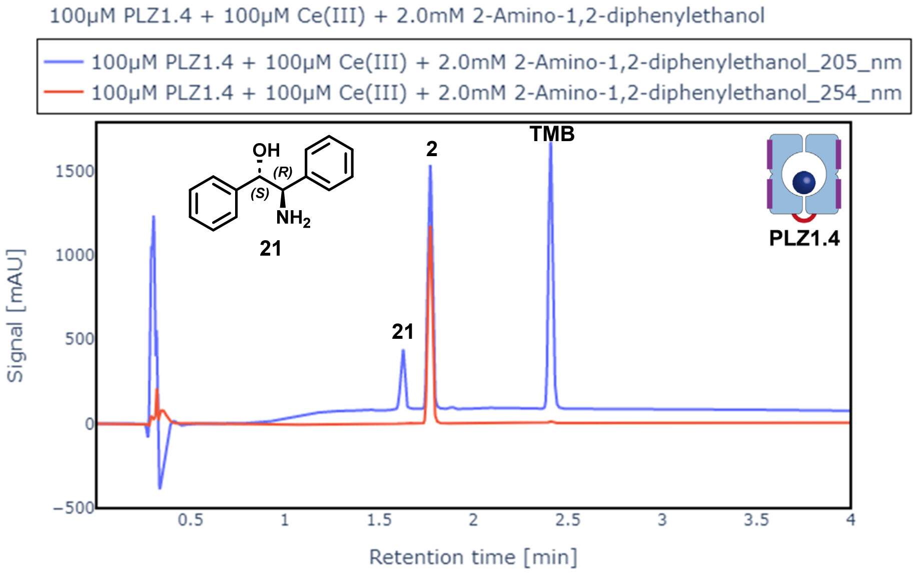

Compound	Retention time [min]	Wavelength [nm]	Area [mAU·min]
(1*S*,2*R*)-2-Amino-1,2-diphenylethan-1-ol (**21**)	1.628	205	9.281
Benzaldehyde (**2**)	1.771	254	31.800
1,3,5-Trimethoxybenzene (**TMB**)	2.409	205	41.426

Figure S31. Photocatalytic cleavage of **21**; HPLC Method 1 (achiral, Hypersil Gold C18).

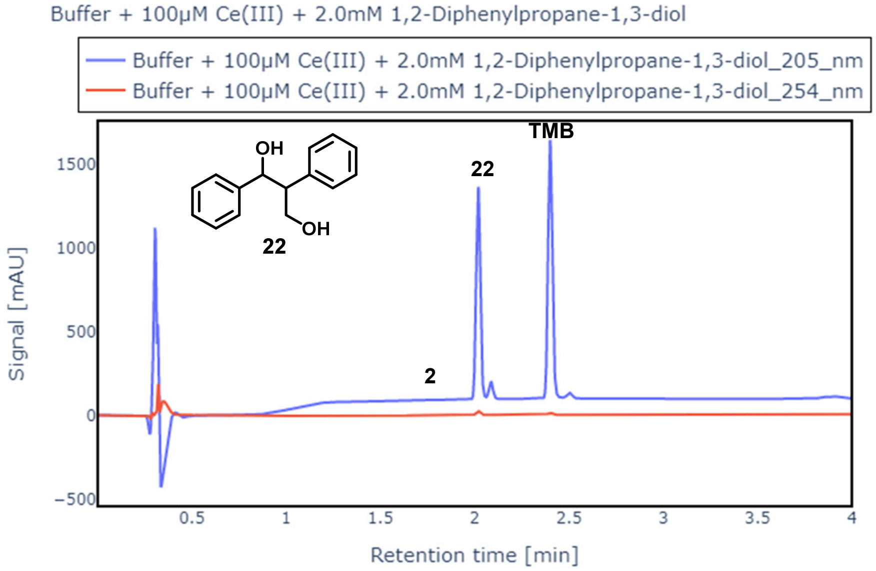

Compound	Retention time [min]	Wavelength [nm]	Area [mAU·min]
Benzaldehyde (**2**)	1.760	254	0.029
1,2-Diphenylpropane-1,3-diol (**22**)	2.019+2.086	205	32.688
1,3,5-Trimethoxybenzene (**TMB**)	2.400	205	41.553

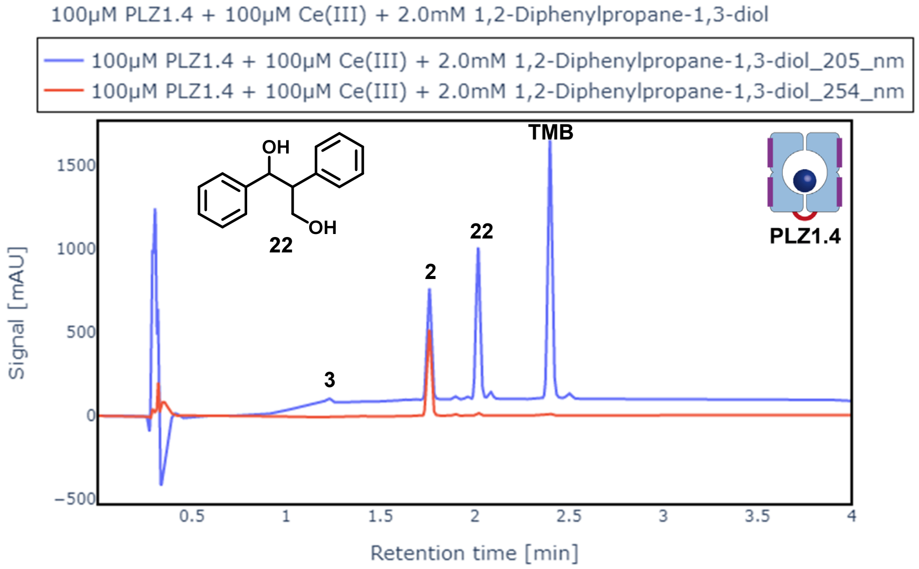

Compound	Retention time [min]	Wavelength [nm]	Area [mAU·min]
Phenyl-1,2-ethandiol (**3**)	1.231	205	1.354
Benzaldehyde (**2**)	1.760	254	14.048
1,2-Diphenylpropane-1,3-diol (**22**)	2.018+2.086	205	23.336
1,3,5-Trimethoxybenzene (**TMB**)	2.399	205	41.958

Figure S32. Photocatalytic cleavage of **22**; HPLC Method 1 (achiral, Hypersil Gold C18).

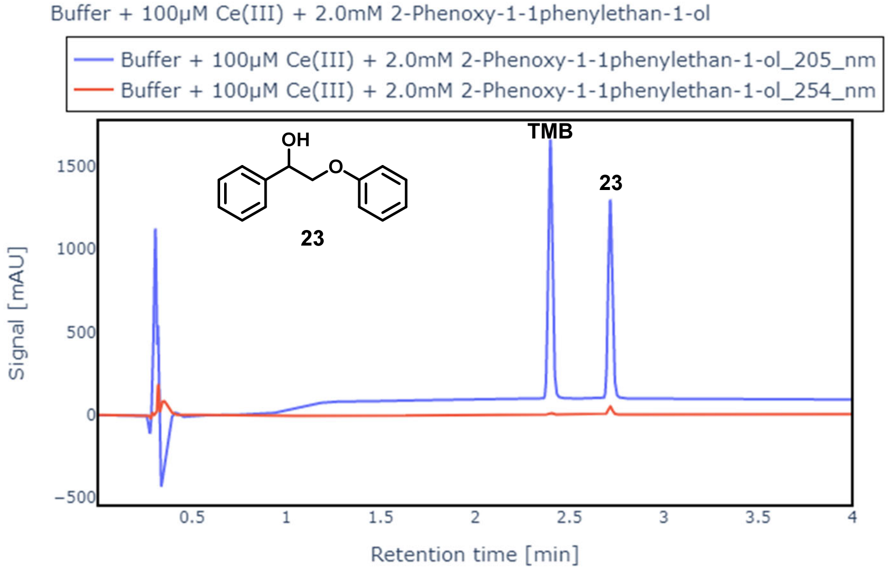

Compound	Retention time [min]	Wavelength [nm]	Area [mAU·min]
1,3,5-Trimethoxybenzene (**TMB**)	1.399	205	41.464
2-Phenoxy-1-1phenylethan-1-ol (**23**)	2.717	205	31.748

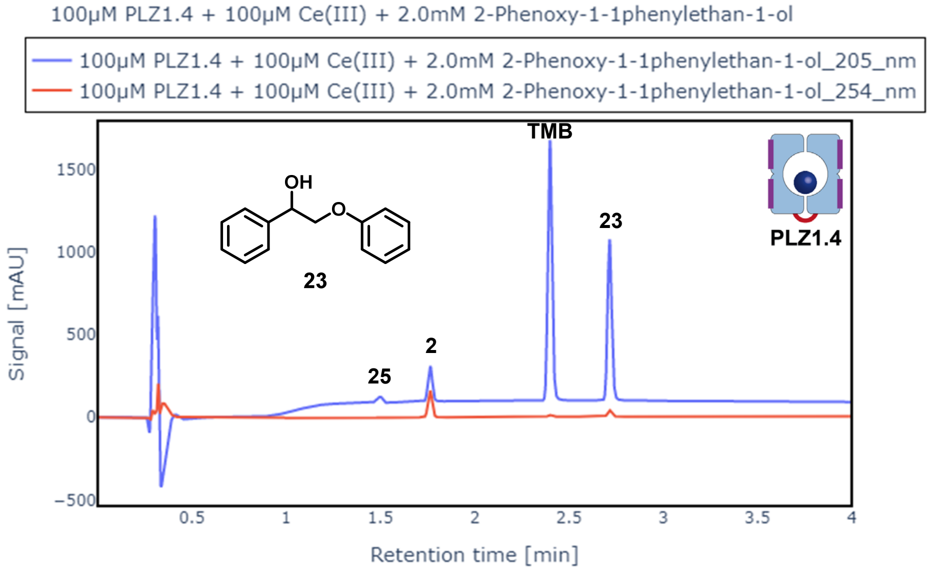

Compound	Retention time [min]	Wavelength [nm]	Area [mAU·min]
Phenol (**25**)	1.500	205	1.356
Benzaldehyde (**2**)	1.765	254	4.307
1,3,5-Trimethoxybenzene (**TMB**)	2.399	205	41.501
2-Phenoxy-1-1phenylethan-1-ol (**23**)	2.716	205	25.601

Figure S33. Photocatalytic cleavage of **23**; HPLC Method 1 (achiral, Hypersil Gold C18).

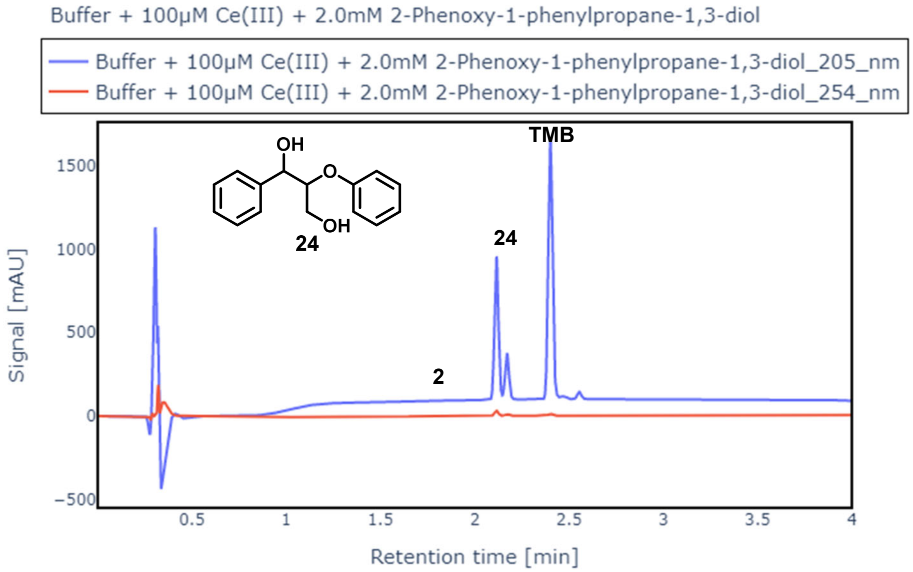

Compound	Retention time [min]	Wavelength [nm]	Area [mAU·min]
Benzaldehyde (**2**)	1.758	254	0.051
2-Phenoxy-1-phenylpropane-1,3-diol (**24**)	2.116+2.172	205	28.075
1,3,5-Trimethoxybenzene (**TMB**)	2.401	205	41.844

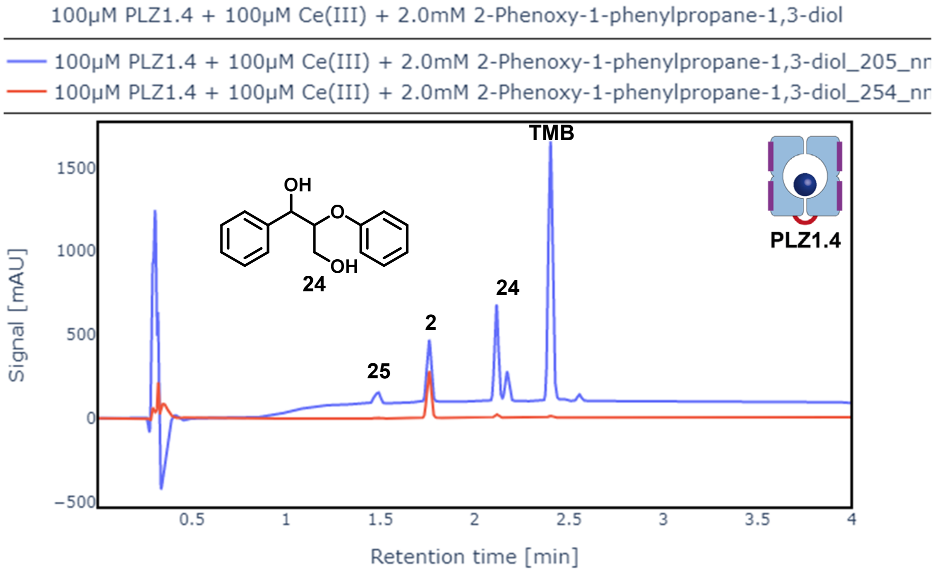

Compound	Retention time [min]	Wavelength [nm]	Area [mAU·min]
Phenol (**25**)	1.488	205	2.556
Benzaldehyde (**2**)	1.758	254	7.765
2-Phenoxy-1-phenylpropane-1,3-diol (**24**)	2.116+2.172	205	18.561
1,3,5-Trimethoxybenzene (**TMB**)	2.403	205	41.801

Figure S34. Photocatalytic cleavage of **24**; HPLC Method 1 (achiral, Hypersil Gold C18).

S8.2 Chiral HPLC measurements to quantify stereoselectivity

Hydrobenzoin (1)

Enantioselectivity towards substrate **1** was determined by manually mixing **(*R*,*R*)-1** and **(*S*,*S*)-1** with ***meso*-1** to produce a stock solution with 20 mM total substrate in acetonitrile. Photoenzymatic diol cleavage of that mixture was performed as described in the general procedure (15 h irradiation). After irradiation and precipitation of the protein, the unreacted substrate stereoisomers were separated by HPLC method 3 (S1, chiral column). Areas listed in **Figure S35** are the averages of triplicates given in mAU·min as determined by integrating the peaks of the respective stereoisomers. While ***meso*-1** is cleaved significantly slower, no significant discrimination between **(*R*,*R*)-1** and **(*S*,*S*)-1** could be determined.

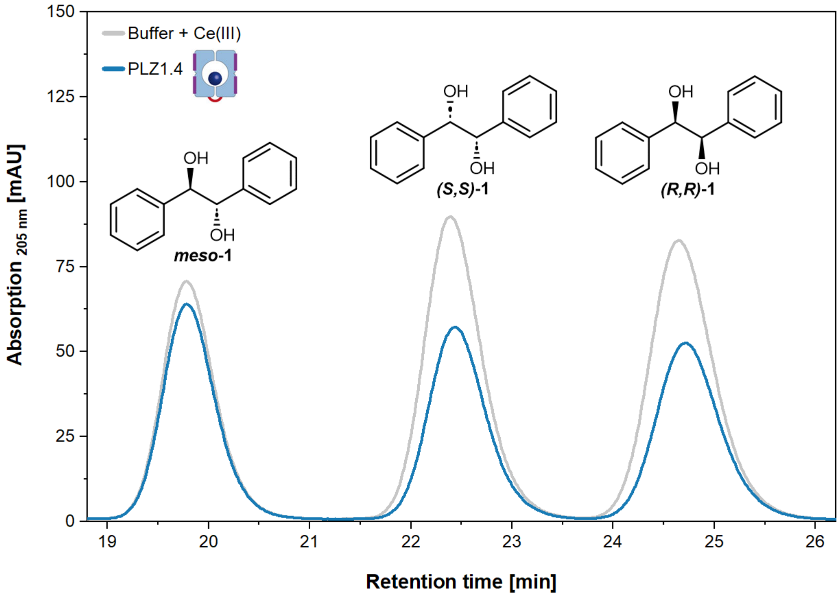

Peak areas	*meso*-1	(*S*,*S*)-1	(*R*,*R*)-1
**Buffer + $CeCl_3$**	43.2 ± 3.4	59.7 ± 4.6	60.8 ± 4.6
**PLZ1.4**	38.3 ± 1.1	36.3 ± 1.7	36.9 ± 1.7

Figure S35. HPLC chromatogram after the photocatalytic cleavage of a mixture of ***meso***-, **(*S*,*S*)-** and **(*R*,*R*)-1** with PLZ1.4 as the catalyst and buffer + $CeCl_3$ as control (Method 3, chiral, Chiralcel OJ-RH). Measurements were performed in triplicates.

1,4-Diphenylbutane-2,3-diol (19)

For evaluating the stereoselectivity of PLZ1.1 and PLZ1.4 towards the photocatalytic cleavage of **19**, triplicates of the photoreactions with the respective enzyme were set up according to the general methods. The analytes were separated on a chiral Chiralcel OJ-RH column at room temperature according to HPLC Method 2 (S1, chiral column). **Figure S36** shows the areas of the chromatogram that are relevant to quantify the unreacted substrate stereoisomers. For full chromatogram see **Figure S30**.

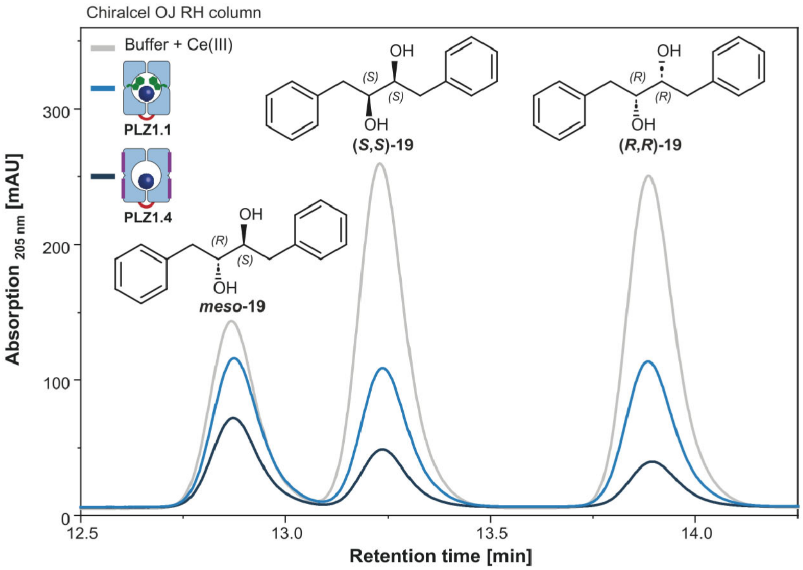

Peak areas	*meso*-19	(*S*,*S*)-19	(*R*,*R*)-19	ratio	*ee*	conversion	S value	
Buffer + Ce(III)	19.38 ± 0.08	3 5.38 ± 0.15	35.22 ± 0.11	1 : 1.83 : 1.82	-	-	-	
PLZ1.1	15.74 ± 0.17	14.54 ± 0.30	15.69 ± 0.27	1 : 0.92 : 0.99	3.8%	57.2%	1.09	preference for **(*S*,*S*)-19**
PLZ1.4	9.3 ± 0.25	5.87 ± 0.17	4.77 ± 0.04	1 : 0.63 : 0.51	10.3%	89.4%	1.12	preference for **(*R*,*R*)-19**

Figure S36. Initial stereoselectivity. Chiral HPLC traces quantifying the three stereoisomers of unreacted diol substrate **19** after overnight photocatalysis. Measurements were performed in triplicates. In addition to a pronounced diastereoselectivity, PLZ1.1 and PLZ1.4 show an initial enantioselectivity with preference for the opposite enantiomer (see values for *ee* and conversion).

Results shown **Figure S36** indicated that the stereoselectivity of the photoenzyme changed from a slight preference to cleave **(*S*,*S*)-19** to **(*R*,*R*)-19** during the optimization of the scaffold. To assess whether this change arose from the mutations in the active site or the surface of the enzyme, we additionally analyzed the stereoselectivity of PLZ1.3. This intermediate variant possesses the same active site as PLZ1.4 and the same surface as PLZ1.1. Triplicates of the photoreactions were set up according to the general methods, but the vials were only illuminated for 6 h to obtain <50% conversion. The analytes were separated on a chiral Chiralcel OJ-RH column at room temperature according to HPLC Method 2 (S1, chiral column). **Figure S37** shows the areas of the chromatogram that are relevant to quantify the unreacted substrate stereoisomers. The analysis shows that PLZ1.3 already exhibits a selectivity for **(*R*,*R*)-19**, indicating that the photoreaction takes place inside the active site.

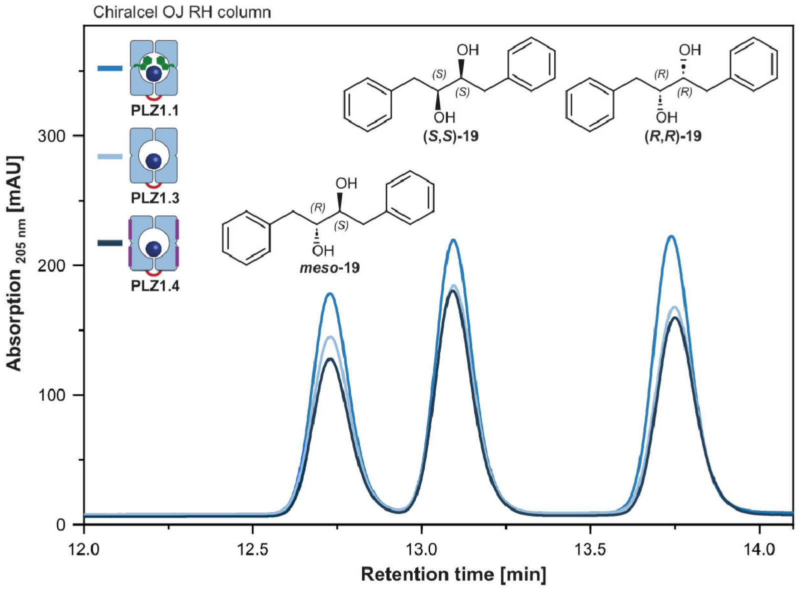

Peak areas	*meso*-19	(*S*,*S*)-19	(*R*,*R*)-19	ratio	*ee*	conversion	S value	
PLZ1.1	22.56 ± 0.56	27.86 ± 1.06	29.18 ± 1.12	1 : 1.23 : 1.29	2.3%	22.7%	1.20	preference for (*S*,*S*)-19
PLZ1.3	18.81 ± 0.24	24.07 ± 0.33	22.48 ± 0.34	1 : 1.28 : 1.20	3.4%	36.9%	1.16	preference for (*R*,*R*)-19
PLZ1.4	16.52 ± 0.29	23.45 ± 0.86	21.42 ± 0.76	1 : 1.42 : 1.30	4.5%	39.2%	1.20	preference for (*R*,*R*)-19

Figure S37. Analysis of the switch in stereoselectivity. Chiral HPLC traces quantifying the three stereoisomers of unreacted diol substrate **19** after photocatalysis over 6 h for PLZ1.1, PLZ1.3, and PLZ1.4. Measurements were performed in triplicates.

The assignment of the peaks to the corresponding stereoisomers was supported by stereoselective synthesis of **(*R*,*R*)-19** and **(*S*,*S*)-19** by catalytic asymmetric dihydroxylation.[12] See S14 and **Figure S38**:

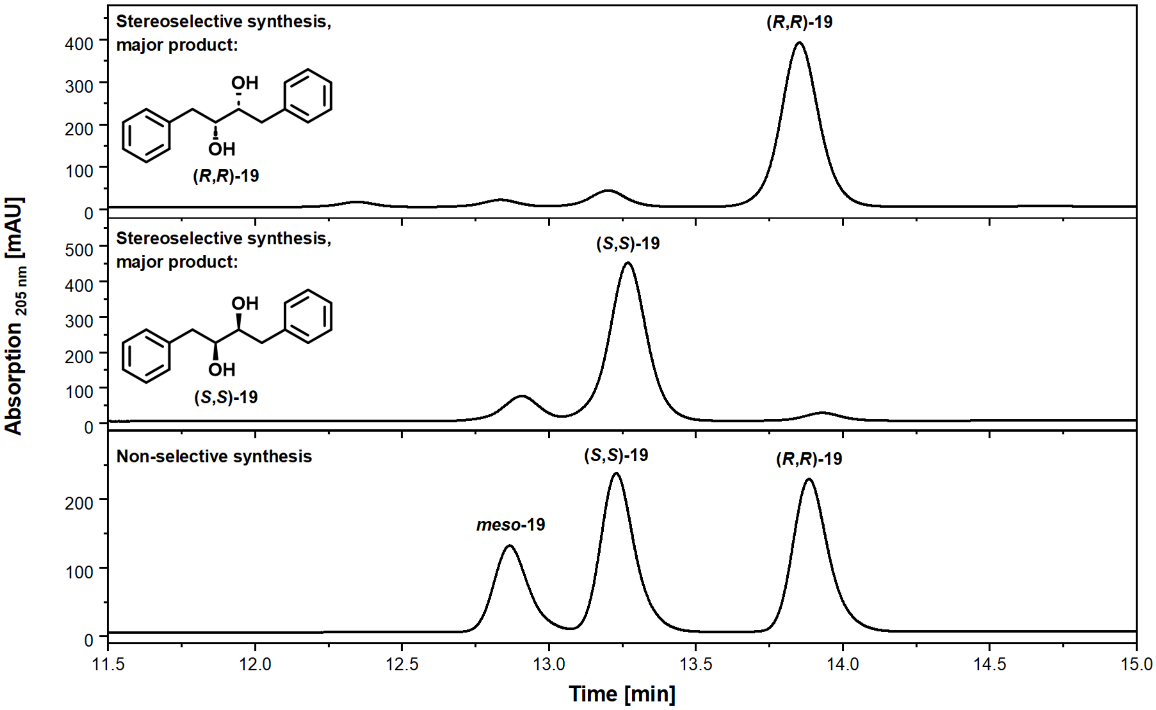

Figure S38. Assignment of stereoisomers of diol **19** to the corresponding peaks in chiral HPLC measurements (Method 2, chiral column, Chiralcel OJ-RH) using synthetic standards.

When peaks were not base line separated, they were split symmetrically using the "split peaks" function of the Chromeleon software. The areas of the peaks were determined using the Chromeleon software and the average and standard deviation of the triplicates were calculated.

Conversion *c* was determined by subtracting the area of **(*S*,*S*)-19** and **(*R*,*R*)-19** after the enzymatic photoreaction from the area of **(*S*,*S*)-19** and **(*R*,*R*)-19** in the control with $CeCl_3$ in buffer but without enzyme and dividing by the sum of areas **(*S*,*S*)-19** and **(*R*,*R*)-19** in the control. Enantiomeric excess (*ee*) was determined according to:

$$ee = \frac{|A_{S,S} - A_{R,R}|}{A_{S,S} + A_{R,R}}$$

Where $A_{S,S}$ is the area of **(*S*,*S*)-19** and $A_{R,R}$ is the area of **(*R*,*R*)-19**, respectively. These values were used to determine the selectivity factor S as follows:

$$S = \frac{ln[(1-c)(1-ee)]}{ln[(1-c)(1+ee)]}$$

For the ratio of the peaks, each area was normalized by dividing by the area of ***meso*-19** in the respective measurement.

S9 Mechanistic studies

S9.1 Observations and mechanistic proposals

During the photocatalytic cleavage of alcohol **12**, benzyl alcohol (**13**) was observed as a side product, which can be obtained by a potential oxidation of a benzyl radical intermediate **Figure S39A**). The cleavage of diol **19** also resulted in a mixture of benzyl alcohol (**13**) and benzaldehyde (**2**), while aldehyde **20** was not observed in this reaction. This is probably due to the higher stability of the benzyl radical intermediate (**Figure S39B**). The cleavage of amino alcohol **21** resulted in two molecules benzaldehyde (**2**) (**Figure S39C**). The oxidative cleavage of **21** into more than one equivalent of benzaldehyde (**2**) was previously described by *Meng et al.* (2019).[13]

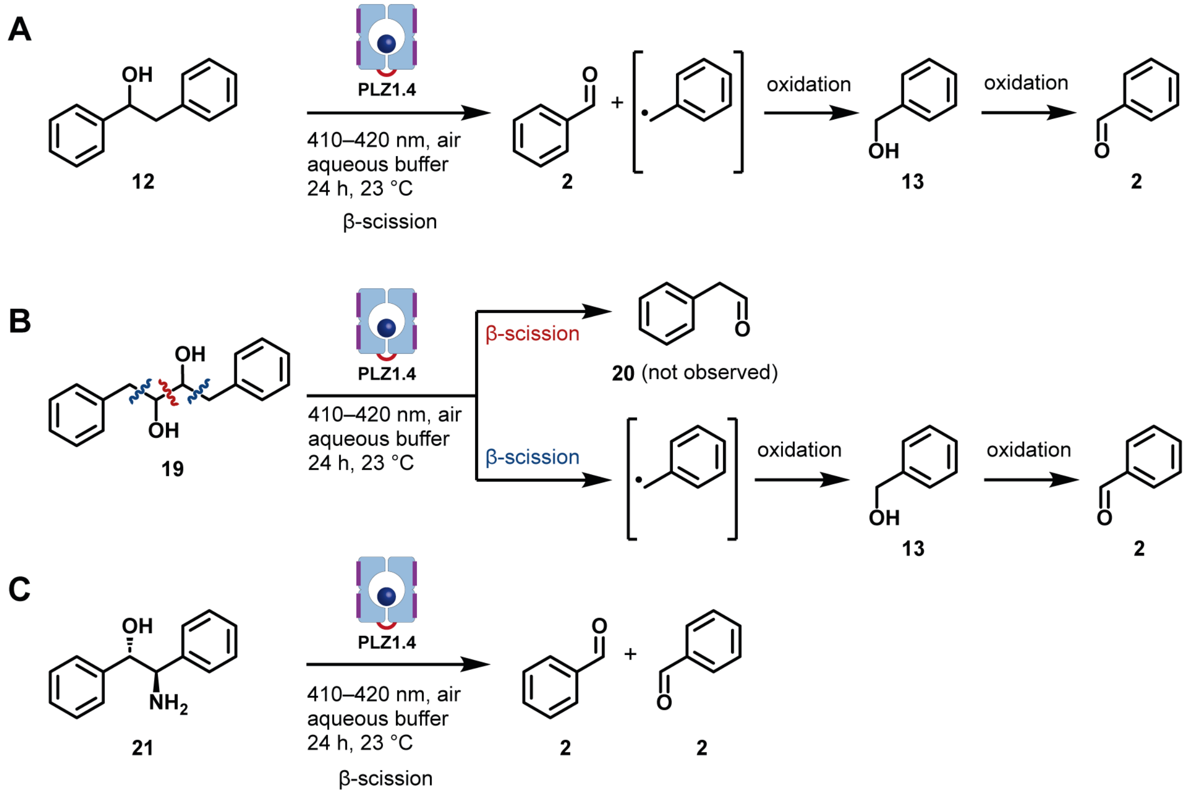

Figure S39. Observed oxidation reactions during the photocatalytic cleavage of substrates **12**, **19** and **21**.

Also for lignin surrogate **22**, we found that two equivalents of benzaldehyde were formed. Furthermore, traces of diol **3** were detected as an intermediate (**Figure S40**). We thus investigated the mechanism by radical trapping and isotope labeling (see sections below).

S9.2 Radical trapping with TEMPO

The photocatalytic cleavage of **22** and radical trapping with TEMPO were performed as described above. Two different benzylic radicals were detected as TEMPO adducts by LC-MS (**Figure S40**). However, at this scale, TEMPO adducts could not be isolated for more in-depth characterization. Therefore, these results do not provide strong mechanistic evidence, but rather help dissect the different C-C bond cleavage options for this substrate.

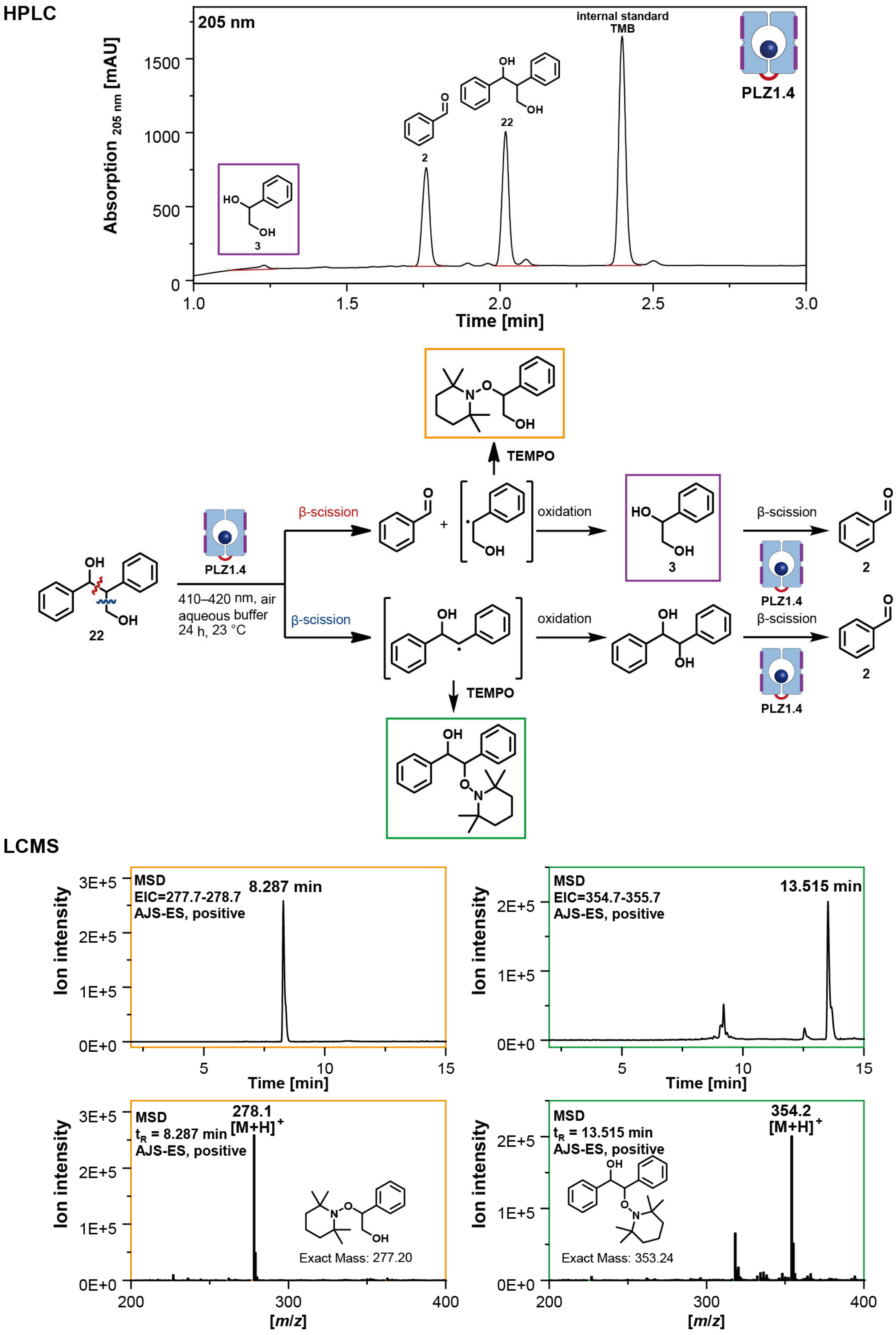

Figure S40. Photocatalytic cleavage of lignin surrogate **22**, verification of intermediate **3** by HPLC, and radical intermediate trapping with TEMPO detected by LC-MS analysis.

S9.3 ^{18}O isotope labeling

For ^{18}O isotope incorporation experiments, PLZ1.4 and $CeCl_3$ were prepared as described in the general procedures for photocatalysis. After incubation, the solution was frozen in liquid N_2 and lyophilized to remove all $H_2{}^{16}O$. Rehydration was carried out either by adding $H_2{}^{18}O$ (97% ^{18}O) or $H_2{}^{16}O$ as a control. Photocatalysis experiments were performed with 6 mM **22**, **2** or **12** for 48 h (**Figure S41**). After irradiation, the reaction mixtures were extracted with methyl *tert*-butyl ether and the isotope ratio in products was analyzed by GC-MS.

Importantly, benzaldehyde (**2**) immediately exchanges with water and incorporates ^{18}O independent of the photoreaction. We thus chose substrate **12** for the $H_2{}^{18}O$ experiment and analyzed the second product benzyl alcohol (**13**), which cannot exchange with water. Here, we saw no ^{18}O incorporation into the alcohol, suggesting the oxygen originates from O_2, not water.

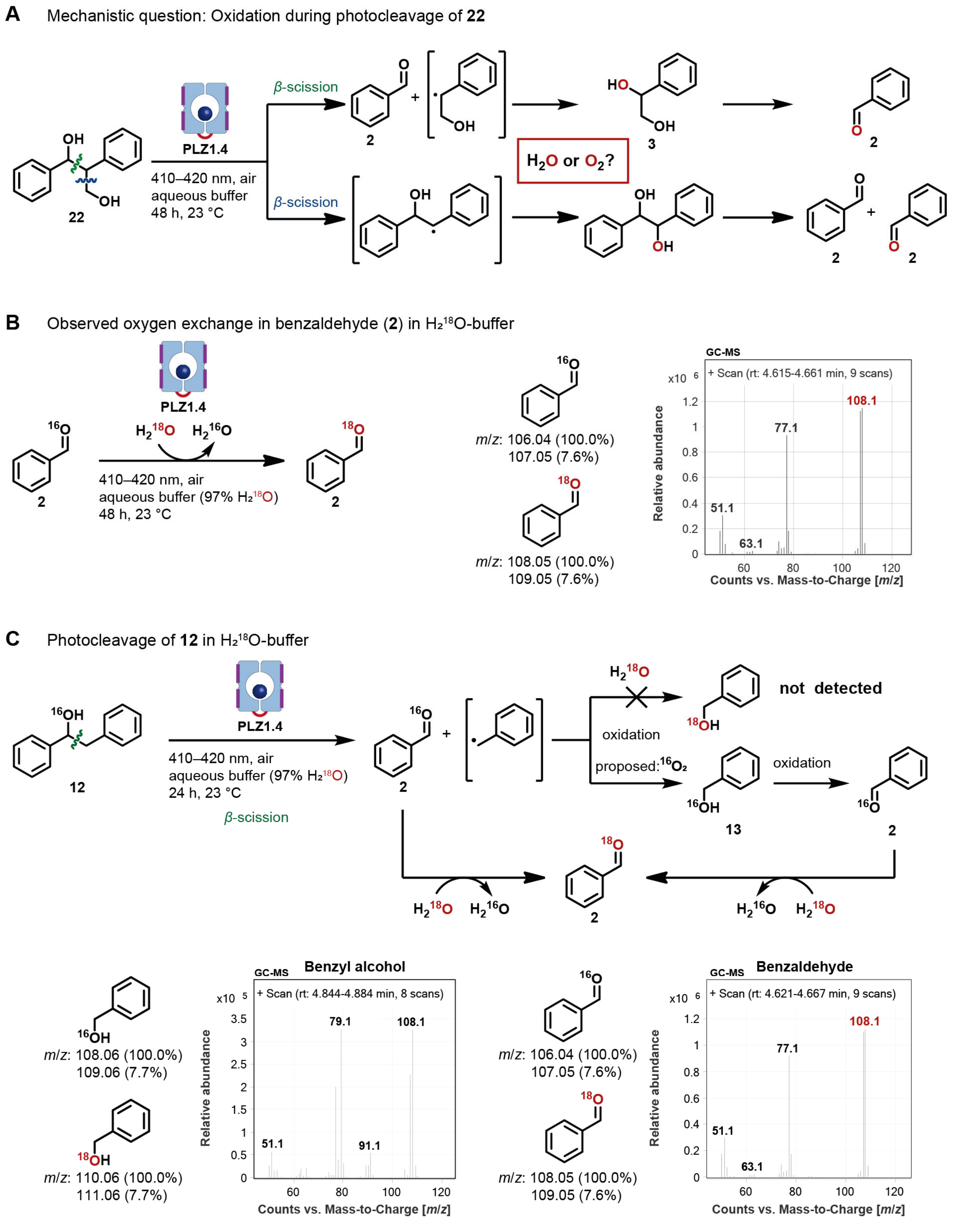

Figure S41. ^{18}O isotope incorporation during photoreactions with PLZ1.4. **(A)** Mechanistic question: What is the origin of the oxygen atom incorporated into the second equivalent of benzaldehyde during the photocleavage of **22**? **(B)** Observed $^{16}O/^{18}O$ exchange of benzaldehyde (**2**) alone. **(C)** Photocleavage of **12** in ^{18}O-buffer gives no incorporation of ^{18}O into benzyl alcohol (**13**).

S9.4 Michaelis-Menten kinetics of lignin surrogate 24

For the Michaelis Menten kinetics of lignin surrogate **24** and PLZ1.4 (**Figure S42**), a metalloenzyme solution of 55.5 µM was incubated for 2.5 h at 40 °C. After addition of 10 µL substrate solution (0–120 mM in acetonitrile) to 90 µL metalloenzyme, the photoreactions were carried out in photoreactors at 410-420 nm (3 W) at approx. 23 °C for 4 h with a final concentration of 50 µM metalloenzyme, 0–12 mM substrate and 10% (*v*/*v*) acetonitrile as co-solvent. The initial velocities of benzaldehyde (**2**) formation were quantified in triplicates, whereby a formation of one benzaldehyde molecule was assumed for the conversion of one substrate molecule **24**. The kinetic parameters k_{cat} and K_M were determined by fitting the data with the Michaelis Menten equation, with $[E]_0$ = enzyme concentration and $[S]_0$ = substrate concentration:

$$k_{obs} = \frac{v}{[E]_0} = \frac{k_{cat}\ [S]_0}{K_M + [S]_0}$$

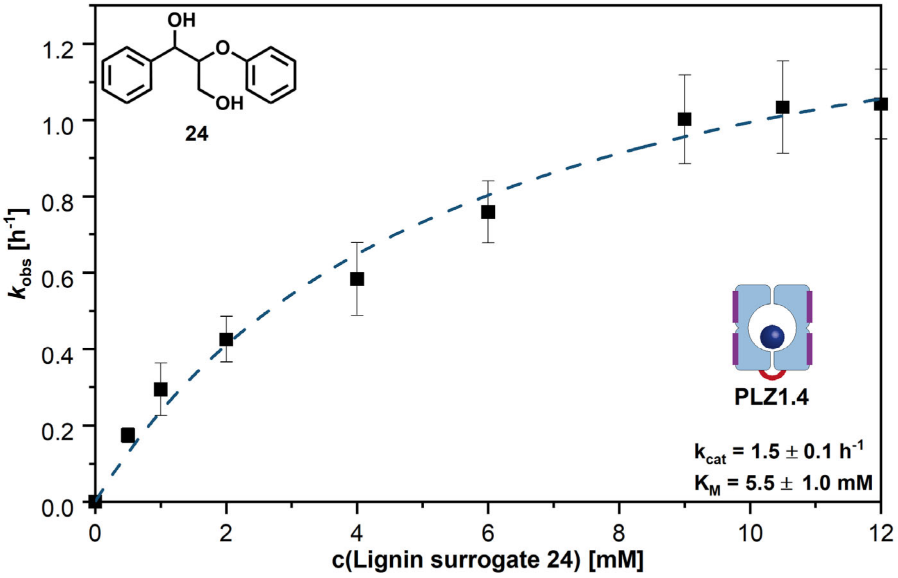

Figure S42. Michaelis-Menten kinetics for the photoenzymatic cleavage of lignin surrogate **24**.

S10 Whole-cell photobiocatalysis

Cloning of Lpp-OmpA-PLZ1.1-HA for PLZ1.1 display on the *E. coli* cell surface: The surface display vector was based on the Lpp-OmpA anchor initially reported by *Francisco et al*. (1992).[14] The *lpp* insert encoding the Lpp signal peptide and the first 9 amino acids was amplified by PCR from genomic *E. coli* BL21-Gold(DE3) DNA using the primer pair #23 and #24. The *omp*A insert encoding the amino acids 46-159 was amplified by PCR from genomic *E. coli* BL21-Gold(DE3) DNA using the primer pair #25 and #26. The backbone pET29b vector encoding PLZ1.1-HA was amplified using the primer pair #21 and #22. Amplification of the fragments was confirmed by agarose gel electrophoresis. *Dpn*I digestion (37 C, 1 h) was performed to remove methylated template DNA from PCR-amplified products. The purification of the PCR products was achieved by using the PCR Purification Kit (*Jena Bioscience* GmbH, Jena, Germany). The three fragments were assembled to the Lpp-OmpA-PLZ1.1-HA vector using an isothermal *Gibson Assembly* (1 h, 50 °C).

Recombinant expression: A 300 mL *Erlenmeyer* flask containing LB medium (50 mL) and the respective antibiotic was inoculated to an OD_{600} of 0.05–0.08 with a pre-culture of *E. coli* BL21-Gold(DE3) harboring the expression vector. The culture was incubated at 37 °C (180 rpm, orbit diameter 2.5 cm) until reaching an OD_{600} of 0.7–1.0, gene expression was induced with IPTG (final concentration 0.5 mM) and incubation was carried out for additional 4 h at 37 °C. Cells were harvested by centrifugation (25 min, 3000 rcf, 4 °C) and resuspended in wash buffer.

Isolation of the membrane fraction: The differential fractionation of *E. coli* Bl21(DE3) was adapted from a protocol described by *Maurer et al*. (1997).[15] *E. coli* cells producing surface displayed PLZ1.1 (Lpp-OmpA-PLZ1.1-HA) were harvested and resuspended in Strep wash buffer to reach a final OD_{600} = 5.0. For cell lysis, 1 mL cells were sonicated using a *Branson* SFX 500 sonifier (*Emerson Electric* Co., St. Louis, MO, USA; power-on time: 3 min, pulsed time: 2 s on, 5 s off, 15% power). The lysate was cleared by centrifugation (10 min, 6000 rcf, 4 °C) and the supernatant subjected to ultracentrifugation (Optima Max E Ultracentrifuge, Beckman, TLA-45, 30 min, 100 000 rcf). The supernatant containing cytosolic proteins was collected and the pellet resuspended in 1 mL membrane isolation buffer (40 mM HEPES, pH 8.0, 150 mM NaCl, 1 mM EDTA, 2% Trition X-100, 0.01 mM $MgCl_2$). After incubating 30 min at 25 °C, the sample was subjected to ultracentrifugation (30 min, 100 000 rcf). The pellet containing the outer membrane fraction was resuspended in 150 µL 1x SDS buffer. As negative controls, *E. coli* Bl21(DE3) cells not producing any recombinant protein were used for SDS-PAGE and Western blot analysis (**Figure S43**).

Western blot: Protein samples were separated via SDS-PAGE [15% (*w*/*v*) gel] and transferred to a nitrocellulose membrane using a semidry electro blotting device (*Biometra, Analytik Jena* GmbH+Co. KG, Göttingen, Germany; 75 mA per gel, 90 min) and 48 mM TRIS, 39 mM glycine pH 9.2, 20% (*v*/*v*) MeOH as transfer buffer. The membrane was blocked with 5% (*w*/*v*) milk powder dissolved in PBS-T [0.05% (*v*/*v*) Tween-20] for 1 h at room temperature and incubated with primary mouse-anti-HA-tag antibody [ABIN559684, antikoerper-online.de, 1:2000 in 5% (*w*/*v*) milk powder dissolved in PBS-T] overnight at 4 °C. The membrane was washed three times with PBS-T and treated with secondary HRP rabbit-anti-mouse antibody [A9044, Merck, *Merck* KGaA, Darmstadt, Germany, 1:10000 in 5% (*w*/*v*) milk powder dissolved in PBS-T] for 1 h at room temperature. After washing three times with PBS-T, the membrane was treated with WesternBright ECL-Spray (*Advansta* Inc., San Jose, CA, USA) and the chemiluminescence recorded on an ImageQuant LAS 4000 system (*GE Healthcare Technologies Inc.*, Chicago, IL, USA).

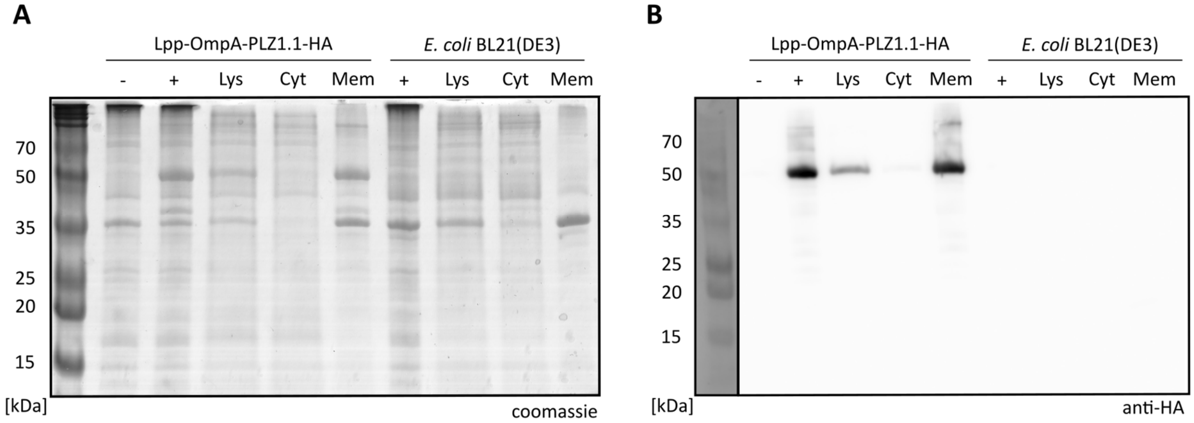

Figure S43. SDS-PAGE **(A)** and Western blot **(B)** analysis of membrane fractionation of *E. coli* Bl21(DE3) cells producing Lpp-OmpA-PLZ1.1-HA and *E. coli* Bl21(DE3) cells without recombinant expression as a negative control. [Samples before induction (–), after induction (+), cell lysate (Lys), cytoplasmic fraction (Cyt) and membrane fraction (Mem)]

Immunofluorescence staining and fluorescence microscopy for surface-displayed PLZ1.1 (Lpp-OmpA-PLZ1.1-HA): *E. coli* Bl21(DE3) cells expressing surface-displayed PLZ1.1 were harvested (3 min, 300 rcf, RT) and resuspended in wash buffer (10 mM HEPES, pH 7.0, 100 mM NaCl) to reach a final OD_{600} of 5.0. For immunofluorescence staining 1 mL cells OD_{600} 5.0 were washed once with 1 mL PBS (30 s, 14000 rcf, RT) and resuspended in 100 µL solution of primary mouse-anti HA antibody (ABIN559684, antikoerper-online.de, 1:500 in PBS). After 30 min incubation at room temperature, cells were washed with 1 mL PBS (30 s, 14000 rcf, RT) and resuspended in 100 µL secondary fluorescent (Alexa Fluor 488) donkey-anti-mouse antibody (A-21202, *Thermo Fisher Scientific* Inc., Waltham, MA, USA; 1:200 in PBS). Cells were incubated 20 min on ice and washed once with PBS (30 s, 14000 rcf, RT) before they were resuspended in the same buffer and analyzed by fluorescent microscopy (DMi8 Thunder,

Leica Camera AG Wetzlar, Germany; bright field channel: exposure time 72.6 ms; FITC channel (488 nm): exposure time 820.0 ms). As a negative control, *E. coli* Bl21(DE3) cells expressing cytosolic PLZ1.1-HA were used (**Figure S44**).

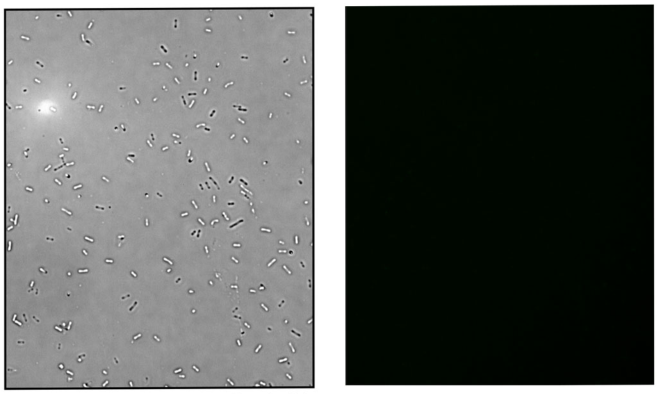

Figure S44. Negative control: fluorescence microscopy of *E. coli* Bl21(DE3) cells producing PLZ1.1-HA cytosolically.

Cerium loading and photocatalysis: For cerium loading, 166 µL of cells at OD_{600} = 50 were harvested by centrifugation (3 min, 300 rcf, RT) and resuspended in 1 mL wash buffer with or without 500 µM $CeCl_3$. Cells were incubated for 2 h at 37 °C and 700 rpm. To remove unbound cerium, cells were washed twice with 1 mL wash buffer before they were resuspended in 300 µL reaction buffer (25 mM HEPES, pH 8.5, 100 mM NaCl). All experiments were performed in triplicates.

Photoreactions were performed in 1.5 mL glass vials in a final volume of 150 µL containing *E. coli* cells with an OD_{600} of 25 and 2 mM substrate in reaction buffer and 10% (*v*/*v*) acetonitrile as co-solvent. Samples were irradiated in the photoreactors at 410–420 nm (3 W) at approx. 23 °C for 16 h. To remove cellular compounds, 150 µL acetonitrile containing the internal standard 1,2,3-Trimethoxybenzene (TMB, 200 µM) was added. After 30 min of incubation, the cellular compounds were separated by centrifugation (10 min, 12066 rcf, RT) and the supernatant was used for quantification *via* HPLC. Analytes were separated using HPLC method 1, but on a different achiral Hypersil Gold C18 column than the one used for the in vitro reactions, explaining the altered retention times.

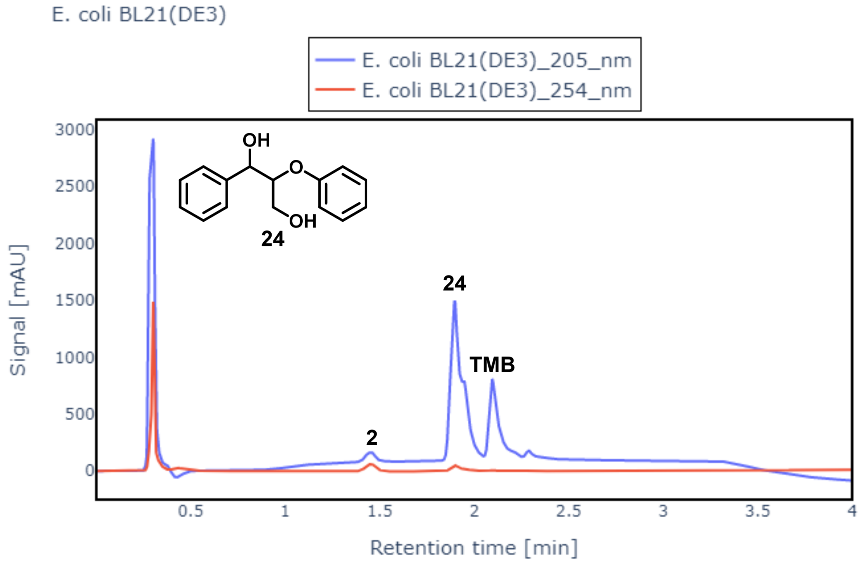

Compound	Retention time [min]	Wavelength [nm]	Area [mAU·min]
Benzaldehyde (**2**)	1.452	254	4.420
2-Phenoxy-1-phenylpropane-1,3-diol (**24**)	1.898	205	103.439
1,3,5-Trimethoxybenzene (**TMB**)	2.095	205	44.242

Figure S45. Photocatalytic cleavage of **24** using *E. coli* BL21 (DE3) whole cells not expressing any recombinant protein as control; HPLC Method 1 (achiral, Hypersil Gold C18_2020).

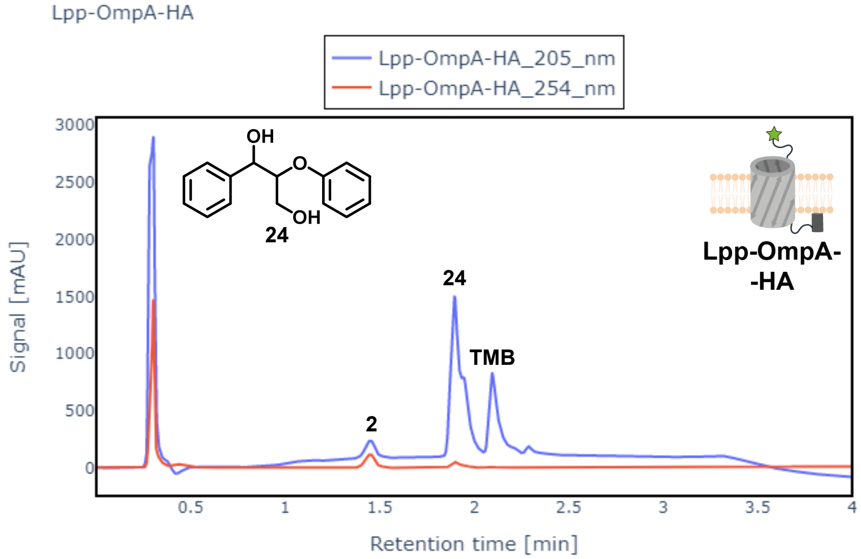

Compound	Retention time [min]	Wavelength [nm]	Area [mAU·min]
Benzaldehyde (**2**)	1.451	254	7.564
2-Phenoxy-1-phenylpropane-1,3-diol (**24**)	1.897	205	101.216
1,3,5-Trimethoxybenzene (**TMB**)	2.095	205	44.115

Figure S46. Photocatalytic cleavage of **24** using whole cells expressing Lpp-OmpA-HA as control; HPLC Method 1 (achiral, Hypersil Gold C18_2020).

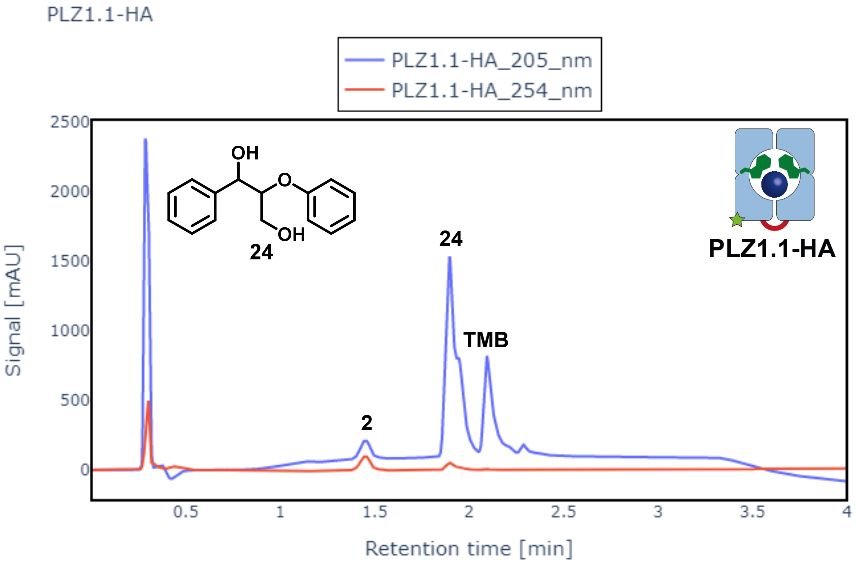

Compound	Retention time [min]	Wavelength [nm]	Area [mAU·min]
Benzaldehyde (**2**)	1.452	254	6.948
2-Phenoxy-1-phenylpropane-1,3-diol (**24**)	1.897	205	103.856
1,3,5-Trimethoxybenzene (**TMB**)	2.094	205	43.513

Figure S47. Photocatalytic cleavage of **24** using whole cells producing PLZ1.1-HA cytosolically as control; HPLC Method 1 (achiral, Hypersil Gold C18_2020).

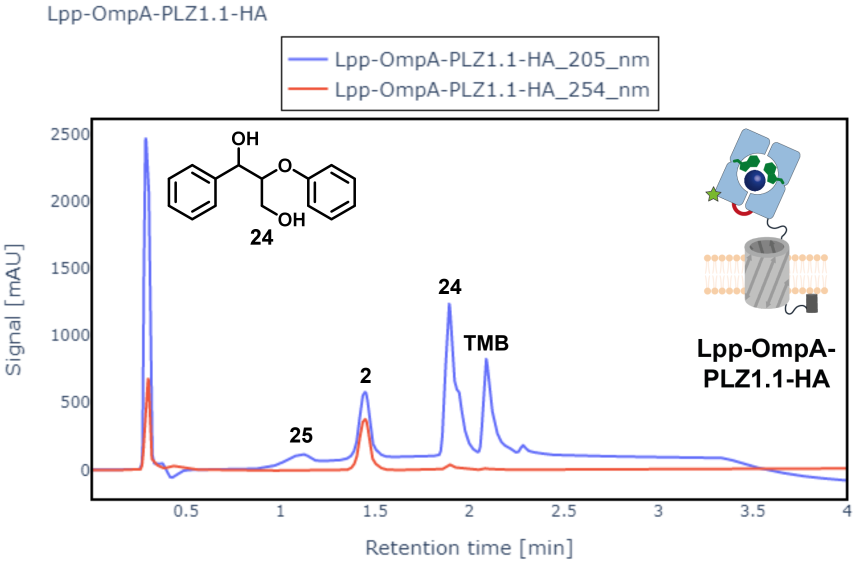

Compound	Retention time [min]	Wavelength [nm]	Area [mAU·min]
Benzaldehyde (**2**)	1.448	254	25.524
2-Phenoxy-1-phenylpropane-1,3-diol (**24**)	1.894	205	76.907
1,3,5-Trimethoxybenzene (**TMB**)	2.089	205	43.870

Figure S48. Photocatalytic cleavage of **24** using whole cells with surface-displayed Lpp-OmpA-PLZ1.1-HA; HPLC Method 1 (achiral, Hypersil Gold C18_2020). Phenol (**25**) was detected, but could not be unambiguously quantified.

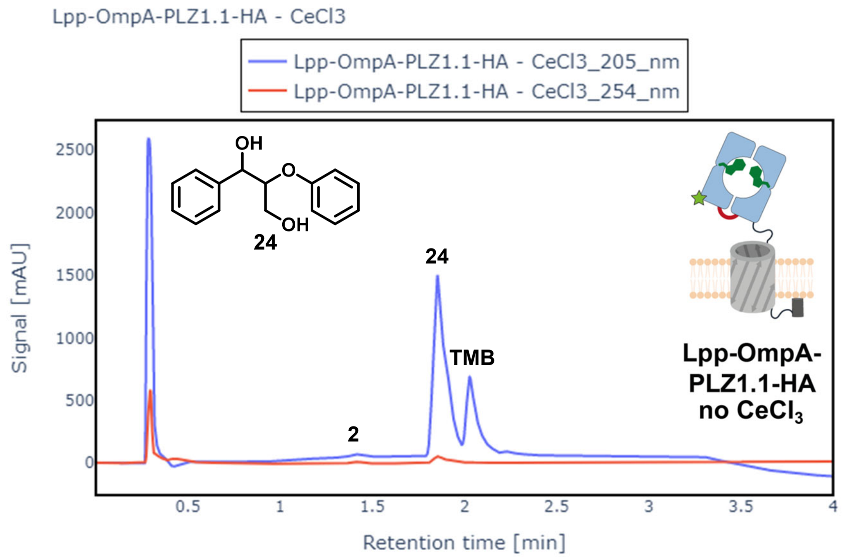

**Compound**	**Retention time [min]**	**Wavelength [nm]**	**Area [mAU·min]**
Benzaldehyde (**2**)	1.412	254	1.496
2-Phenoxy-1-phenylpropane-1,3-diol (**24**)	1.853	205	120.871
1,3,5-Trimethoxybenzene (**TMB**)	2.028	205	49.861

Figure S49. Photocatalytic cleavage of **24** using whole cells with surface-displayed Lpp-OmpA-PLZ1.1-HA without addition of $CeCl_3$; HPLC Method 1 (achiral, Hypersil Gold C18_2020).

S11 Lanthanide binding measurements with PedH

S11.1 The Tb(III) binding affinity of PedH in the absence of PQQ

Tryptophan-enhanced terbium luminescence spectra were recorded in a microplate reader (Varioskan LUX, *Thermo Fisher Scientific*) in time-resolved fluorescence mode (TRF, 50 µs delay time, 1 ms integration time, 200 ms measurement time). All experiments were performed in reaction buffer (25 mM HEPES, 100 mM NaCl, pH 8.5) that had been treated with Chelex100 resin. Additionally, PedH was also dialyzed overnight at 4 °C in the presence of Chelex100 resin to remove metal ions prior to measurement. Increasing concentrations of $TbCl_3$ (0–10 µM) in reaction buffer were prepared in black 96-well microtiter plates. Immediately after addition of protein (yielding a final concentration of 1 µM PedH in a total volume of 100 µL), the luminescence signal was monitored (λ_{ex} = 280 nm, λ_{em} = 545 nm) in intervals of 5 min over a total time of 14 h at room temperature (**Figure S50A**). The recorded spectra were analyzed and plotted using Python (version 3.9.4). The titration curve was fitted with a quadratic equation[16] using the curve_fit function of the scipy package (version 1.8.1):

$$F = F_0 + F_{Ampl} \cdot \frac{\frac{[A_0]+[B_0]+K_{d_{AB}}}{2} - \sqrt{\left(\frac{[A_0]+[B_0]+K_{d_{AB}}}{2}\right)^2 - [A_0]\cdot[B_0]}}{[A_0]}$$

The signal amplitude (F_{Ampl}) and the dissociation constant K_d between the protein and Tb(III) were treated as unknown variables for which the fit was optimized (F_0 = fluorescence signal in absence of metal, [A_0] = Protein concentration, [B_0] = concentration of $TbCl_3$, $K_{d,AB}$ = K_d of protein and Tb(III)).

The binding affinity of PedH for Tb(III) in the absence of PQQ was determined to K_D = 100 nM (**Figure S50B**).

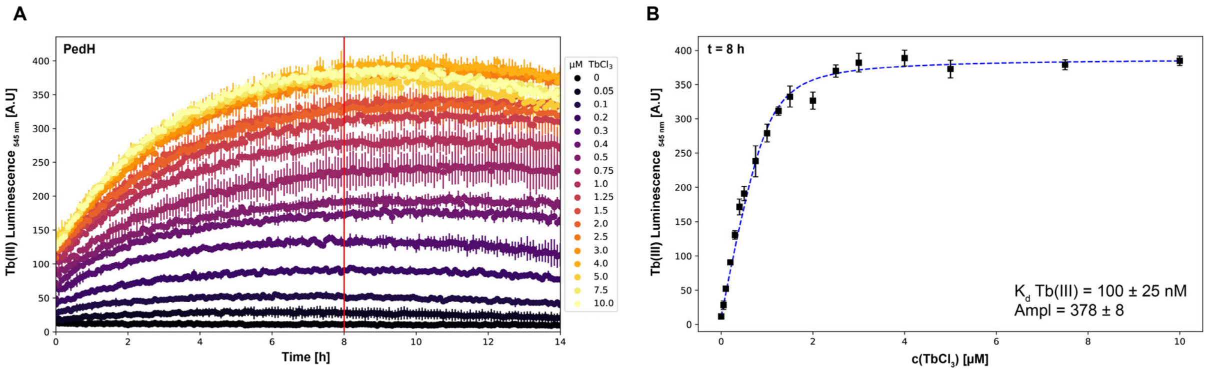

Figure S50. Tb(III) binding to PedH. (**A**) Time courses for increasing concentrations of $TbCl_3$ at 1 µM enzyme; λ_{ex} = 280 nm, λ_{em} = 545 nm) (**B**) Titration curve plotted from signals at t = 8h and quadratic fit to determine the Tb(III) binding affinity of PedH.

S11.2 The Ce(III) binding affinity of PedH in the absence of PQQ

The affinity of the protein towards Ce(III) was determined by displacing Tb(III) from the metal binding site. After pre-incubation of 1.5 µM protein and 1.5 µM $TbCl_3$ in reaction buffer (25 mM HEPES, 100 mM NaCl, pH 8.5) for 3 h at room temperature, increasing amounts of $CeCl_3$ (yielding final concentrations of 0–50 µM in a total volume of 100 µL) were added and the plate was further incubated at room temperature to reach a binding equilibrium between the protein and both competing metals. After 8 h, the tryptophan-enhanced Tb(III) luminescence signal was measured (λ_{ex} = 280 nm, λ_{em} = 545 nm). The recorded spectra were analyzed and plotted using Python (version 3.9.4) and fitted with a cubic equation[16,17] using the curve_fit function from the scipy package (version 1.8.1). The respective cubic equation is derived below with F_0 = fluorescence signal in absence of metal, [A_0] = Protein concentration, [B_0] = concentration of $TbCl_3$, [C_0] = concentration of $CeCl_3$, [AB] = concentration of the complex consisting of protein and Tb(III), K_{dAB} = K_d of protein and Tb(III) (set to 100 nM), K_{dAC} = K_d of protein and Ce(III) (unknown):

$$[AB]^3 + a_1[AB]^2 + a_2[AB] + a_3 = 0$$

$$a_0 = K_{d_{AB}} - K_{d_{AC}}$$

$$a_1 = \frac{[A_0] \cdot (K_{d_{AC}} - K_{d_{AB}}) + [B_0] \cdot (2 \cdot K_{d_{AC}} - K_{d_{AB}}) + [C_0] \cdot K_{d_{AB}} - K_{d_{AB}} \cdot K_{d_{AB}} + K_{d_{AB}} \cdot K_{d_{AC}}}{a_0}$$

$$a_2 = \frac{[A_0] \cdot [B_0] \cdot (K_{d_{AB}} - 2 \cdot K_{d_{AC}}) - [B_0]^2 \cdot K_{d_{AC}} - [B_0] \cdot K_{d_{AB}} \cdot ([C_0] + K_{d_{AC}})}{a_0}$$

$$a_3 = \frac{[A_0] \cdot [B_0]^2 \cdot K_{d_{AC}}}{a_0}$$

$$Q = \frac{a_1^2 - 3 \cdot a_2}{9}$$

$$R = \frac{2 \cdot a_1^3 - 9 \cdot a_1 \cdot a_2 + 27 \cdot a_3}{54}$$

$$\Theta = \arccos\left(\frac{R}{\sqrt{Q}^3}\right)$$

$$[AB]_1 = -2 \cdot \sqrt{Q} \cdot cos\left(\frac{\Theta}{3}\right) - \frac{a_1}{3}$$

$$[AB]_2 = -2 \cdot \sqrt{Q} \cdot cos\left(\frac{\Theta + 2 \cdot \pi}{3}\right) - \frac{a_1}{3}$$

$$[AB]_3 = -2 \cdot \sqrt{Q} \cdot cos\left(\frac{\Theta + 4 \cdot \pi}{3}\right) - \frac{a_1}{3}$$

$$F_x = F_0 + F_{Ampl} \cdot \frac{[AB]_x}{[A_0]}$$

The binding affinity of PedH for Ce(III) was determined to be ca. 7-fold weaker than for Tb(III). However, both metal ions bind with nanomolar affinity in the absence of PQQ (**Figure S51**).

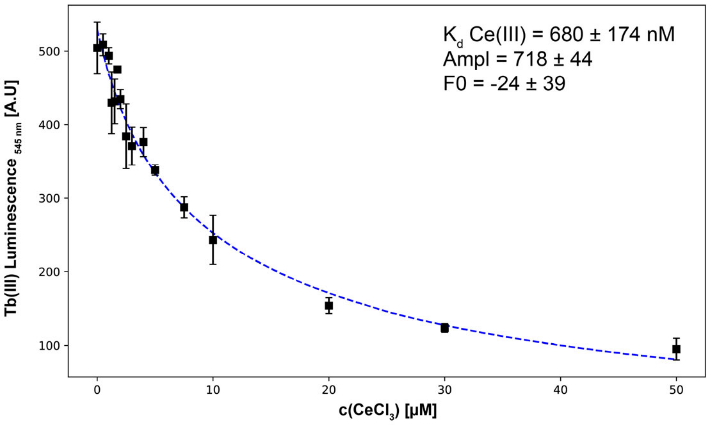

Figure S51. Ce(III) binding to PedH measured by Tb(III) displacement. 1.5 µM $TbCl_3$ and 1.5 µM PedH were incubated and then titrated with increasing concentrations of $CeCl_3$. The tryptophan-enhanced terbium luminescence signal was measured in a 96-well plate 8 h after $CeCl_3$ addition (λ_{ex} = 280 nm, λ_{em} = 545 nm) and the curve was fitted with the cubic equation with K_D(Tb(III)) = 100 nM as a constant.

S12 Molecular modeling and docking of diol substrates

S12.1 Structure prediction of PLZ1.4

PLZ1.4: The AlphaFold2 structure prediction of PLZ1.4 was generated using the ColabFold server (version 1.5.2).[18,19] The confidence scores are high for the individual domains, but low for loop regions and linkers (**Figure S52**). This is in agreement with crystallographic data available for several other variants of this de novo protein scaffold. In all cases, the four domain-connecting GGG linkers were found to be flexible, resulting in various conformations with different relative domain orientation. We calculated RMSD values between the predicted structure of PLZ1.4 and the previously determined crystal structure of Tb(III)-bound PLZ1.0 (PDB entry: 6ZV9). The overall RMSD is 3.23 Å. However, the individual domains give significantly lower RMSD values of 1.73 Å for the TIM barrel and 0.35 Å for the dimeric ferredoxin domain.

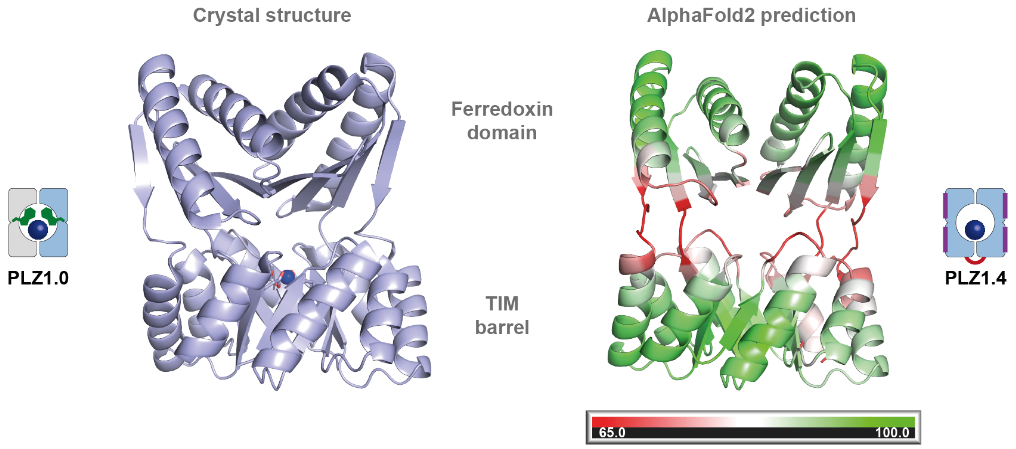

Figure S52. AlphaFold2 model of PLZ1.4. The crystal structure of PLZ1.0 (blue, PDB entry: 6ZV9) is shown on the left for comparison. The AlphaFold2 predicted structure of PLZ1.4 is shown on the right. The color code represents the confidence score of the prediction from 65% (red) to 100% (green).

S12.2 Docking of diol substrates

Molecular docking was performed using the *Maestro* molecular modeling program in *Schrödinger* (release version 2024-1, access via the Leibniz Supercomputing Center, LRZ). The proteins were prepared using the protein preparation wizard in *Maestro* to remove any clashes, remove water, add missing side-chain atoms, add hydrogen atoms followed by energy minimization with constraints on heavy atoms. The receptor grid was generated by defining the center of the binding pocket. For PedH, we selected the centroids (xyz coordinates) of the bound PQQ cofactor based on PDB entry 6ZCV. The inner box length in xyz was set to default values of 10 Å and the outer box to 20 Å. The diol substrates were prepared by the LigPrep tool in *Maestro*, generating low energy structures of all possible stereoisomers of the ligands. Docking was performed by single precision Glide docking (molecular docking tool in *Maestro*)[20] using default parameters. The resulting poses were compared in energies and analyzed in *Maestro* regarding their types of interactions with the proteins. Movies and figures were made in Pymol and ChimeraX (version 1.6.1), respectively.

To validate our procedure, we first docked the natural PQQ cofactor into PedH. This redocking of PQQ resulted in a single pose that almost perfectly resembled the PQQ-bound crystal structure (**Figure S53**).

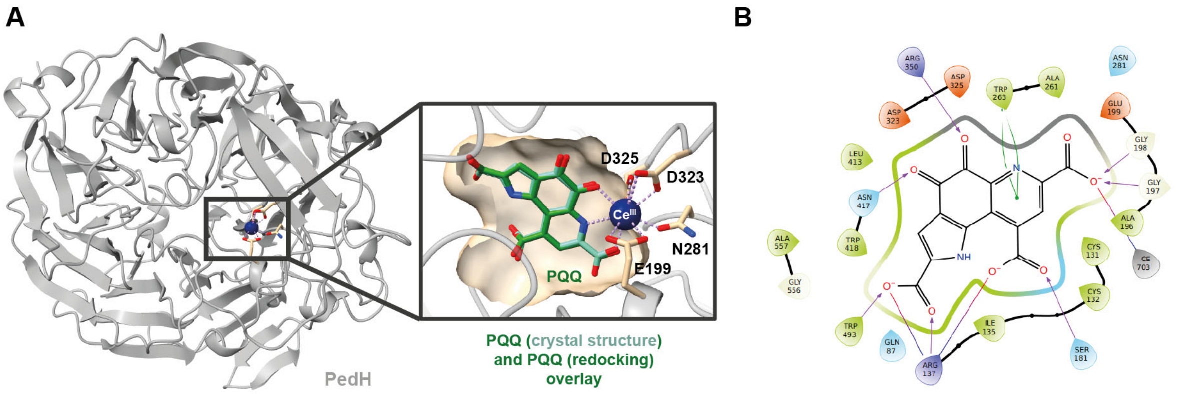

Figure S53. Validation of the docking method. **(A)** Redocking of PQQ into the crystal structure of PedH (PDB entry: 6ZCV) in the presence of Ce(III) instead of Pr(III) and **(B)** map of molecular interactions.

PLZ1.4: The docking of all stereoisomers of diol substrate **19** resulted in many different poses of similar energy (docking score: -5.3 to -4.0). It can be concluded that all stereoisomers of **19** fit into the large cavity above the active site, but that there is no clear preference for a single stereoisomer or binding conformation. **Figure S54** contrasts selected docking poses for **(*R*,*R*)-19** and **(*S*,*S*)-19**. For visualization, we prepared a video showing all obtained docking poses for PLZ1.4 (**Video S1**).

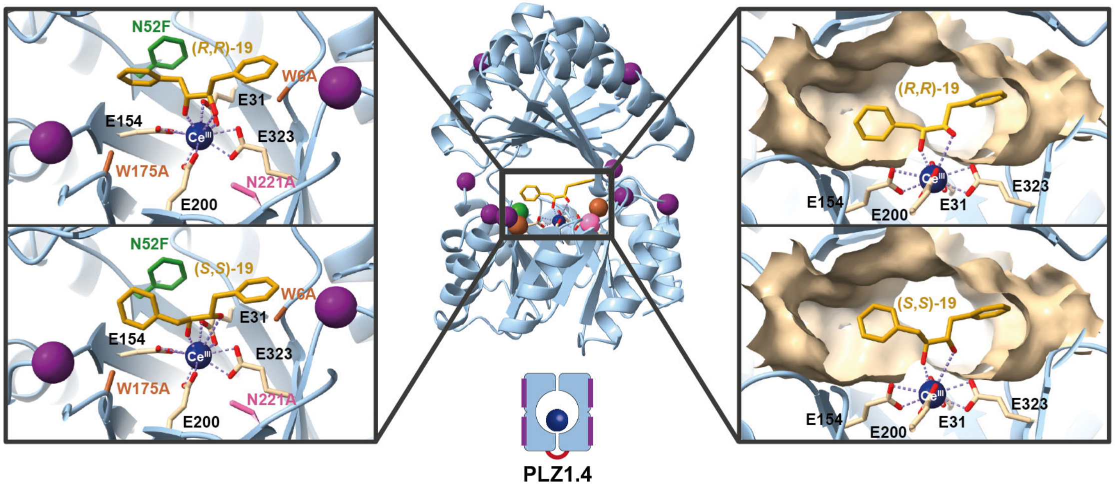

Figure S54. Docking of **(*R*,*R*)-19** and **(*S*,*S*)-19** into the AlphaFold structure prediction of PLZ1.4. Active-site mutations are shown in orange (W6A, W175A), green (N52F) and pink (N221A); surface modifications to reduce unspecific metal binding are shown as purple spheres.

PedH: The docking of hydrobenzoin (**1**) resulted in many different poses of similar energy (docking score: approx. -8). The energy scores were significantly less negative than for PQQ (docking score: -12.6). It can be assumed that all stereoisomers of **1** can bind weakly, but that there is no clear preference for a single stereoisomer or binding conformation. For visualization, we prepared a video showing all obtained docking poses for PedH (**Video S2**).

S13 Circular dichroism spectra of PLZ variants and PedH

Circular dichroism was measured as described in the general procedures to assess the folding state of different PLZ protein variants (**Figure S55**). PLZ proteins were diluted to 50 µM with ddH_2O before adding 50 µM $CeCl_3$. The mixtures were incubated at 40 °C for 2.5 h to ensure incorporation of the metal before diluting them to a final protein concentration of 10 µM with ddH_2O.

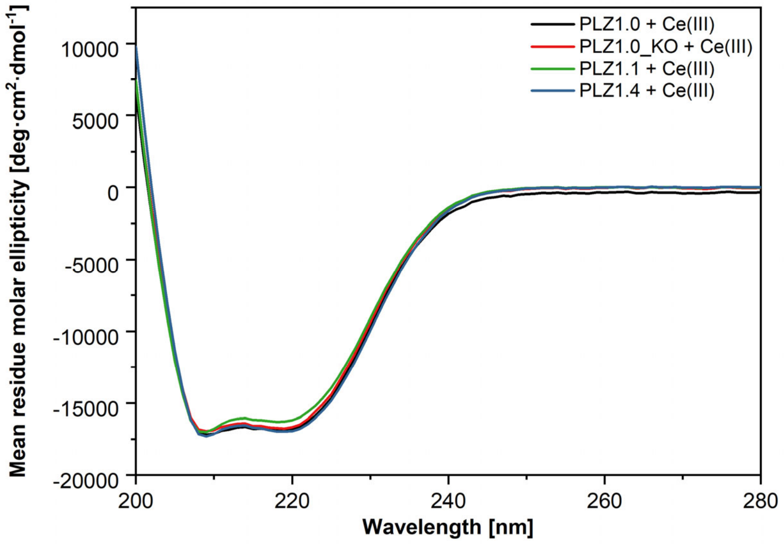

Figure S55. CD spectra of 10 µM PLZ1.0, PLZ1.0_KO, PLZ1.1 and PLZ1.4 with $CeCl_3$ in H_2O.

For the CD spectrum of PedH (**Figure S56**), the buffer of the protein solution was exchanged to CD buffer (10 mM Tris-HCl, 150 mM NaF, pH 7.5) and diluted to a final protein concentration of 10 µM. The protein's alpha-helical content is very low, which leads to a lower CD signal.

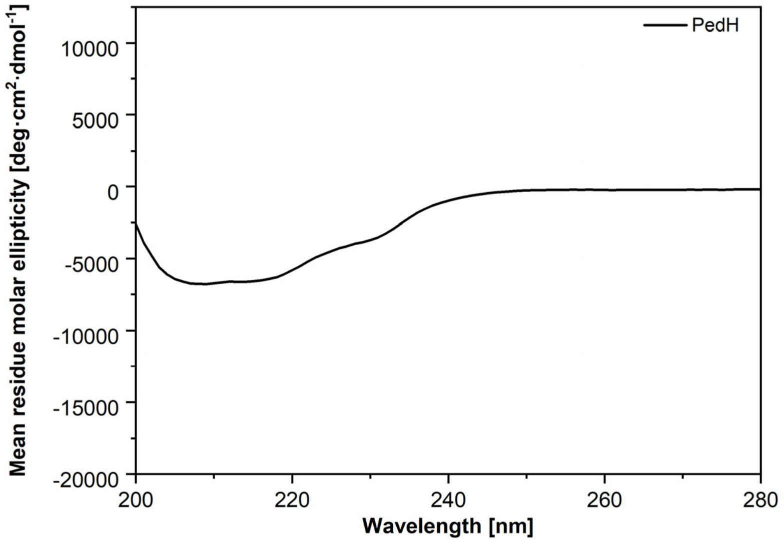

Figure S56. CD spectrum of 10 µM PedH in buffer (10 mM Tris-HCl, 150 mM NaF, pH 7.5).

S14 Chemical syntheses

General procedure for the synthesis of vicinal diols (4, 6, 8)

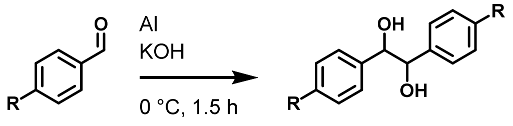

R = Me: 5
R = F: 7
R = Cl: 9

R = Me: 4
R = F: 6
R = Cl: 8

The synthesis was performed as previously described by *Kim et al.* (2014).[21] In a *Schlenk* flask under nitrogen atmosphere aluminum powder (2.0 equiv) and potassium hydroxide (9.0 equiv) were added to a solution of the *para*-substituted benzaldehyde derivative (**5**, **7** or **9**, 1.0 equiv) in MeOH (0.85 mL/mmol aldehyde) at 0 °C. After stirring for 1.5 h the resulting slurry was filtered through a pad of Celite, washed with MeOH and quenched with addition of 5% (*w*/*w*) $HCl_{(aq)}$. The reaction mixture was extracted with dichloromethane (3 x 50 mL), the combined organic layers dried over $MgSO_4$ and the solvent was removed under reduced pressure. Chromatography on silica gel (dichloromethane/MeOH) provided the *para*-substituted *vicinal* diols (**4**, **6**, **8**) as a mixture of all possible stereoisomers.

1,2-*di-para*-tolylethane-1,2-diol (4)

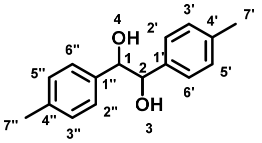

4

The *vicinal* diol **4** was obtained after column chromatography [Dichloromethane + 3% (*v/v*) MeOH] as a white solid (384 mg, 1.6 mmol, 38%). **^{1}H-NMR** (400 MHz, CD_3OD): δ [ppm] = 7.14–6.92 (m, 8H, 2′-, 3′-, 5′-, 6′-, 2″-, 3″-, 5″-, 6″-H), 4.71 and 4.58 (s, 2H, 1-, 2-H), 2.30 and 2.24 (s, 6H, 7′-, 7″-H); **^{13}C-NMR** (101 MHz, CD_3OD): δ [ppm] = 139.8 and 139.5 (C-1′, -1″), 138.1 and 137.9 (C-4′, -4″), 129.4 and 129.3 (C-3′, -5′, -3″, -5″), 128.5 and 128.4 (C-2′, -6′, -2″, -6″), 80.2 and 78.9 (C-1, -2), 21.2 and 21.1 (C-7′, -7″).

1,2-bis(*para*-fluorophenyl)ethane-1,2-diol (6)

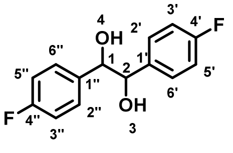

6

The *vicinal* diol **6** was obtained after column chromatography [Dichloromethane + 5% (*v/v*) MeOH] as a white solid (822 mg, 3.3 mmol, 80%). **^{1}H-NMR** (400 MHz, CD_3OD): δ [ppm] = 7.25–6.82 (m, 8H, 2′-, 3′-, 5′-, 6′-, 2″-, 3″-, 5″-, 6″-H), 4.75 and 4.63 (s, 2H, 1-, 2-H); **^{13}C-NMR** (101 MHz, CD_3OD): δ [ppm] = 164.8 + 164.7 + 162.4 + 162.3 (C-4′, -4″), 138.7 + 138.6 + 138.5 + 138.5 (C-1′, -1″), 130.3 + 130.2+ 130.2 + 130.1 (C-2′, -6′, -2″, -6″), 115.5 + 115.4 + 115.3 + 115.2 (C-3′, -5′, -3″, -5″), 79.4 and 78.2 (C-1, -2).

1,2-bis(*para*-chlorophenyl)ethane-1,2-diol (8)

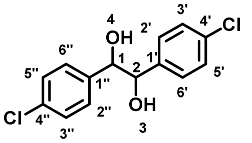

8

The *vicinal* diol **8** was obtained after column chromatography [Dichloromethane + 3% (*v/v*) MeOH] as a white solid (788 mg, 2.8 mmol, 77%). **^{1}H-NMR** (400 MHz, CD_3OD): δ [ppm] = 7.35–7.04 (m, 8H, 2′-, 3′-, 5′-, 6′-, 2″-, 3″-, 5″-, 6″-H), 4.75 and 4.64 (s, 2H, 1-, 2-H); **^{13}C-NMR** (101 MHz, CD_3OD): δ [ppm] = 141.4 and 141.3 (C-1′, -1″), 134.1 and 134.0 (C-4′, -4″), 130.1 and 129.9 (C-2′, -6′, -2″, -6″), 128.8 and 128.7 (C-3′, -5′, -3″, -5″), 79.2 and 78.1 (C-1, -2).

Synthesis of 1,2-diphenylethan-1-ol (12)

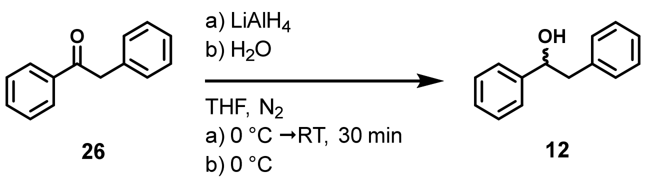

1,2-Diphenylethan-1-ol (**12**) was synthesized by a modified procedure of *Yang* and *Dudley* (2010).[22]

In a *Schlenk* flask under nitrogen atmosphere lithium aluminum hydride (134 mg, 3.5 mmol) was added to a solution of 1,2-diphenylethan-1-one (**26**, 2.0 g, 10.2 mmol) in THF (8 mL) at 0 °C. The reaction mixture was warmed to room temperature, stirred for 30 min. Afterwards the reaction was quenched with the addition of water (8 mL) at 0 °C and extracted with ethyl acetate (3 x 25 mL). The combined organic layers were washed with brine, dried over $MgSO_4$ and the solvent was removed under reduced pressure. Both enantiomers of 1,2-diphenylethan-1-ol (**12**) were obtained after column chromatography on silica gel (petrol ether/ethyl acetate, 80:20) as a white solid (2.03 g, 10.2 mmol, 100%, er = 50:50).

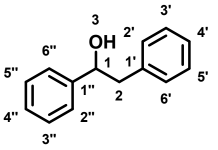

12

^{1}H-NMR (400 MHz, $CDCl_3$): δ [ppm] = 7.40–7.15 (m, 10H, 2′-, 3′-, 4′-, 5′-, 6′-, 2″-, 3″-, 4″-, 5″-, 6″-H), 4.88 (ddd, *J*=7.7 Hz, 5.0 Hz, 2.1 Hz, 1H, 1-H), 3.09–2.94 (m, 2H, 2-H), 1.97 (d, *J*=2.5 Hz, 1H, 3-OH); **^{13}C-NMR** (101 MHz, $CDCl_3$): δ [ppm] = 143.9 (C-1″), 138.2 (C-1′), 129.6 + 128.6 + 128.5 + 127.7+ 126.7 + 126.0 (C-2′, -3′, -4′, -5′, -6′, -2″, -3″, -4″, -5″, -6″), 75.5 (C-1), 46.2 (C-2).

Synthesis of 2-methoxy-1,2-diphenylethan-1-ol (14)

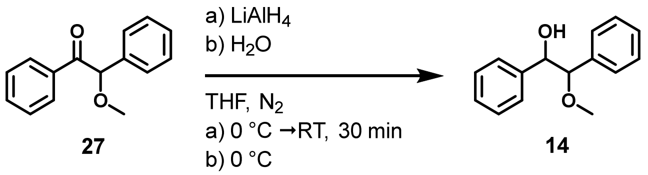

1,2-Diphenylethan-1-ol (**14**) was synthesized by a modified procedure of *Yang* and *Dudley* (2010).[22]

In a *Schlenk* flask under nitrogen atmosphere lithium aluminum hydride (282 mg, 7.4 mmol) was added portion-wise to a solution of 2-methoxy-1,2-diphenylethan-1-one (**27**, 5 g, 22 mmol) in THF (20 mL) at 0 °C. The reaction mixture was warmed to room temperature, stirred for 30 min. Afterwards the reaction was quenched with the addition of water (20 mL) at 0 °C and extracted with ethyl acetate (3 x 50 mL). The combined organic layers were washed with brine, dried over $MgSO_4$ and the solvent was removed under reduced pressure. 1,2-diphenylethan-1-ol (**14**) was obtained after column chromatography on silica gel (petrol ether/ethyl acetate, 80:20) as a white solid (4.94 g, 21.6 mmol, 98%).

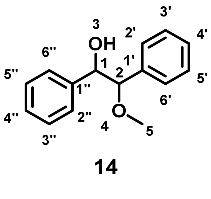

^{1}H-NMR (400 MHz, $CDCl_3$): δ [ppm] = 7.31–7.09 (m, 10H, 2′-, 3′-, 4′-, 5′-, 6′-, 2″-, 3″-, 4″-, 5″-, 6″-H), 4.88 (dd, *J*=5.4 Hz, 3.6 Hz, 1H, 1-H), 4.33 (d, *J*=5.4 Hz, 1H, 2-H), 3.22 (s, 3H, 5-H), 2.41 (d, *J*=3.8 Hz, 1H, 3-OH); **^{13}C-NMR** (101 MHz, $CDCl_3$): δ [ppm] = 140.4 (C-1″), 137.4 (C-1′), 128.2 + 128.2 + 128.1 + 128.0 + 127.7 + 127.1 (C-2′, -3′, -4′, -5′, -6′, -2″, -3″, -4″, -5″, -6″), 87.7 (C-2), 77.0 (C-1), 57.3 (C-5).

Synthesis of 1,4-diphenylbutane-2,3-diol (19)

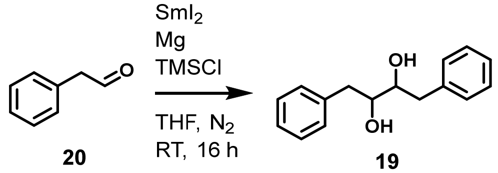

1,4-Diphenylbutane-2,3-diol (**19**) was synthesized following a protocol by *Nomura et al.* (1996).[23] In a *Schlenk* flask under nitrogen atmosphere Mg turnings (8 equiv, 0.78 g, 32 mmol) were stirred for 1 h to activate. 0.1 equiv of SmI_2 (0.1 M in THF, 4 mL, 0.4 mmol) and 0.5 equiv TMSCl (250 µL, 2 mmol) were added. 0.48 g (4 mmol) 2-phenylacetaldehyde (**20**) was mixed with 1 equiv TMSCl (510 µL, 4 mmol) and added dropwise. Upon addition, the SmI_2 mixture turned from blue to grey. The next drop of aldehyde was added when the solution had returned to blue. Due to the increasingly slow regeneration of SmI_2, a syringe pump was used to slowly add the aldehyde at a rate of ca. 0.1 mL/h.

After 16 h 1 M HCl (30 mL) was added. The mixture was extracted with ethyl acetate twice (40 mL), washed with brine and dried over Na_2SO_4. The combined organic phases were concentrated under reduced pressure. 1,4-diphenylbutane-2,3-diol (**19**) was obtained after column chromatography on silica gel (petrol ether/ethyl acetate, 85:15 to 70:30) as a white solid (155 mg, 0.64 mmol, 32%) as a mixture of all possible stereoisomers.

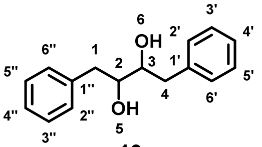

19

^{1}H-NMR (300 MHz, $CDCl_3$): δ [ppm] = 7.40–7.16 (m, 10H, 2′-, 2″-, 3′-, 3″-, 4′-, 4″-, 5′-, 5″-, 6′-, 6″-H), 3.94–3.58 (m, 2H, 2-, 3-H), 3.08–2.73 (m, 4H, 1-, 4-H), 2.09 (s, 2H, 5-OH, 6-OH); **^{13}C-NMR** (101 MHz, $CDCl_3$): δ [ppm] = 138.1 (C-1′, -1″), 129.4 + 128.6 + 126.6 (C-2′, -2″, -3′, -3″, -4′, -4″, -5′, -5″, -6′, -6″), 74.8 and 74.0 (C-2, -3), 40.4 and 38.6 (C-1, -4).

Synthesis of 1,2-dicyclohexylethane-1,2-diol (15)

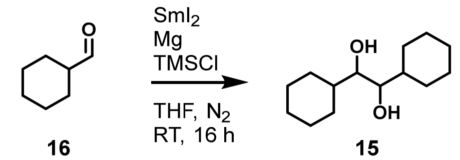

1,2-dicyclohexylethane-1,2-diol (**15**) was synthesized similarly to compound **19** (*vide supra*). In a *Schlenk* flask under nitrogen atmosphere Mg turnings (0.78 g, 32 mmol, 8 equiv) were stirred for 1 h to activate. 0.1 equiv of SmI_2 (0.1 M in THF, 4 mL, 0.4 mmol) and 0.5 equiv TMSCl (250 µL, 2 mmol) were added. Cyclohecancarbaldehyde (**16**, 485 µL 4 mmol) and 1 equiv TMSCl (510 µL, 4 mmol) were added dropwise from separate syringes. Upon addition, the SmI_2 mixture turned from blue to grey. The next drop of aldehyde and TMSCl was added when the solution had returned to blue. Due to the increasingly slow regeneration of SmI_2, syringe pumps were used to slowly add the reactants at a rate of ca. 0.1 mL/h.

After 16 h 1 M HCl (30 mL) was added. The mixture was extracted with diethyl ether twice (40 mL), washed with brine and dried over Na_2SO_4. The combined organic phases were concentrated under reduced pressure. 1,2-dicyclohexylethane-1,2-diol (**15**) was obtained after column chromatography on silica gel (petrol ether/ethyl acetate, 85:15 to 70:30) as a white solid (288 mg, 1.26 mmol, 63%) as a mixture of all possible stereoisomers.

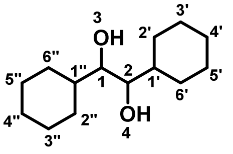

15

^{1}H-NMR (400 MHz, $CDCl_3$) δ [ppm] = 3.48–3.30 (m, 2H, 1-, 2-H), 2.13–0.77 (m, 24H, 1′-, 1″-, 2′-, 2″-, 3′-, 3″-, 4′-, 4″-, 5′-, 5″-, 6′-, 6″-H, 3-, 4-OH); **^{13}C-NMR** (101 MHz, $CDCl_3$): δ [ppm] = 75.3 (C-1, -2), 40.5 (C-1′, -1″), 29.8 + 28.4 (C-2′, -6′, -2″, -6″), 26.6 + 26.3 + 26.2 (C-3′, -4′, -5′, -3″, -4″, -5″).

Synthesis of octane-4,5-diol (17)

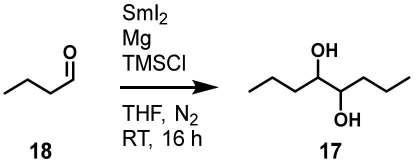

Octane-4,5-diol (**17**) was synthesized similarly to compound **19** (*vide supra*). In a *Schlenk* flask under nitrogen atmosphere Mg turnings (0.78 g, 32 mmol, 8 equiv) were stirred for 1 h to activate. 0.1 equiv of SmI_2 (0.1 M in THF, 4 mL, 0.4 mmol) and 0.5 equiv TMSCl (250 µL, 2 mmol) were added. Butyraldehyde (**18**, 360 µL 4 mmol) and 1 equiv TMSCl (510 µL, 4 mmol) were added dropwise. Upon addition, the SmI_2 mixture turned from blue to grey. The next drop of aldehyde and TMSCl was added when the solution had returned to blue. Due to the increasingly slow regeneration of SmI_2, a syringe pump was used to slowly add the reactants at a rate of ca. 0.1 mL/h.

After 16 h, 1 M HCl (30 mL) was added. The mixture was extracted with diethyl ether (40 mL) twice, washed with brine and dried over Na_2SO_4. The combined organic phases were concentrated under reduced pressure. Octane-4,5-diol (**17**) was obtained after column chromatography on silica gel (petrol ether/ethyl acetate, 80:20 to 70:30) as a white solid (10 mg, 0.07 mmol, 3%) as a mixture of all possible stereoisomers.

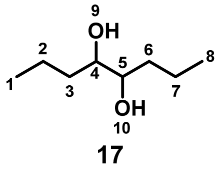

^{1}H-NMR (300 MHz, $CDCl_3$) δ [ppm] = 3.72–3.33 (m, 2H, 4-, 5-H), 1.68–1.21 (m, 8H, 2-, 3-, 6-, 7-H), 1.01–0.84 (m, 6H, 1-, 8-H); **^{13}C-NMR** (75 MHz, $CDCl_3$): δ [ppm] = 74.4 (C-4, -5), 34.6 (C--3, -6), 19.0 (C-2, -7), 14.1 (C-1, -8).

Synthesis of lignin surrogate molecule 1,2-diphenylpropane-1,3-diol (22)

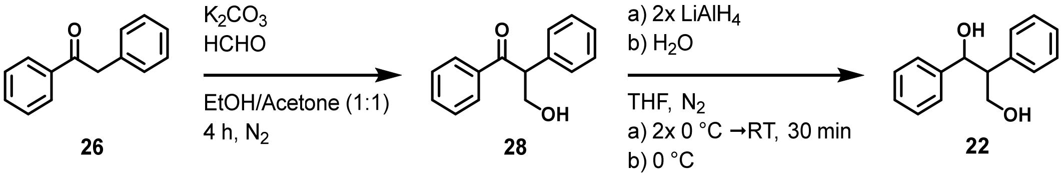

3-Hydroxy-1,2-diphenylpropan-1-one (**28**) was synthesized by a modified procedure of *Liu et al.* (2020).[24]

In a *Schlenk* flask under nitrogen atmosphere a water solution of formaldehyde [37% (*w*/*w*), 1.5 mL, 18.7 mmol] was added to a stirring solution of 1,2-diphenylethan-1-one (**26**, 2.0 g, 10.2 mmol) and potassium carbonate (1.55 g, 11.2 mmol) in a mixture of EtOH/acetone (1:1, 50 mL). After 4 h the reaction mixture was filtered through a pad of Celite and concentrated under reduced pressure. 3-hydroxy-1,2-diphenylpropan-1-one (**28**) was obtained after column

chromatography on silica gel (petrol ether/ethyl acetate, 90:10) as white solid (669 mg, 2.96 mmol, 29%).

1,2-diphenylpropane-1,3-diol (**22**) was synthesized by a modified procedure of *Yang* and *Dudley* (2010).[22]

In a *Schlenk* flask under nitrogen atmosphere lithium aluminum hydride (28 mg, 0.73 mmol) was added to a solution of 3-hydroxy-1,2-diphenylpropan-1-one (**28**, 484 mg, 2.14 mmol) in THF (5 mL) at 0 °C. The reaction mixture was warmed to room temperature, stirred for 30 min and was cooled again to 0 °C. Lithium aluminum hydride (56 mg, 1.47 mmol) was added in 2 portions to the reaction mixture. The reaction was again warmed to room temperature and stirred for additional 30 min. Afterwards the reaction was quenched with the addition of water (5 mL) at 0 °C and extracted with ethyl acetate (3 x 20 mL). The combined organic layers were washed with brine, dried over $MgSO_4$ and the solvent was removed under reduced pressure. 1,2-diphenylpropane-1,3-diol (**22**) was obtained after column chromatography on silica gel (petrol ether/ethyl acetate, 65:35) as a white solid (417 mg, 1.83 mmol, 86%, dr = 7:93).

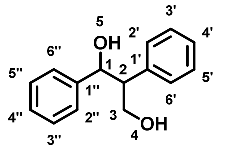

22

H-NMR (400 MHz, $CDCl_3$): δ [ppm] = 7.37–6.99 (m, 10H, 2′-, 3′-, 4′-, 5′-, 6′-, 2″-, 3″-, 4″-, 5″-, 6″-H), 5.03 (d, *J*=7.1 Hz, 1H, 1-H), 3.90–3.64 (m, 2H, 3-H), 3.16 (q, *J*=6.6 Hz, 1H, 2-H), 2.17 (d, *J*=4.9 Hz, 1H, 5-OH), 1.66–1.42 (brs, 1H, 4-OH); **^{13}C-NMR** (101 MHz, $CDCl_3$): δ [ppm] = 142.1 (C-1′), 138.7 (C-1″), 129.2 + 128.8 + 128.5 + 128.0 + 127.5 + 126.7 (C-2′, -3′, -4′, -5′, -6′, -2″, -3″, -4″, -5″, -6″), 75.9 (C-1), 64.2 (C-3), 55.9 (C-2).

Synthesis of lignin surrogate molecule 2-phenoxy-1-phenylethan-1-ol (23)

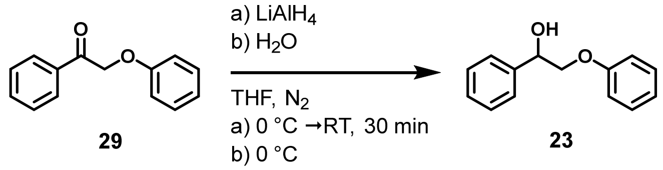

2-Phenoxy-1-phenylethan-1-ol (**23**) was synthesized by a modified procedure of *Yang* and *Dudley* (2010).[22]

In a *Schlenk* flask under nitrogen atmosphere lithium aluminum hydride (30 mg, 0.78 mmol) was added to a solution of 2-phenoxy-1-phenylethan-1-one (**29**, 500 mg, 2.36 mmol) in THF (5 mL) at 0 °C. The reaction mixture was warmed to room temperature, stirred for 30 min. Afterwards the reaction was quenched with the addition of water (5 mL) at 0 °C and extracted with ethyl acetate (3 x 20 mL). The combined organic layers were washed with brine, dried over $MgSO_4$ and the solvent was removed under reduced pressure. 2-phenoxy-1-phenylethan-1-ol (**23**)

was obtained after column chromatography on silica gel (petrol ether/ethyl acetate, 80:20) as a white solid (332 mg, 1.55 mmol, 66%).

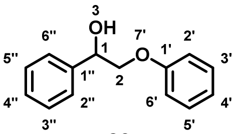

23

^{1}H-NMR (400 MHz, $CDCl_3$): δ [ppm] = 7.50–7.22 (m, 7H, 3′-, 5′-,2″-, 3″-, 4″-, 5″-, 6″-H), 7.02–6.85 (m, 3H, 2′-, 4′-, 6′-H), 5.13 (dt, *J*=8.9 Hz, 2.9 Hz, 1H, 1-H), 4.20–3.94 (m, 2H, 2-H), 2.77 (s, 1H, 3-OH); **^{13}C-NMR** (101 MHz, $CDCl_3$): δ [ppm] = 158.4 (C-1′), 139.7 (C-1″), 129.6 + 128.6 + 128.2 + 126.3 (C-3′, -5′, -2″, -3″, -4″, -5″, -6″), 121.3 (C-4′), 114.7 (C-2′, -6′), 73.3 (C-2), 72.6 (C-1).

Synthesis of lignin surrogate molecule 2-phenoxy-1-phenylpropane-1,3-diol (24)

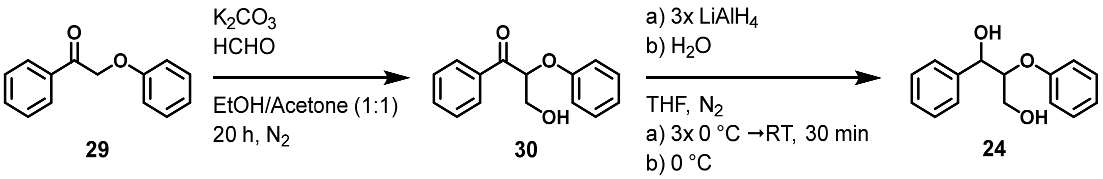

3-Hydroxy-2-phenoxy-1-phenylpropan-1-one (**30**) was synthesized by a modified procedure of *Liu et al.* (2020).[24]

In a *Schlenk* flask under nitrogen atmosphere a water solution of formaldehyde [37% (*w*/*w*), 491 µL, 6.1 mmol] was added to a stirring solution of 2-phenoxy-1-phenylethan-1-one (**29**, 700 mg, 3.3 mmol) and potassium carbonate (502 mg, 3.6 mmol) in a mixture of EtOH/acetone (1:1, 16 mL). After 20 h the reaction mixture was filtered through a pad of Celite and concentrated under reduced pressure. 3-hydroxy-2-phenoxy-1-phenylpropan-1-one (**30**) was obtained after column chromatography on silica gel (petrol ether/ethyl acetate, 90:10 to 85:15) as colorless oil (166 mg, 0.69 mmol, 21%).

2-Phenoxy-1-phenylpropane-1,3-diol (**24**) was synthesized by a modified procedure of *Yang* and *Dudley* (2010).[22]

In a *Schlenk* flask under nitrogen atmosphere lithium aluminum hydride (9 mg, 225 µmol) was added to a solution of 3-hydroxy-2-phenoxy-1-phenylpropan-1-one (**30**, 165 mg, 0.68 mmol) in THF (5 mL) at 0 °C. The reaction mixture was warmed to room temperature, stirred for 30 min and was cooled again to 0 °C and the procedure of the addition of lithium aluminum hydride was repeated two times. Afterwards the reaction was quenched with the addition of water (5 mL) at 0 °C and extracted with ethyl acetate (3 x 25 mL). The combined organic layers were washed with brine, dried over $MgSO_4$ and the solvent was removed under reduced pressure. 2-phenoxy-1-phenylpropane-1,3-diol (**24**) was obtained after column chromatography on silica gel (petrol ether/ethyl acetate, 70:30) as colorless oil (156 mg, 0.64 mmol, 94%, dr = 25:75).

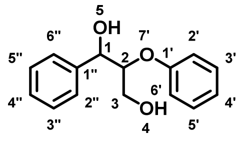

24

H-NMR (400 MHz, $CDCl_3$): δ [ppm] = 7.54–7.20 (m, 7H, 3′-, 5′-,2″-, 3″-, 4″-, 5″-, 6″-H), 7.16–6.82 (m, 3H, 2′-, 4′-, 6′-H), 5.08 (m, 1H, 1-H), 4.43 (dt, *J*=6.7 Hz, 4.0 Hz, 1H, 2-H), 3.98–3.52 (m, 2H, 3-H), 2.85 (brs, 1H, 5-OH), 2.22 and 1.85 (brs, 1H, 4-OH); ^{13}**C-NMR** (101 MHz, $CDCl_3$): δ [ppm] = 158.2 and 157.7 (C-1′), 140.4 and 139.8 (C-1″), 129.9 + 129.8 + 128.7 + 128.5 + 128.1 + 127.1 + 126.4 (C-3′, -5′, -2″, -3″, -4″, -5″, -6″), 122.2 and 122.1 (C-4′), 116.7 (C-2′, -6′), 83.2 and 82.0 (C-2), 74.2 and 74.1 (C-1), 61.4 and 61.2 (C-3).

Synthesis of (2*R*,3*R*)-1,4-diphenylbutane-2,3-diol [(*R*,*R*)-19] and (2*S*,3*S*)-1,4-diphenylbutane-2,3-diol [(*S*,*S*)-19]

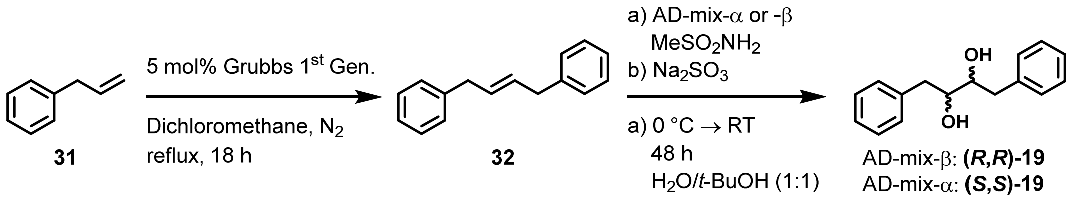

(*E*)-1,4-Diphenylbut-2-ene (**32**) was synthesized by a modified procedure of *Banwell et al.* (2006).[25]

In a *Schlenk* flask under nitrogen atmosphere 1st generation Grubbs catalyst (M102, 695 mg, 845 µmol) was added to a solution of allyl benzene (**31**, 2.0 g, 16.9 mmol) in dry and degassed dichloromethane (40 mL). The reaction mixture was heated at reflux for 18 h, cooled to room temperature and concentrated under reduced pressure. The residue was purified by column chromatography on silica gel (petrol ether/ethyl acetate, 100:0 to 98:2) and (*E*)-1,4-Diphenylbut-2-ene (**32**) was obtained as white solid (820 mg, 3,9 mmol, 47%).

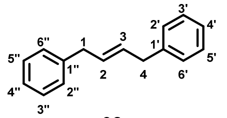

32

^{1}**H-NMR** (400 MHz, $CDCl_3$): δ [ppm] = 7.33–7.14 (m, 10H, 2′-, 3′-, 4′-, 5′-, 6′-, 2″-, 3″-, 4″-, 5″-, 6″-H), 5.69 (dddd, *J*=16.3 Hz, 5.4 Hz, 4.1 Hz, 1.4 Hz, 2H, 2-, 3-H), 3.55–3.32 (m, 4H, 1-, 4-H); ^{13}**C-NMR** (101 MHz, $CDCl_3$): δ [ppm] = 140.8 (C-1′, --1″), 130.5 (C-2, -3), 128.6 + 128.5 + 128.4 + 126.0 (C-2′, -3′, -4′, -5′, -6′, -2″, -3″, -4″, -5″, -6″), 39.0 (C-1, -4).

(2*R*,3*R*)-1,4-diphenylbutane-2,3-diol [**(*R*,*R*)-19**] and (2*S*,3*S*)-1,4-diphenylbutane-2,3-diol [**(*S*,*S*)-19**] were synthesized were synthesized by a modified procedure of *Gresser et al.* (2010).[26]

(2*R*,3*R*)-1,4-diphenylbutane-2,3-diol [**(*R*,*R*)-19**]: A solution of AD-mix β (2.695 g, 3,46 mmol) and methanesulfonamide (183 mg, 1.92 mmol) in a mixture of water/*t*-BuOH (1:1, 30 mL) was cooled to 0 °C. (*E*)-1,4-Diphenylbut-2-ene (**32**, 394 mg, 1.89 mmol) was added to the reaction mixture and the resulting slurry was stirred for 48 h at room temperature. Sodium sulfite (2.87 g,

22.75 mmol) was added and stirring was continued for additional 30 min. The reaction mixture was extracted with ethyl acetate (3 x 20 mL), the combined organic layers were washed with 2 M KOH (2 x 15 mL), dried over $MgSO_4$ and the solvent was removed under reduced pressure. (2*R*,3*R*)-1,4-diphenylbutane-2,3-diol [**(*R*,*R*)-19**] was obtained after column chromatography on silica gel (dichloromethane/MeOH 99:1 to 90:10) as a white solid (223 mg, 0.92 mmol, 48%, (*R*,*R*):(*S*,*S*):*meso* = 87.7:8.6:3.7; **Figure S57**).

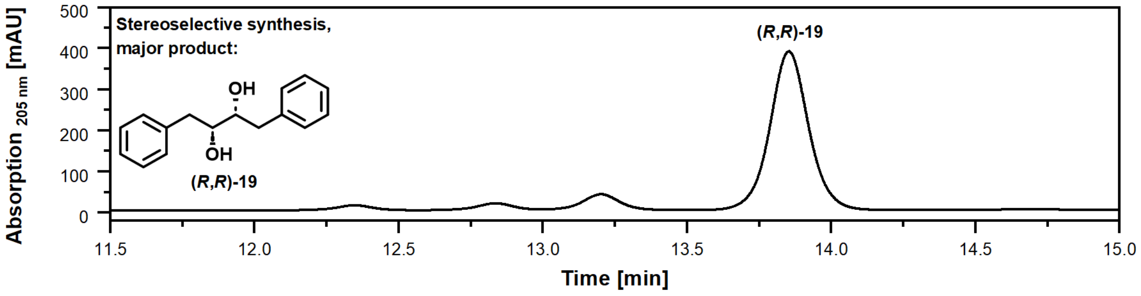

Figure S57. HPLC chromatogram of the stereoselective synthesis of **(*R*,*R*)-19**; Method 2 (chiral, Chiralcel OJ-RH).

(2*S*,3*S*)-1,4-diphenylbutane-2,3-diol [**(*S*,*S*)-19**]: A solution of AD-mix α (2.36 g, 3,03 mmol) and methanesulfonamide (162 mg, 1.70 mmol) in a mixture of water/*t*-BuOH (1:1, 25 mL) was cooled to 0 °C. (*E*)-1,4-Diphenylbut-2-ene (**32**, 337 mg, 1.62 mmol) was added to the reaction mixture and the resulting slurry was stirred for 48 h at room temperature. Sodium sulfite (2.51 g, 19.91 mmol) was added and stirring was continued for additional 30 min. The reaction mixture was extracted with ethyl acetate (3 x 20 mL), the combined organic layers were washed with 2 M KOH (2 x 15 mL), dried over $MgSO_4$ and the solvent was removed under reduced pressure. (2*S*,3*S*)-1,4-diphenylbutane-2,3-diol [**(*S*,*S*)-19**] was obtained after column chromatography on silica gel (dichloromethane/MeOH 99:1 to 90:10) as a white solid (375 mg, 1.55 mmol, 96%, (*R*,*R*):(*S*,*S*):*meso* = 4.1:82.9:13; **Figure S58**).

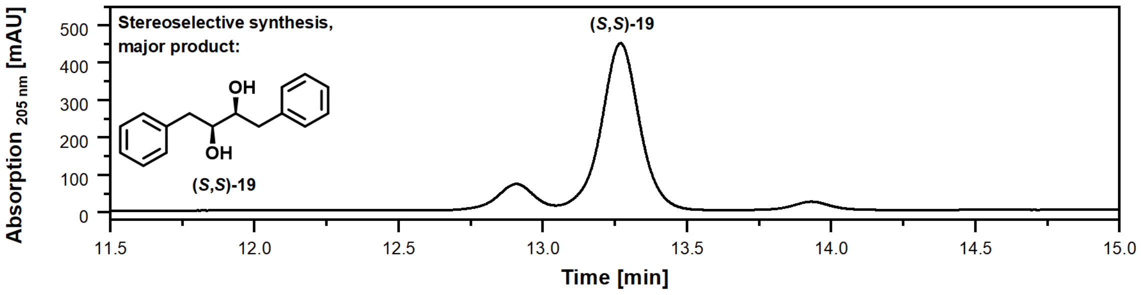

Figure S58. HPLC chromatogram of the stereoselective synthesis of **(*S*,*S*)-19**; Method 2 (chiral, Chiralcel OJ-RH).

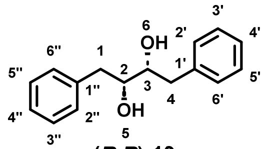
(*R*,*R*)-19

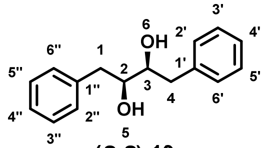
(*S*,*S*)-19

^{1}H-NMR (400 MHz, CD_3OD): δ [ppm] = 7.11–7.29 (m, 10H, 2′-, 3′-, 4′-, 5′-, 6′-, 2″-, 3″-, 4″-, 5″-, 6″-H), 4.57 (brs, 5-, 6-OH), 3.61–3.70 (m, 2H, 2-, 3-H), 2.73–2.96 (m, 4H, 1-, 4-H); **^{13}C-NMR** (101 MHz, CD_3OD): δ [ppm] = 140.6 (C-1′, -1″), 130.4 + 129.2 + 127.0 (C-2′, -3′, -4′, -5′, -6′, -2″, -3″, -4″, -5″, -6″), 75.3 (C-2, -3), 40.8 (C-1, -4).

The assignment of the stereoisomer formed as the major product in catalytic *Sharpless* asymmetric dihydroxylation is based on *Kolb et al.* (1994).[12]

Synthesis of 2-aminobenzamidoxime (ABAO, 34)

ABAO (**34**) was synthesized by a procedure of *Ressmann et al.* (2019).[5]

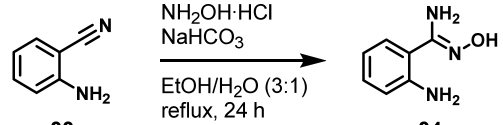

2-aminobenzonitrile (**33**, 1.18 g, 10 mmol) was dissolved in EtOH (18 mL) and added to H_2O (6 mL). Hydroxylamine·HCl (15 mmol, 1.04 g) and $NaHCO_3$ (18 mmol, 1.51 g) were added and the mixture was heated to reflux for 24 h. The pH of the mixture was adjusted to pH = 7 by adding 1 M $HCl_{(aq.)}$. Subsequently the EtOH was removed under reduced pressure and the remaining aqueous phase was extracted with ethyl acetate (40 mL) twice. The combined organic phases were washed with brine, dried over $MgSO_4$ and the solvent was removed under reduced pressure. The remaining solid was washed with dichloromethane/petrol ether (1:1) to yield ABAO (**34**) as a white solid (1.01 g, 66.9 mmol, 67%)

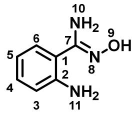
34

^{1}H-NMR (300 MHz, DMSO-d6): δ [ppm] = 9.55 (s, 1H, 9-OH), 7.36 (d, *J*=7.8 Hz, 1H, 6-H), 7.02 (t, *J*=7.6 Hz, 1H, 4-H), 6.66 (d, *J*=8.1 Hz, 1H, 3-H), 6.53 (t, *J*=7.5 Hz, 1H, 5-H), 6.20 (s, 2H, 10-NH_2), 5.71 (s, 2H, 11-NH_2); **^{13}C-NMR** (75 MHz, DMSO-d6): δ [ppm] = 153.3 (C-7), 147.2 (C-2), 129.4 (C-4), 127.7 (C-6), 115.9 (C-5), 115.3 (C-3), 114.7 (C-1).

S15 NMR spectra

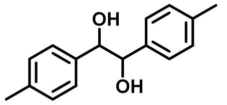

4

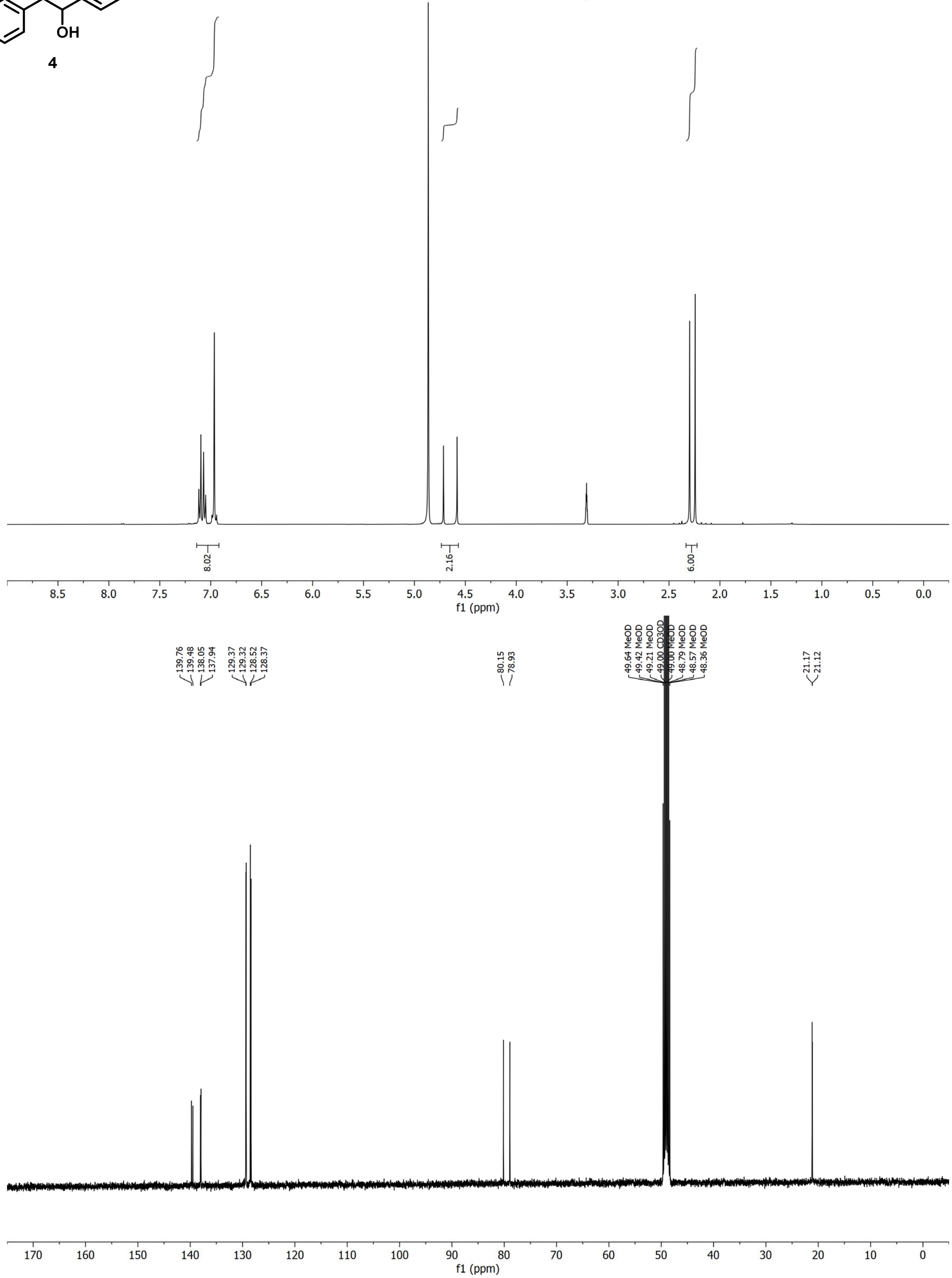

Figure S59. ^{1}H- and ^{13}C-NMR-spectra of diol **4** in CD_3OD (400 MHz/101 MHz).

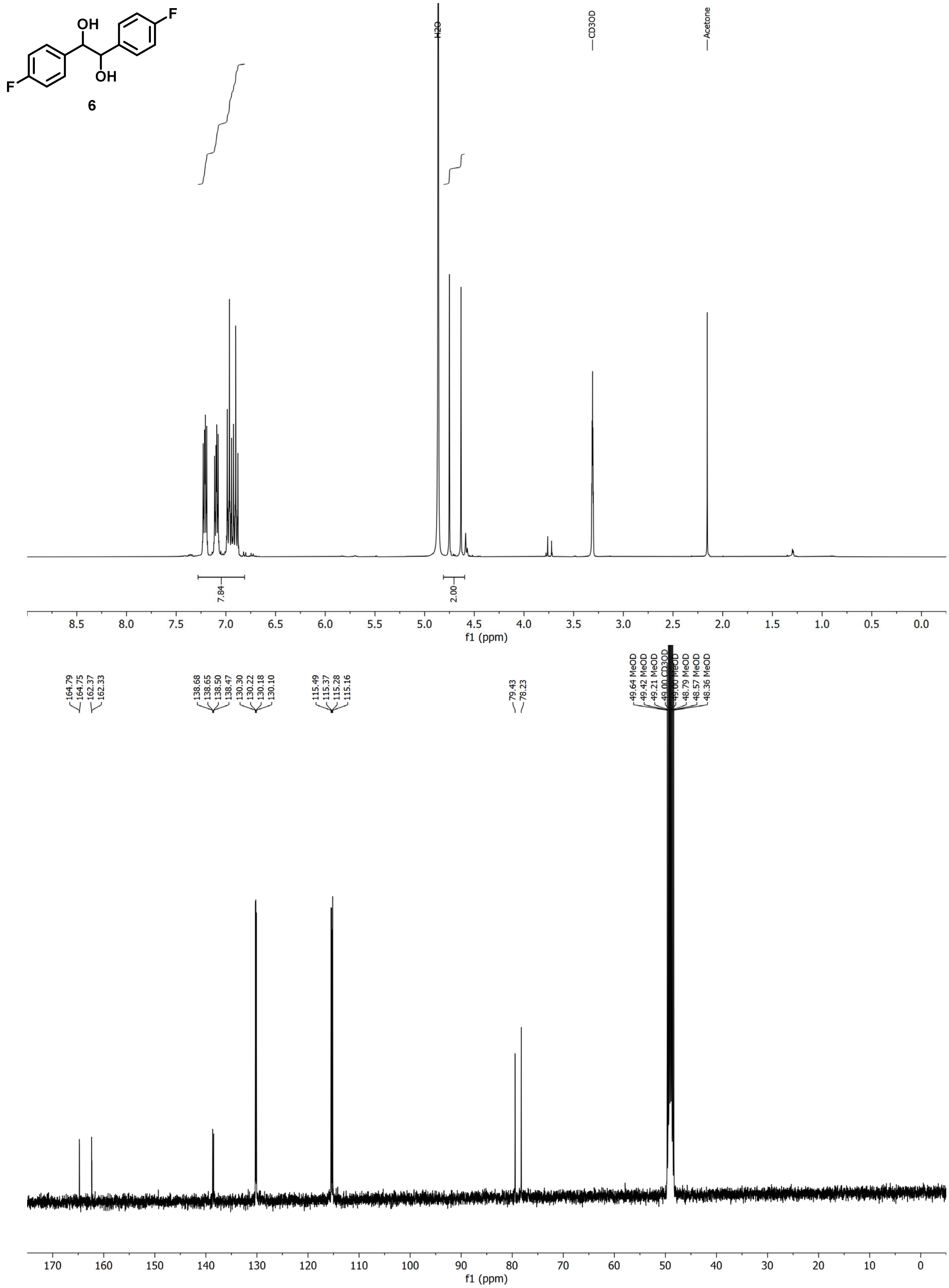

Figure S60. ^{1}H- and ^{13}C-NMR-spectra of diol **6** in CD_3OD (400 MHz/101 MHz).

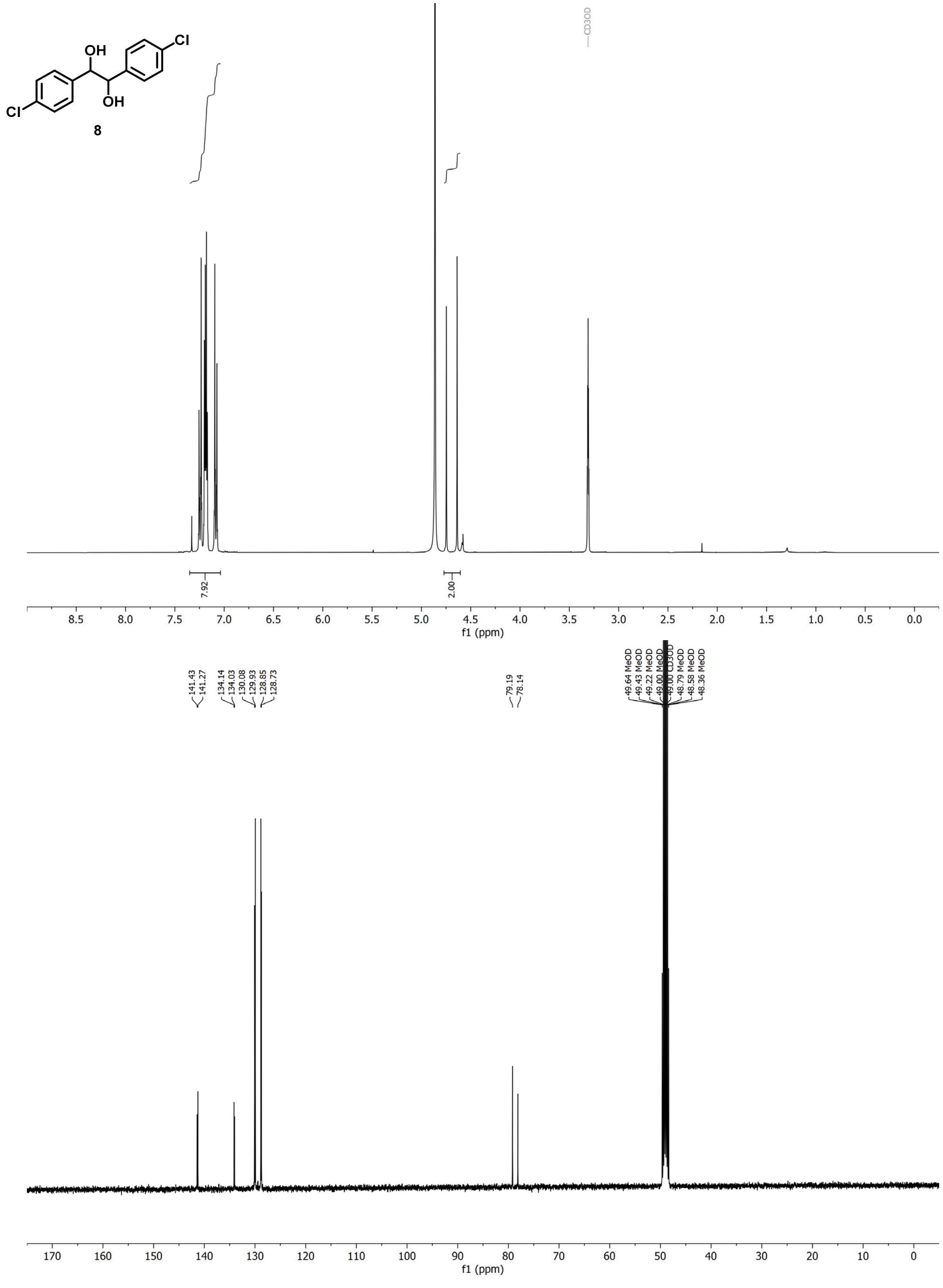

Figure S61. ^{1}H- and ^{13}C-NMR-spectra of diol **8** in CD_3OD (400 MHz/101 MHz).

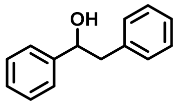

12

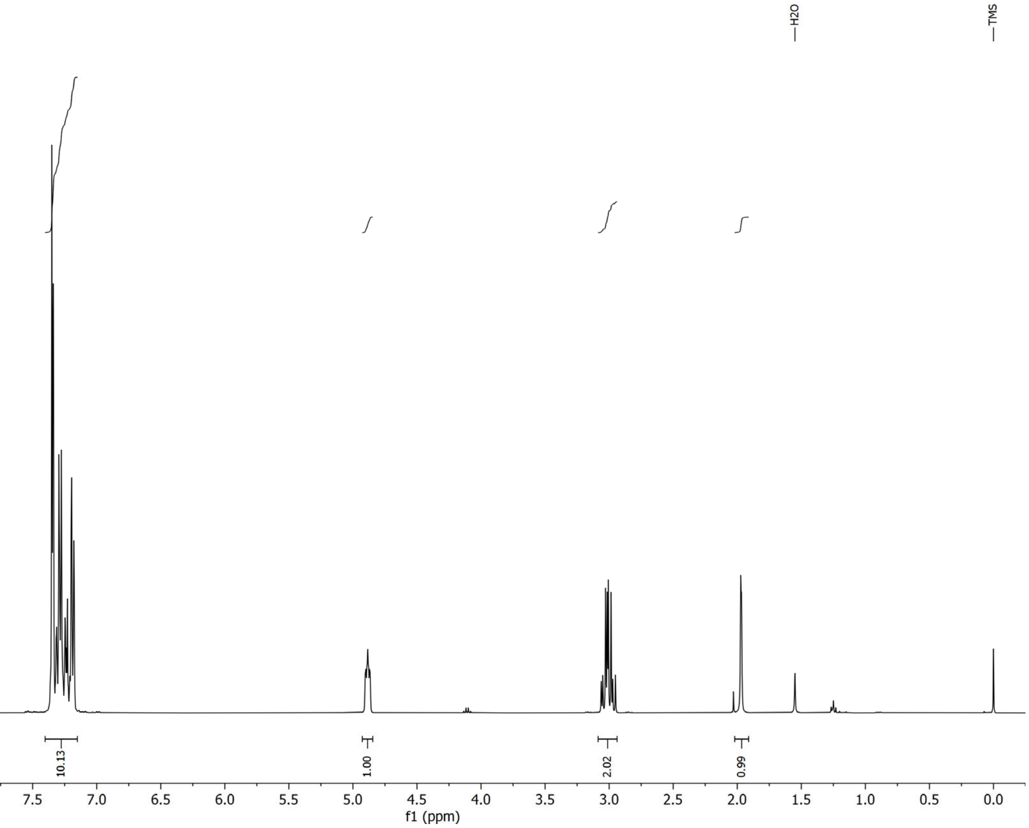

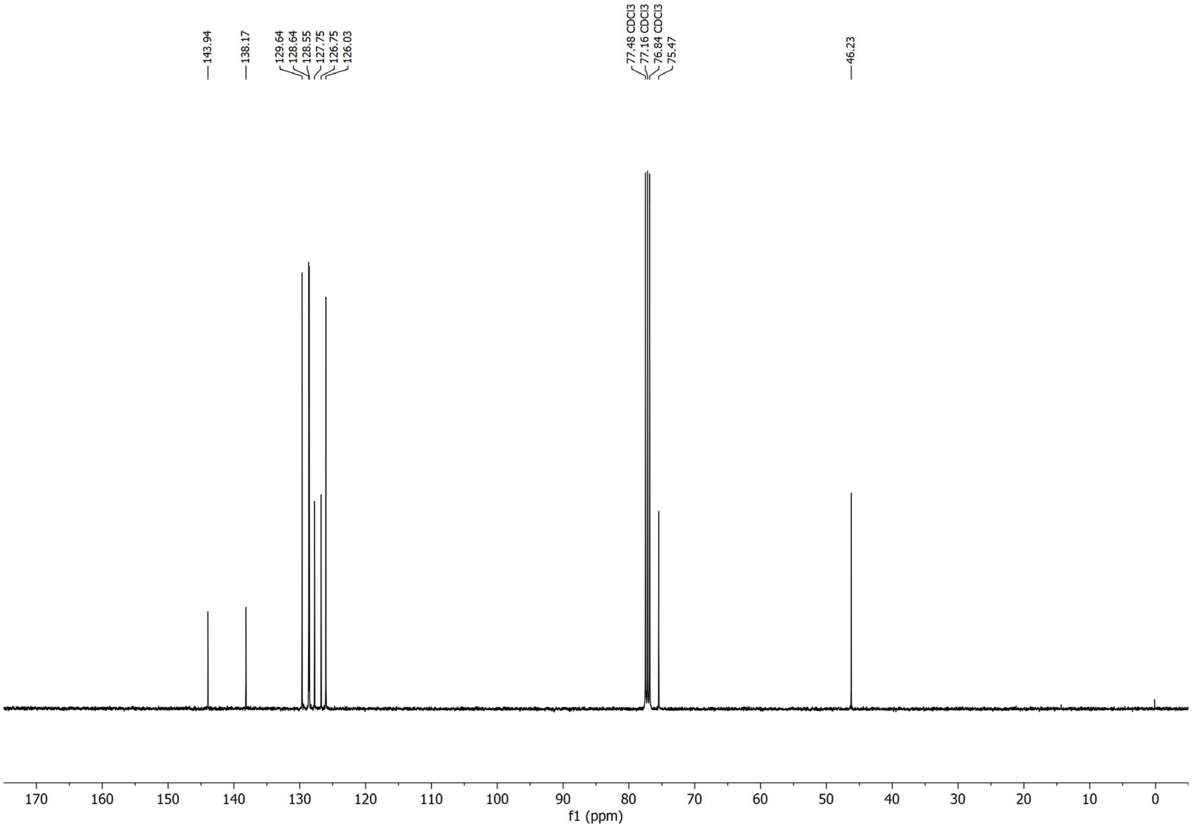

Figure S62. ^{1}H- and ^{13}C-NMR-spectra of alcohol **12** in $CDCl_3$ (400 MHz/101 MHz).

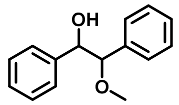

14

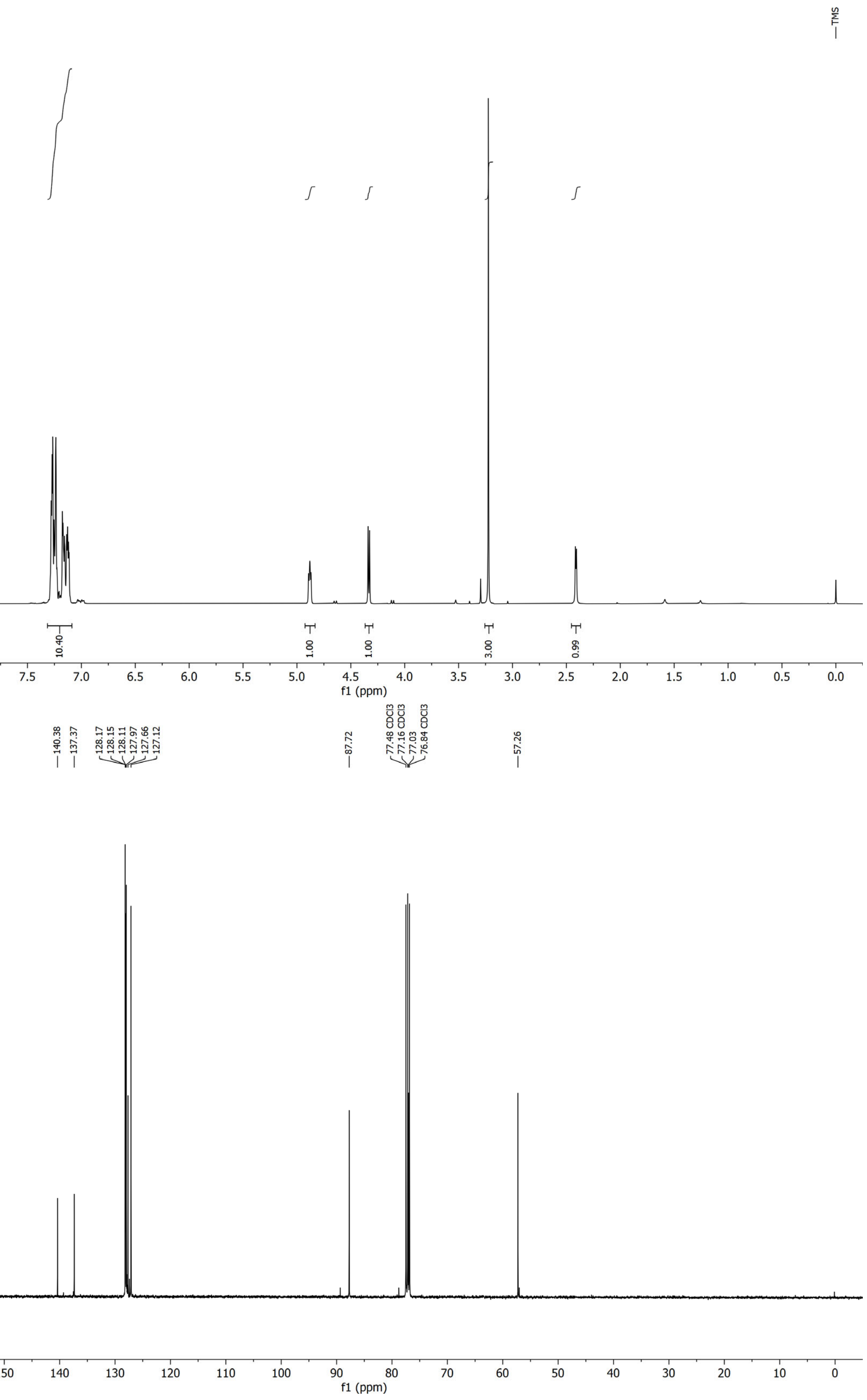

Figure S63. ^{1}H- and ^{13}C-NMR-spectra of alcohol **14** in $CDCl_3$ (400 MHz/101 MHz).

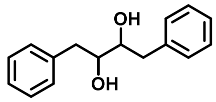

19

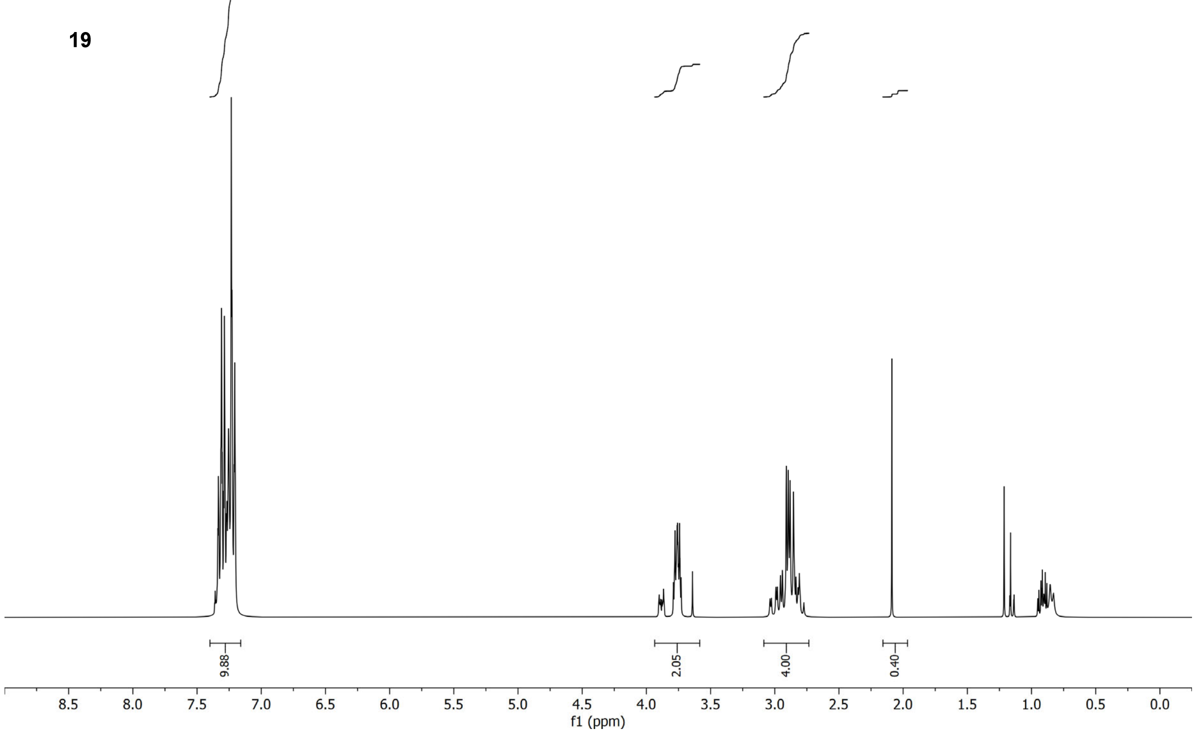

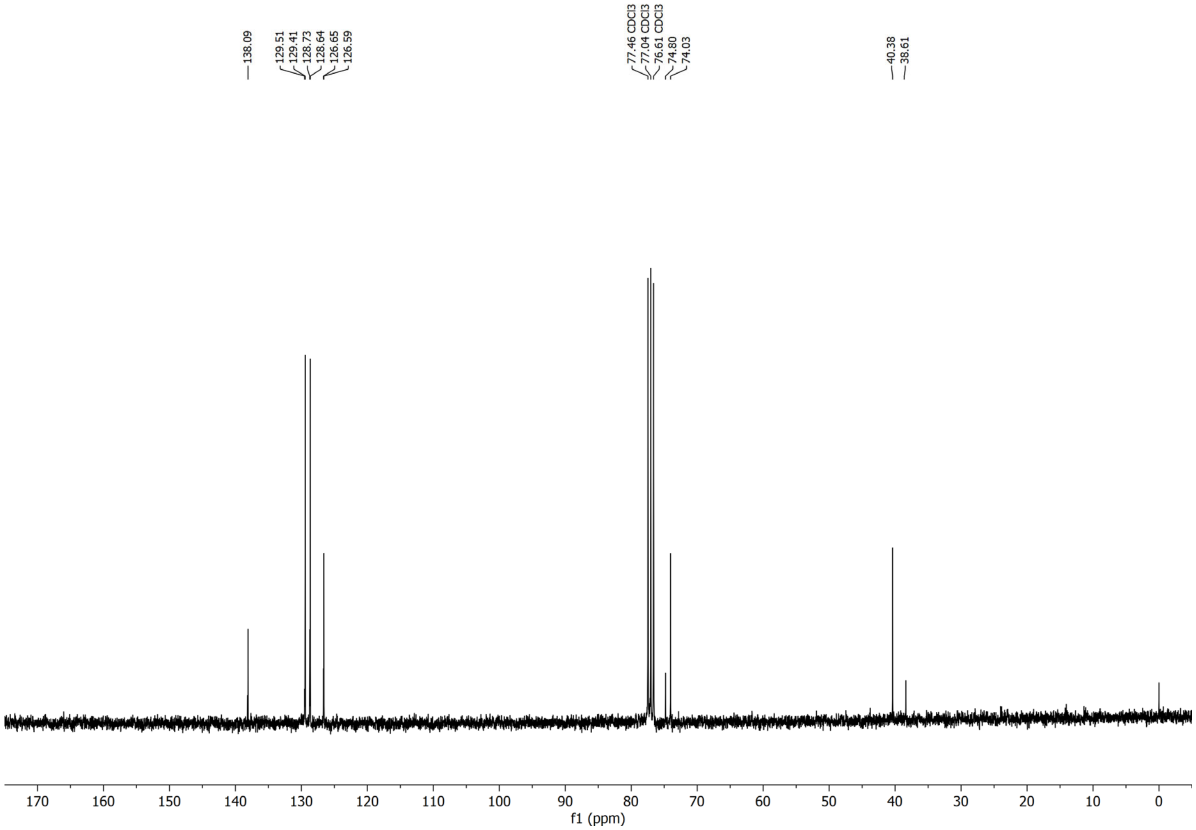

Figure S64. ^{1}H- and ^{13}C-NMR-spectra of diol **19** (mixture of stereoisomers) in $CDCl_3$ (300 MHz/75 MHz).

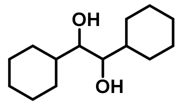

15

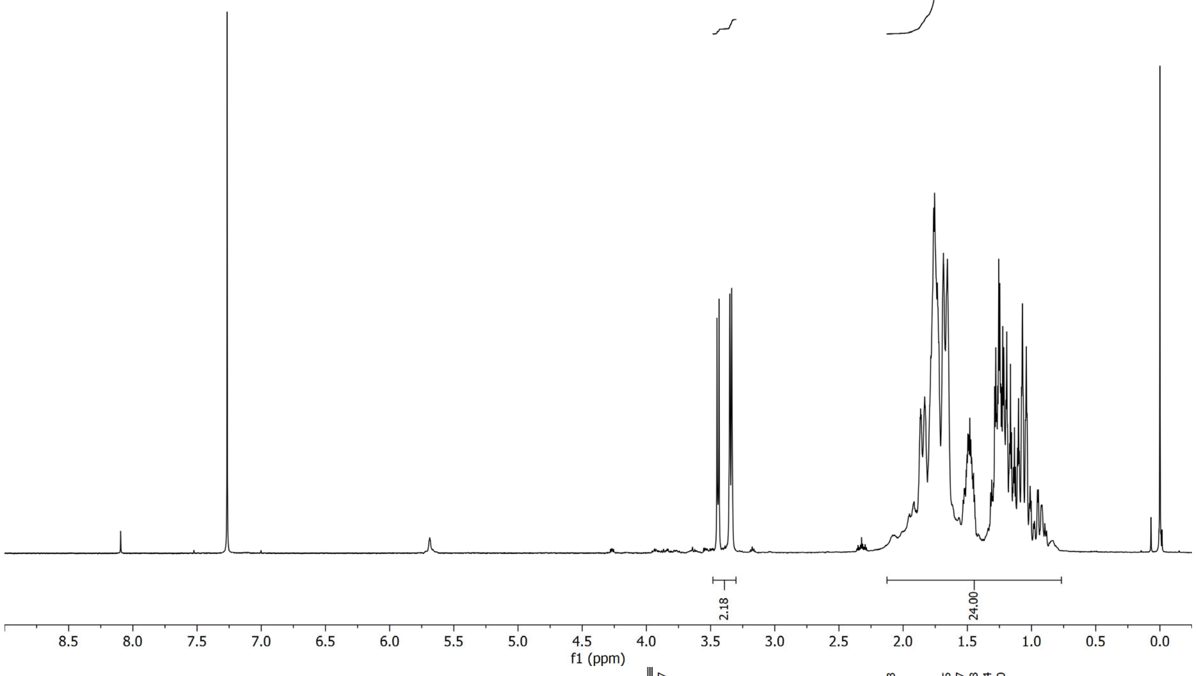

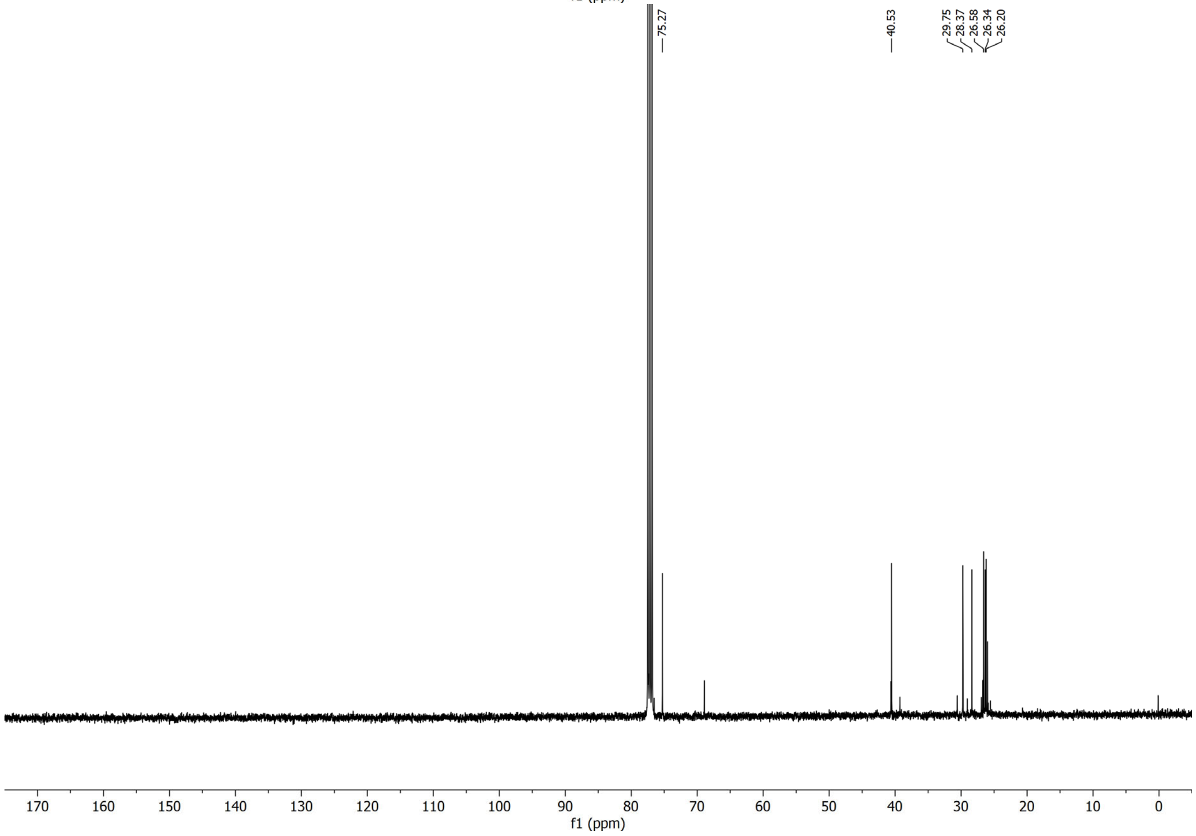

Figure S65. ^{1}H- and ^{13}C-NMR-spectra of diol **15** in $CDCl_3$ (400 MHz/101 MHz).

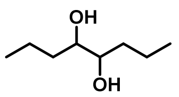

17

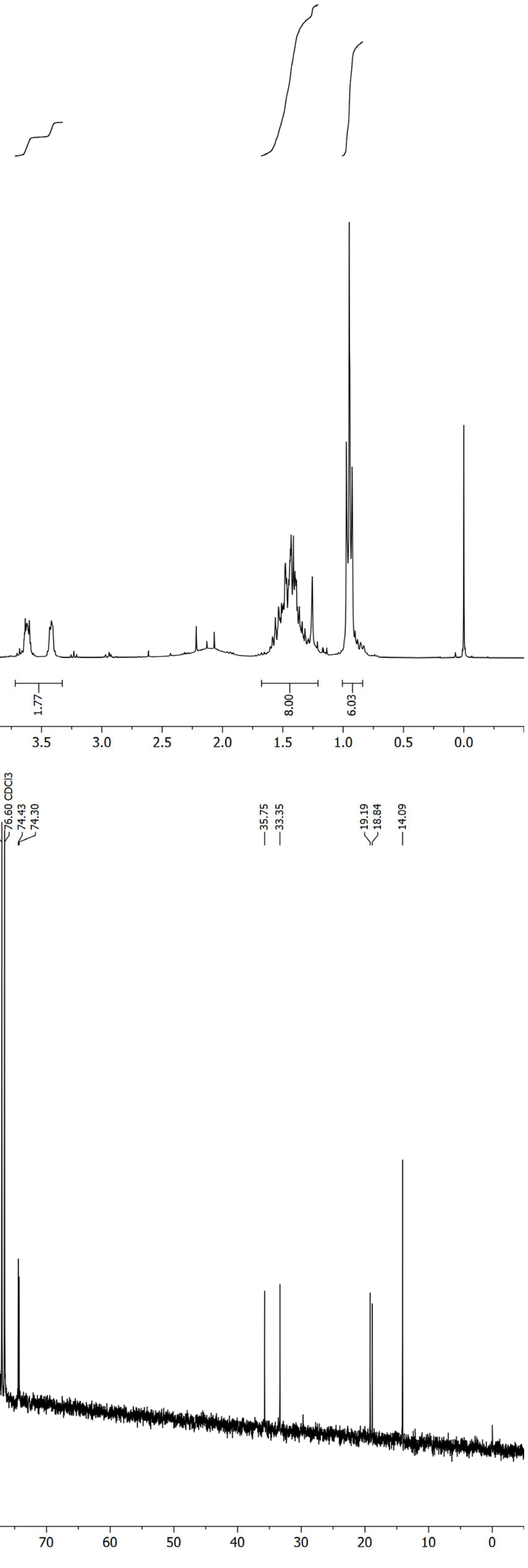

Figure S66. ^{1}H- and ^{13}C-NMR-spectra of diol **17** in $CDCl_3$ (300 MHz/75 MHz).

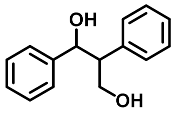

22

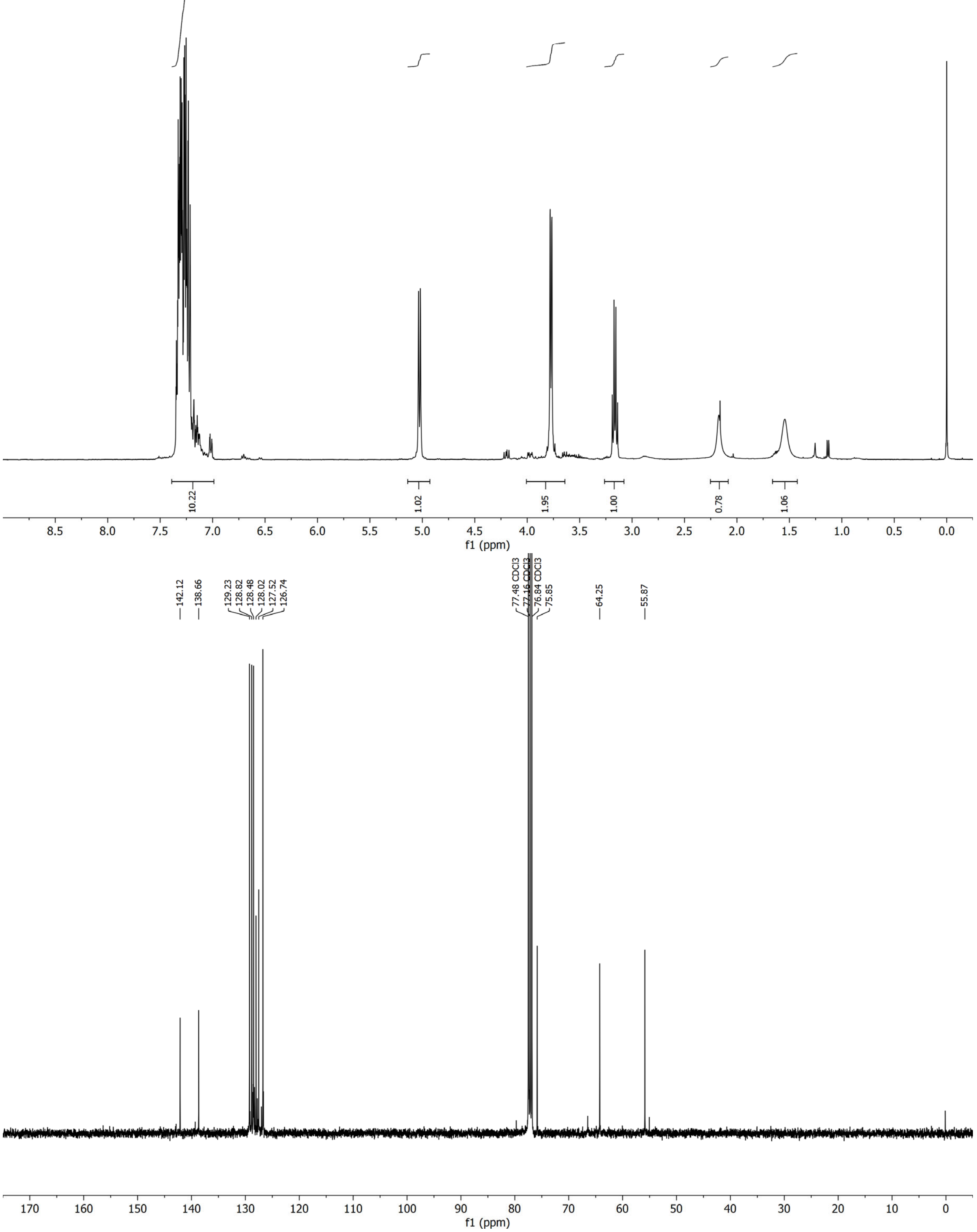

Figure S67. ^{1}H- and ^{13}C-NMR-spectra of lignin surrogate **22** in $CDCl_3$ (400 MHz/101 MHz).

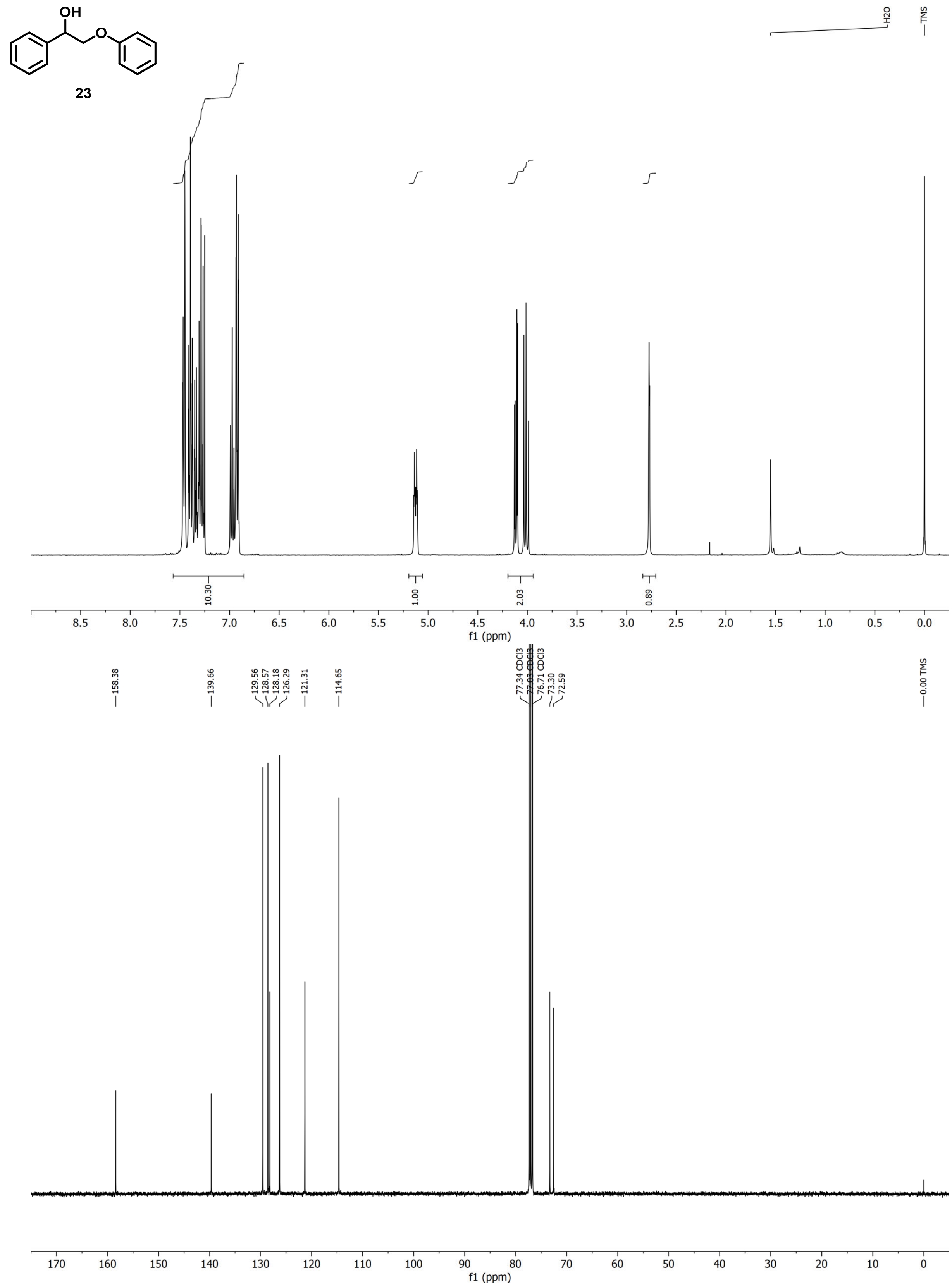

Figure S68. ^{1}H- and ^{13}C-NMR-spectra of lignin surrogate **23** in $CDCl_3$ (400 MHz/101 MHz).

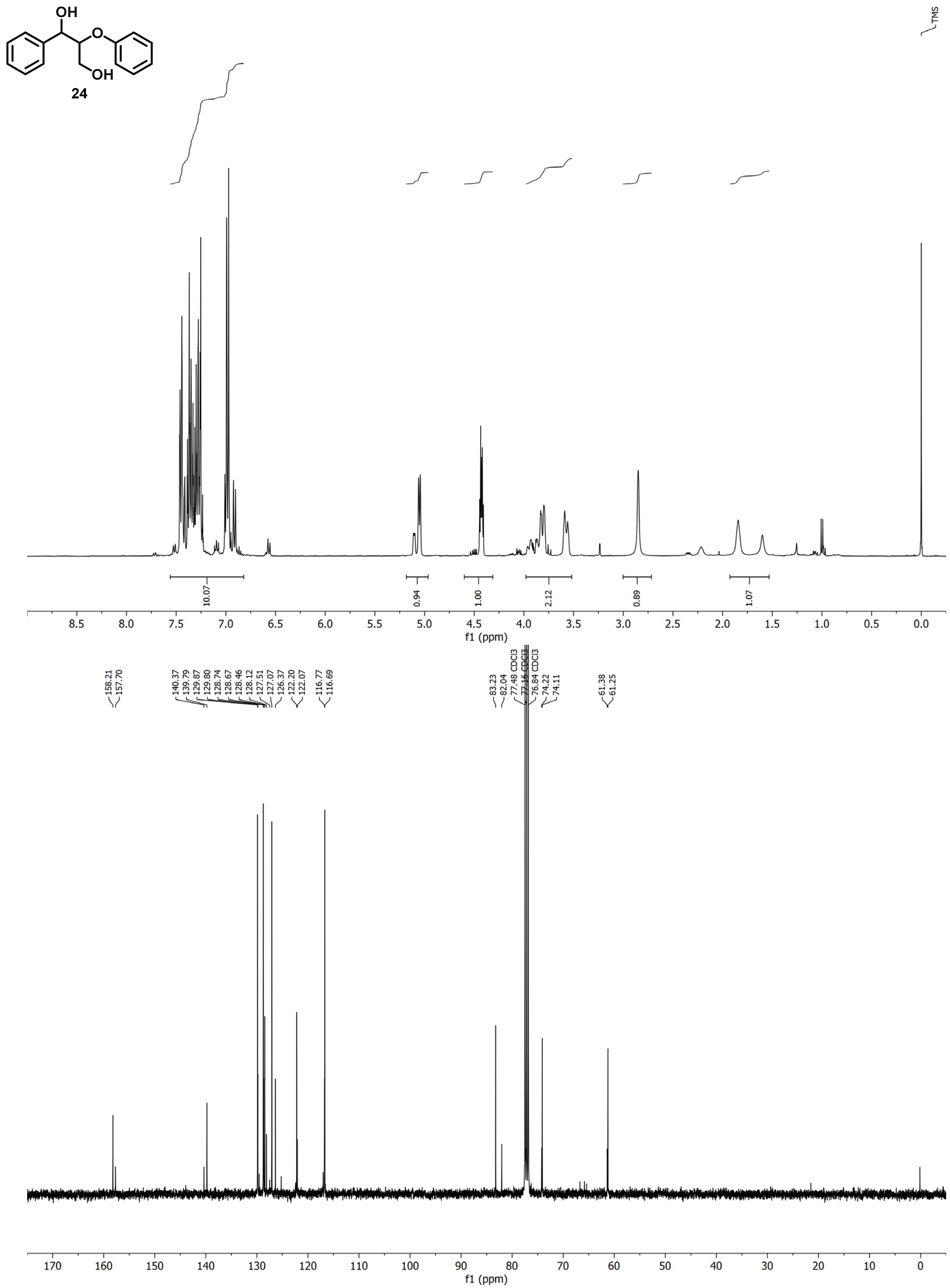

Figure S69. ^{1}H- and ^{13}C-NMR-spectra of lignin surrogate **24** in $CDCl_3$ (400 MHz/101 MHz).

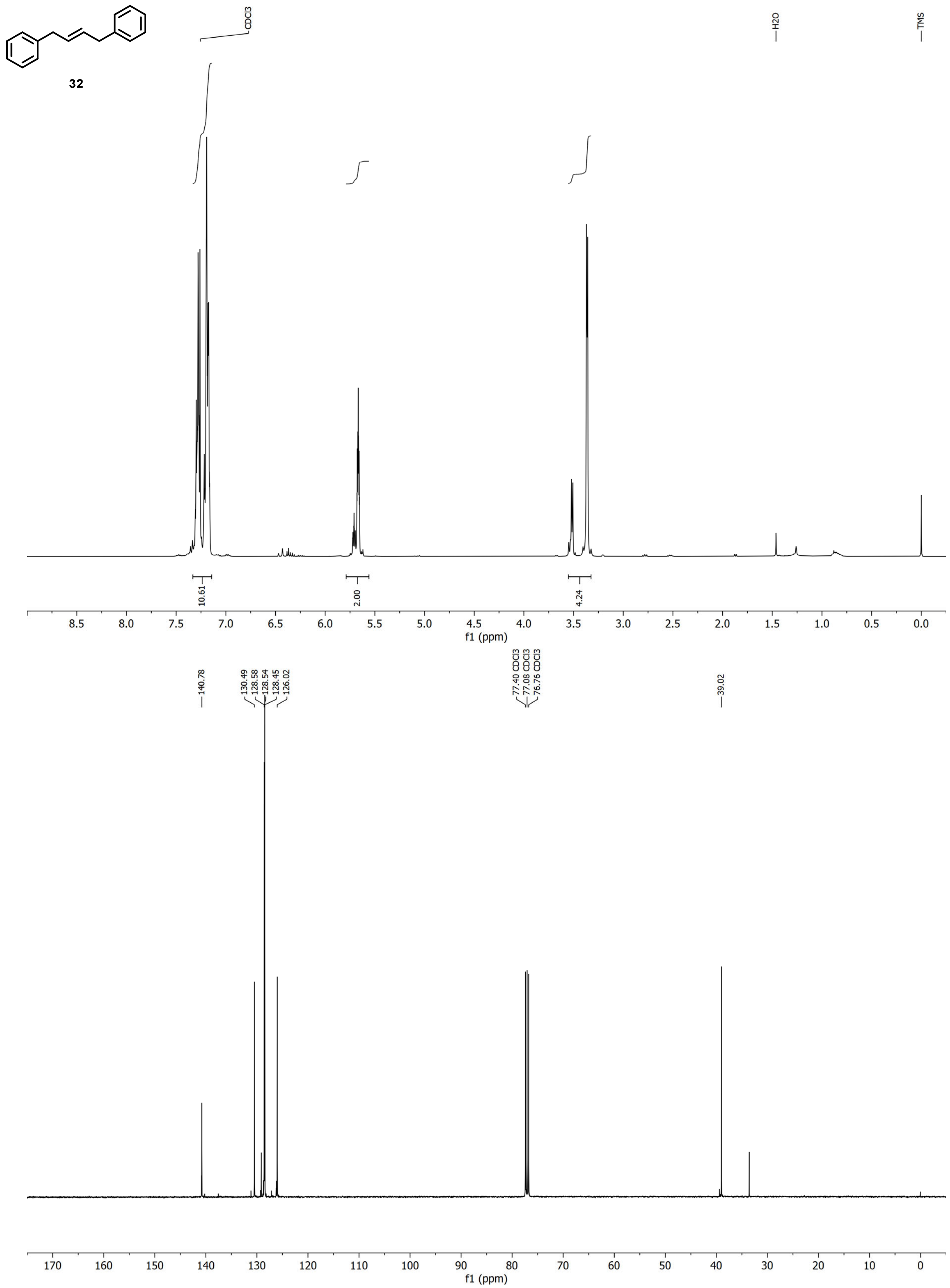

Figure S70. ^{1}H- and ^{13}C-NMR-spectra of (*E*)-1,4-Diphenylbut-2-ene (**32**) in $CDCl_3$ (400 MHz/101 MHz).

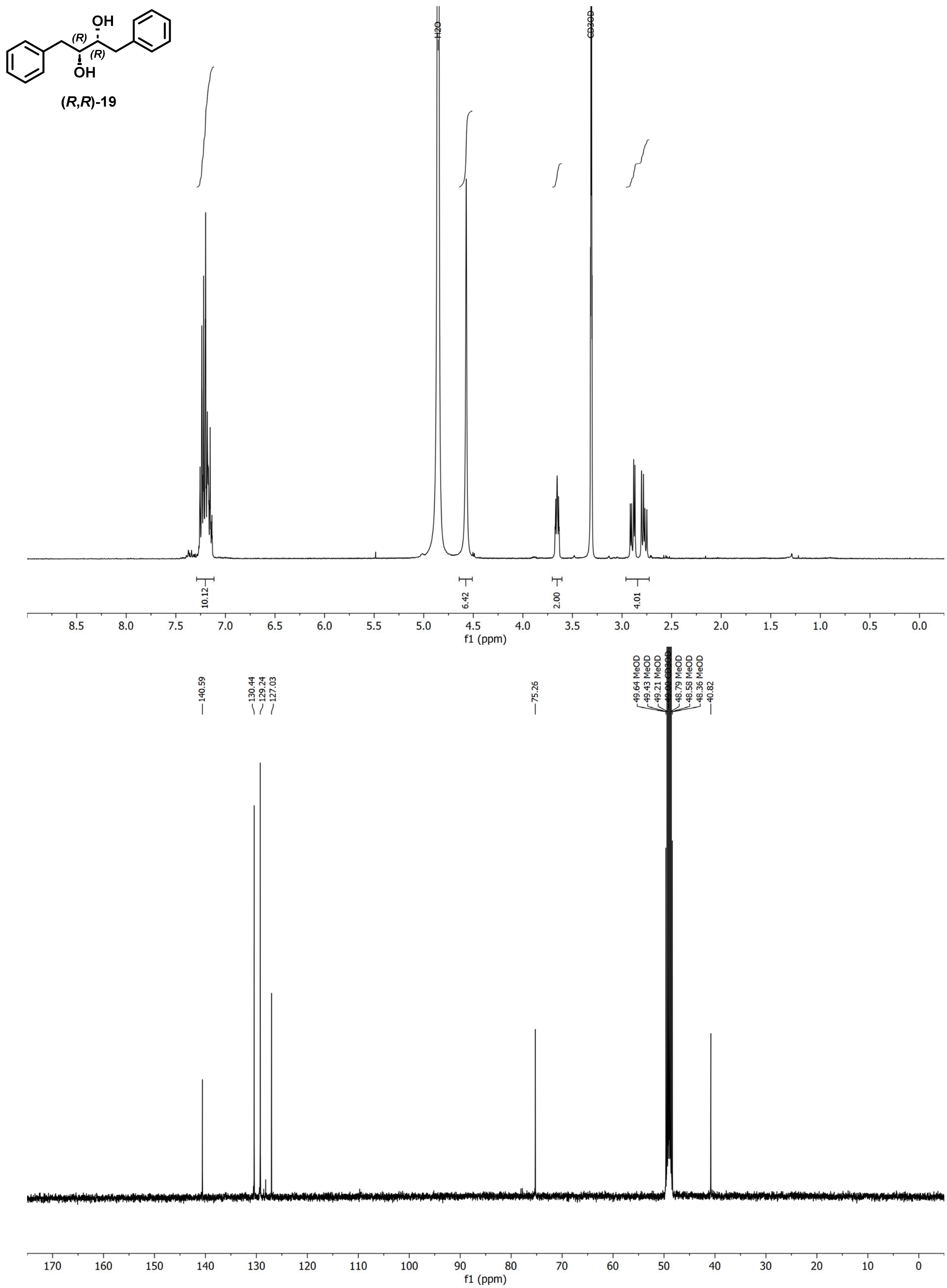

Figure S71. ^{1}H- and ^{13}C-NMR-spectra of diol ***(R,R)*-19** in CD_3OD (400 MHz/101 MHz).

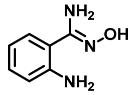

34

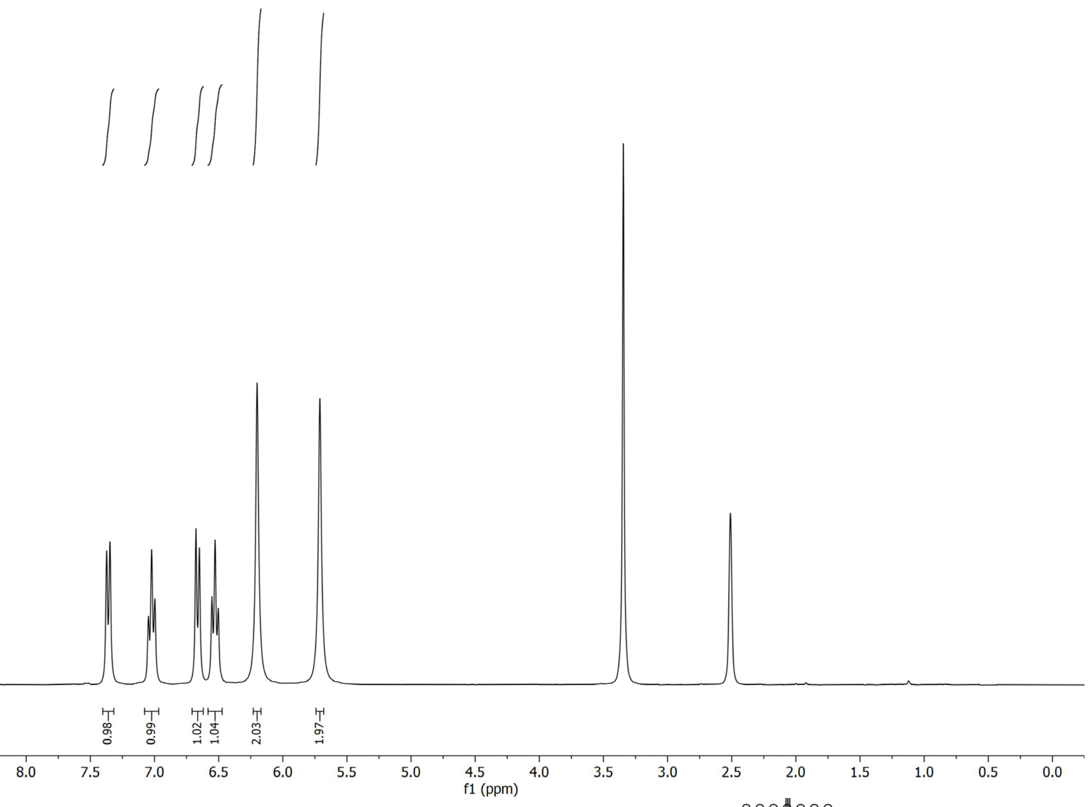

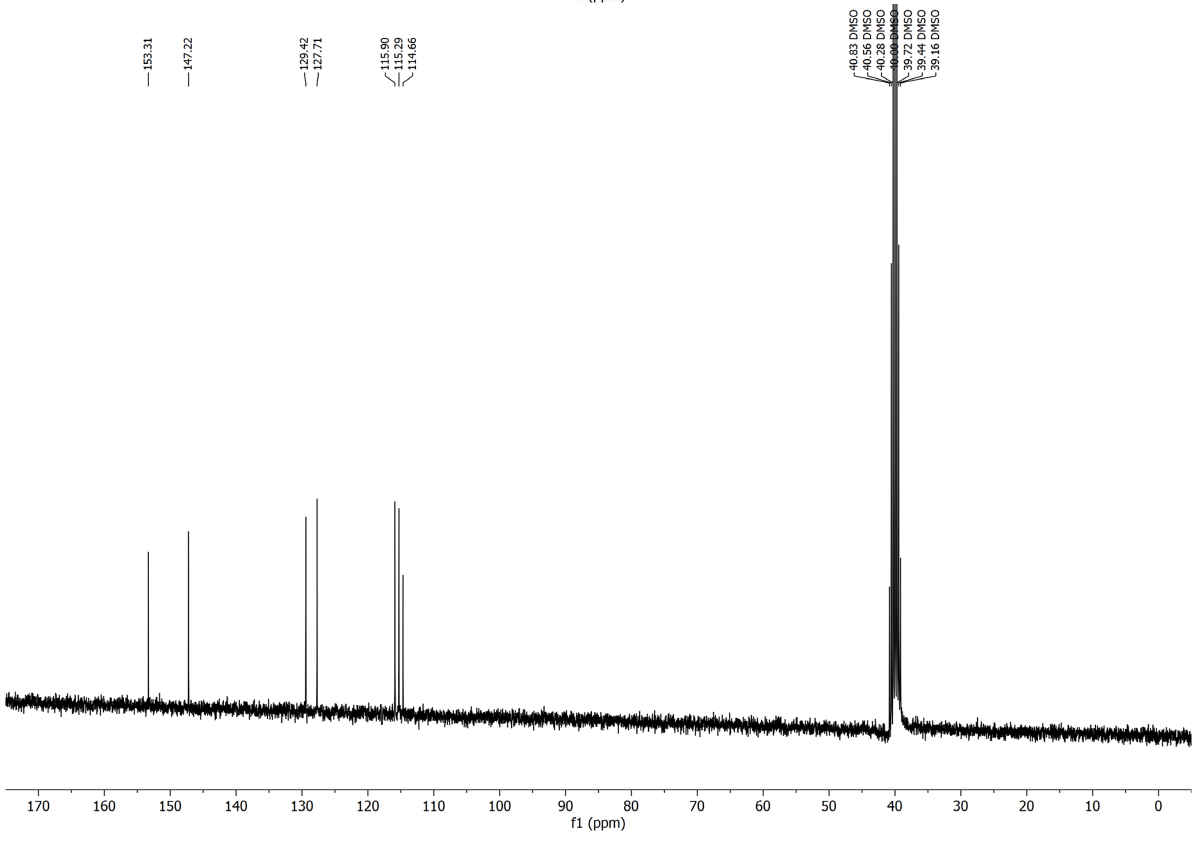

Figure S72. ^{1}H- and ^{13}C-NMR-spectra of ABAO (**34**) in DMSO-d6 (300 MHz/75 MHz).

S16 HPLC traces of all substrate and product reference compounds

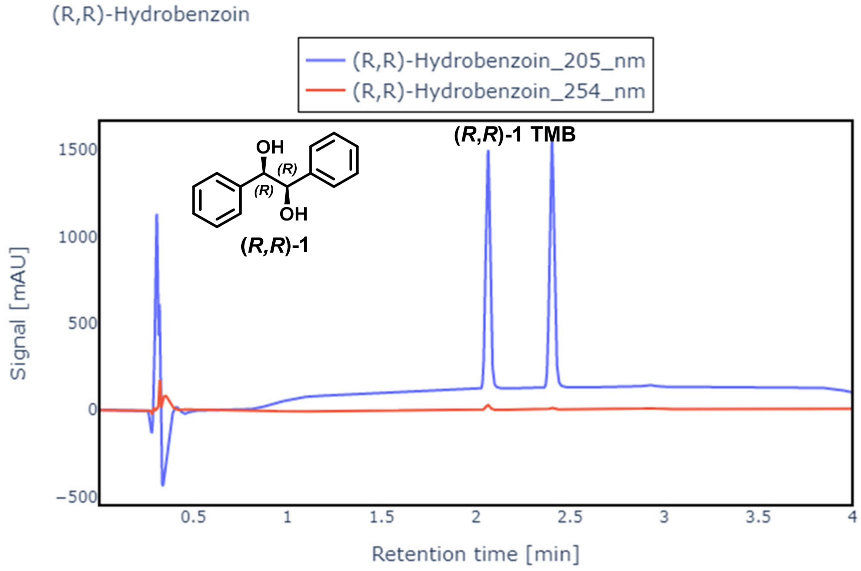

Compound	Retention time [min]
(*R*,*R*)-Hydrobenzoin [(***R*,*R***)-**1**]	2.065
1,3,5-Trimethoxybenzene (**TMB**)	2.404

Figure S73. HPLC chromatogram of (***R*,*R***)-**1**; Method 1 (achiral, Hypersil Gold C18).

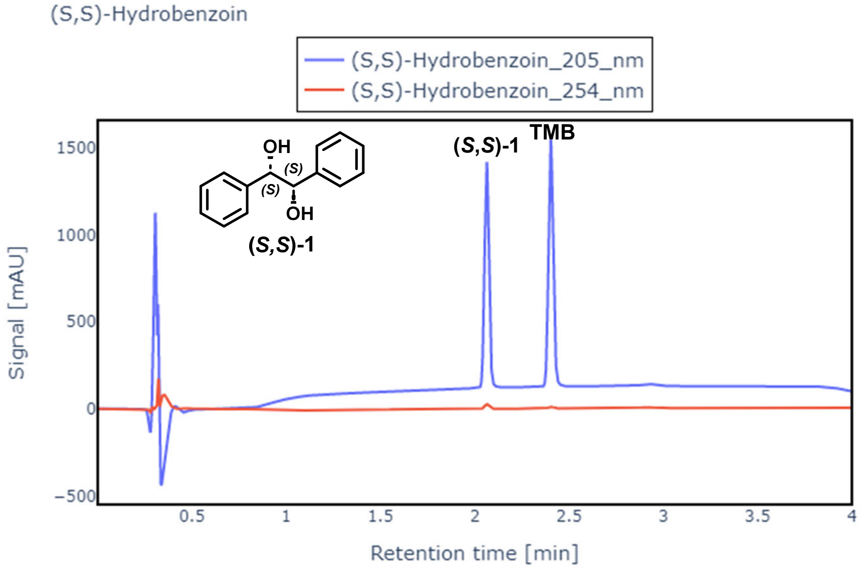

Compound	Retention time [min]
(*S*,*S*)-Hydrobenzoin [(***S*,*S***)-**1**]	2.065
1,3,5-Trimethoxybenzene (**TMB**)	2.403

Figure S74. HPLC chromatogram of (***S*,*S***)-**1**; Method 1 (achiral, Hypersil Gold C18).

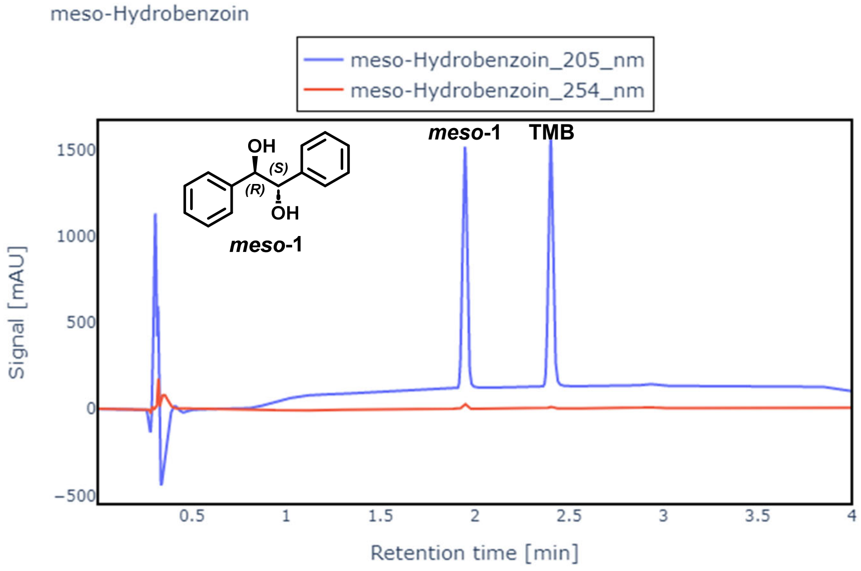

Compound	Retention time [min]
meso-Hydrobenzoin (***meso*-1**)	1.948
1,3,5-Trimethoxybenzene (**TMB**)	2.403

Figure S75. HPLC chromatogram of ***meso*-1**; Method 1 (achiral, Hypersil Gold C18).

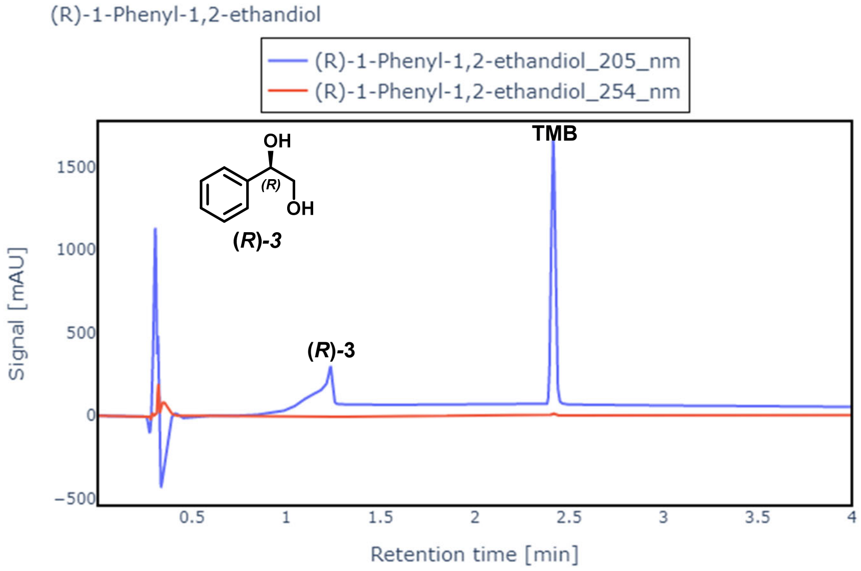

Compound	Retention time [min]
(*R*)-Phenyl-1,2-ethandiol [**(*R*)-3**]	1.235
1,3,5-Trimethoxybenzene (**TMB**)	2.416

Figure S76. HPLC chromatogram of **(*R*)-3**; Method 1 (achiral, Hypersil Gold C18).

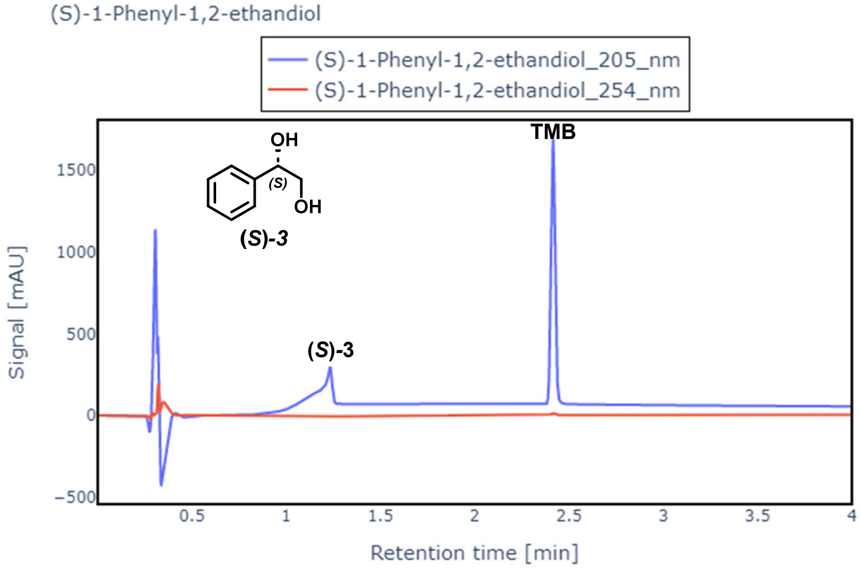

**Compound**	**Retention time [min]**
(*S*)-Phenyl-1,2-ethandiol [***(S)*-3**]	1.233
1,3,5-Trimethoxybenzene (**TMB**)	2.415

Figure S77. HPLC chromatogram of **(*S*)-3**; Method 1 (achiral, Hypersil Gold C18).

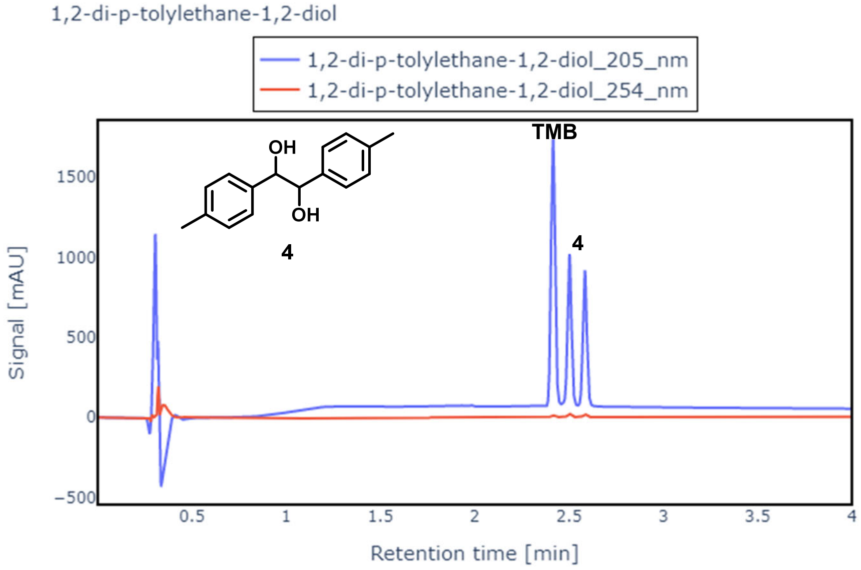

**Compound**	**Retention time [min]**
1,3,5-Trimethoxybenzene (**TMB**)	2.415
1,2-di-p-tolylethane-1,2-diol (**4**)	2.503+2.584

Figure S78. HPLC chromatogram of **4**; Method 1 (achiral, Hypersil Gold C18).

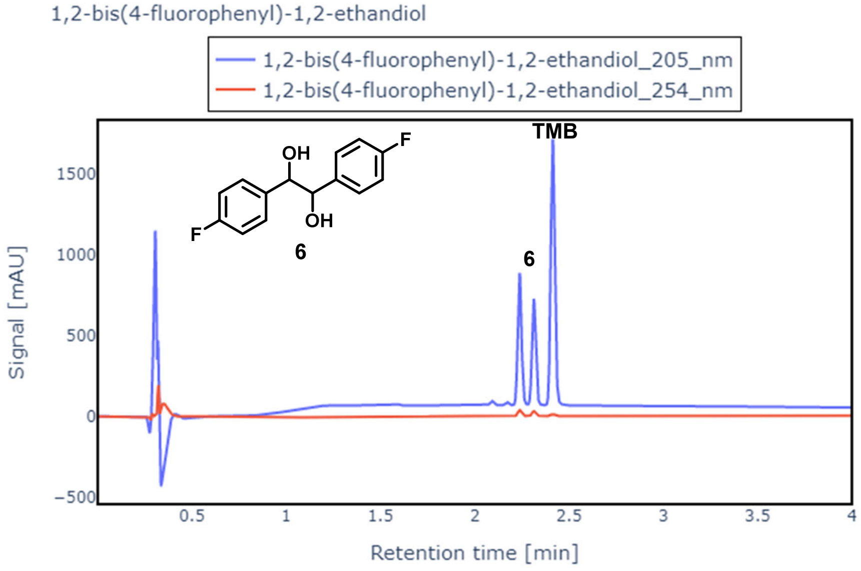

Compound	Retention time [min]
1,2-bis(4-Fluorophenyl)-1,2-ethandiol (**6**)	2.238+2.313
1,3,5-Trimethoxybenzene (**TMB**)	2.414

Figure S79. HPLC chromatogram of **6**; Method 1 (achiral, Hypersil Gold C18).

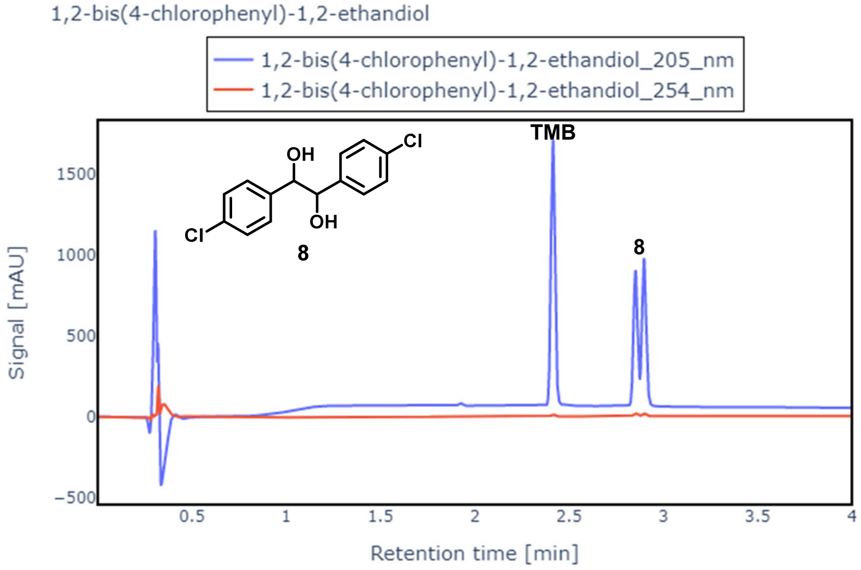

Compound	Retention time [min]
1,3,5-Trimethoxybenzene (**TMB**)	2.415
1,2-bis(4-Chlorophenyl)-1,2-ethandiol (**8**)	2.853+2.898

Figure S80. HPLC chromatogram of **8**; Method 1 (achiral, Hypersil Gold C18).

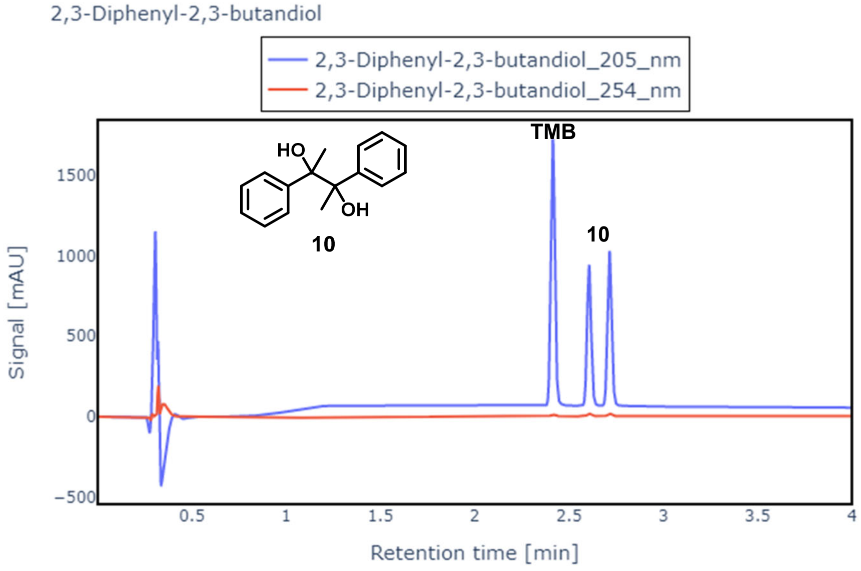

Compound	Retention time [min]
1,3,5-Trimethoxybenzene (**TMB**)	2.414
2,3-Diphenyl-2,3-butandiol (**10**)	2.607+2.715

Figure S81. HPLC chromatogram of **10**; Method 1 (achiral, Hypersil Gold C18).

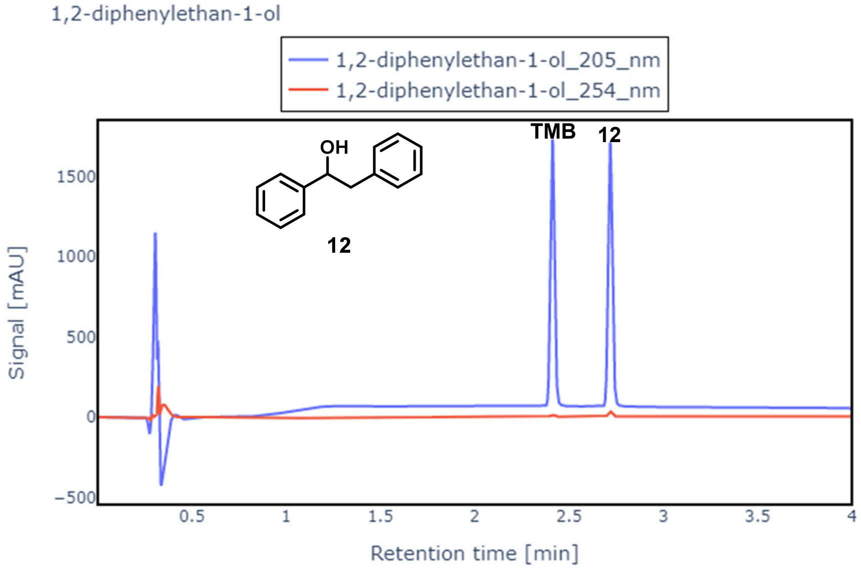

Compound	Retention time [min]
1,3,5-Trimethoxybenzene (**TMB**)	2.413
1,2-Diphenylethan-1-ol (**12**)	2.718

Figure S82. HPLC chromatogram of **12**; Method 1 (achiral, Hypersil Gold C18).

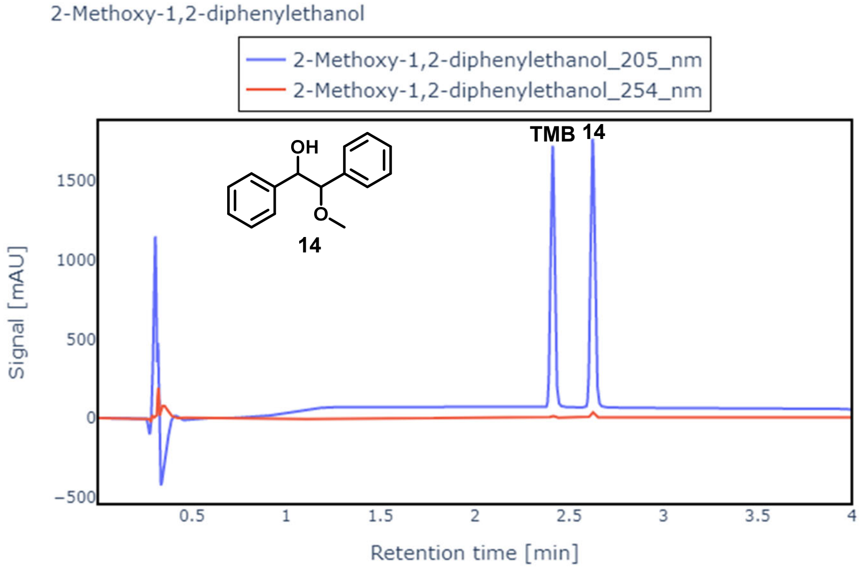

Compound	Retention time [min]
1,3,5-Trimethoxybenzene (**TMB**)	2.413
2-Methoxy-1,2-diphenylethanol (**14**)	2.625

Figure S83. HPLC chromatogram of **14**; Method 1 (achiral, Hypersil Gold C18).

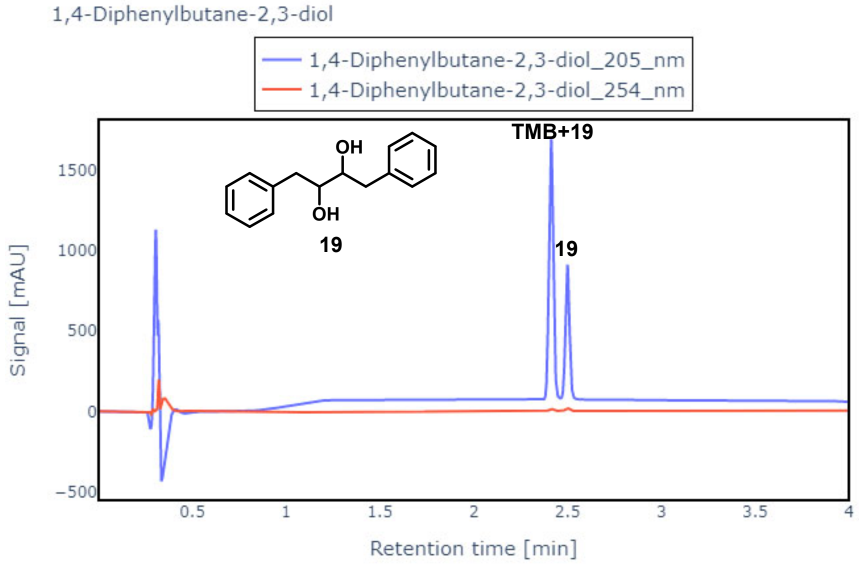

Compound	Retention time [min]
1,3,5-Trimethoxybenzene (**TMB**)	2.413
1,4-Diphenylbutane-2,3-diol (**19**)	2.413+2.500

Figure S84. HPLC chromatogram of **19**; Method 1 (achiral, Hypersil Gold C18).

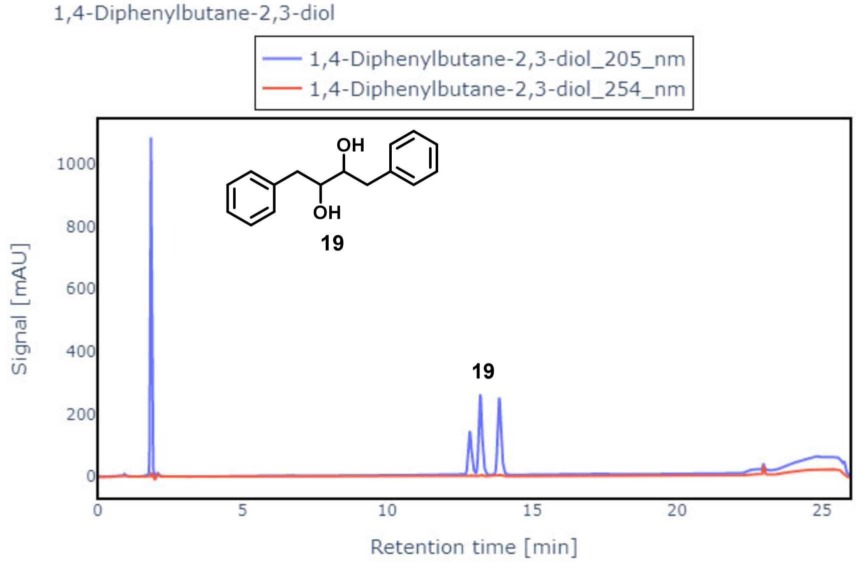

Compound	Retention time [min]
1,4-Diphenylbutane-2,3-diol [**19**: *meso*,(*S*,*S*),(*R*,*R*)]	12.848, 13.210, 13.863

Figure S85. HPLC chromatogram of **19**; Method 2 (chiral, Chiralcel OJ-RH).

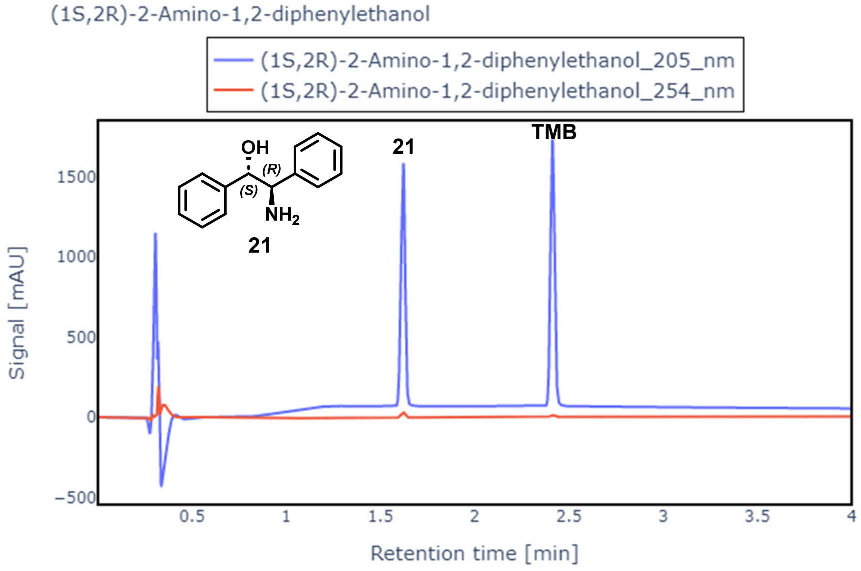

Compound	Retention time [min]
(1*S*,2*R*)-2-Amino-1,2-diphenylethan-1-ol (**21**)	1.622
1,3,5-Trimethoxybenzene (**TMB**)	2.413

Figure S86. HPLC chromatogram of **21**; Method 1 (achiral, Hypersil Gold C18).

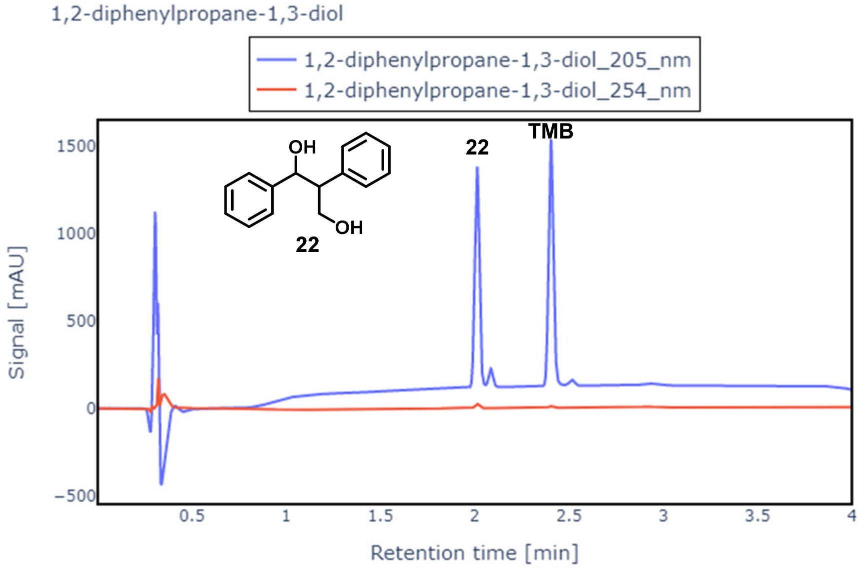

Compound	Retention time [min]
1,2-Diphenylpropane-1,3-diol (**22**)	2.013+2.086
1,3,5-Trimethoxybenzene (**TMB**)	2.404

Figure S87. HPLC chromatogram of **22**; Method 1 (achiral, Hypersil Gold C18).

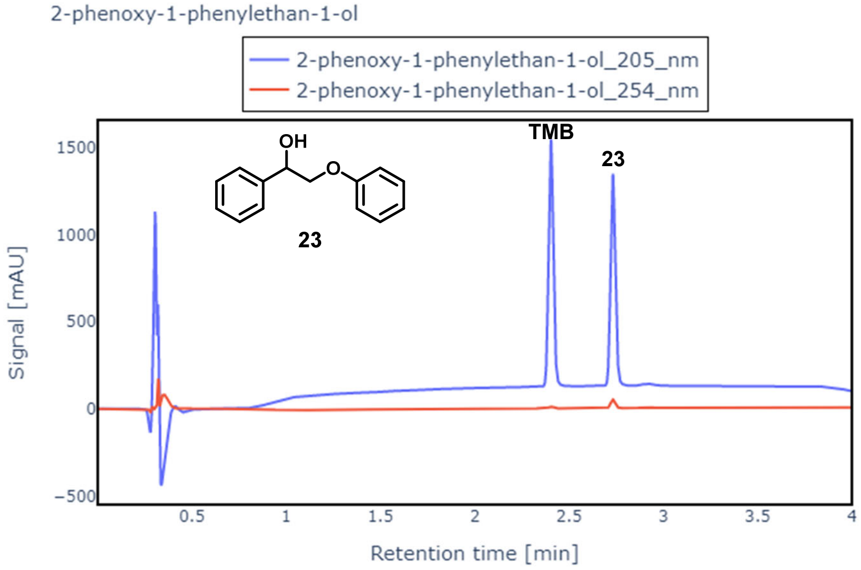

Compound	Retention time [min]
1,3,5-Trimethoxybenzene (**TMB**)	2.404
2-Phenoxy-1-1phenylethan-1-ol (**23**)	2.733

Figure S88. HPLC chromatogram of **23**; Method 1 (achiral, Hypersil Gold C18).

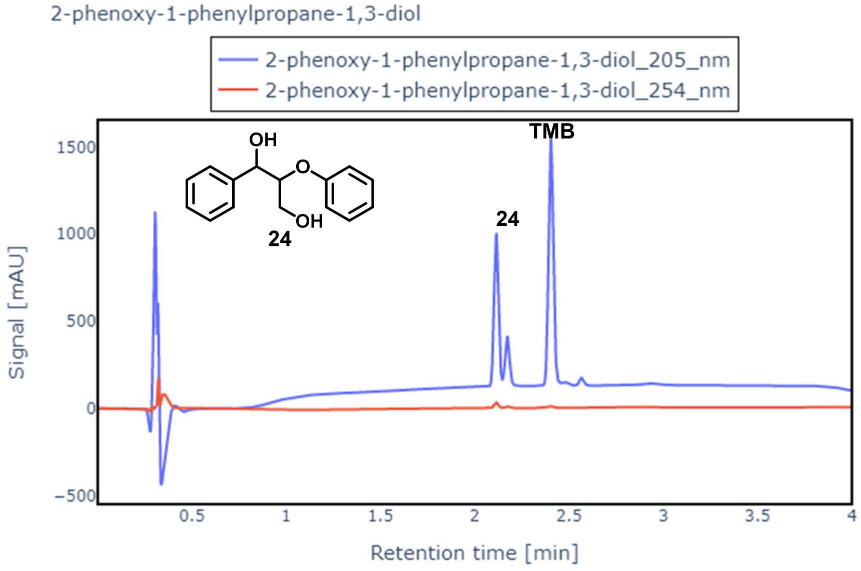

Compound	Retention time [min]
2-Phenoxy-1-phenylpropane-1,3-diol (**24**)	2.114+2.173
1,3,5-Trimethoxybenzene (**TMB**)	2.403

Figure S89. HPLC chromatogram of **24**; Method 1 (achiral, Hypersil Gold C18).

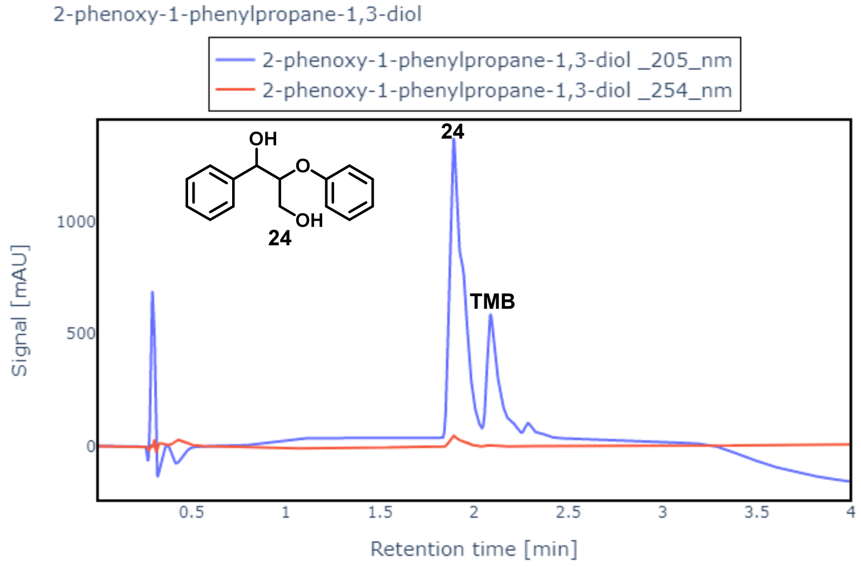

Compound	Retention time [min]
2-Phenoxy-1-phenylpropane-1,3-diol (**24**)	1.892
1,3,5-Trimethoxybenzene (**TMB**)	2.088

Figure S90. HPLC chromatogram of **24**; Method 1 (achiral, Hypersil Gold C18_2020).

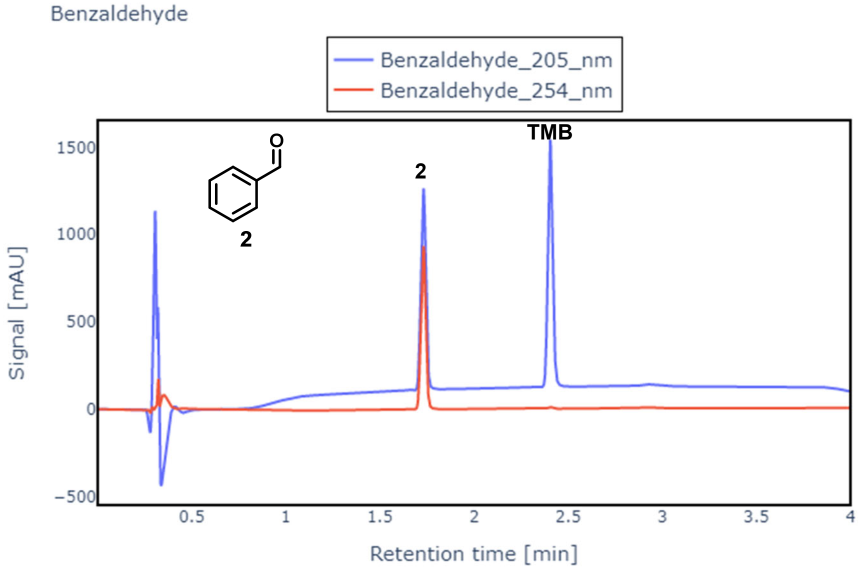

**Compound**	**Retention time [min]**
Benzaldehyde (**2**)	1.732
1,3,5-Trimethoxybenzene (**TMB**)	2.405

Figure S91. HPLC chromatogram of **2**; Method 1 (achiral, Hypersil Gold C18).

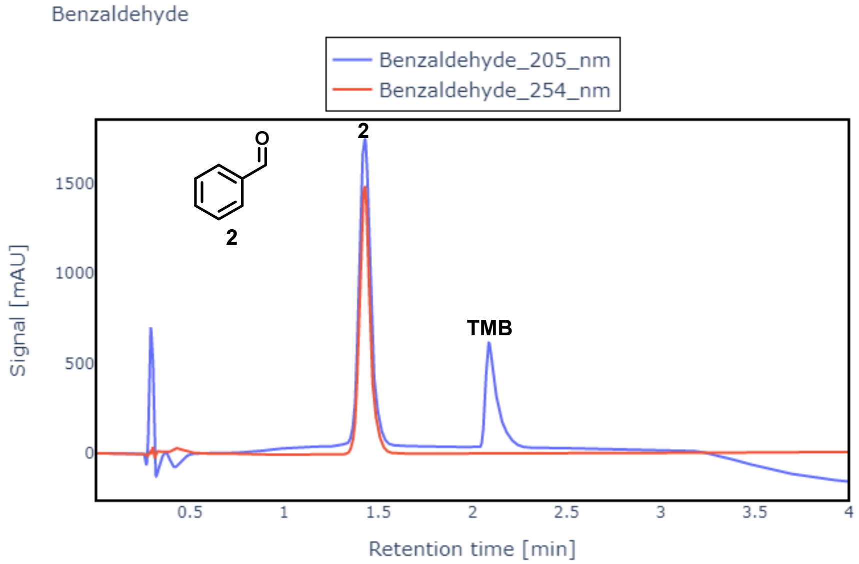

**Compound**	**Retention time [min]**
Benzaldehyde (**2**)	1.429
1,3,5-Trimethoxybenzene (**TMB**)	2.088

Figure S92. HPLC chromatogram of **2**; Method 1 (achiral, Hypersil Gold C18_2020).

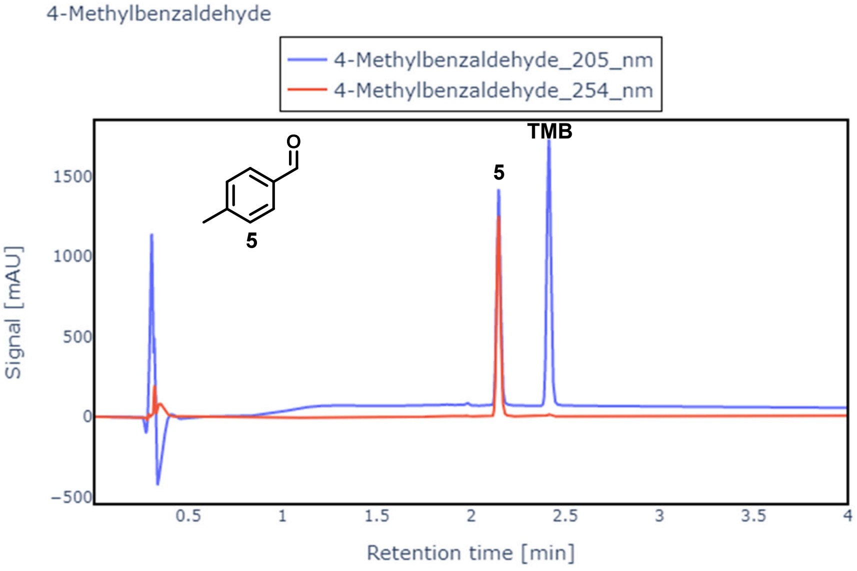

Compound	Retention time [min]
4-Methylbenzaldehyde (**5**)	2.147
1,3,5-Trimethoxybenzene (**TMB**)	2.413

Figure S93. HPLC chromatogram of **5**; Method 1 (achiral, Hypersil Gold C18).

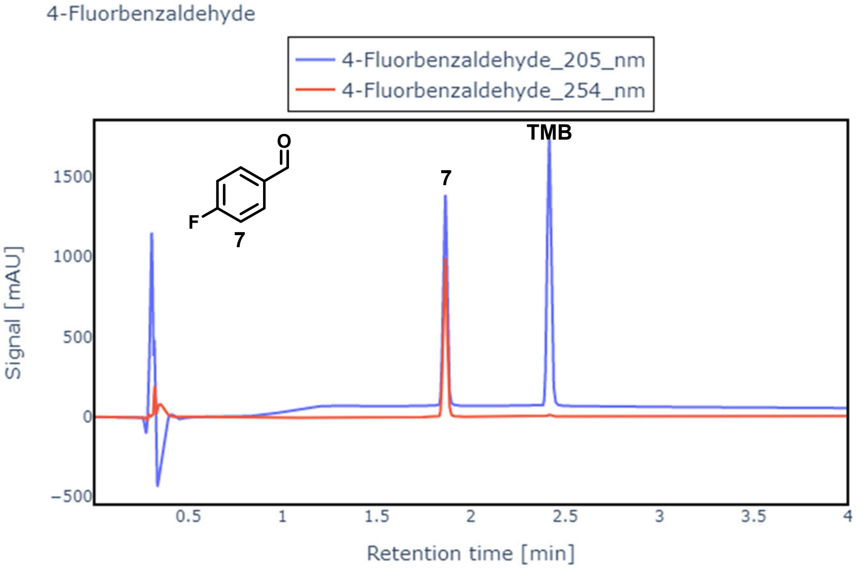

Compound	Retention time [min]
4-Flourobenzaldehyde (**7**)	1.864
1,3,5-Trimethoxybenzene (**TMB**)	2.415

Figure S94. HPLC chromatogram of **7**; Method 1 (achiral, Hypersil Gold C18).

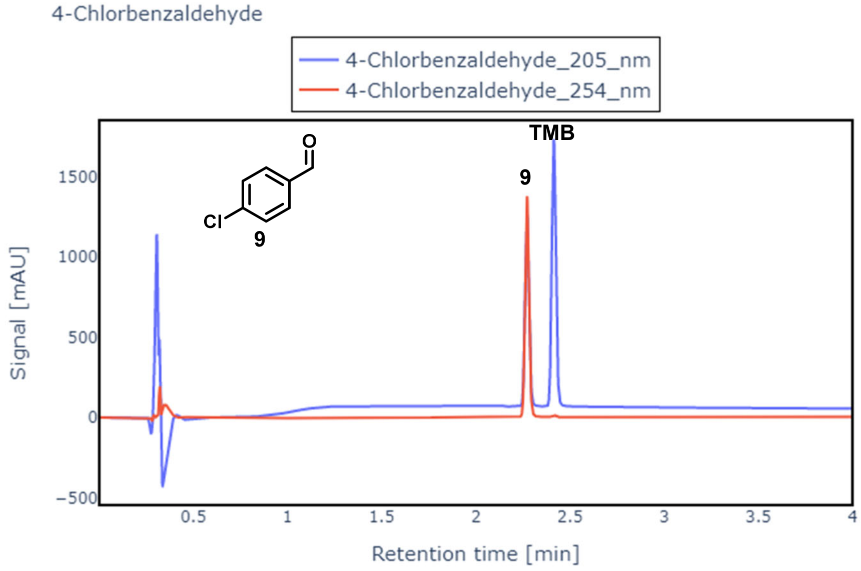

Compound	Retention time [min]
4-Chlorobenzaldehyde (**9**)	2.273
1,3,5-Trimethoxybenzene (**TMB**)	2.414

Figure S95. HPLC chromatogram of **9**; Method 1 (achiral, Hypersil Gold C18).

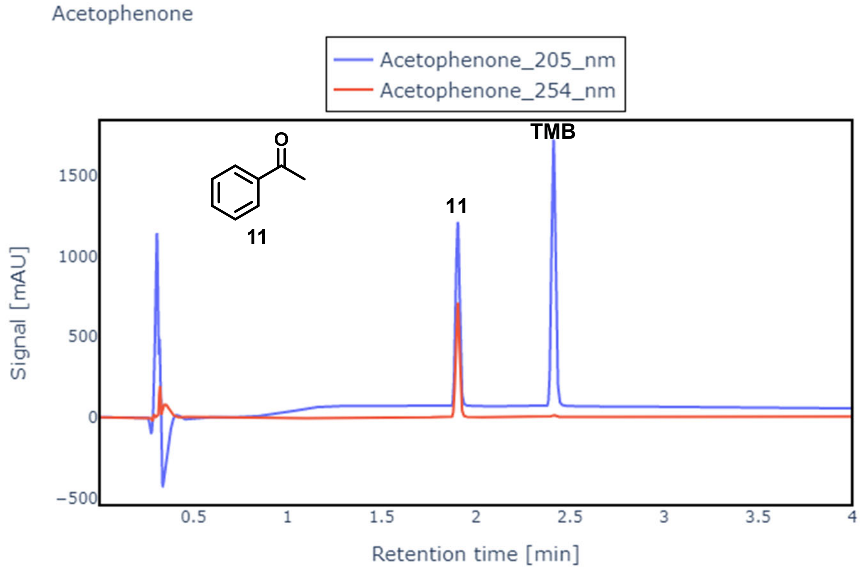

Compound	Retention time [min]
Acetophenone (**11**)	1.904
1,3,5-Trimethoxybenzene (**TMB**)	2.413

Figure S96. HPLC chromatogram of **11**; Method 1 (achiral, Hypersil Gold C18).

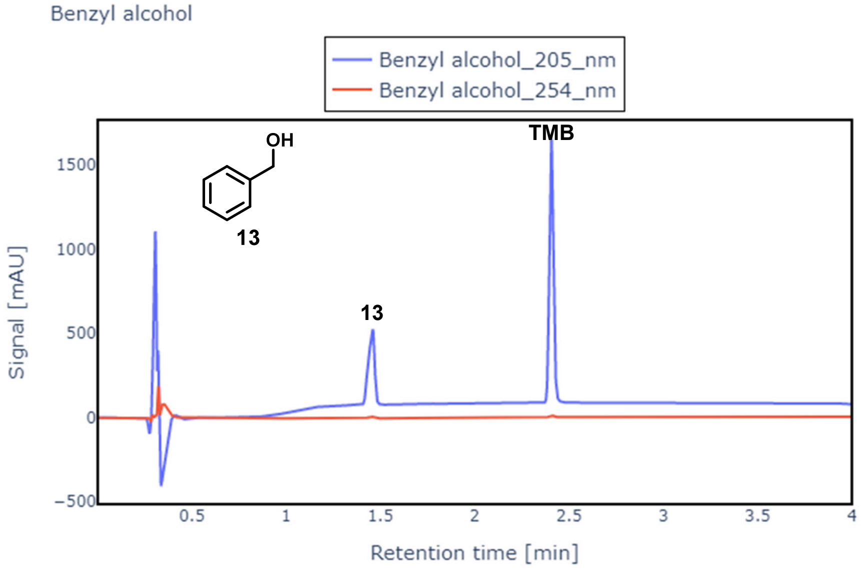

Compound	Retention time [min]
Benzyl alcohol (**13**)	1.459
1,3,5-Trimethoxybenzene (**TMB**)	2.407

Figure S97. HPLC chromatogram of **13**; Method 1 (achiral, Hypersil Gold C18).

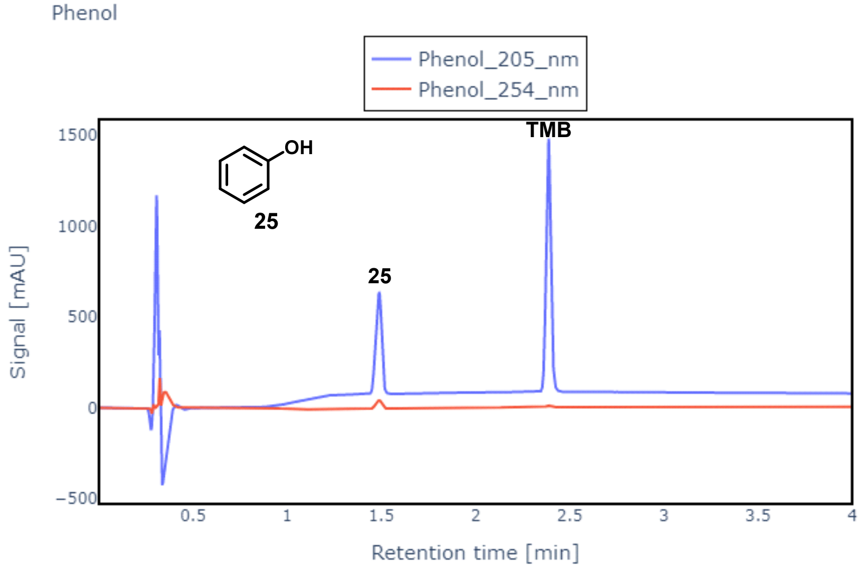

Compound	Retention time [min]
Phenol (**25**)	1.489
1,3,5-Trimethoxybenzene (**TMB**)	2.388

Figure S98. HPLC chromatogram of **25**; Method 1 (achiral, Hypersil Gold C18).

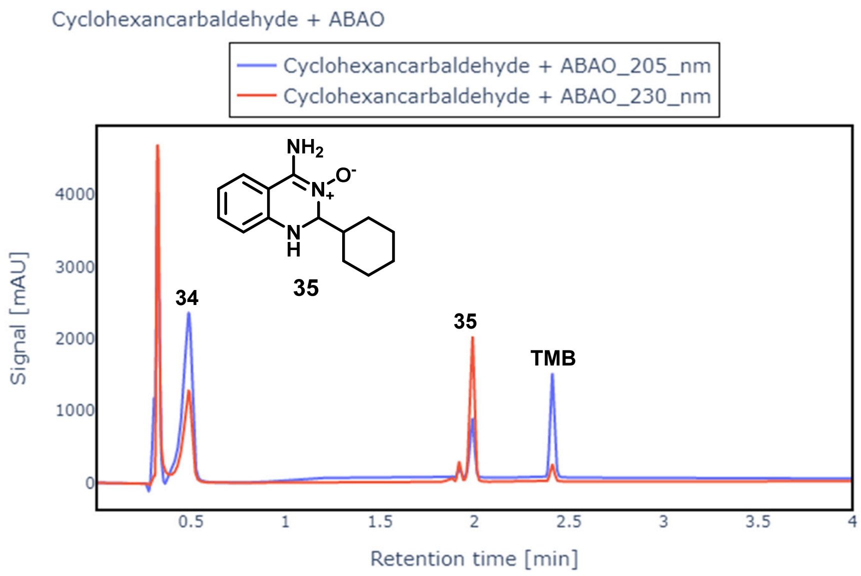

Compound	Retention time [min]
Unreacted ABAO (**34**)	0.488
Cyclohexancarbaldehyde-ABAO (4-amino-2-cyclohexyl-1,2-dihydroquinazoline-3-oxide, **35**)	1.989
1,3,5-Trimethoxybenzene (**TMB**)	2.412

Figure S99. HPLC chromatogram of **35**; Method 1 (achiral, Hypersil Gold C18).

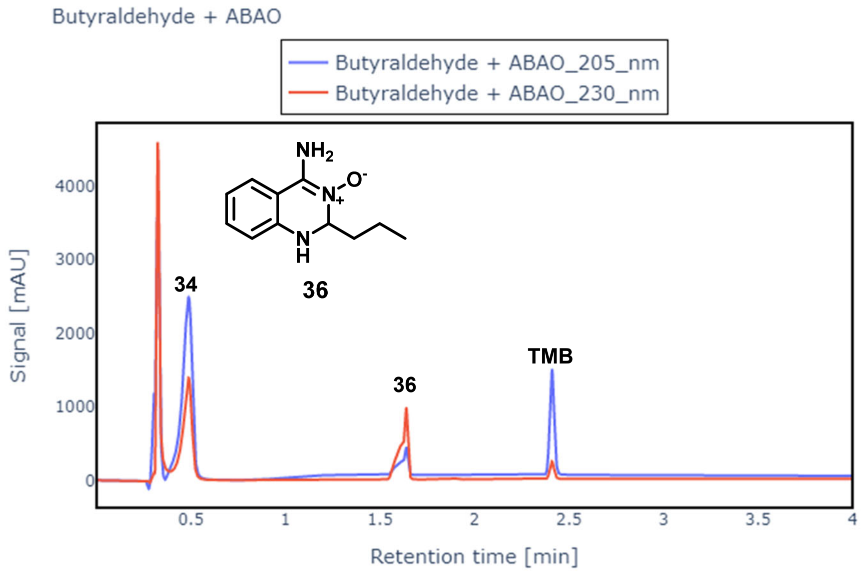

Compound	Retention time [min]
Unreacted ABAO (**34**)	0.488
Butyraldehyde-ABAO (**36**, 4-amino-2-propyl-1,2-dihydroquinazoline-3-oxide)	1.639
1,3,5-Trimethoxybenzene (**TMB**)	2.410

Figure S100. HPLC chromatogram of **36**; Method 1 (achiral, Hypersil Gold C18).

S17 Overview of molecules

Table S4. Overview of molecules used in this publication.

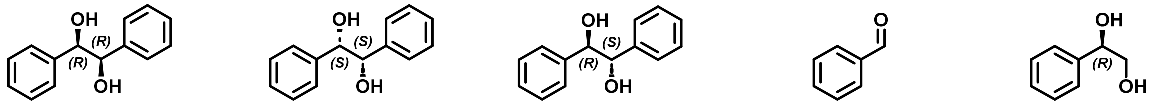

(***R***,***R***)**-1** (***S***,***S***)**-1** ***meso*-1** **2** (***R***)**-3**

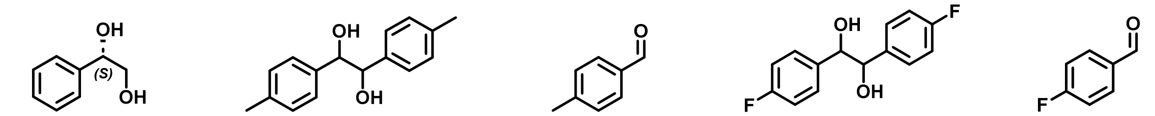

(***S***)**-3** **4** **5** **6** **7**

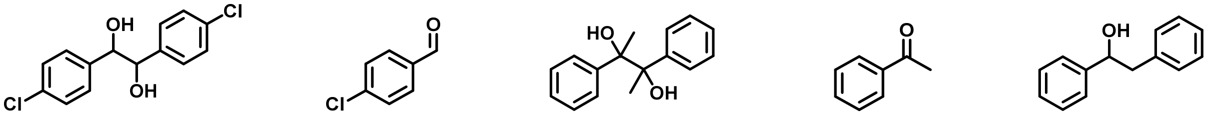

8 **9** **10** **11** **12**

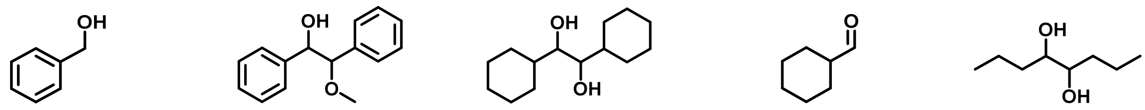

13 **14** **15** **16** **17**

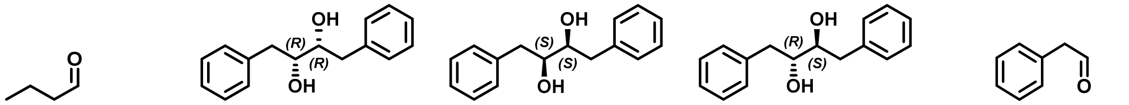

18 (***R***,***R***)**-19** (***S***,***S***)**-19** ***meso*-19** **20**

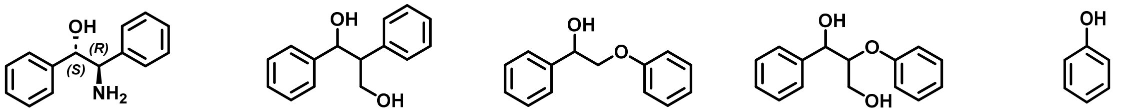

21 **22** **23** **24** **25**

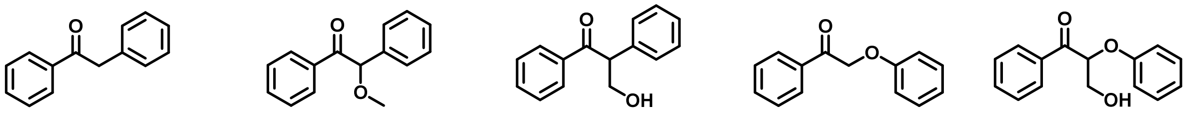

26 **27** **28** **29** **30**

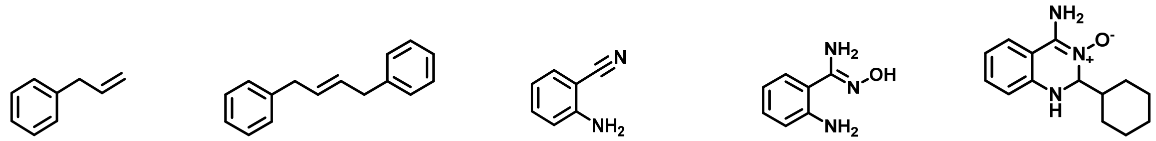

31 **32** **33** **34** **35**

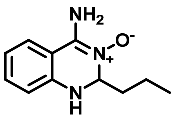

36

S18 References

(1) Gibson, D. G. Enzymatic Assembly of Overlapping DNA Fragments. In *Methods in Enzymology*, Christopher, V., Ed.; Academic Press, 2011; Vol. 498, pp 349-361.

(2) Heydenreich, F. M.; Miljuš, T.; Jaussi, R.; Benoit, R.; Milić, D.; Veprintsev, D. B. High-throughput mutagenesis using a two-fragment PCR approach. *Sci. Rep.* **2017**, *7* (1), 6787.

(3) Gasteiger, E.; Hoogland, C.; Gattiker, A.; Duvaud, S.; Wilkins, M. R.; Appel, R. D. Protein Identification and Analysis Tools on the Expasy Server. In *The Proteomics Protocols Handbook*, Walker, J. M., Ed.; Humana Press, 2005; pp 571-607.

(4) Wehrmann, M.; Elsayed, E. M.; Köbbing, S.; Bendz, L.; Lepak, A.; Schwabe, J.; Wierckx, N.; Bange, G.; Klebensberger, J. Engineered PQQ-Dependent Alcohol Dehydrogenase for the Oxidation of 5-(Hydroxymethyl)furoic Acid. *ACS Catal.* **2020**, *10* (14), 7836-7842.

(5) Ressmann, A. K.; Schwendenwein, D.; Leonhartsberger, S.; Mihovilovic, M. D.; Bornscheuer, U. T.; Winkler, M.; Rudroff, F. Substrate-Independent High-Throughput Assay for the Quantification of Aldehydes. *Adv. Synth. Catal.* **2019**, *361* (11), 2538-2543.

(6) Pettinger, N. W.; Williams, R. E. A.; Chen, J.; Kohler, B. Crystallization kinetics of cerium oxide nanoparticles formed by spontaneous, room-temperature hydrolysis of cerium(IV) ammonium nitrate in light and heavy water. *Phys. Chem. Chem. Phys.* **2017**, *19* (5), 3523-3531.

(7) An, Q.; Wang, Z.; Chen, Y.; Wang, X.; Zhang, K.; Pan, H.; Liu, W.; Zuo, Z. Cerium-Catalyzed C–H Functionalizations of Alkanes Utilizing Alcohols as Hydrogen Atom Transfer Agents. *J. Am. Chem. Soc.* **2020**, *142* (13), 6216-6226.

(8) Bellmaine, S.; Schnellbaecher, A.; Zimmer, A. Reactivity and degradation products of tryptophan in solution and proteins. *Free Radical Biol.Med.* **2020**, *160*, 696-718.

(9) Ghesquière, B.; Gevaert, K. Proteomics methods to study methionine oxidation. *Mass Spectrom. Rev.* **2014**, *33* (2), 147-156.

(10) Kay, C. W. M.; Mennenga, B.; Görisch, H.; Bittl, R. Structure of the Pyrroloquinoline Quinone Radical in Quinoprotein Ethanol Dehydrogenase. *J. Biol. Chem.* **2006**, *281* (3), 1470-1476.

(11) Sánchez-Aparicio, J.-E.; Tiessler-Sala, L.; Velasco-Carneros, L.; Roldán-Martín, L.; Sciortino, G.; Maréchal, J.-D. BioMetAll: Identifying Metal-Binding Sites in Proteins from Backbone Preorganization. *J. Chem. Inf. Model.* **2021**, *61* (1), 311-323.

(12) Kolb, H. C.; VanNieuwenhze, M. S.; Sharpless, K. B. Catalytic Asymmetric Dihydroxylation. *Chem. Rev.* **1994**, *94* (8), 2483-2547.

(13) Meng, S.-S.; Lin, L.-R.; Luo, X.; Lv, H.-J.; Zhao, J.-L.; Chan, A. S. C. Aerobic oxidation of alcohols with air catalyzed by decacarbonyldimanganese. *Green Chem.* **2019**, *21* (22), 6187-6193.

(14) Francisco, J. A.; Earhart, C. F.; Georgiou, G. Transport and anchoring of β-lactamase to the external surface of *Escherichia coli*. *Proc. Natl. Acad. Sci. U.S.A.* **1992**, *89* (7), 2713-2717.

(15) Maurer, J.; Jose, J.; Meyer, T. F. Autodisplay: One-Component System for Efficient Surface Display and Release of Soluble Recombinant Proteins from *Escherichia coli*. *J. Bacteriol.* **1997**, *179* (3), 794-804.

(16) Schlee, S.; Reinstein, J. Characterization of ATPase Cycles of Molecular Chaperones by Fluorescence and Transient Kinetic Methods. In *Protein Folding Handbook*, Buchner, J.; Kiefhaber, T., Eds.; Wiley-VCH, 2005; pp 105-161.

(17) Wang, Z.-X. An exact mathematical expression for describing competitive binding of two different ligands to a protein molecule. *FEBS Lett.* **1995**, *360* (2), 111-114.

(18) Jumper, J.; Evans, R.; Pritzel, A.; Green, T.; Figurnov, M.; Ronneberger, O.; Tunyasuvunakool, K.; Bates, R.; Žídek, A.; Potapenko, A.; Bridgland, A.; Meyer, C.; Kohl, S. A. A.; Ballard, A. J.; Cowie, A.; Romera-Paredes, B.; Nikolov, S.; Jain, R.; Adler, J.; Back, T.; Petersen, S.; Reiman, D.; Clancy, E.; Zielinski, M.; Steinegger, M.; Pacholska, M.; Berghammer, T.; Bodenstein, S.; Silver, D.; Vinyals, O.; Senior, A. W.; Kavukcuoglu, K.; Kohli, P.; Hassabis, D. Highly accurate protein structure prediction with AlphaFold. *Nature* **2021**, *596* (7873), 583-589.

(19) Mirdita, M.; Schütze, K.; Moriwaki, Y.; Heo, L.; Ovchinnikov, S.; Steinegger, M. ColabFold: making protein folding accessible to all. *Nat. Methods* **2022**, *19* (6), 679-682.

(20) Halgren, T. A.; Murphy, R. B.; Friesner, R. A.; Beard, H. S.; Frye, L. L.; Pollard, W. T.; Banks, J. L. Glide: A New Approach for Rapid, Accurate Docking and Scoring. 2. Enrichment Factors in Database Screening. *J. Med. Chem.* **2004**, *47* (7), 1750-1759.

(21) Kim, S. M.; Kim, D. W.; Yang, J. W. Transition-Metal-Free and Chemoselective NaO^{t}Bu–O_2-Mediated Oxidative Cleavage Reactions of *vic*-1,2-Diols to Carboxylic Acids and Mechanistic Insight into the Reaction Pathways. *Org. Lett.* **2014**, *16* (11), 2876-2879.

(22) Yang, J.; Dudley, G. B. Pyridine-Directed Organolithium Addition to an Enol Ether. *Adv. Synth. Catal.* **2010**, *352* (18), 3438-3442.

(23) Nomura, R.; Matsuno, T.; Endo, T. Samarium Iodide-Catalyzed Pinacol Coupling of Carbonyl Compounds. *J. Am. Chem. Soc.* **1996**, *118* (46), 11666-11667.

(24) Liu, H.; Li, H.; Luo, N.; Wang, F. Visible-Light-Induced Oxidative Lignin C–C Bond Cleavage to Aldehydes Using Vanadium Catalysts. *ACS Catal.* **2020**, *10* (1), 632-643.

(25) Banwell, M. G.; Ma, X.; Taylor, R. M.; Willis, A. C. Concise Assembly of the Polycyclic Frameworks Associated with the Hapalindole and Fischerindole Alkaloids. *Org. Lett.* **2006**, *8* (21), 4959-4961.

(26) Gresser, M. J.; Wales, S. M.; Keller, P. A. The attempted stereoselective synthesis of chiral 2,2'-biindoline. *Tetrahedron* **2010**, *66* (34), 6965-6976.
